# Study protocol of the Diabetes and Depression Study (DAD): a multi-center randomized controlled trial to compare the efficacy of a diabetes-specific cognitive behavioral group therapy versus sertraline in patients with major depression and poorly controlled diabetes mellitus

**DOI:** 10.1186/1471-244X-13-206

**Published:** 2013-08-06

**Authors:** Frank Petrak, Stephan Herpertz, Christian Albus, Norbert Hermanns, Christoph Hiemke, Wolfgang Hiller, Kai Kronfeld, Johannes Kruse, Bernd Kulzer, Christian Ruckes, Matthias J Müller

**Affiliations:** 1 Department of Psychosomatic Medicine and Psychotherapy, LWL-University Clinic Bochum, Ruhr-University Bochum, Bochum, Germany; 2 Department of Psychosomatic Medicine and Psychotherapy, University of Cologne, Köln, Germany; 3 Diabetes Center Mergentheim, Bad Mergentheim, Germany; 4 Department of Psychiatry and Psychotherapy, University Medical Centre, Johannes Gutenberg-University, Mainz, Germany; 5 Department of Clinical Psychology and Psychotherapy, Johannes Gutenberg University Mainz, Mainz, Germany; 6 Interdisciplinary Centre for Clinical Trials Mainz (IZKS Mainz), University Medical Centre, Johannes Gutenberg-University, Mainz, Germany; 7 Clinic for Psychosomatic and Psychotherapy, University Clinic Gießen/Marburg, Justus-Liebig-University Gießen, Marburg, Germany; 8 Vitos Clinical Centre Gießen-Marburg and Justus-Liebig-University Gießen, Marburg, Germany

## Abstract

**Background:**

Depression is common in diabetes and associated with hyperglycemia, diabetes related complications and mortality. No single intervention has been identified that consistently leads to simultaneous improvement of depression and glycemic control. Our aim is to analyze the efficacy of a diabetes-specific cognitive behavioral group therapy (CBT) compared to sertraline (SER) in adults with depression and poorly controlled diabetes.

**Methods/Design:**

This study is a multi-center parallel arm randomized controlled trial currently in its data analysis phase. We included 251 patients in 70 secondary care centers across Germany. Key inclusion criteria were: type 1 or 2 diabetes, major depression (diagnosed with the Structured Clinical Interview for DSM-IV, SCID) and hemoglobin A1_C_ >7.5% despite current insulin therapy. During the initial phase, patients received either 50–200 mg/d sertraline or 10 CBT sessions aiming at the remission of depression and enhanced adherence to diabetes treatment and coping with diabetes. Both groups received diabetes treatment as usual. After 12 weeks of this initial open-label therapy, only the treatment-responders (50% depression symptoms reduction, Hamilton Depression Rating Scale, 17-item version [HAMD]) were included in the subsequent one year study phase and represented the primary analysis population. CBT-responders received no further treatment, while SER-responders obtained a continuous, flexible-dose SER regimen as relapse prevention. Adherence to treatment was analyzed using therapeutic drug monitoring (measurement of sertraline and N-desmethylsertraline concentrations in blood serum) and by counting the numbers of CBT sessions received. Outcome assessments were conducted by trained psychologists blinded to group assignment. Group differences in HbA1c (primary outcome) and depression (HAMD, secondary outcome) between 1-year follow-up and baseline will be analyzed by ANCOVA controlling for baseline values. As primary hypothesis we expect that CBT leads to significantly greater improvement of glycemic control in the one year follow-up in treatment responders of the short term phase.

**Discussion:**

The DAD study is the first randomized controlled trial comparing antidepressants to a psychological treatment in diabetes patients with depression.

The study is investigator initiated and was supported by the ‘Förderprogramm Klinische Studien (Clinical Trials)’ and the ‘Competence Network for Diabetes mellitus’ funded by the Federal Ministry of Education and Research (FKZ 01KG0505).

**Trial registration:**

Current controlled trials ISRCTN89333241.

## Background

### Diabetes and depression

Diabetes mellitus is a highly prevalent chronic disease affecting approximately 9 to 10% of the global adult population [1]. Based on estimates of the International Diabetes Federation there were 366 million people with diabetes in 2011, and this rate is expected to rise to 552 million by 2030 [2]. Diabetes is a major cause of morbidity and mortality [3] and extremely costly in economic terms [4]. People with diabetes are at twice the risk of developing depression compared with control groups without diabetes. Accordingly, 9% of patients with diabetes meet the criteria for major depression (controls: 5%) and approximately 25% of patients with diabetes mellitus are suffering from single or multiple depression symptoms (controls: 14%), which includes major depression as well as subclinical depression [5].

There is a strong body of evidence for multiple adverse interactions between diabetes and depression. Overall, the comorbidity of depression and diabetes is associated with adverse diabetes outcomes, especially higher hemoglobin A1c (HbA1c) levels [6], micro- and macrovascular complications [7], mortality [8,9], greater diabetes-related symptom burden [10], increased functional impairment, and poorer adherence to diet, exercise, and diabetes self-management [11]. In addition, coexisting depression in people with diabetes is associated with poorer mental and physical quality of life [12] and markedly increased health care costs [13,14].

### Treatment of depression in diabetes

According to the above mentioned results, the treatment of comorbid depression is considered as essential for the clinical care of diabetes patients [15]. When treating depressed diabetes patients, there are two major treatment goals:

(1) remission or improvement of depression, and

(2) improvement of the often poor glycemic control. The second goal is generally considered as fundamental to the management of diabetes, in order to prevent or delay long-term complications [16].

Up to now, three different approaches to treat depression in diabetes have been evaluated in randomized controlled trials (RCT): Antidepressant medication, psychological treatments (e. g. cognitive behavioral therapy, counseling, problem solving training) and a flexible combination of both in stepped care approaches. In 2010, treatment effects were summarized in a meta-analysis of 14 RCTs [17] and in 2012 in a systematic Cochrane review including 19 RCTs [18].

As a result of both analyses there is growing evidence that depression can be treated with moderate success in patients with diabetes using either psychological, pharmacological and stepped care interventions (combined effect sizes [ES] d −0.51; 95% confidence interval [CI] -0.63 to −0.39). With regard to the goal of improved glycemic control the results are controversial: while the meta-analysis calculated small combined effect sizes for all treatments taken together (ES d −0.27; 95% Kl −0.40 to −0.15) [17], the Cochrane review concluded that ‘*glycemic control improved moderately in pharmacological trials, while the evidence is inconclusive for psychological intervention. Overall, the evidence is sparse and inconclusive due to several low-quality trials with substantial risk of bias and the heterogeneity of examined populations and interventions*’ [18].

In sum, despite the relevance of the topic and the increased research activity over the last two decades, there is still a considerable lack of knowledge when facing the question to identify the most effective treatment for depression in diabetes. Especially, due to the combination of interventions in the collaborative care trials with sound methodology, e.g. [19-21], it is impossible to identify the effective components of compound treatments or to evaluate the superiority of one treatment (e.g. antidepressants) above another (e.g. cognitive behavioral therapy, problem solving training). Finally, there exist no published data to identify the best treatments among effective treatments; and more specifically, to our knowledge no trial was conducted comparing pharmacological to psychological interventions in patients with diabetes and comorbid depression.

To address this lack of knowledge we conducted the Diabetes and Depression Study (DAD study), a randomized, multicenter, controlled trial comparing a diabetes-specific CBT to SER in poorly controlled diabetes patients with major depression.

#### Current status of the trial

Data analysis phase.

### Research objectives and endpoints

#### Primary objective

The aim of the planned study is to evaluate the efficacy of a diabetes-specific CBT combined with diabetes education versus a continuous treatment with SSRI (SER) combined with diabetes education in high-risk patients with poorly controlled insulin-treated type 1 or type 2 diabetes mellitus and depression.

#### Principal research question

Does a diabetes-specific psychotherapy (CBT) or antidepressant medication (SER) improve long-term glycemic control in patients with poorly controlled diabetes and depression?

#### Primary hypothesis

CBT leads to a better improvement of glycemic control when compared with SER at the one-year follow-up in patients who initially responded to short-term therapy (CBT or SER) with regard to improvement in depression.

Primary endpoint: Change of glycemic control (hemoglobin A1C).

#### Secondary objectives

To provide clear scientific evidence whether the two most widely established treatments (SSRI and CBT) for patients with depression but without additional somatic diseases can be applied to high-risk patients with poor metabolic control of insulin-treated diabetes and comorbid depression.

#### Secondary hypothesis

CBT and SER are both effective in terms of remission of depression after 12 weeks in an open label trial as well as at the one-year follow-up.

### Secondary endpoints

(a) remission of depression: no longer fulfilling the DSM-IV-TR criteria for depression according to the Structured Clinical Interview for DSM-IV, SCID [22], and depression score according to the HAMD –Interview [23] < = 7;

(b) improvement of depression (>/= 50% reduction of (HAMD-baseline score);

(c) improved generic HRQoL, per SF-36 [24]; and

(d) decreased problems in daily living with diabetes, per PAID [25].

## Methods/Design

### Study design, setting, recruitment and procedures

**Figure 1:**
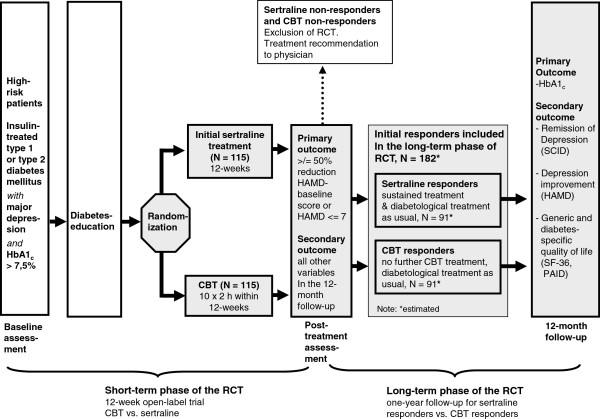
Design of the DAD study.

This trial is a prospective, randomized, multicenter, parallel arm controlled clinical trial that involved the two treatment conditions CBT vs. SER in a parallel group design (see Figure 1).

The recruitment of the patients took place between April 2006 and May 2009 in 70 trial centers of outpatient secondary care (specialized diabetological practices and ambulatory care health services in clinics) located in different parts of Germany (predominantly in the Rhine-Main area, Ruhr area and Düsseldorf/Köln). Four coordinating trial centers (Bochum/Dortmund, Mainz, Düsseldorf/Köln and Bad Mergentheim) organized the recruitment activities in the diabetological trial centers located in their vicinity. All insulin-treated outpatients with diagnosed type 1 or type 2 diabetes registered in the respective diabetological trial centers within the age range of 21 to 69 years and with an HbA1_c_ value >7.5% received a letter by their diabetologist with a patient information leaflet about the trial. They were invited to participate and informed that they would receive a telephone call by a research assistant. During the telephone call, the research assistant provided further information about the trial and asked consent for the baseline assessment, which then took place in one of the diabetological trial centers. After having provided written informed consent, medical eligibility criteria of a patient was checked by his or her diabetologist.

Psychological eligibility criteria such as depression, excluded history of schizophrenia etc. were assessed by clinical psychologists who had undergone an intensive standardized training provided by one of the authors (MJM). Depression was measured in a two stage screening-procedure starting with a questionnaire-based screening (German version [26] of the Centre for Epidemiologic Studies Depression Scale (CES-D) [27], followed by the Structured Clinical Interview for DSM-IV (SCID) [22] for those patients who were screened positive. Severity of depression was measured with the HAMD. To assure the quality of the HAMD training we first established an expert standard rating (which consisted of an agreement of three highly experienced raters). During the training we computed chance-corrected coefficients (weighted κ) of rating agreement with the expert standards for the single items and the total score of the HAMD. Item ratings within the accepted range of the standard rating ± 1 obtained a full credit of 1 and ratings outside the accepted range a weight of 0 (no credit). Values of κw > 0.40 denote acceptable chance-corrected agreement, coefficients κw > 0.80 indicate almost perfect strength of agreement [28]. High interrater reliability (κ>0.80) was achieved for most of the HAMD items and excellent interrater reliability was demonstrated for the total score (κ_w_ 0.96 ± 0.08), thus indicating that the raters were well trained before the beginning of their assessment in the DAD study. Further psychosocial variables were assessed using psychometric questionnaires (described in the ‘Measures’ section).

Patients meeting all eligibility criteria (see ‘Eligibility criteria’ section) were offered a short structured diabetes education (2 × 3 hours) by trained diabetes educators as an update to ensure sufficient diabetes knowledge in all trial participants (see ‘Diabetes education’ section). All patients completing the diabetes education program were allocated randomly to either 10 sessions of group CBT or an algorithm based SER- treatment with 50 to 200 mg/day according to treatment response and side effects (see ‘Randomization and blinding’ section and ‘Interventions’ section). Diabetes treatment was not part of the trial protocol and was continued ‘as usual’.

The trial consisted of a 12-week open label therapy short-term phase. Treatment responders of both groups (≥ 50% reduction of the HAMD baseline score or HAMD post-treatment score ≤ 7) were included in a 12-month long-term phase. These patients constituted the intention-to-treat (ITT) population. Non-responders of the short-term phase were excluded from the treatment protocol and referred to the best available treatment using standard medical care. The general recommendation to the treating physicians was to give SER or other medication to CBT-non-responders and to give CBT or other medication to SER- non-responders.

All patients entering the long-term phase received ongoing diabetological treatment as usual by their treating physicians in the diabetological trial center at 3-month intervals during the following 12 months. SER-responders received a continuous SER- treatment as relapse prevention. CBT responders did not receive further treatment but were encouraged to work with a patients manual in the sense of a bibliotherapy [29] during the one year follow-up phase. The difference in the active treatment duration between both interventions corresponds to usual clinical practice and thus assure external validity. Generally, group CBT is offered for a limited period of time assuming that ‘carry over’ effects will stabilise the results [30,31], while SER in patients responding to the treatment is given for a longer period of time as relapse prevention (see also German National Disease Management Guidelines [32]). In order to control for the amount of physician contact patients of both groups underwent the same number of visits. During the visits, the CES-D questionnaire was applied and the HbA1_c_ value was assessed. For the patients treated with SER, additional analyses of SER and its major metabolite (N-desmethylsertraline) in blood serum were performed as measurement of treatment adherence (see ‘Assessment of adherence’ section).

Drug analyses were performed as described previously using a validated high performance liquid chromatographic method with column switching and spectrophotometric detection [33]. Intra- and interassay variabilities were below 10%.

At the 12-month follow-up, both treatment groups were re-examined regarding the primary outcome variable HbA1C. In addition, SCID and HAMD interviews as well as psychometric questionnaires were administered among others to evaluate the treatment effects regarding depression, health-related quality of life, and disease-specific burdens.

### Eligibility criteria (inclusion and exclusion criteria)

Initially, all insulin-treated diabetes patients within the age range of 21 to 65 years, with an HbA1_c_ value > 8% and major depression according to DSM-IV criteria were to be included, provided that they also met the other inclusion and exclusion criteria. Two eligibility criteria and two exclusion criteria were revised and amended to the protocol in August 2006 and April 2007. The age range was changed from originally 21–65 years to 21–69 years to increase the number of eligible subjects. The inclusion criterion of HbA1_c_ > 8% was changed into >7.5%, because we faced serious difficulties to find poorly controlled patients in the secondary care recruitment centers. As an explanation for this unexpected situation we think that the successful introduction of disease management programs (DMP) in Germany at that time might have led to a decrease in HbA1_c_ values in patients with type 2 diabetes [34,35]. Moreover, we assumed that patients with very poorly controlled type 2 diabetes would have still been treated in primary care settings not participating in the DMPs.

To enhance comparability with international studies and to prevent a selection bias, our independent scientific advisory board (see ‘Quality assurance’ section) recommended excluding patients only in case of clinically significant suicide risk or history of attempted suicide in the past 12 months. The exclusion criterion regarding the current use of psychotropic drugs was modified to allow the inclusion of patients treated with low potency neuroleptics in low doses (less than 300 mg chlorpromazine dose equivalents per day), as these drugs are often used to treat sleeping disorders and restlessness in diabetes patients, particularly in those suffering from diabetic neuropathy. Detailed inclusion and exclusion criteria are listed in Table 1.

**Table 1:** Eligibility criteria for the DAD study

**Inclusion criteria**	
-	Type 1 or type 2 diabetes mellitus diagnosed at least 12 months before entering the trial
-	Insulin treatment for at least the preceding 6 months
-	21 to 69 years of age (*amended to protocol in August 2006*) Initially: 21 to 65 years of age
-	Poor glycemic control: HbA1_c_ level > 7.5% within the nine preceding months, at least 3 months before screening and again in screening measurement (*amended to protocol in August 2006/April 2007) Initially: HbA1c value > 8% twice between the preceding 9 months*
-	Current major depression (DSM-IV-TR criteria)
-	Residence near the coordination institution where CBT treatment will take place (<1 hour access)
-	Ability of subject to understand character and individual consequences of clinical trial
-	Women with child bearing potential in the sertraline group: Women will be informed that women with childbearing potential in the sertraline group should use highly effective birth control methods (e.g. combined oral contraceptives, implants)
-	Written informed consent
**Exclusion criteria**	
-	Clinically significant suicide risk or history of attempted suicide in the past 12 months (*amended to protocol in April 2007) Initially: Clinically significant suicide risk or history of attempted suicide*
-	History of schizophrenia or psychotic symptoms
-	Bipolar disorder
-	Organic brain syndrome or dementia
-	Alcohol or substance abuse or dependence in the past 6 months
-	Insufficient ability to understand German
-	Psychotherapy in the preceding 3 months
-	Pregnant or lactating patient
-	History of convulsion or seizure disorder
-	Current use of mood stabilizers, neuroleptics, antidepressants, or benzodiazepines except for
	(1) continuation of unchangeable stable amitriptyline given to treat painful diabetic neuropathy up to 50 mg per day
	(2) short-term use of benzodiazepines (less than 2 weeks)
	(3) low-potency neuroleptics in low doses, i.e. less than 300 mg chlorpromazine dose equivalents/day (*amended to protocol 08/2006*)
	Continuation of stable treatment with thyroid hormones is also permitted
-	Significant liver enzyme elevations: SGOT (aspartate aminotransferase, AST) or SGPT (alanine aminotransferase, ALT) above 3-fold of normal upper limits or significant other laboratory findings (physician’s decision)
-	Pre-treatment with reversible MAO inhibitors within the past 2 weeks or current, unchangeable co-medication with tryptophan, fenfluramine, or serotonin agonists (triptans)
-	History of hypersensitivity to the investigational medicinal product or to any drug with similar chemical structure or to any excipient present in the pharmaceutical form of the investigational medicinal product
-	Participation in other clinical trials and observation period of competing trials, respectively

Note: All changes in eligibility criteria were amended to the protocol during the trial and before breaking of the blinding.

### Randomization and blinding

#### Randomization

Patients were randomized using block randomization to ensure equal group sizes. For each coordinating institution, a separate randomization procedure stratified by type of diabetes was performed to ensure balanced treatment groups across and within coordinating institutions. Randomization lists were generated by the Interdisciplinary Center for Clinical Trials Mainz (IZKS), University Medical Center Mainz by means of a computer program. These randomization lists included the patient identification number (SUBJID) used for identifying all data collected from the subject during the study and for the treatment allocation. The randomization lists were maintained by the IZKS. The treatment allocation at the coordinating institutions was performed using FAX randomization. After checking subject’s eligibility, the investigator faxed a randomization request form to the IZKS randomization center. This form included information on the patient’s sex, year of birth and type of diabetes. The IZKS randomization center entered the SUBJID and treatment allocation on the request form and faxed the form back to the investigator’s site.

#### Blinding

In the DAD study, psychotherapy (CBT) was compared to medication and there was no placebo condition. Thus, a blinding of patients or health care providers was not possible. However, a blinding of all members of the research team involved in the assessment of outcomes was carried out.

### Interventions

#### Diabetes education

Given the poor glycemic control of the patients and considering that most patients treated with insulin had likely undergone diabetes education previously (according to the German evidence based guidelines that were in force at the time of the patient recruitment [36]), a short diabetes education program was offered to all patients as an update. Patients received structured diabetes education (2 × 3 hours) before randomization. The program included the following modules: (1) insulin treatment; (2) self-monitoring with regard to diabetes-relevant behavioral strategies (e.g., nutrition, physical activity, foot care); (3) the recognition, treatment, and prevention of acute complications; and (4) risk factors (e.g., hyperlipidemia, arterial hypertension, smoking) for macroangiopathic diabetes-related illnesses. This diabetes education was offered by trained diabetes educators using a manual that was established for the DAD study (see Additional file 1 ‘*Structured diabetes education manual for diabetes educators*’ in German).

#### Diabetes specific cognitive behavioral group therapy (CBT)

CBT was offered as manualized diabetes-specific treatment delivered by clinical psychologist who had undergone a systematic training regarding the manual. Each session was videotaped and a random sample of 13% of the sessions (N = 32 sessions) was used in order to continuously evaluate and ensure the adherence to the manual and to give continuous supervision by one of the authors, who is a CBT trainer and supervisor (FP). We additionally developed and evaluated a rating system to assess the expertise of the therapists with acceptable to good interrater reliability (intraclass correlation coefficient, ICC 0.73 to 0.96). A second rating system to assess adherence to the CBT manual was also developed and showed good to excellent interrater reliability (coefficient 0.74 to 1.0) (details of the rating systems will be published separately).

CBT was delivered in groups of 4 to 10 patients per group in an outpatient setting within a 12–week time period. This treatment consisted of 10 sessions (20 hours) using a manualized semi-structured CBT for depression, including different diabetes-specific aspects in order to improve adherence to diabetes treatment and coping with diabetes (see Additional file 2 ‘*Diabetes-specific CBT manual for therapists*’, in German). The psychoeducation section (sessions 1–3) included information about the association of mood and activities and the development and maintenance of depression. In addition, participants learned about the link between diabetes and mood and ways to influence impaired mood with cognitive techniques. Furthermore, participants were encouraged to discuss diabetes-specific goals such as HbA1_c_ target values with their diabetologists and to specify behavioral goals in order to improve their glycemic control (see Additional file 3 ‘*Working sheet for diabetologists and patients: strategies to improve glycemic control*’, in German). Individual goal achievement was assessed in sessions six and ten and possible barriers to the goal attainment were identified and modified if possible. To help patients identifying and reducing perceived barriers to various aspects of self-management and to enhance coping skills, the problem-solving section (sessions 4–7) covered cognitive and behavioral techniques (e.g. cognitive restructuring, stress management). Fear of diabetes complications and appropriate coping-strategies were topics of further sessions. In the last session participants learned how to prevent and cope with depression relapse. Each participant received a patient workbook including theoretical background, worksheets, and exercises for each session (see Additional file 4 ‘*working book for patients of the diabetes-specific CBT group*’, in German). Patients were encouraged to continue working with the book after the end of the short-term phase to stabilize and generalize the improvement in the sense of a bibliotherapy [29].

#### Sertraline treatment

SER treatment was started at a dose of 50 mg/day in the morning following an algorithm which is described in Table 2. If no clinical response was achieved within 2–4 weeks, the dose could be raised to 100 mg/day in the morning. Further dose escalation up to 200 mg per day was possible with changes not exceeding 50 mg/ week. Recommendation for dose changes according to response and side effects were based on the Clinical Global Impression Rating (CGI) [37] and the UKU side effects rating scale [38]. Patients and clinician’s ratings of side effects and treatment response as well as the current course of treatment (duration of treatment and changes) were taken into account. Optional supplementary visits and telephone contact were possible at any time. Additionally one of the authors, a psychiatrist and clinical psychologist (MJM), offered advice to the treating diabetologists providing a psychiatric hotline if questions regarding the medication occurred. During the long-term phase of the trial SER responders received a continuous SER treatment as relapse prevention.

**Table 2:** Algorithm for the sertraline treatment in the DAD trial

	**No improvement (< 20%)**			**At least minimal improvement**		
	**(CGI = 0)**			**(CGI 1 to 3)**		
**UKU side effects**	**50 mg**	**75-175 mg**	**200 mg**	**50 mg**	**75-175 mg**	**200 mg**
Absent (0) mild (1)	⇔ or ⇧	⇔ or ⇧	⇔ or **x**	⇔ or ⇧	⇔ or ⇧	⇔
Moderate (2) severe (3)	**x**	⇩	⇩	**x**	⇩	⇩

Note: Moderate/severe side effects are side effects that impairs the patient ⇧ Increase dose in 25-50 mg/day steps, ⇩ reduce dose in 25-100 mg/day steps, ⇔ wait and see without change in dose (generally up to 4–6 weeks), **x** = consult psychiatric hotline and decide how to procede afterwards or discontinue the medication without further consultation. The decisions should always be based on the tolerability as assessed by the treating physician with the UKU, the treatment response assessed by the physician using the CGI rating, and treatment effects and tolerability as judged by the patient. In the end, the decision has to be made by the treating physician based on the algorithm recommendations and the judgement of the patients.

#### Measures

**Figure 2:**
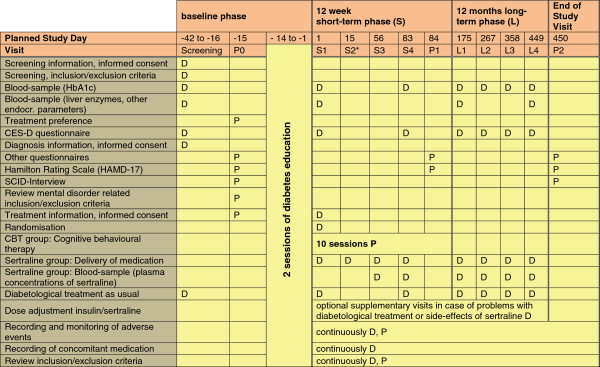
**Measurements in the DAD study.** Note: P = visit to the coordinating institutions were questionnaire and interviews will be administered by members of the research team (research assistants, psychologists); D = visit to the treating physician or research assistants in the diabetologic trial centres. *Visit S2 is optional by telefone or personally.

Outcome measures were assessed at baseline, at the end of the short-term phase (12 weeks), at months 6, 9, 12 during the long-term phase and at the end of the long-term phase (15 months). For patients randomized to SER treatment, additional assessments were scheduled at week 2 and 8 during the short term phase to deliver medication and monitor the response and side-effects. At week 8 and 12 SER and desmethylsertraline blood levels were assessed. Figure 2 summarizes all medical and psychosocial variables, which were assessed in the current trial, by time of data collection. Adverse events were monitored and recorded continuously during the trial (see Additional file 5 ‘*Safety manual*’, in German; and ‘*Definitions and assessment of adverse events*’).

#### Primary outcome

Initially, the primary outcome was ‘improvement of glycemic control’, defined as a decrease of at least 1% in HbA1_c_ value (yes/no) from baseline to the end of the long-term phase. Due to an advice of the advisory board, the analysis of the primary outcome parameter was changed and amended to the protocol in November 2008 into ‘change of glycemic control’, defined as the difference in HbA1_c_ value from baseline to the end of the long-term phase. By changing the dichotomous endpoint into a continuous endpoint, the statistical power of the trial could be enhanced and the planned number of trial subjects had to be reduced (see ‘Sample size calculation’ section).

#### Secondary outcomes

Secondary outcomes were ‘remission of depression’; i.e. not fulfilling the DSM-IV-TR criteria for depression according to the SCID and HAMD scores ≤ 7, ‘improvement of depression’, i.e. a reduction of the HAMD score from baseline to the end of trial by at least 50%, ‘improvement of health-related quality of life (HRQoL)’, i.e. change in generic HRQoL from baseline to end of trial as assessed per SF-36 mental and physical component scores [24], and ‘improvement of diabetes related stress’, from baseline to end of trial as measured per PAID [39]. As amended to the protocol, the previous primary outcome (improvement of glycemic control of at least 1% in HbA1_c_ value from baseline to the end of the long-term phase) was assessed as a secondary outcome.

#### Other psychometric measures

A number of other psychometric variables were investigated and measured with validated German questionnaires or German versions of internationally validated questionnaires at baseline, after the end of the short-term phase and in the one year follow-up: Diabetes knowledge was measured with a Diabetes Knowledge Questionnaire for Type 1 (Diabetes-Wissens-Test Typ-I -, DWT-I) [40] and Type 2 Diabetes (Diabetes-Wissens-Test Typ-II, DWT-II) [41], diabetes self-care by the Summary of diabetes self-care activities measure (SDSCA) [42], diabetes-specific locus of control by the IPC-Diabetes-Questionnaire (IPC-D1) [43] and psychological barriers to insulin therapy by the Barriers to Insulin Therapy Questionnaire (BIT) [44]. Coping was assessed using the Freiburger Coping with Illness Questionnaire (15-item version, FKV-15) [45], self-esteem by the Rosenberg Self-Esteem Scale (RSE) [46]; hopelessness by the Hopelessness Scale (H-Scale) [47]; incongruence by Inkongruence Questionnaire (K-INK) [48], childhood traumata by Childhood Trauma Questionnaire (CTQ) [49,50] psychological problems and symptoms of psychopathology by the Short-Form of the Symptom Checklist SCL-90-R [51] (German version, SCL-K-9) [52]; and social support by the Questionnaire of perceived family support and communication (PFUK) [53].

#### Assessment of treatment preference

Treatment preference was assessed as follows: ‘If you were depressed and you could choose between two equally effective treatments that might cure your depression, which ONE would you choose?’

(1) Free medication daily for 15 months (no or only minor side effects to be expected);

(2) 10 sessions (2 hours each, within a 3–month time period) of group psychotherapy with a maximum of 10 group members, with a focus on depression and diabetes-related problems;

(3) I do not prefere either treatment.

#### Assessment of the patient- and therapist perspectives of the (CBT) sessions

Each CBT session was rated by patients and therapists assessing the perceived personal involvement and confidence regarding the group therapy. The group therapists and the supervisor received the results as continuous feedback before the next session in order to reflect and if necessary to improve the therapeutic strategy in the respective group. The questionnaire was validated within the DAD study with good psychometric results [54].

#### Assessment of adherence to treatment

Adherence to treatment was defined by measuring the received therapy 'dose' (see Table 3 for an overview about the definition of adherence to CBT and SER treatment).

**Table 3:** Definition of treatment adherence in the CBT and sertraline treatment groups

**Adherence categories**	**CBT**	**Sertraline (randomized sample)**	**Sertraline (ITT sample)**
Adherent	Participation in ≥ 8 sessions of CBT	2 measures SER and corresponding ratio DSER:SER in target range	≥ 5 measures SER and corresponding ratio DSER:SER in target range
Partially non-adherent	Participation in 1–7 sessions of CBT	1 measures SER and corresponding ratio DSER:SER in target range	1-4 measures SER and corresponding ratio DSER:SER in target range
Non-adherent	No participation in any session of CBT	no measures SER and corresponding ratio DSER:SER in target range	no measures SER and corresponding ratio DSER:SER in target range

Note: SER: blood concentration of sertraline. DSER:SER: ratio of desmethylsertraline to sertraline concentrations. Target ranges of SER were defined as concentrations between 10–100 ng/ml [55]. Target ranges of DSER:SER were defined as 0.85–4.25, corresponding to the mean ± 2 standard deviations (M = 2.55, SD = 0.85) derived from 348 samples by Reis et al. [51].

**Adherence to CBT** was assessed by the number of attended CBT-sessions. As there exists no established measure of adherence for psychotherapy, in the current trial sufficient adherence to CBT was defined as participation in at least eight out of ten sessions.

**Adherence to SER treatment** was assessed by repeated measures of serum SER concentrations and the ratio of metabolite to parent compound, i.e. ratio of N-desmethylsertraline (DSER) to SER as recommended by Reis and colleagues [56]. As patients identified as treatment non-responders after the end of the short-term phase were excluded from the treatment protocol, SER adherence was evaluated separately for randomized patients and ITT patients. For randomized patients, adherence was assessed based on SER concentrations and DSER: SER measured at week 8 and 12. For ITT patients, adherence was assessed based on SER concentrations and DSER: SER measured at week 8, 12, 24, 36, 48 and 60. Notwithstanding initial planning and Therapeutic Drug Monitoring (TDM) guidelines [57], which recommend concentrations between 10–50 ng/ml, target ranges of SER were defined as concentrations between 10–100 ng/ml, as up to 100 ng/ml no severe adverse effects are to be expected. Furthermore, it has to be taken into account that the trial participants suffer from chronic disease and receive concomitant medications, which affect drug metabolism potentially resulting in higher serum concentrations [56]. To avoid biased adherence ratings, patients discontinuing the trial participation due to severe adverse events or discontinuing SER intake because of their physician’s recommendation, were excluded from the adherence rating. In cases of missing blood samples or concentrations not detectable due to interferences, all available blood samples for the patient were discussed with one of the authors with long-standing expertise in therapeutic drug monitoring (CH). Based on the course of available SER concentrations, DSER concentrations and ratios DSER:SER, adherence was estimated for these patients. If only one blood sample was available, the patient was excluded from the adherence rating. As high SER concentrations (> 100 ng/ml) are often observed in case of concomitant medication, SER concentrations above the target range were considered adherent, if the corresponding ratio DSER:SER fell into the target range.

In addition, a binary overall adherence rating was employed distinguishing adherent from non-adherentpartially non-adherent patients for all randomized patients and all ITT patients, respectively.

For the SER group, we also aimed at identifying overt and hidden forms of non-adherence. Non-adherence was considered overt, if patients chose to discontinue or refuse SER treatment or withdrew informed consent during the short-term phase of the trial for randomized patients and during the whole trial phase for ITT patients, respectively. All other cases of non-adherence were considered as hidden non-adherence.

### Statistical analysis

#### Sample size calculation

Initially, the power calculation was based on expected differences in the percentage of patients with 1% improvement in HbA1_c_ levels in both treatment groups. As the primary outcome variable was changed into a continuous variable due to an amendment to the protocol in November 2008, the power calculation was changed as follows: The power calculation was based on expected differences in HbA1_c_ levels of the comparison groups in the (ITT) sample. This sample consisted of all randomized patients who entered the 12-month follow-up phase. Considering RCTs in which CBT [58] or SSRIs [59] were evaluated, a treatment difference of 1.0% HbA1_c_ could be assumed as relevant. Standard deviations of HbA1_c_ values of 1.42 and 1.8 were observed in these studies. For the current trial a standard deviation of slightly more than 1.4 could be expected. However, a standard deviation of 1.6 as a conservative approach was taken. Therefore, with 2×46 evaluable subjects, the trial will have 85% power to detect a treatment difference of 1.0% HbA1_c_ by means of a t-test on a two-sided level of significance α = 0.05. Assuming a drop-out rate of 60%, 230 (2×115) subjects had to be randomized.

Analyses will be performed using an intent-to-treat principle. However, if in the short-term phase dropout rates in the two treatment groups differ by more than 10%, the primary analysis will employ all randomized patients. This would be done because the comparison of long-term treatment effects on glycemic control in patients who have an initial short-term improvement of depressive disorder (HAMD reduction ≥ 50% or HAMD score ≤ 7 after the short-term phase) is valid only if the dropout rates in the short-term phase are similar.

#### Analysis of outcome variables

No interim analyses are planned. The primary outcome of the trial ‘change of glycemic control’ is defined as the difference in HbA1_c_ value from baseline to the end of the long-term phase compared for the different treatment groups using an analysis of covariance (ANCOVA) controlling for baseline HbA1_c_ value and baseline HAMD score. The primary analysis will be focused on observed cases. The analysis will be repeated after employing the last observation carried forward method for missing HbA1_c_ and HAMD scores. An appropriate method has to be chosen on an individual basis when the missing value pattern becomes known. If more than 10% but less than 40% of the values are missing, multiple imputation methods will be considered.

The main secondary analysis is based on the numbers and proportions of patients who demonstrate improvement of glycemic control (≥ 1% improvement in HbA1_c_ values from baseline to the end of the long-term phase). Differences between treatment groups will be tested using logistic regression analysis controlling for baseline HbA1_c_ value and baseline HAMD-score. Further secondary outcomes such as remission of depression and improvement of depression will be analyzed using logistic regression analysis controlling for baseline HbA1_c_ value and baseline HAMD score to evaluate treatment effects. Differences between treatment groups in improvement in HrQoL (SF-36 mental and physical component scores) and improvement of diabetes related distress (as assessed by PAID) will be analyzed using analyses of covariance controlling for baseline HbA1_c_ values and baseline SF-36 scores (mental and accordingly physical component score) or baseline PAID scores, respectively.

All primary and secondary analyses will be repeated controlling for potential confounders. To identify confounders such as age, sex, coordinating institution, diabetes type, diabetes complications, education years, income, single/recurrent episode(s) and comorbidity with other mental disorders, a correlation analysis will be employed. Baseline-variables associated (p < .10) with long-term outcome variables (HbA1_c_, HAMD score, SF-36 component scores and PAID score, respectively) will then be included as further control variables.

According to the intent-to-treat principle all analyses will be conducted for the ITT population. The analyses for safety population, randomized population and per protocol population will be considered as secondary.

An exploratory subgroup analysis for type of diabetes will be employed for the primary outcome. Therefore, the primary analysis will be repeated including an interaction term of diabetes type and treatment group in the ANCOVA. Further covariates will be baseline HbA1_c_ and baseline HAMD score. For remission of depression from the end of the short-term phase to the end of follow-up a logistic regression including an interaction term of diabetes type and treatment group, baseline HbA1_c_ and baseline HAMD score will be performed, respectively. These analyses have to be considered exploratory.

Adherence to treatment will be displayed descriptively overall and for each treatment group. Differences in adherence between both treatment groups will be analyzed using chi-square tests. An exploratory subgroup analysis for adherence groups will be employed for change in HbA1_c_ from the end of short-term phase to the end of the follow-up. Therefore, an ANCOVA will be performed including an interaction term of adherence group and treatment group. Further covariates will be HbA1_c_ and HAMD score at the end of the short-term phase. For remission of depression from the end of the short-term phase to the end of follow-up a logistic regression including an interaction term of adherence group and treatment group, HbA1_c_ and HAMD score at the end of short-term phase will be performed, respectively. These analyses have to be considered exploratory, as the statistical power might not be sufficient.

Further exploratory secondary analyses will be performed such as the analysis, whether improvement in depression shows a stronger association with better HbA1c in the CBT group than in the on-going medication group; and, if this is the case, to test, if the degree of diabetes self-care will increase more in the CBT group than in the on-going medication group (see Additional file 6 ‘*Detailed statistical analysis plan*’).

#### Quality assurance

**Safety** Differences between treatment groups regarding rates of adverse events such newly emerging tendency will be displayed descriptively. Numbers and percentages of adverse events and number and percentage of subjects with adverse events will be presented for each occurring system organ class and for each preferred term within system organ class by treatment group and with a total column. Additionally, this will be done for relationship, intensity and seriousness of adverse events. The results will be displayed for the safety population (see Additional file 5 ‘*Safety manual*’ (in German); and ‘*Definitions and assessment of adverse events*’).

**Data management** A data management plan (DMP) was established for the DAD study to specify each data management process for the DAD trial. The purpose of the DMP is to describe the underlying data management process which governs collection, management, review and reporting of data from this clinical trial. The content of a DMP includes, but is not limited to, trial timelines, data management procedures including data collection and data flow and the Data Validation Plan. The DMP documents the validation strategy and tools, and references responsibilities for different tasks. All data management activities were performed according to the current Standard Operating Procedures (SOPs) of the IZKS (see Additional file 7 ‘*Data management plan*’).

**Monitoring** Clinical on-site monitoring was performed by personal visits from a clinical monitor according to SOPs of the IZKS. To initiate the study, the monitor visited each participating local trial site and coordinating study centers. The monitors ensured that the investigators and their staff understand all requirements of the protocol and their regulatory responsibilities. Each site was visited by the monitor at regular intervals to ensure compliance with the study protocol, GCP and legal aspects. The monitors reviewed the entries into the Case Report Forms (CRF) on the basis of the source documents. The presence of correct informed consents was checked for every patient. Source document verification (SDV) was performed for 100% of core data (randomization, primary endpoint and SAE) and 20% of other data in this study. Details were specified in the monitoring manual for the DAD study. The investigators had to allow the monitor to look at all relevant documents and were requested to provide support at all times to the monitor. By frequent communications (letters, telephone, fax), the monitors ensured that the trial was conducted according to the protocol and regulatory requirements.

**Advisory board** An independent scientific advisory board was established to supervise the conduct of the trial and to issue recommendations for early termination, modifications or continuation of the trial, if necessary.

Members of the Advisory Board are:

– Prof. Dr. Ulrich Hegerl, Clinic and Policlinic of Psychiatry and Psychotherapy of the University of Leipzig, Germany.

– Prof. Dr. Walter Lehmacher, (Institute for Medical Statistics, Informatics and Epidemiology of the University of Cologne, Germany.

– Prof. Dr. Martin Hautzinger, Eberhard-Karls-University, Institute of Psychology, Department of Clinical and Developmental Psychology, Tuebingen, Germany.

### Ethical aspects

The procedures set out in the trial protocol regarding the conduct, evaluation, and documentation of this trial are designed to ensure that all persons involved in the trial abide by Good Clinical Practice (GCP) and the ethical principles described in the current revision of the Declaration of Helsinki. Clinical monitoring, data management, pharmacovigilance, regulatory affairs and biometry according to GCP were conducted by the IZKS Mainz. The trial was carried out in keeping with local legal and regulatory requirements. The requirements of the AMG, the GCP regulation, and the Federal Data Protection Law (BDSG) have been kept. The trial was approved by Medical Ethics Committee Hessen (Ethikkommission der Landesärztekammer Hessen no. 2/2006) on 14/03/2006. The Federal Institute for Drugs and Medical Devices approved the trial on 17/03/2006. Three substantial amendments have been approved by both institutions in August 2006, April 2007 and November 2008. All patients were in treatment, regularly seen by diabetologists and psychologists. Before being admitted to the clinical trial, the patients agreed to participate after the nature, scope, and possible consequences of the trial have been explained in a form understandable to them. The patients gave written informed consent and received a copy of the signed consent document. The documents were in language that was understandable to the patients and specified who informed them. During the trial, patients were identified solely by means of year of birth and an individual identification code (patient number, randomization number).

## Discussion

Despite the increasing number of studies evaluating treatment options for depression in diabetes, the evidence for the identification of a single treatment, which could significantly improve psychological outcomes and glycemic control at the same time remains inconclusive [17,18]. Furthermore, as pointed out by Markowietz and colleagues [60], most interventions were not designed to directly improve diabetes self-care and did not evaluate effects on further outcome variables such as quality of life or remission of depression. The current trial attempts to address these limitations by including only patients with poor glycemic control (HbA1c > 7.5%), developing a cognitive behavioral therapy covering diabetes-specific aspects such as goal-setting to enhance diabetes-self-care, and by assessment of further outcome variables such as remission of depression, improvement in quality of life and reduction of diabetes-related distress. As was observed in the most recent systematic review on this topic the quality of the trials in this field is very heterogeneous and especially active comparison trials are rare [18]. Therefore, we conducted this trial comparing for the first time the efficacy of a psychological treatment to psychopharmacological treatment for depression in patients with depression with a strong emphasis on sound methodology. Thus, we think that the DAD study will add important knowledge to the field of depression treatment in diabetes.

### Status of the trial

The trial commenced in April 2006, and patient recruitment was completed in May 2009, resulting in a sample of 251 randomized patients. After reaching the calculated sample size of 230 patients, all patients already under screening have been randomized additionally. Currently we are in the analysis phase.

## Competing interests

The authors declare that they have no competing interests.

## Authors’ contributions

FP principal investigator (PI), MJM coordinating investigator, KK clinical trialist, CR biometrician. Significant contribution to the development of the study design and methods: FP, SH, NH, CH, KK; JK, BK, CR, MJM; Writing of the manuscript. FP. All co-authors read, edited, and approved the final manuscript. Responsibility for the coordination, recruitment and treatment in the coordinating regional trial centers: FP, SH, CA, NH, WH, KK, JK, BK. All authors participated in the work sufficiently to take public responsibility for their respective parts of the paper. Significant contribution to biometrics: CR, FP, MJM. Significant contribution to monitoring and quality assurance of the trial: KK, FP, MJM.

## Pre-publication history

The pre-publication history for this paper can be accessed here:

http://www.biomedcentral.com/1471-244X/13/206/prepub

## Supplementary Material

Additional file 1 Structured diabetes education manual for diabetes educators (in German). Click here for file

Additional file 2 Diabetes-specific CBT manual for therapists (in German). Click here for file

Additional file 3 Working sheet for diabetologists and patients: strategies to improve glycemic control’ (in German). Click here for file

Additional file 4 Working book for patients of the diabetes-specific CBT group (in German). Click here for file

Additional file 5 ***‘Safety manual***’ **(in German); and ‘*Definitions and assessment of adverse events*’.** Click here for file

Additional file 6 Detailed statistical analysis plan. Click here for file

Additional file 7 Data management plan. Click here for file
