# Supplementary material for: Study protocol of the Diabetes and Depression Study (DAD): a multi-center randomized controlled trial to compare the efficacy of a diabetes-specific cognitive behavioral group therapy versus sertraline in patients with major depression and poorly controlled diabetes mellitus
Source: BMC Psychiatry. 2013 Aug 6;13:206. doi: 10.1186/1471-244X-13-206 (PMC3750698; doi:10.1186/1471-244X-13-206)
Supplement: Additional file 1 — Structured diabetes education manual for diabetes educators (in German). [file 1471-244X-13-206-S1.pdf]

Diabetesschulung im Rahmen der Diabetes-Depressions-Studie

Liebe Diabetesberaterin, lieber Diabetesberater,

Sie haben sich entschlossen die Diabetesschulung der Diabetes-Depressions-Studie (DAD-Studie) durchzuführen. Deshalb möchten wir Ihnen ***wichtige Informationen*** zum Hintergrund, Zielsetzung und Ablauf der DAD-Studie geben.

Die Diabetes-Depressions-Studie (DAD-Studie) ist eine multizentrische Studie. Die Studienzentrale ist in der Westfälischen Klinik Dortmund / Ruhr-Universität Bochum. Weitere beteiligte Studienzentren sind das Diabetes Zentrum in Bad Mergentheim, das Psychologische Institut der Johannes Gutenberg-Universität Mainz und die Klinik für Psychosomatische Medizin und Psychotherapie der Heinrich Heine-Universität in Düsseldorf. Die Studie wird gefördert vom Bundesministerium für Bildung und Forschung (BMBF). Die Projektlaufzeit ist voraussichtlich vom 01.02.2006 bis 01/2009.

Hintergrund der DAD-Studie ist, dass die Komorbidität von Diabetes und Depression mit einer deutlich schlechteren Prognose des Diabetes einhergeht. Interventionen für diese Patientengruppe sind bisher nicht ausreichend evaluiert.

Zielsetzung der Studie ist eine Verbesserung der Behandlungsmöglichkeiten depressiver Diabetiker. Hierzu soll die Wirksamkeit des bereits zugelassenen Antidepressivum „Sertralin" mit einer Kognitiven Verhaltenstherapie verglichen werden. Hypothese ist, dass sich durch eine Verbesserung der Depression längerfristig auch die Stoffwechseleinstellung (gemessen am HbA1c) verbessert.

Die ***Studienteilnehmer*** sind Menschen, bei denen die Diagnose eines Diabetes mellitus vom Typ I oder Typ II seit mindestens 12 Monaten bekannt ist und die seit mindestens sechs Monaten mit Insulin behandelt werden. Sie sind im Alter zwischen 21 und 65 Jahren, leiden zusätzlich zu ihrem Diabetes an einer Depression und weisen eine schlechte Stoffwechseleinstellung auf (HbA1c zweimalig >8% in den letzten 9 Monaten).

Das ***Studiendesign*** sieht vor, die Depression der Probanden mit einer 12-wöchigen Kurzzeittherapie (entweder Kognitiven Verhaltenstherapie oder medikamentös mit dem Antidepressivum „Sertralin") zu behandeln. Beide Gruppen erhalten während der Studienteilnahme ihre Diabetestherapie wie bisher.

Diabetesschulung: Alle Studienteilnehmer erhalten vor Beginn der Behandlungsphase eine kurze Diabetesschulung. Diese dient vor allem dem Auffrischen bisher geschulten diabetesbezogenen Wissens. Da die Studienteilnehmer in erster Linie in diabetologischen Schwerpunktpraxen rekrutiert werden und bei ihnen seit mehr als 12 Monaten die Diagnose Diabetes bekannt ist, gehen wir davon aus, dass diese bereits eine ausführliche Diabetesschulung erhalten haben.

Die Schulung findet an insgesamt 2 Tagen statt und ist auf die Dauer von jeweils 3 Stunden ausgerichtet. Die Studienteilnehmer, Typ I und Typ II Diabetiker, werden gemeinsam in der Gruppe von max. 8 Personen geschult. In der Diabetesschulung sollen die Themen wiederholt, vertieft und ggf. Fragen oder Probleme besprochen werden. Da es sich bei den Studienteilnehmern um Menschen mit einer bisher unbehandelten Depression handelt, deren Konzentrationsfähigkeit meist beeinträchtigt ist, sollten die Kursstunden nicht länger als 45 Minuten dauern und zwischen den einzelnen Kursstunden jeweils eine 10 – 15 min. Pause liegen.

Da es sich um eine Diabetesschulung im Rahmen einer multizentrischen Studie handelt, ist es unbedingt notwendig, diese standardisiert durchzuführen (d.h. in allen vier Studienzentren mit den gleichen Inhalten, Umfang, Materialien und Vorgehen). Da Typ I und Typ II Diabetiker gemeinsam geschult werden, wurden die Arbeitsmaterialien aus dem Schulungsprogramm „Medias 2" (Für Typ II) und dem Schulungsprogramm von „Lilly" (für Typ I) kombiniert.

Vielen Dank für Ihre Mitarbeit!

Tag 1:	Inhalte der Kursstunden
1. Kursstunde:	**Einführung:**<br>➢ Begrüßung<br>➢ Inhaltliche und organisatorische Rahmenbedingungen<br>➢ Vorstellungsrunde<br>**Diabetesgrundlagen:**<br>➢ Erklärungsmodell für Entstehung des Typ I und Typ II Diabetes<br>➢ Insulinwirkung<br>**Selbstkontrolle:**<br>➢ Blutzuckermessung<br>➢ Blutdruckkontrolle
2. Kursstunde:	**Ernährung**<br>➢ Gesunde Ernährung<br>➢ Kohlehydrate / BE<br>➢ Glykämischer Index
3. Kursstunde:	**Grundlagen Insulintherapie**<br>➢ Insulinwirkung<br>➢ Dosisanpassung<br>➢ Spritztechnik
**Tag 2:**	
4. Kursstunde:	**Akutreaktionen: Unterzuckerung**<br>➢ Erkennen und Wahrnehmen<br>➢ Persönliche Problematik
5. Kursstunde:	**Körperliche Aktivität**<br>➢ Motivation zu körperlicher Aktivität<br>➢ Dosisanpassung an körperliche Aktivität
6. Kursstunde:	**Risikofaktoren für Folgeerkrankungen**<br>**Folgeerkrankungen**<br>➢ Rethinopathie<br>➢ Füße<br>**Kontrolle**

Kursstunde 1: Überblick

	Inhalt	Seite
**1.1**	**Einführung**	**5**
1.1.1	Begrüßung der Teilnehmer	5
1.1.2	Inhaltliche und organisatorische Rahmenbedingungen	6
1.1.3	Vorstellungsrunde	6
**1.2**	**Grundlagen des Diabetes**	**6**
1.2.1	Erklärungsmodell Typ II	8
1.2.2	Erklärungsmodell Typ I	9
1.2.3	Insulinwirkung	9
**1.3**	**Selbstkontrolle**	**10**
1.3.1	Blutzuckerselbstkontrolle	10
1.3.2	Blutdruckmessung	13

Kursstunde 1: Materialien

**Themen**	**Folien**
**1.1 Einführung**	
1.1.1 Begrüßung	„Herzlich Willkommen“
1.1.2 Inhalte	„Übersicht Inhalte“
1.1.3 Vorstellungsrunde	---
**1.2 Grundlagen**	
	„Die Zellen brauchen Zucker“
	„Insulin senkt den Blutzucker“
1.2.1 Erklärungsmodell Typ II	„Übergewicht stört die Insulinwirkung“
	„ Schlankwerden hilft“
1.2.2 Erklärungsmodell Typ I	„Fehlende Insulinproduktion“
	„Häufige Merkmale“
1.2.3 Insulinwirkung	„Was Insulin bewirkt“
**1.3 Selbstkontrolle**	
1.3.1 Blutzuckerselbstkontrolle	„Warum ist Blutzuckerselbstkontrolle wichtig?“
	„Praktische Blutzucker-Selbstkontrolle „Blutgewinnung“
	„Hinweis zur Teststreifenhandhabung“
	„Blutzuckertagebuch“
1.3.2 Bluthochdruck	„Bluthochdruck ein „leichtes oder ernsthaftes Problem“
	„Bluthochdruck: was versteht man darunter?“
	„Was sind günstige Blutdruckwerte?“
	„Den Blutdruck selbst messen: Welche Geräte gibt es?“
	„Blutdruckmessung: die klassische Methode“

Kursstunde 1: Durchführung

1.1 Einführung

1.1.1 Begrüßung

Ziele

- Schaffen einer angenehmen Atmosphäre. Die Teilnehmer sollen sich wohl fühlen.

Vorgehen

Begrüßung der Teilnehmer und Vorstellen des Kursleiters.

Folie: Herzlich Willkommen

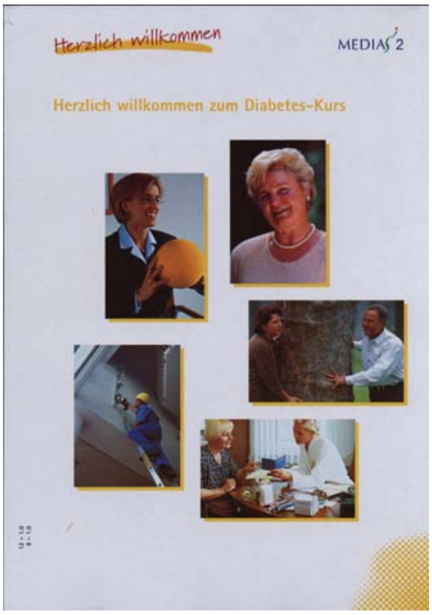

1.1.2 Inhaltliche und organisatorische Rahmenbedingungen

Ziele:

- Die Kursteilnehmer sollen über die Inhalte des Kurses informiert werden.
- Die Teilnehmer sollen jeweils zu Beginn der Kursstunde über die Themen und den Ablauf orientiert sein.

Vorgehen:

Informieren Sie die Teilnehmer über die Inhalte des Kurses und geben Sie anschließend einen Überblick über die Themen der ersten Kursstunde.

Folie: Themen des Kurses

Weisen Sie darauf hin, dass es in dem Kurs um ein „Auffrischen“ der bisher geschulten Inhalte geht.

Klären Sie anschließend mit den Teilnehmern Einzelheiten zur Organisation und zum Ablauf des Kurses (Termine, Zeitrahmen).

1.1.3 Vorstellungsrunde

Ziele

- Die Teilnehmer sollen sich untereinander kennenlernen.

Vorgehen

Freie Vorstellungsrunde: Sie bitten die Teilnehmer, sich kurz vorzustellen.

(Die Vorstellungsrunde sollte nicht zu viel Zeit in Anspruch nehmen, max. 10 Minuten).

1.2 Grundlagen des Diabetes: Erklärungsmodell Typ I und Typ II- Diabetiker

Ziele

- Die Teilnehmer sollen ein Verständnis über die Ursache des Diabetes mellitus gewinnen und sich das Zustandekommen erhöhter Blutzuckerwerte erklären können. Dazu stellen sich die Teilnehmer zunächst die Verstoffwechselung einer Mahlzeit bei einer Person ohne Diabetes vor, bevor sie im zweiten Schritt die diabetesbedingten Unterschiede in verständlichen Bildern erarbeiten.
- Die Teilnehmer sollen sich Stoffwechselvorgänge im eigenen Körper plastisch vorstellen können.
- Die Teilnehmer sollen den Unterschied zwischen Diabetes Typ I und Typ II verstehen.
- Die Teilnehmer sollen die Insulinwirkung verstehen können (Schlüssel-Schloss-Prinzip)

Vorgehen

Anhand der Folien erklären Sie zunächst die Vorgänge bei der Nahrungsverwertung bei gesunden Personen, um anschließend die Unterschiede beim Diabetes mellitus zu erarbeiten.

Stellen Sie heraus, dass der Mensch seine ganze Energie, die er zum Leben braucht, in Form der Nahrung zu sich nehmen muss. Im Folgenden stellen Sie dar, wie der Körper aus der Nahrung Energie gewinnt, die er zum Leben benötigt.

Zeigen Sie in bildhafter Form den Unterschied zwischen Stoffwechselgesunden und Menschen mit Diabetes.

Erklären Sie wie in einer gesunden Zelle die Zuckerverbrennung funktioniert:

Folie: Die Zellen brauchen Zucker

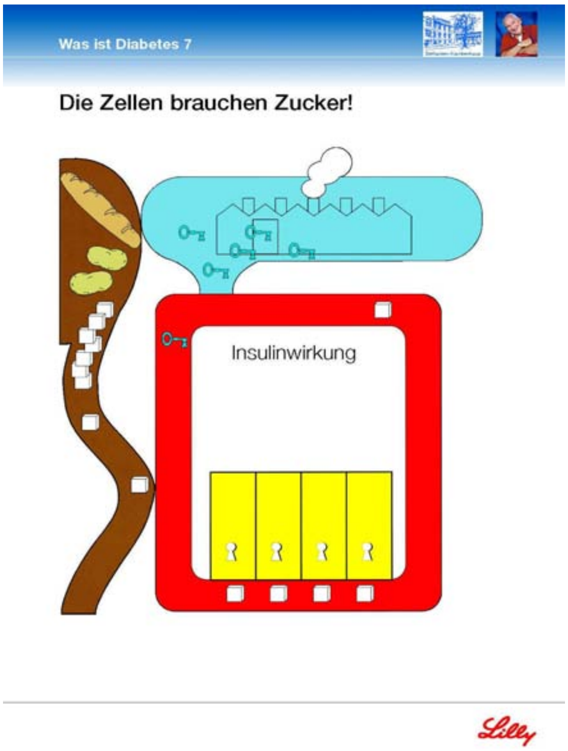

Erläutern Sie die Insulinwirkung in der Zelle:

Folie: Insulin senkt den Blutzucker

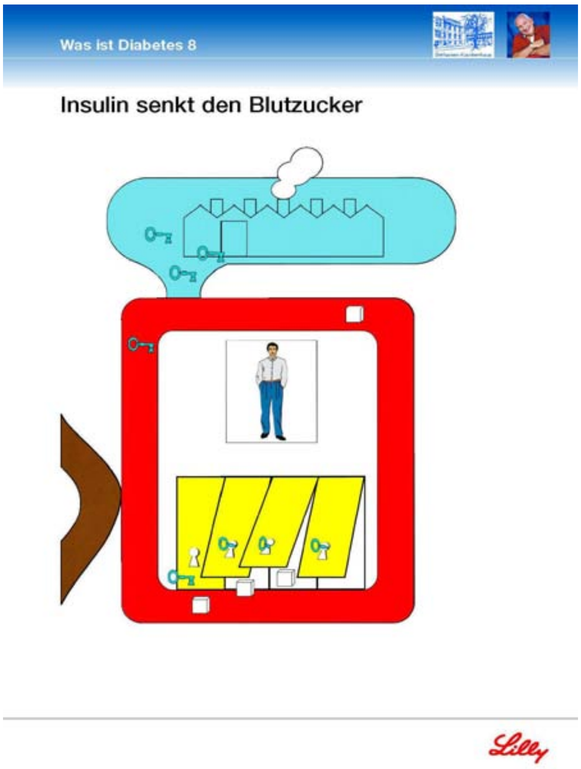

1.2.1 Erklärungsmodell: Typ II Diabetes

Erläutern Sie die Faktoren, die bei der Entstehung des Diabetes mellitus Typ II eine Rolle spielen.

Folie: Übergewicht stört die Insulinwirkung

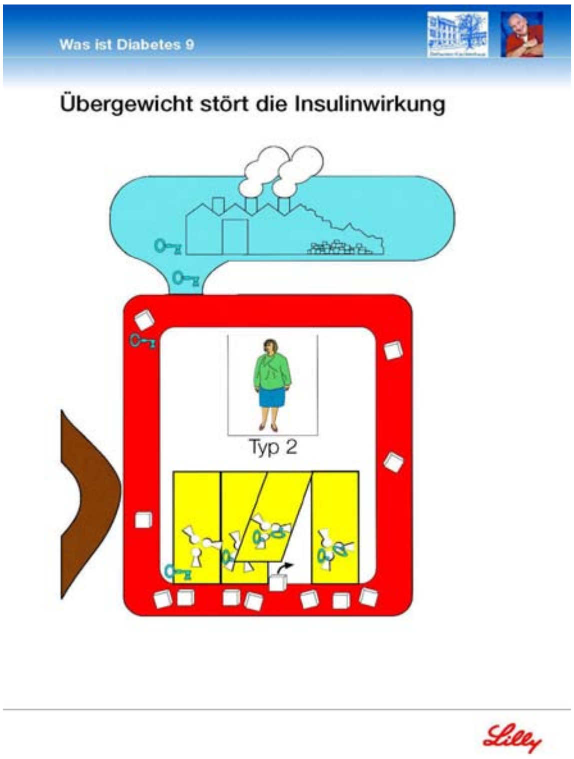

Regen Sie im nächsten Schritt die Teilnehmer an, Möglichkeiten abzuleiten, die „Zelltüren gängiger zu machen“ um erhöhte Blutzuckerwerte zu vermeiden und das Risiko von Folgeerkrankungen zu minimieren.

Folie: Schlankwerden hilft

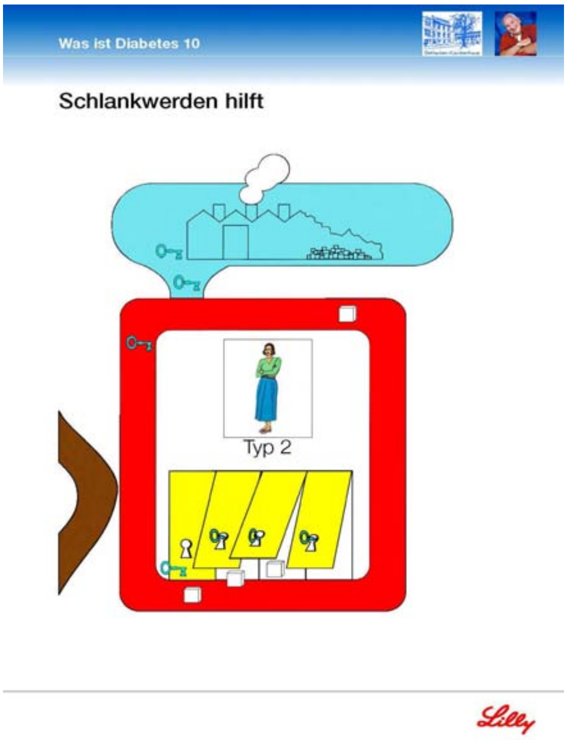

1.2.2 Erklärungsmodell: Typ I Diabetiker

Erläutern Sie die Faktoren, die bei der Entstehung des Diabetes mellitus Typ I eine Rolle spielen.

Folie: Fehlende Insulinproduktion

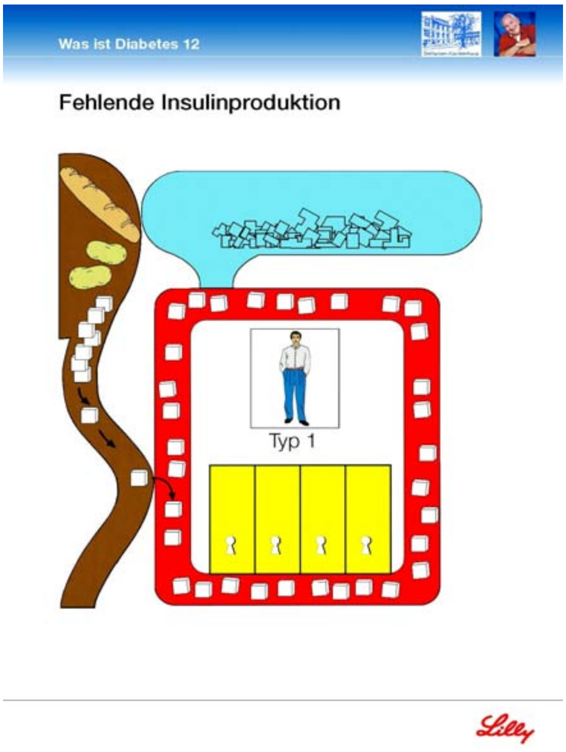

1.2.3 Insulinwirkung:

Wiederholen Sie die Wirkung von Insulin an der Zelle.

Folie: Was Insulin bewirkt

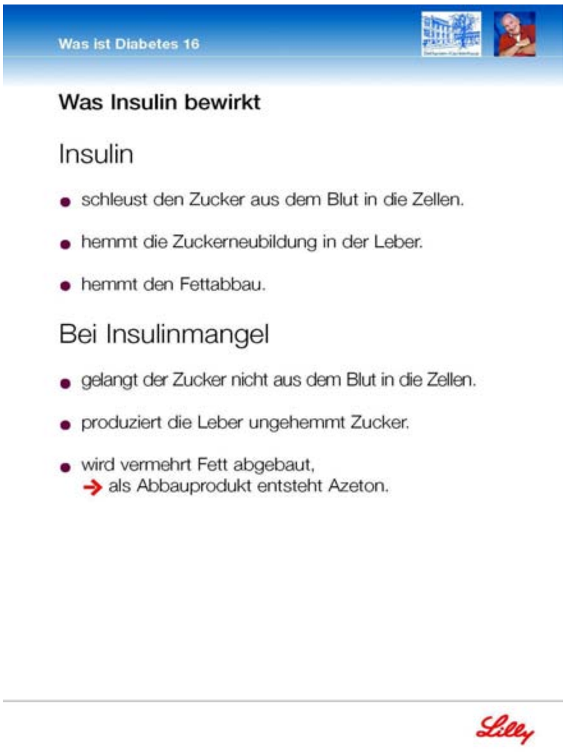

Merkmale des Diabetes mellitus: Unterschied zwischen Typ I und Typ II

Sammeln Sie mit den Patienten gemeinsam die häufigsten Merkmale des Diabetes mellitus und zeigen Sie so die Unterschiede auf zwischen Typ I und Typ II.

Folie: Diabetes mellitus – Typ?

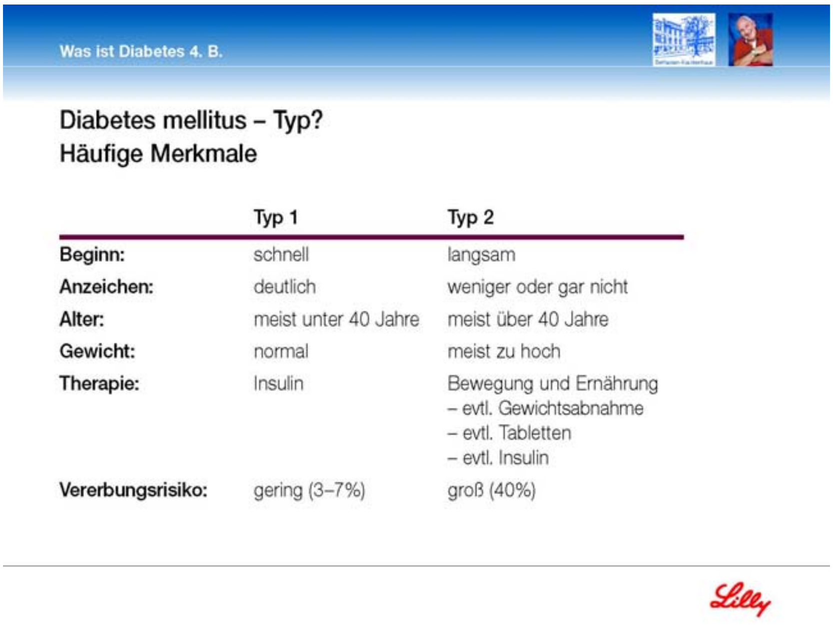

1.3 Selbstkontrolle

Ziele

Den Teilnehmern sollte deutlich werden, dass sie Risikofaktoren für diabetische Folgeerkrankungen selbst im Alltag überwachen können. Die regelmäßige Kontrolle von Blutzucker und Blutdruck kann zu Hause durchgeführt werden. Die Teilnehmer sollten motiviert werden, bei der Stoffwechselkontrolle im Alltag selbst aktiv zu werden.

Die Teilnehmer haben Gelegenheit die praktische Durchführung der Selbstkontrolle zu besprechen.

Die Teilnehmer lernen, dass regelmäßige Selbstkontrollen eine Möglichkeit darstellen, sich abzeichnende Stoffwechselentgleisungen frühzeitig zu entdecken.

1.3.1 Blutzuckerselbstkontrolle

Ziele:

- Die Teilnehmer wissen, warum Selbstkontrolle wichtig ist.
- Die Teilnehmer wissen worauf sie bei der Handhabung der Teststreifen achten müssen.
- Die Teilnehmer beherrschen die Blutzucker-Selbstkontrolle.
- Die Teilnehmer wissen, wann die Blutzucker-Kontrolle sinnvoll ist.
- Die Teilnehmer können ihr Tagebuch korrekt führen und wissen welchen Nutzen ein Tagebuch haben kann.
- Die Teilnehmer können sich entscheiden wie viele Selbstkontrollen sie langfristig durchführen möchten.

Vorgehen

Begründen Sie die Notwendigkeit und den Sinn, im Alltag Selbstkontrolle zu betreiben.

Folie: Warum ist Blutzuckerselbstkontrolle wichtig?

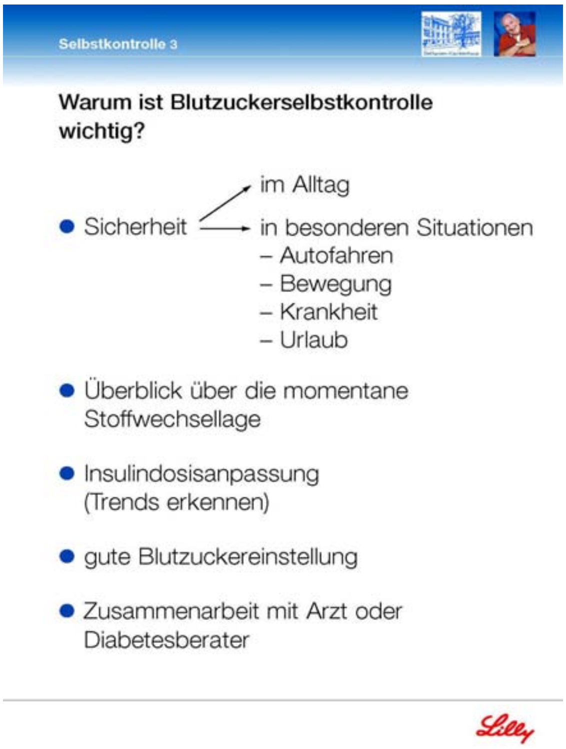

Besprechen Sie die praktische Blutzuckerkontrolle:

Wiederholen Sie mit den Teilnehmern die Durchführung der Blutzuckerselbstkontrolle und erläutern Sie mögliche Fehler bei der Blutzuckermessung per Gerät.

Folie: Praktische Blutzuckerkontrolle

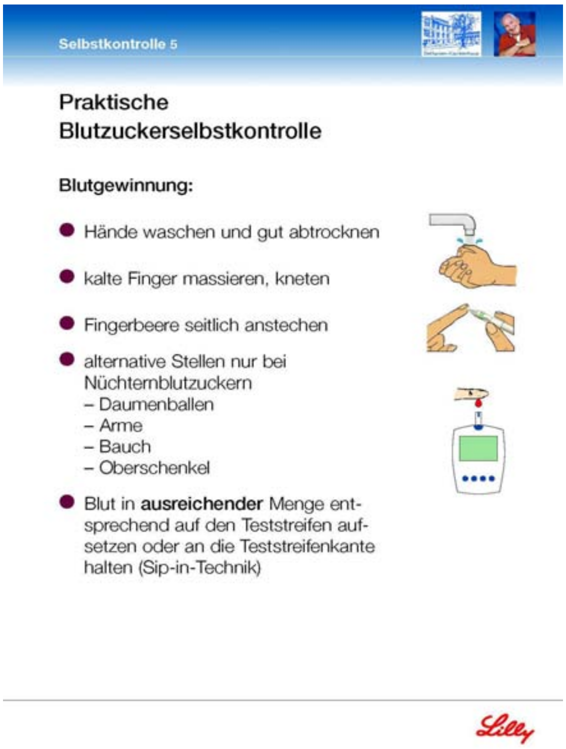

Besprechen Sie die korrekte Handhabung der Teststreifen:

Folie: Hinweis zur Teststreifenhandhabung

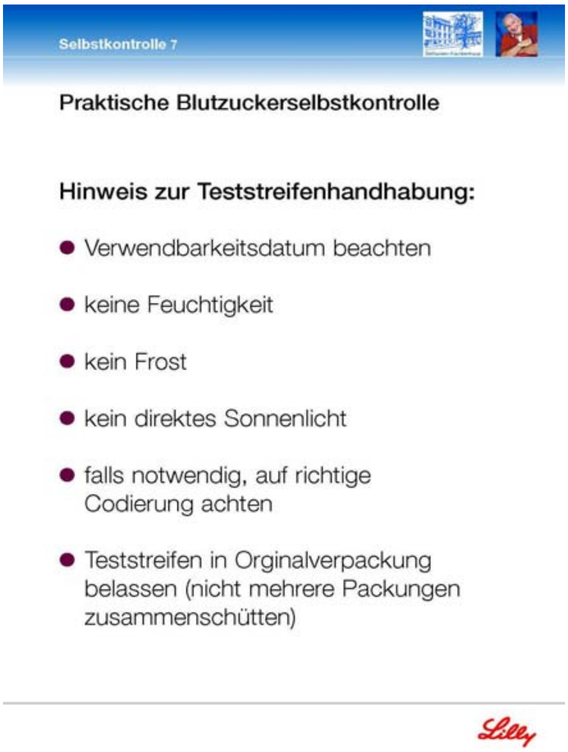

Besprechen Sie das Führen des Blutzuckertagebuchs:

Folie: Blutzuckertagebuch

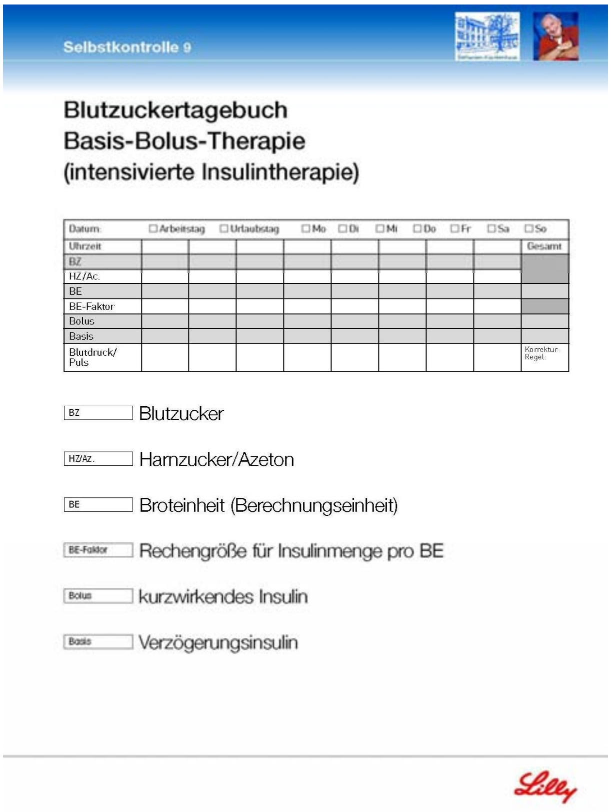

1.3.2 Blutdruck

Erhöhte Blutdruckwerte bereiten ähnlich wie chronisch erhöhte Blutzuckerwerte in der Regel wenig Beschwerden. Viele Menschen mit erhöhten Blutdruckwerten nehmen sie wegen ihrer relativen Beschwerdefreiheit nicht richtig ernst.

Ziele

- Die Teilnehmer sollen erkennen, dass das Erkennen und die Behandlung erhöhter Blutdruckwerte bei Menschen mit Diabetes genauso wichtig ist, wie dies Therapie erhöhter Blutzuckerwerte.
- Die Teilnehmer sollen ein Verständnis über mögliche Ursachen und die Folgen eines unbehandelten Bluthochdrucks entwickeln.
- Die Teilnehmer sollen motiviert werden regelmäßig ihren Blutdruck zu kontrollieren und zu dokumentieren.
- Die Teilnehmer sollen Lernen ihren Blutdruck selbst zu messen und die gemessenen Werte selbst beurteilen.

Vorgehen:

<u>Blutdruckerleben:</u>

Leiten Sie in das Thema ein und erfragen Sie, wie die Teilnehmer „über ihren Blutdruck denken“. Fragen Sie die Teilnehmer danach, inwieweit sie den Bluthochdruck bisher als persönliches Problem erlebt haben. Lassen Sie die Teilnehmer von ihren bisherigen Behandlungsbemühungen im Alltag berichten.

Folie: Blutdruck: ein „leichtes“ oder ernsthaftes Problem?

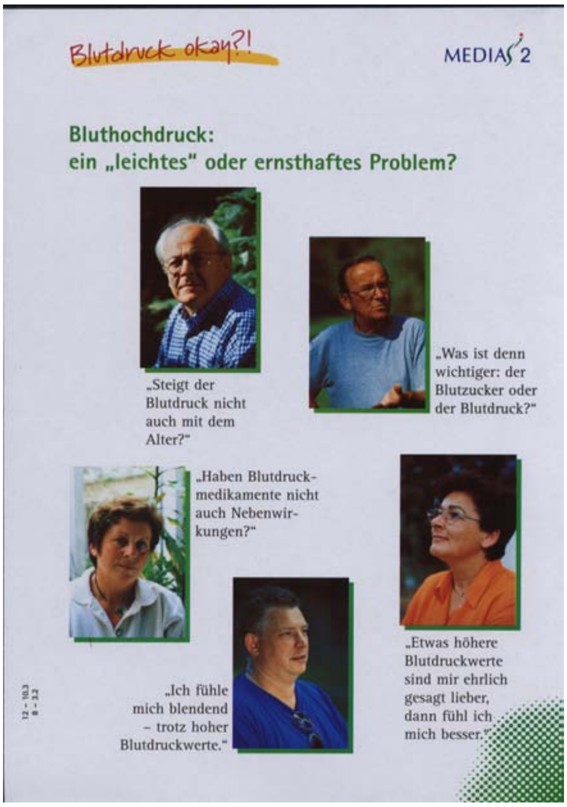

Plausibles Erklärungsmodell

Erläutern Sie zunächst in einfachen Worten den Begriff „Blutdruck“.

Erklären Sie die Bedeutung der beiden Werte, die bei der Messung des Blutdrucks angegeben werden.

Folie: Was versteht man darunter?

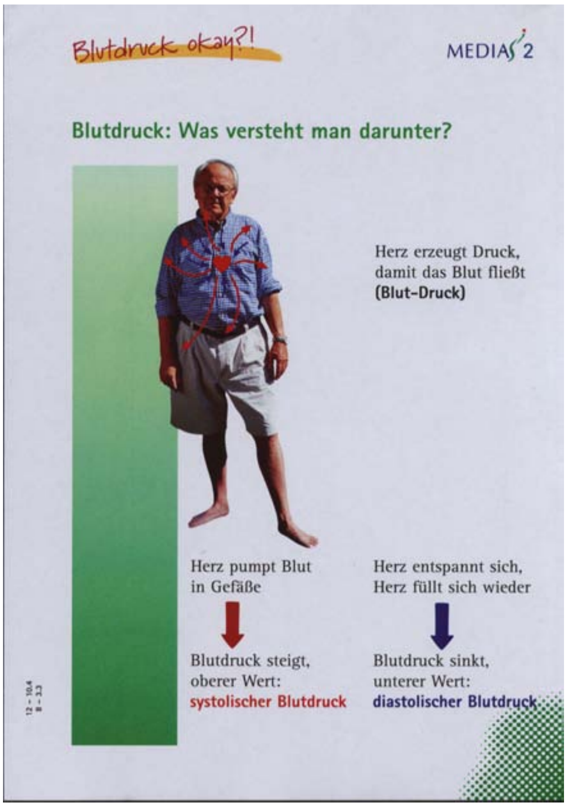

Informieren Sie die Teilnehmer darüber, welche Blutdruckwerte als „normal“ oder günstig einzuschätzen sind.

Folie: Was sind günstige Blutdruckwerte?

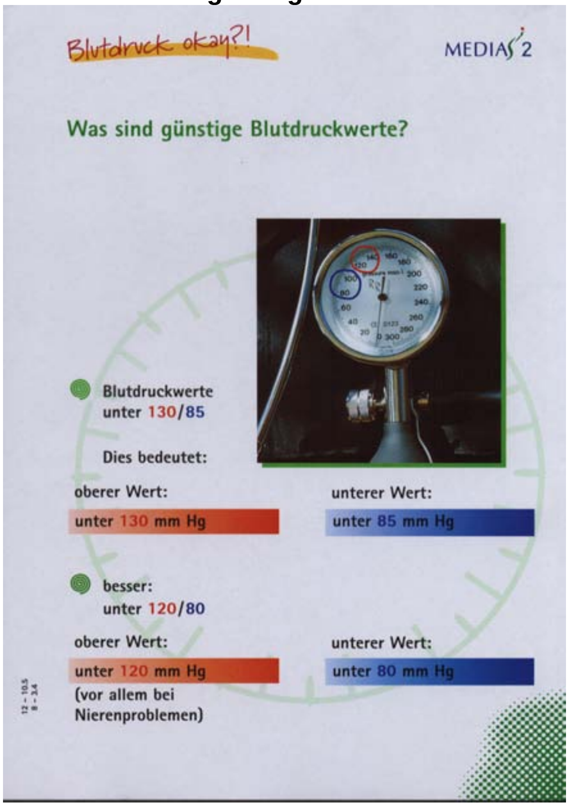

Blutdruckmessung:

Regen Sie die Teilnehmer an, regelmäßig den Blutdruck zu kontrollieren.

Informieren Sie die Teilnehmer anschließend über Möglichkeiten der Blutdruckmessung.

Folie: Den Blutdruck selbst messen: Welche Geräte gibt es?

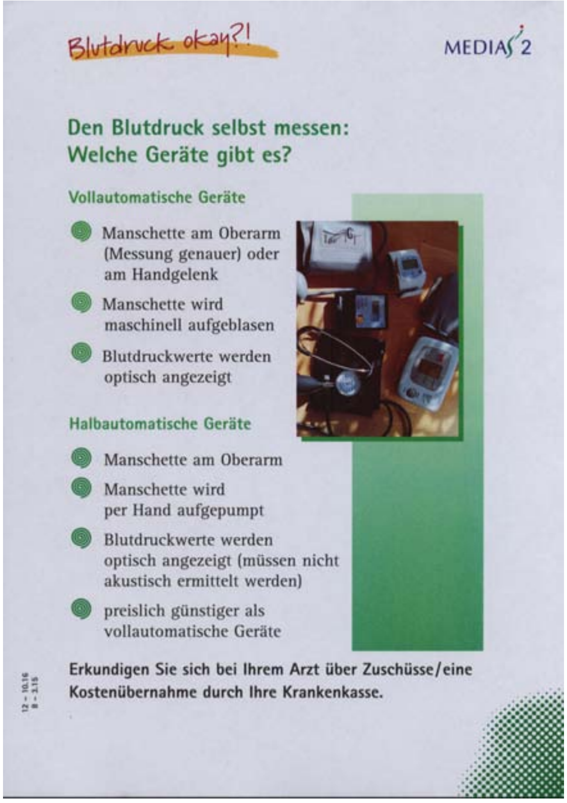

Folie: Blutdruckmessung: die klassische Methode

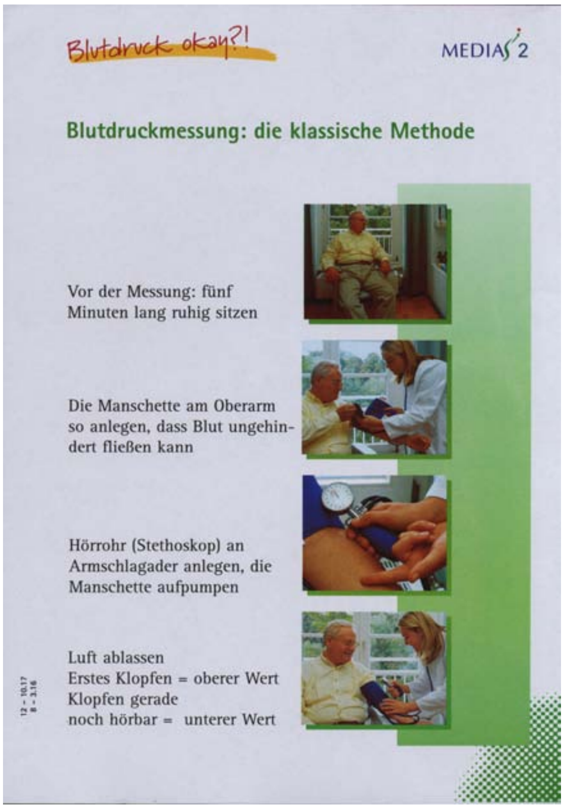

Kursstunde 2: Überblick

2.	Ernährung	Seite
**2.1**	**Gesunde Ernährung**	**18**
2.1.1	Lebensmittel Pyramide	19
2.1.2	Gesunde Mischkost	19
**2.2.**	**Kohlehydrate**	**20**
**2.3**	**BE**	**20**
**2.4**	**Glykämischer Index**	**21**

Kursstunde 2: Materialien

**2. Ernährung**	**Folien**
**2.1 Gesunde Ernährung**	„Gesunde Ernährung auch für Diabetiker“
	„Die Ernährungspyramide“<br>„Gesunde Mischkost“
**2.2 Kohlehydrate**	“Kohlehydrate“
**2.3 BE**	„Schätzeinheiten“
**2.4 Glykämischer Index**	„Glykämischer Index“
	Blutzucker erhöhend?“<br>„Nahrungsmittel mit langsamer Blutzuckerwirkung“<br>„Nahrungsmittel oder gemischte Mahlzeiten mit mittlerer Blutzuckerwirkung“<br>„Nahrungsmittel mit schneller Blutzuckerwirkung“<br>„Nahrungsmittel mit sehr schneller Blutzuckerwirkung“

2. Ernährung

2.1 Gesunde Ernährung

Ziele:

- Der Teilnehmer weiß, dass es keinen Unterschied zwischen der Ernährung von Diabetikern und der empfohlenen gesunden Ernährung für Nichtdiabetiker gibt.
- Der Teilnehmer weiß, welche Nährstoffe in den Nahrungsmitteln enthalten sind.
- Der Teilnehmer weiß, wieviel Energie die einzelnen Nährstoffe liefern.

Vorgehen:

Erläutern Sie den Teilnehmern, dass es keinen Unterschied zwischen einer gesunden Ernährung von Diabetikern und Nichtdiabetikern gibt.

Folie: Gesunde Ernährung auch für Diabetiker

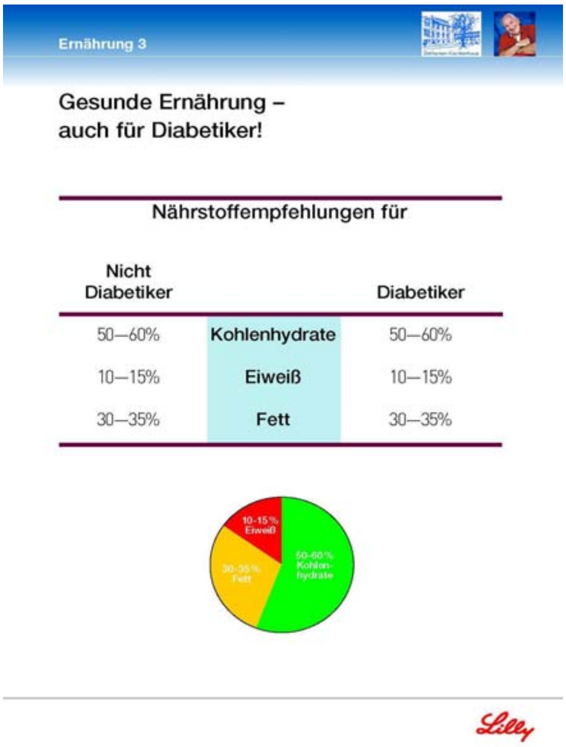

Erläutern Sie die Ernährungspyramide.

Folie: Die Ernährungspyramide

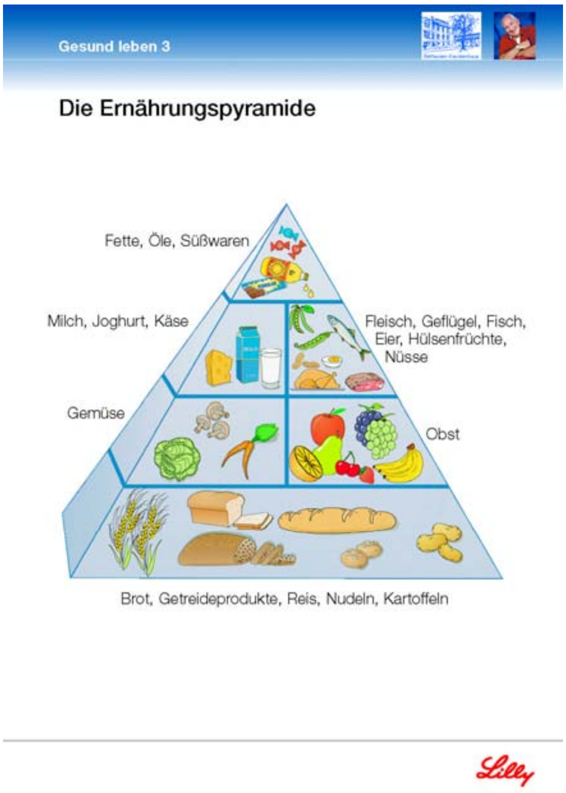

Erläutern Sie den Teilnehmern wie sich eine gesunde Mischkost zusammensetzt.

Folie: Gesunde Mischkost

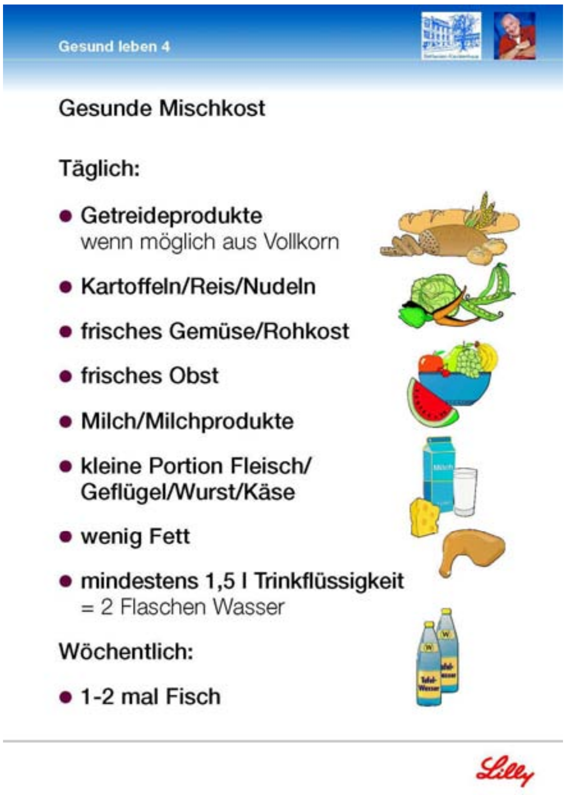

2.2 Kohlehydrate

Ziele:

- Der Patient weiß, dass kohlehydrathaltige Nahrungsmittel den Blutzucker erhöhen.

Vorgehen:

Erläutern Sie den Teilnehmern welche Kohlehydrate es gibt und worin diese enthalten sind.

Folie: Kohlehydrate

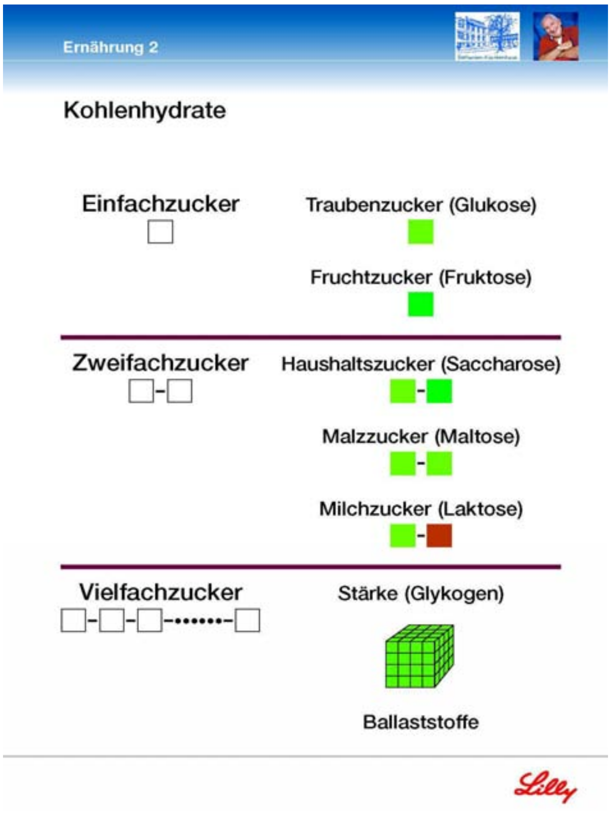

2.3 BE

Ziele:

- Die Teilnehmer wissen, was eine BE ist.
- Die Teilnehmer können Nahrungsmittel aufzählen, die den Blutzucker erhöhen bzw. nicht erhöhen.
- Die Teilnehmer können kohlehydrathaltige Nahrungsmittel mengenmäßig einschätzen (BE).
- Die Teilnehmer kennen Nahrungsmittel mit schneller bzw. langsamer Blutzuckerwirkung.

Vorgehen:

Wiederholen Sie mit den Teilnehmern die unterschiedlichen Maße, die es gibt um kohlehydrathaltige Nahrungsmittel einzuschätzen.

Folie: Schätzeinheiten

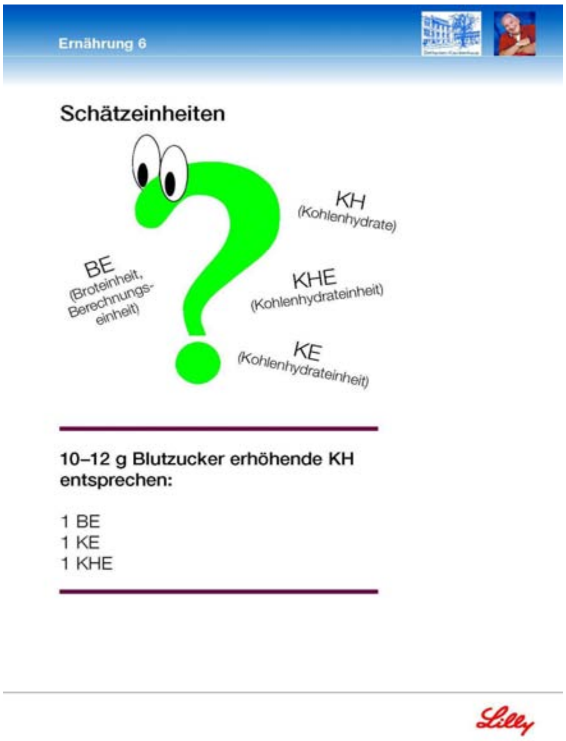

2.4 Glykämischer Index

Ziele:

- die Teilnehmer sollen den Glykämischen Index der verschiedenen Nahrungsmittel kennen.

Vorgehen:

Erläutern Sie den Teilnehmern den Glykämischen Index der Nahrungsmittel und deren Wirkungsweisen auf den Blutzucker.

Folie: Anstieg des Blutzuckerspiegels

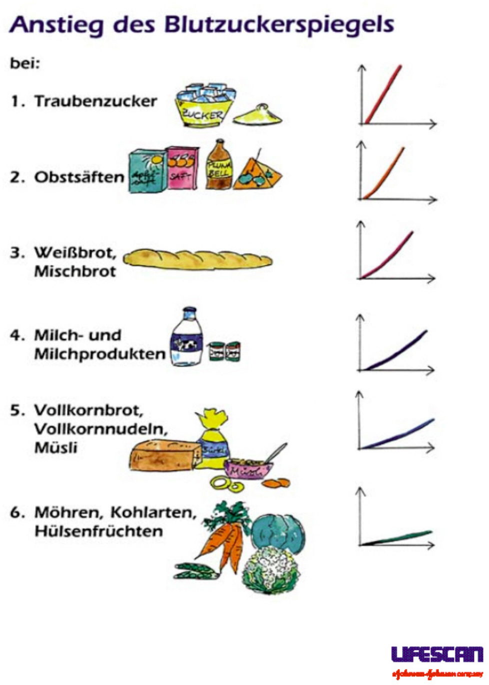

Besprechen Sie mit den Teilnehmern, welche Nahrungsmittel Blutzucker erhöhend wirken.

Folie: Blutzucker erhöhend?

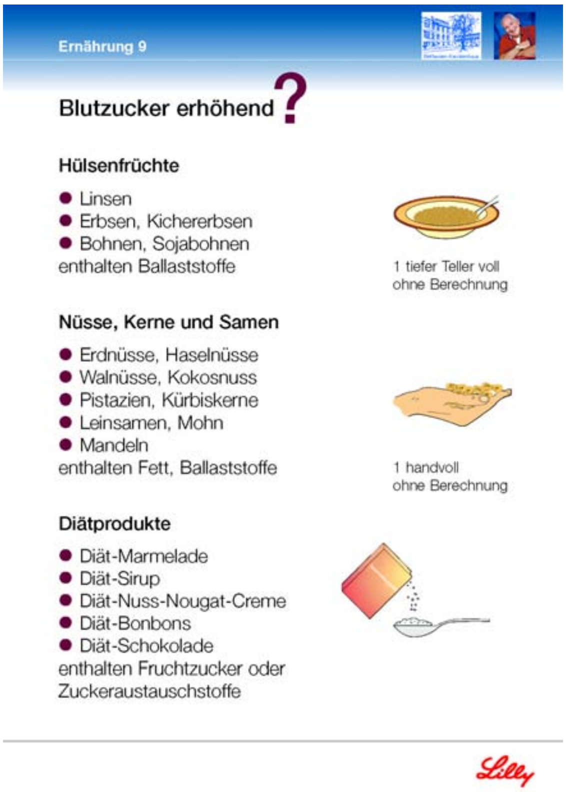

Besprechen Sie mit den Teilnehmern, welche Nahrungsmittel einen langsamen Blutzuckeranstieg bewirken.

Folie: Nahrungsmittel mit langsamer Blutzuckerwirkung

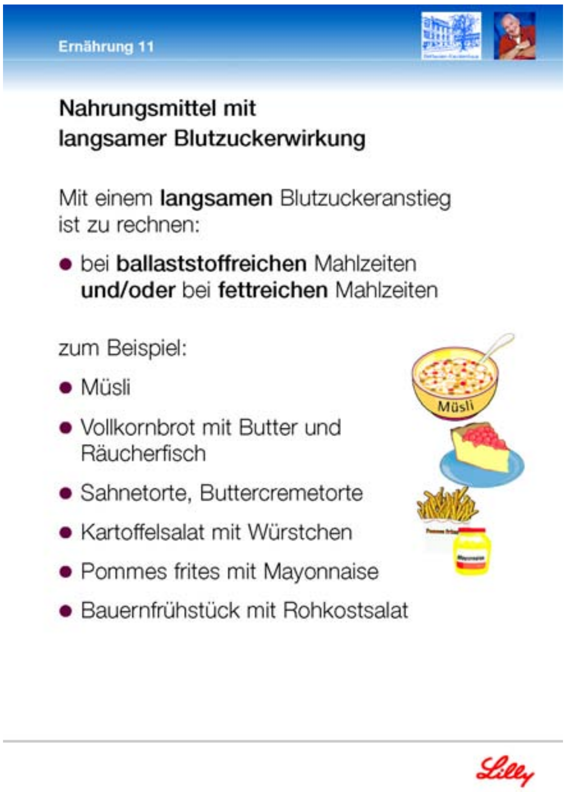

Besprechen Sie mit den Teilnehmern, welche Nahrungsmittel einen mittleren Blutzuckeranstieg bewirken.

Folie: Nahrungsmittel oder gemischte Mahlzeiten mit mittlerer Blutzuckerwirkung

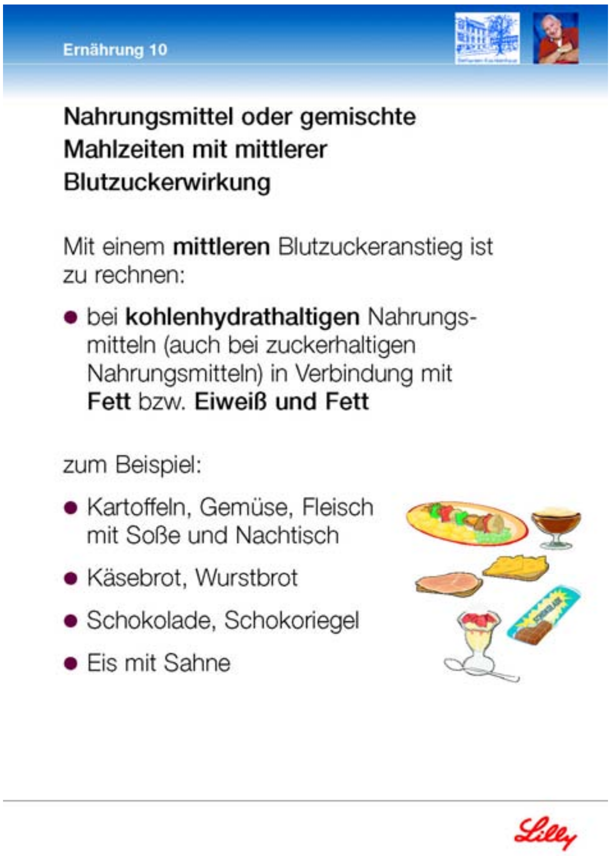

Besprechen Sie mit den Teilnehmern, welche Nahrungsmittel einen schnellen Blutzuckeranstieg bewirken.

Folie: Nahrungsmittel mit sehr schneller Blutzuckerwirkung

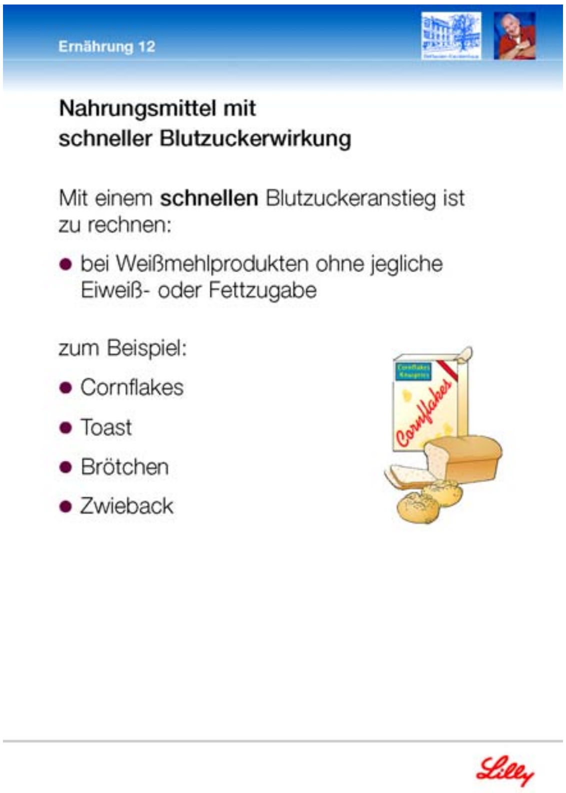

Besprechen Sie mit den Teilnehmern, welche Nahrungsmittel einen sehr schnellen Blutzuckeranstieg bewirken.

Folie: Nahrungsmittel mit sehr schneller Blutzuckerwirkung

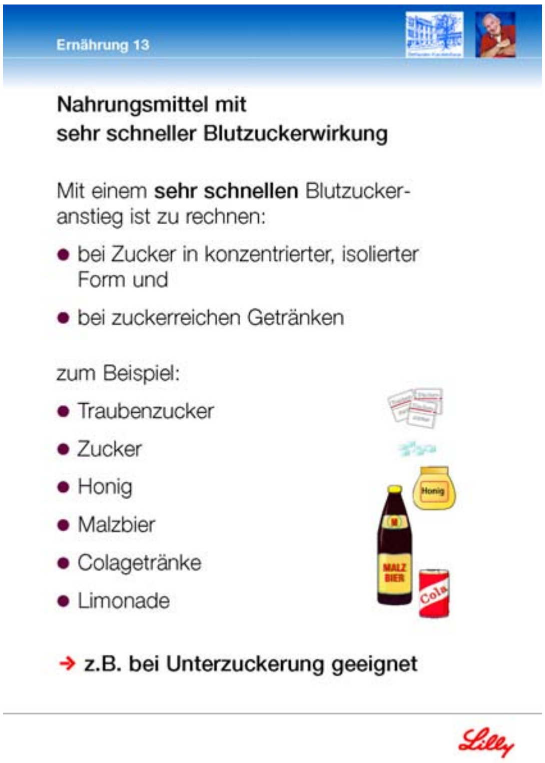

Kursstunde 3: Überblick

**3.**	**Grundlagen der Insulintherapie**	**Seite**
3.1.	Insulinwirkung	27
3.2	Dosisanpassung	30
3.3	Spritztechnik	33

Kursstunde 3: Materialien

	Folien
**3. Grundlagen der Insulintherapie**	
3.1 Insulinwirkung	„Insulinkurven: Kurzwirkendes Analoginsulin“
	„Insulinkurven: Verzögerungsinsulin“
	„Insulinkurven: Mischinsulin“
	„Analoginsulin vs. Normalinsulin“
	„Insulinkurven und KH-Aufnahme“
3.2 Insulinanpassung	„Basis – Bolus Prinzip“
	„Korrektur des Blutzuckers“
	„BZ-Therapieziel“
	„Spritz-Ess-Abstand bei Normalinsulin“
	„Spritz-Ess-Abstand bei Kurzwirkendem Analoginsulin“
3.3 Spritztechnik	„Spritztechnik“
	„Spritztechnik“
	„Ungeeignete Körperregionen“

Bitte den Teilnehmern Arbeitsblatt 13 für Kursstunde 5 mit nach Hause geben!

3. Grundlagen Insulintherapie

3.1 Insulinkurven

Ziele:

- der Teilnehmer kennt Wirkungseintritt, stärkste Wirkung und Wirkdauer der verschiedenen Insuline.
- der Teilnehmer weiß, dass die Kohlehydratmenge und das Insulin aufeinander abgestimmt werden müssen, um eine gute Blutzuckereinstellung zu haben.
- der Teilnehmer weiß, wodurch sich der Insulinbedarf verändern kann.

Vorgehen:

Besprechen Sie mit den Teilnehmern die Wirkung (Wirkungseintritt, stärkste Wirkung und Wirkdauer) der verschiedenen Insuline.

Folie: Insulinkurven

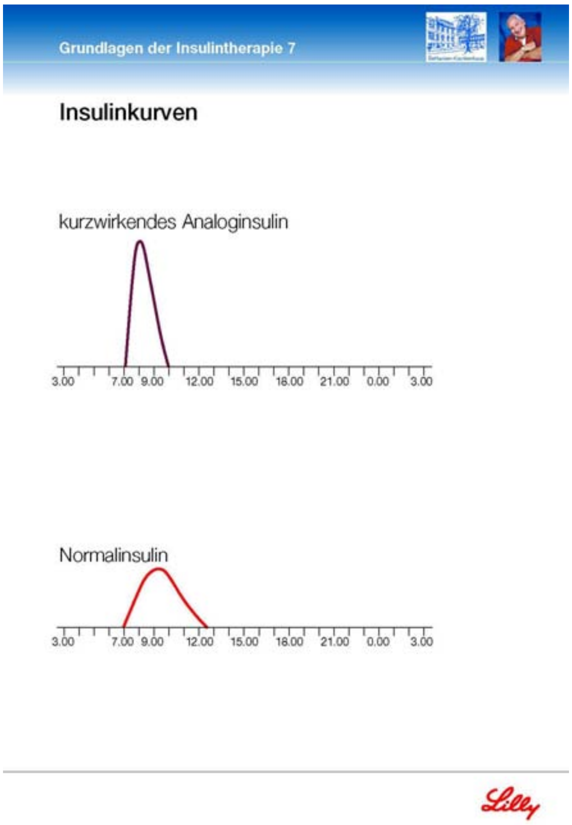

Folie: Insulinkurven

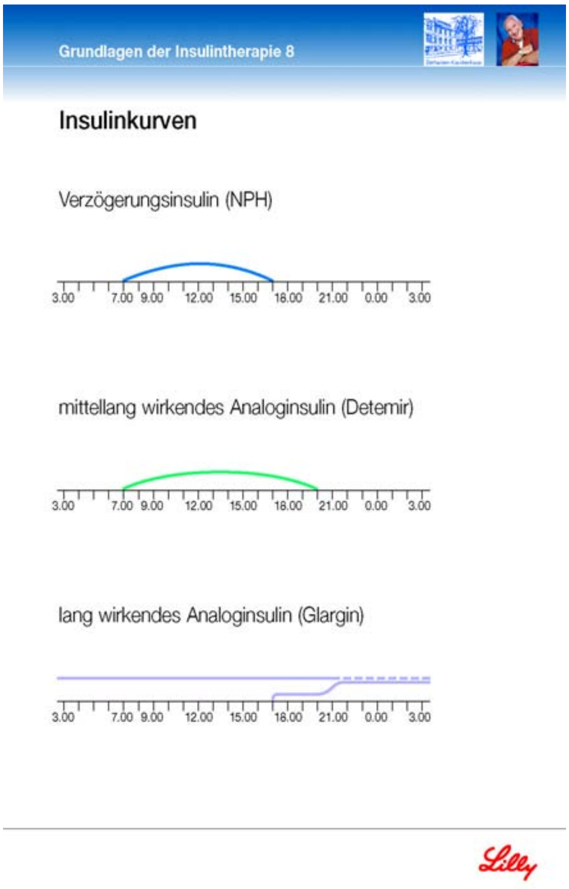

Folie: Insulinkurven

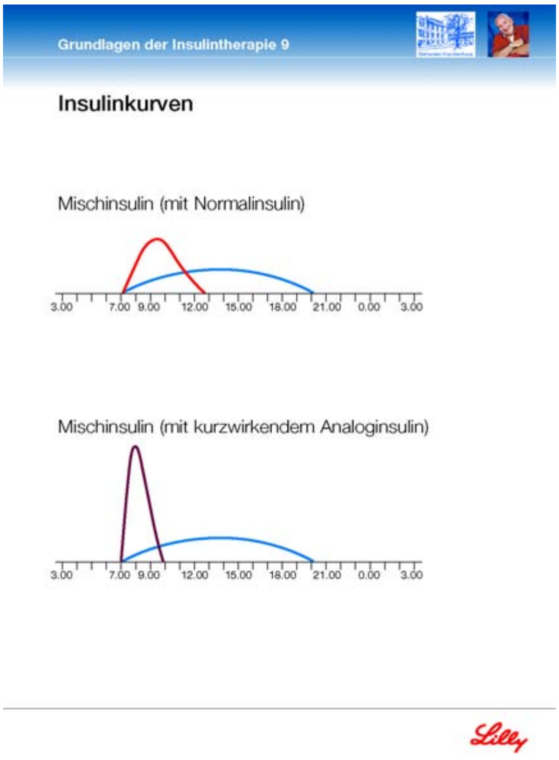

Folie: Kurzwirkendes Analoginsulin

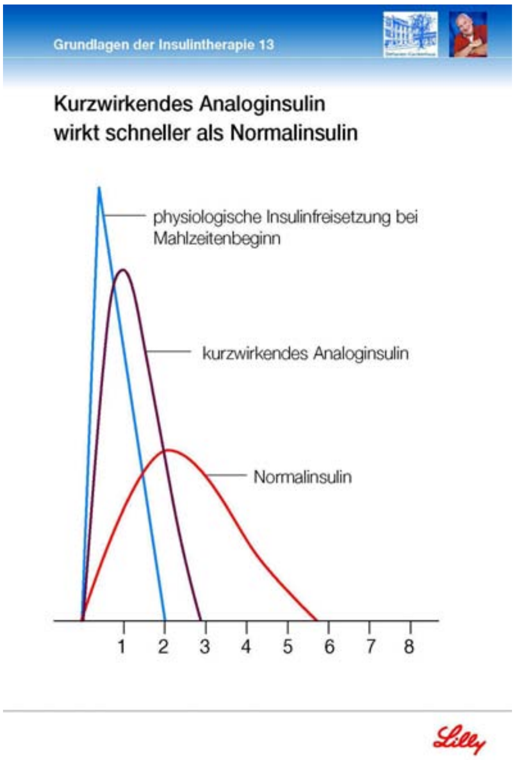

Besprechen Sie mit den Teilnehmern wie das Insulin auf die Kohlehydratmenge abgestimmt werden muss.

Folie: Insulinkurven und KH-Aufnahme

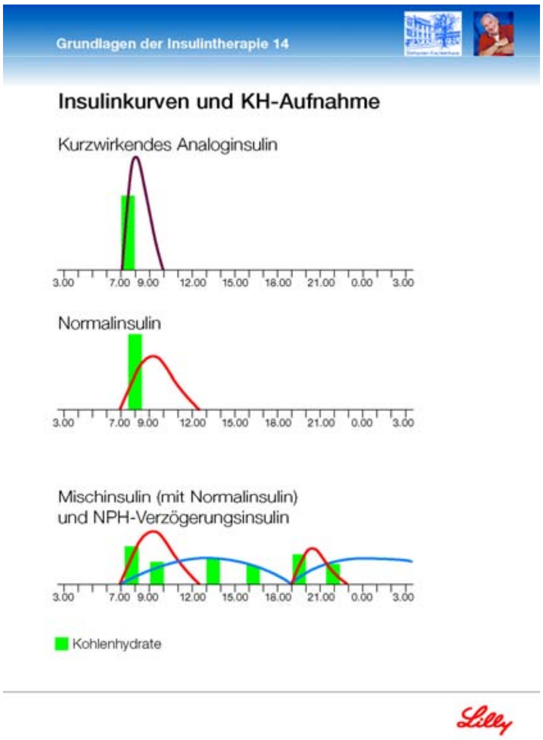

3.2 Dosisanpassung

Ziele:

- die Teilnehmer wissen, dass das Essens-Insulin und das Insulin für die Blutzucker-Korrektur als Bolus bezeichnet werden.
- die Teilnehmer wissen, dass der Bolus mit einem kurz wirkenden Insulin gespritzt wird
- die Teilnehmer wissen, dass sie bei jedem kurz wirkenden Insulin für Zwischenmahlzeiten extra spritzen können.
- die Teilnehmer wissen, dass sie mit Normalinsulin frühestens 4 Stunden nach dem letzten Bolus ihren Blutzucker korrigieren dürfen.
- die Teilnehmer wissen, dass das Blutzucker-Therapieziel vor den Hauptmahlzeiten individuell verschieden sein kann.
- die Teilnehmer kennen ihren eigenen Zielwert für den Blutzucker.
- die Teilnehmer wissen, dass der Blutzucker vor dem Schlafengehen nicht unter 120 mg/dl liegen sollte
- die Teilnehmer wissen, wann es sinnvoll ist, einen Spritz-Eß-Abstand einzuhalten und wann ein Spritz-Eß-Abstand kontraindiziert ist.
- die Teilnehmer wissen, wie sie ihre BE-Faktoren überprüfen und anpassen können.
- die Teilnehmer wissen, wie sie ihre Korrektur-Regeln überprüfen und verändern können.

Vorgehen:

Besprechen Sie mit den Teilnehmern das Prinzip der Basis Bolus Therapie.

Folie: Basis-Bolus Therapie

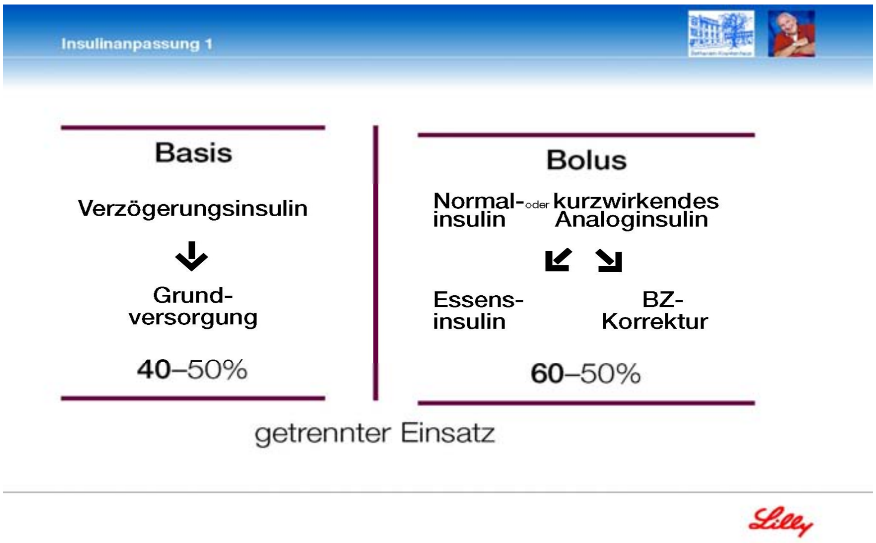

Erläutern Sie welche Korrekturmöglichkeiten die verschiedenen Insuline besitzen.

Folie: Korrektur des Blutzuckers

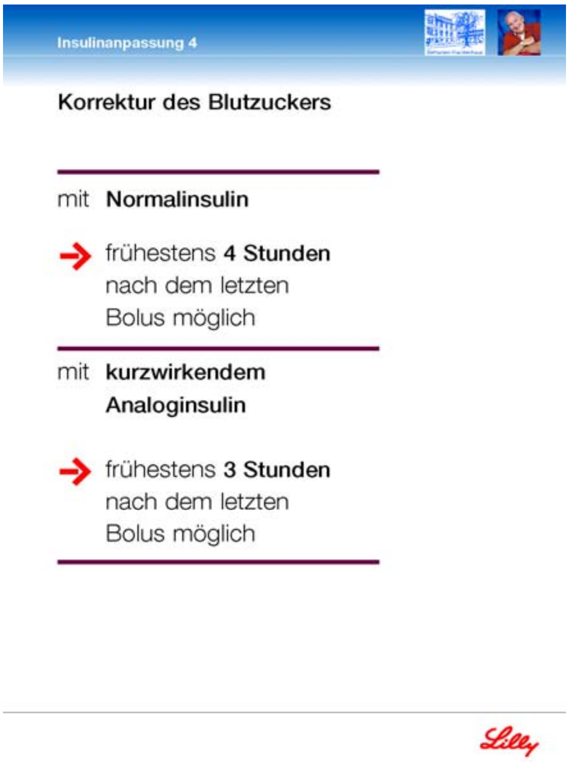

Erläutern Sie den Teilnehmern, dass das Blutzucker-Therapieziel vor den Mahlzeiten individuell verschieden sein kann.

Folie: BZ-Therapieziel

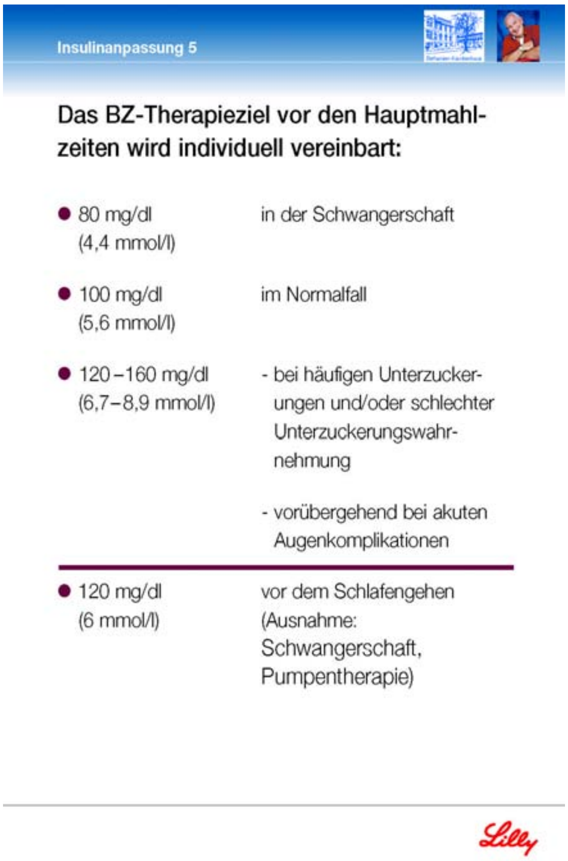

Erläutern Sie den Teilnehmern wichtige Prinzipien zur Einhaltung des Spritz-Ess-Abstandes in Abhängigkeit des Insulintyps

Folie: Spritz-Ess-Abstand bei Normalinsulin

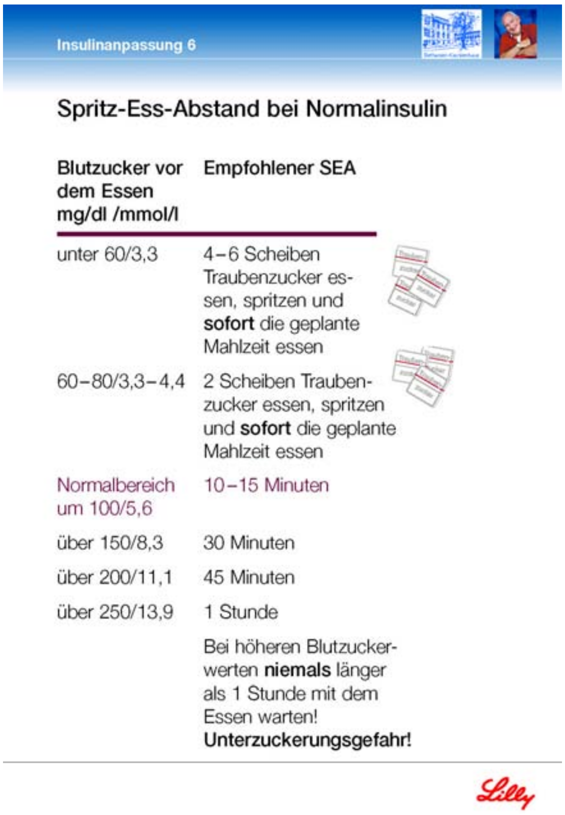

Folie: Spritz-Essabstand bei Kurzwirkendem Analoginsulin

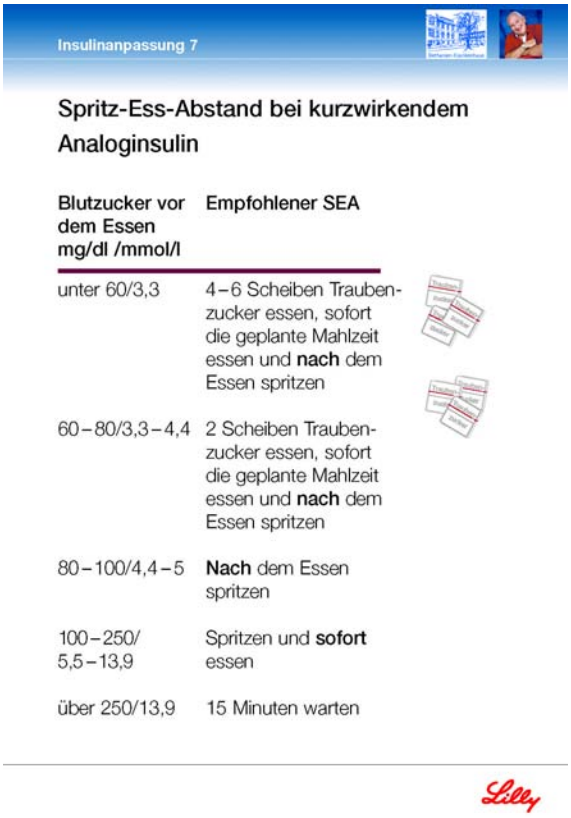

Erläutern Sie an einem Beispiel die Dosisanpassung.

Folie: Insulinanpassung

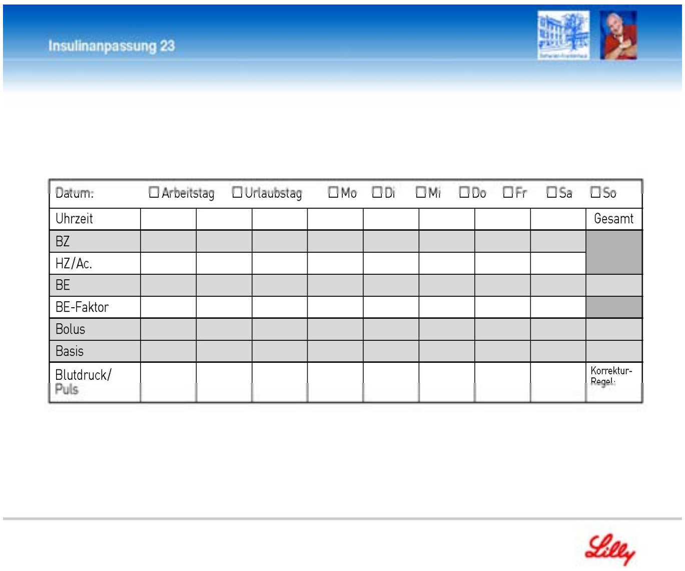

Datum:	☐ Arbeitstag	☐ Urlaubstag	☐ Mo	☐ Di	☐ Mi	☐ Do	☐ Fr	☐ Sa	☐ So
Uhrzeit									Gesamt
BZ									
HZ/Ac.									
BE									
BE-Faktor									
Bolus									
Basis									
Blutdruck/ Puls									Korrektur-Regel:

3.3 Spritztechnik

Ziele:

- Die Teilnehmer wissen, wie sie Veränderungen an den Spritzstellen vorbeugen können.
- Die Teilnehmer wissen, in welche Körperregionen sie injizieren können.

Vorgehen:

Wiederholen Sie mit den Teilnehmern die korrekte Spritztechnik und Fehler, die hierbei gemacht werden können.

Folie: Insulin wird in das Unterhautfettgewebe gespritzt

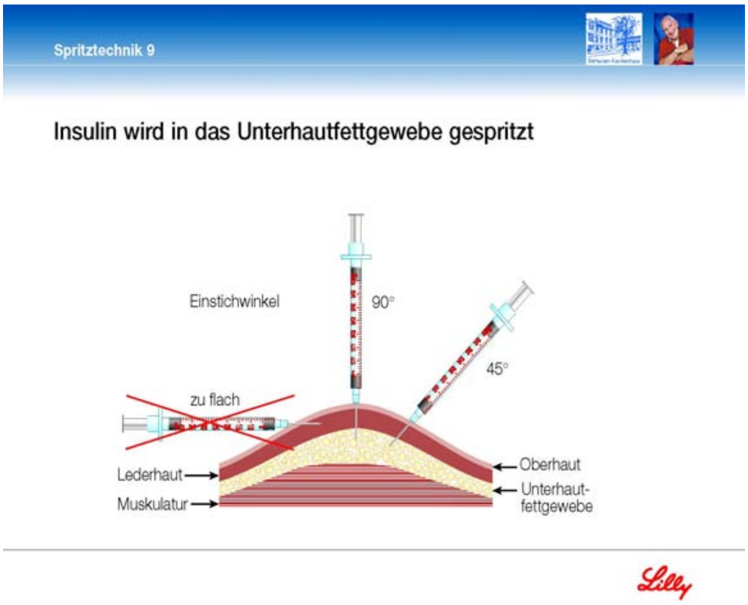

Besprechen Sie mit den Teilnehmern gut geeignete und ungeeignete Körperregionen, in die sie injizieren können.

Folie: Gut geeignete Körperregionen

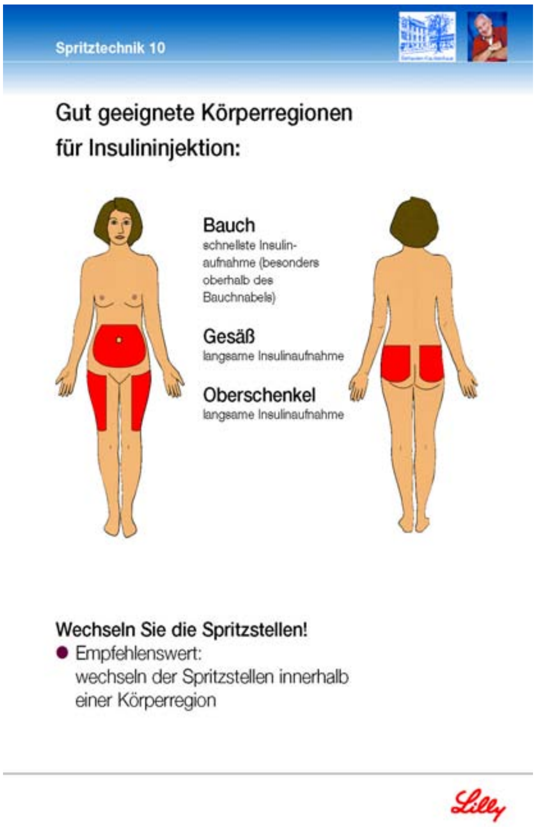

Folie: Ungeeignete Körperregionen

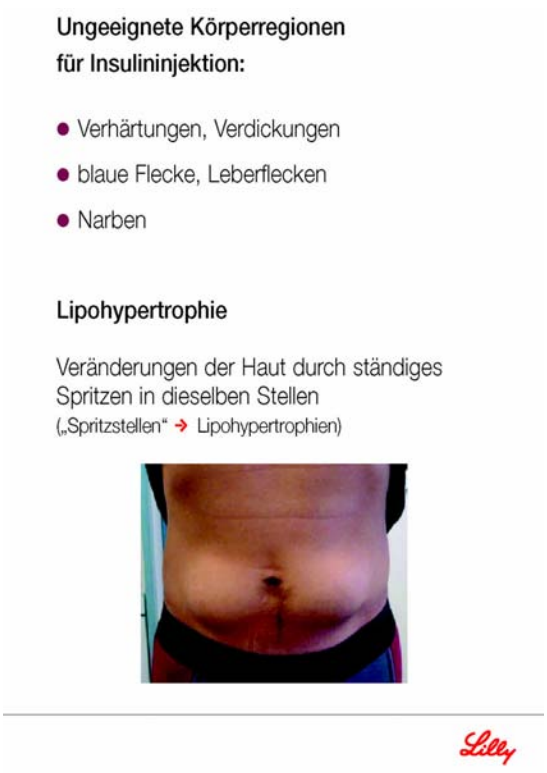

Kursstunde 4: Überblick

4.	Akutkomplikationen: Unterzuckerung	Seite
4.1.	Erkennen und Wahrnehmen	37
4.2.	Persönliche Problematiken	40

Kursstunde 4: Materialien

**4. Akutkomplikationen:**	**Folien:**
4.1 Unterzuckerung	Medias: Anzeichen von Unterzuckerung
	Lilly: Unterzuckerung 1
	Medias: Ursachen von Unterzuckerung
	Medias: Behandlung von Unterzuckerung
	Medias: Vermeidung von Unterzuckerung
	Lilly: Schwere Unterzuckerung
4.2. Persönliche Problematiken	-------

4. Akutkomplikationen:

4.1 Unterzuckerung

Ziele:

- die Teilnehmer erhalten genauere Informationen über Erscheinungsbild und Folgen akuter Blutzuckerentgleisungen.
- die Teilnehmer erwerben Kenntnisse über Behandlungsmaßnahmen bei auftretenden Unterzuckerungen.
- durch die Erarbeitung/Wiederholung von Kenntnissen zu Stoffwechselentgleisungen sollen die Teilnehmer zusätzlich motiviert werden, regelmäßig Blutzuckerselbstkontrollen durchzuführen.

Vorgehen:

Erarbeiten Sie gemeinsam mit den Teilnehmern mögliche Anzeichen einer Unterzuckerung.

Befragen Sie die Teilnehmer nach bisherigen Erfahrungen mit Unterzuckerungen und sammeln Sie Informationen zum erlebten Erscheinungsbild.

Fassen Sie die gesammelten Symptome anschließend zusammen:

Folie: Anzeichen von Unterzuckerungen

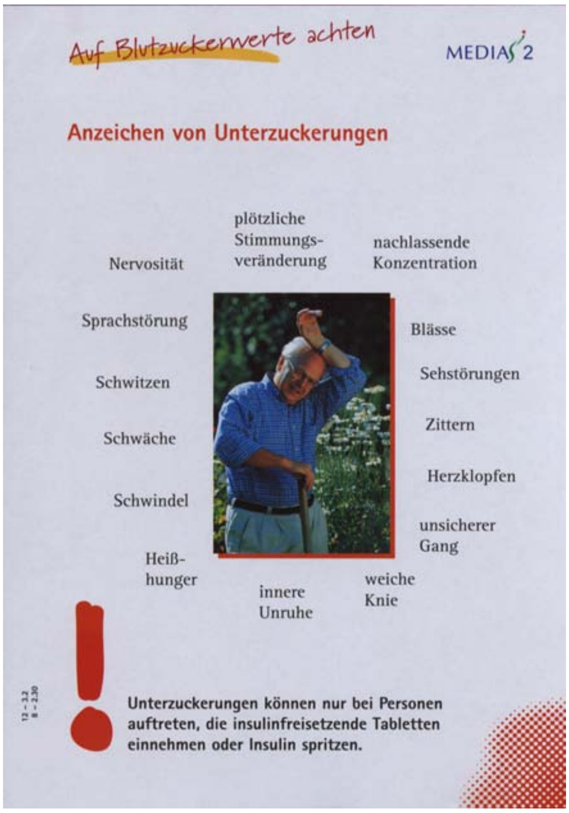

Wiederholen Sie nochmals den Wertebereich von Unterzuckerungen.

Folie: Hypoglykämie

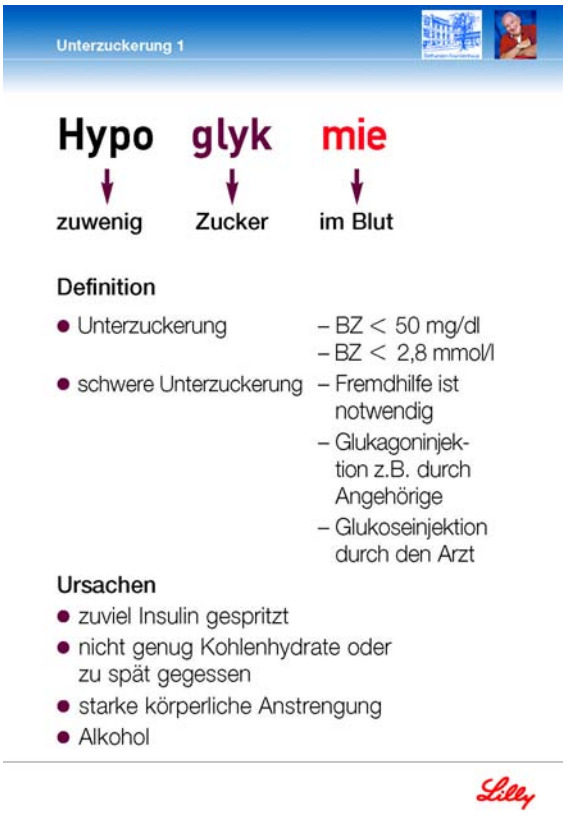

Besprechen Sie zusammen mit den Teilnehmern mögliche Ursachen einer Unterzuckerung.

Folie: Ursachen von Unterzuckerung

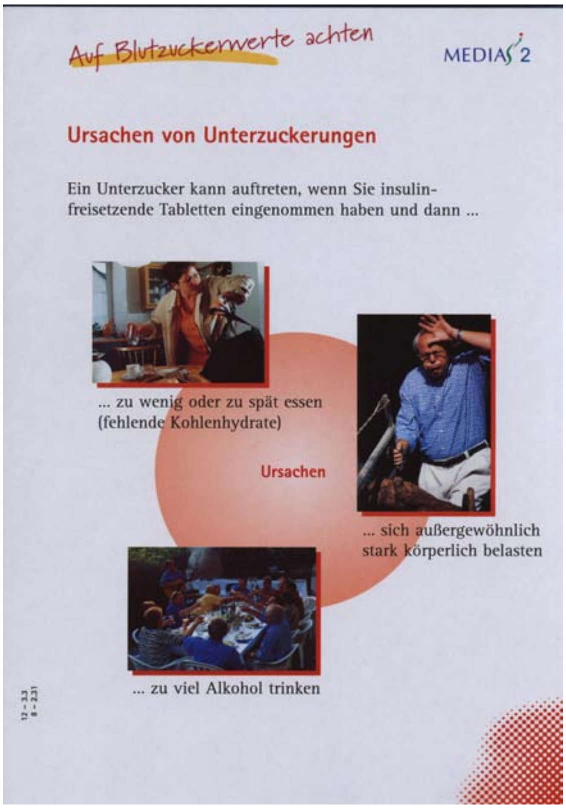

Informieren Sie anschließend die Teilnehmer über geeignete Behandlungsmaßnahmen bei Unterzuckerungen.

Folie: Behandlung von Unterzuckerung

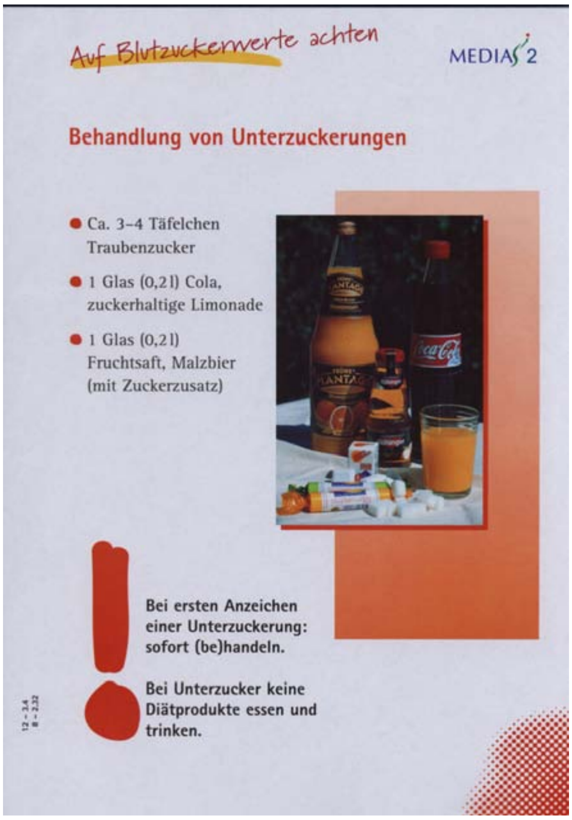

Besprechen Sie anschließend mit den Teilnehmern geeignete Möglichkeiten der Vermeidung von Unterzuckerungen.

Folie: Tipps zur Vermeidung von Unterzuckerung

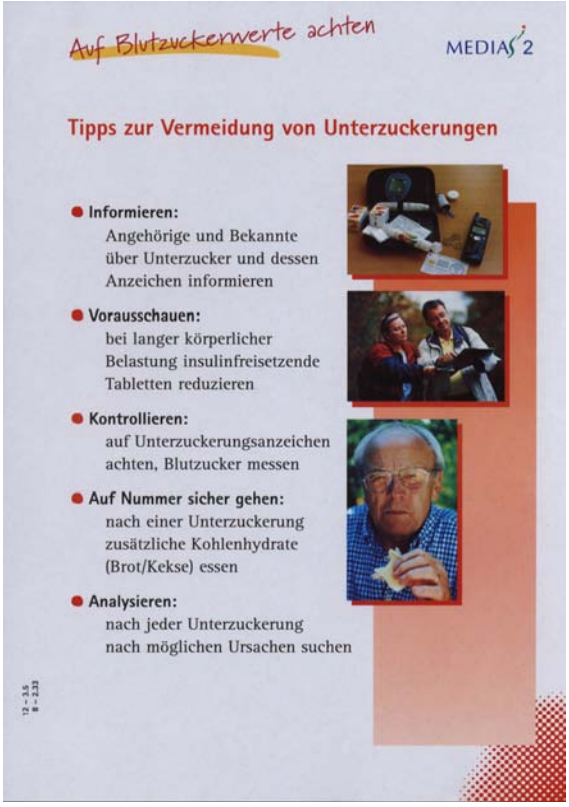

Besprechen Sie mit den Teilnehmern die Anwendung der Glukagon Injektion

Folie: Schwere Unterzuckerung

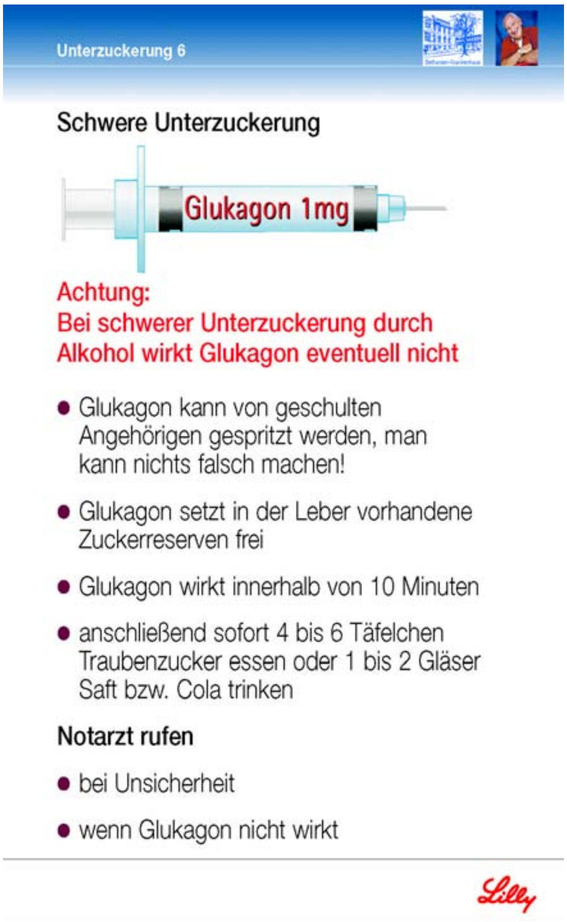

4.2. Persönliche Problematiken

Geben Sie den Patienten die Gelegenheit über persönliche Problematiken zu sprechen.

Wenn noch Zeit ist kann an dieser Stelle, auch Hyperglykämie besprochen werden.

Kursstunde 5: Überblick

**5.**	**Körperliche Aktivität**	**Seite**
5.1	Motivation zu körperlicher Aktivität	43
5.2	Dosisanpassung an körperliche Aktivität	47

Kursstunde 5: Materialien

5. Körperliche Aktivität	Folien:
**5.1 Motivation zu körperlicher Aktivität**	„Wie aktiv sind Sie in Ihrem Alltag?“
	„Vorteile von Bewegung bei Diabetes“
	„Sich wohler fühlen“
	„Tipps zur körperlichen Bewegung“
	„Günstig Ausdauersport“
	„Körperliche Bewegung im Alltag“
	„Anzeichen von Überanstrengung“
	„Daran sollten Sie auch denken“
**5.2 Dosisanpassung bei körperlicher Aktivität**	„Körperliche Aktivität und Energiebedarf“
	„Insulinausschüttung bei körperlicher“ Aktivität
	„Konsequenzen für insulinbehandelte Diabetiker“
	„Allgemeine Hinweise“
	„Zusatz-BE“
	„Zusatz-BE oder Insulinreduktion“
	„Anpassung bei Langzeitaktivität“
	„Anpassung bei Langzeitaktivität“
	Handgeschriebene Folie: eigenes Beispiel

5. Körperliche Bewegung

5.1. Motivation zu körperlicher Bewegung

Ziele

- Die Teilnehmer sollen in dieser Einheit verstehen, dass körperliche Bewegung eine wirksame Maßnahme innerhalb der Diabetestherapie darstellt.
- Die Teilnehmer sollen Möglichkeiten finden sich zu körperlicher Bewegung zu motivieren.
- Die Teilnehmer sollen geeignete Sportarten für sich entdecken.
- Die Teilnehmer sollen Möglichkeiten entdecken, im Alltag körperliche Aktivität einfließen zu lassen.

Vorgehen

Lassen Sie die Teilnehmer berichten, wie sie körperliche Aktivitäten in ihrem Alltag erleben. Gehen Sie hierzu auf das Arbeitsblatt ein, das sie am ersten Kurstag ausgegeben haben.

Folie: Wie aktiv sind Sie in Ihrem Alltag?

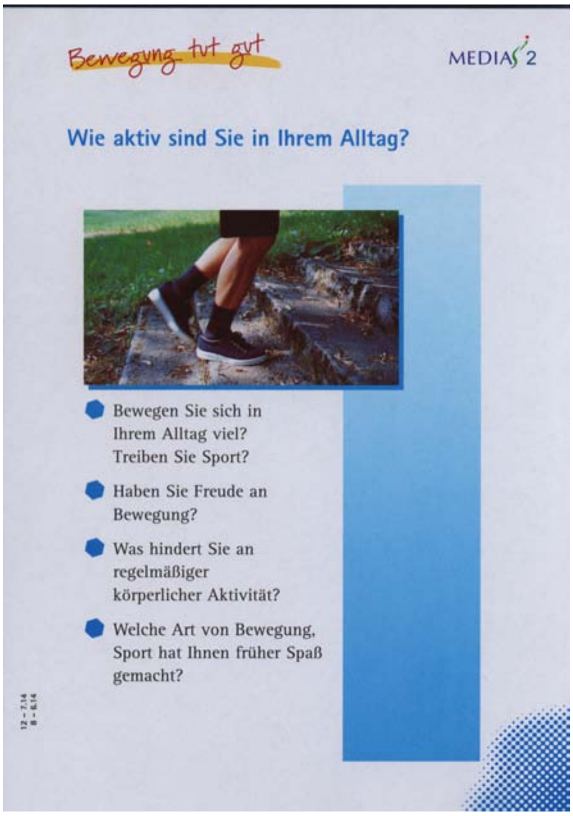

Besprechen von Arbeitsblatt 13

Erläutern Sie den Teilnehmern die Effekte, die Bewegung auf den Diabetes hat.

Folie: Vorteile von Bewegung bei Diabetes

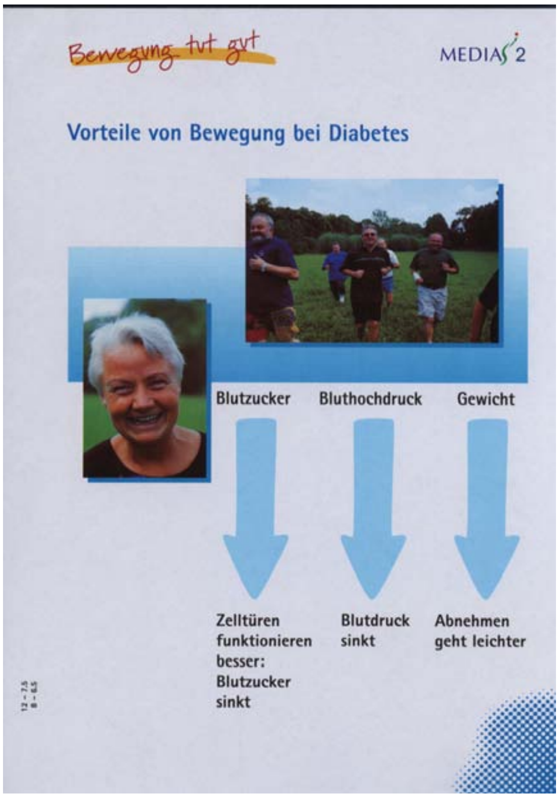

Folie: Sich wohler fühlen

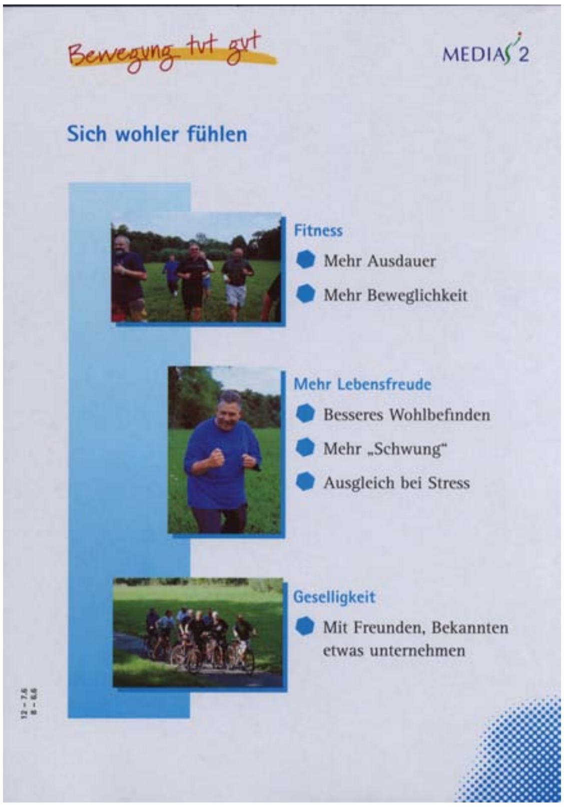

Erarbeiten Sie mit den Teilnehmern gemeinsam Möglichkeiten wie sie sich zur körperlichen Bewegung motivieren können.

Folie: Tipps zur körperlichen Bewegung

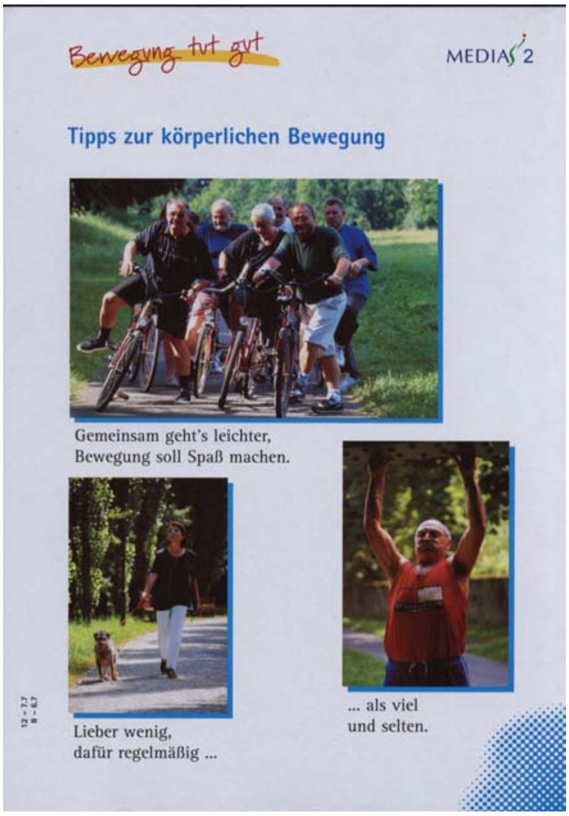

Vermitteln Sie welche Sportarten günstig sind.

Folie: Günstige Ausdauersportarten

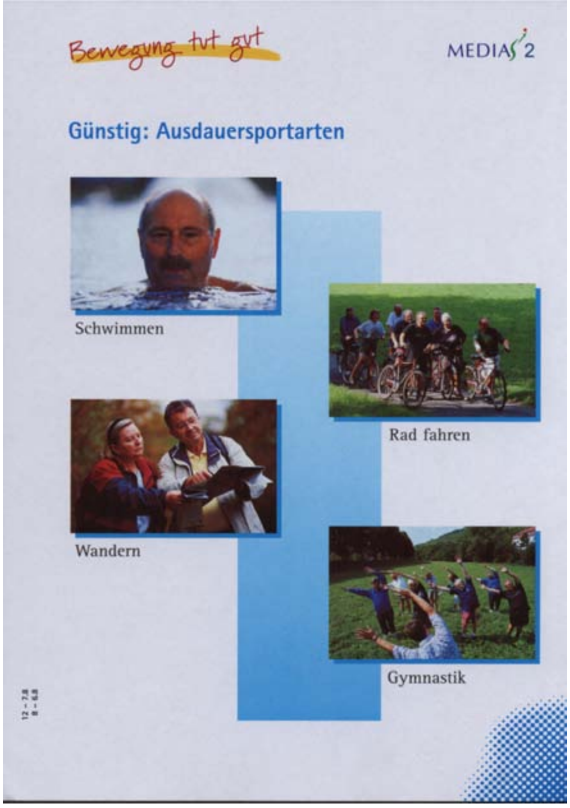

Überlegen Sie gemeinsam mit den Teilnehmern, wo sie im Alltag körperliche Bewegung einfließen lassen können.

Folie: Körperliche Bewegung im Alltag

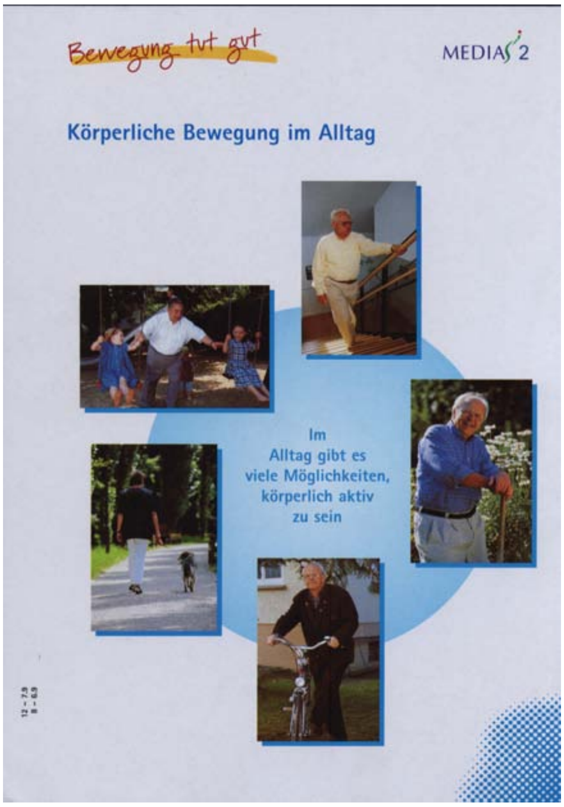

Erläutern Sie den Teilnehmern, die Anzeichen einer Überanstrengung.

Folie: Anzeichen von Überanstrengung

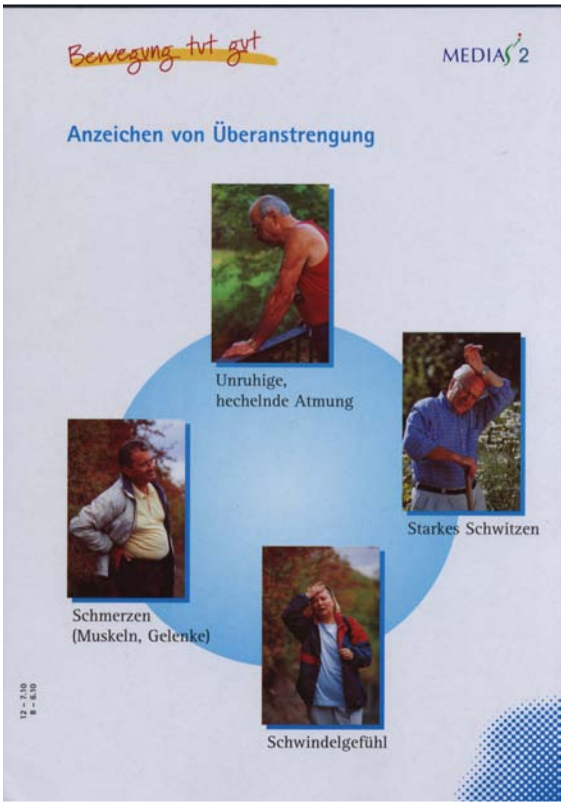

Besprechen Sie weitere wichtige Punkte bei körperlicher Bewegung.

Folie: Daran sollten Sie auch denken

5.2. Dosisanpassung bei körperlicher Aktivität

Ziele:

- die Teilnehmer wissen, wie der Körper seinen höheren Kohlehydratbedarf bei Bewegung deckt.
- die Teilnehmer wissen, dass der Insulinbedarf bei körperlicher Bewegung sinkt.
- die Teilnehmer wissen, dass sie Unterzuckerungen durch Kohlehydrat-Zufuhr und/oder Insulinreduktion vorbeugen können.
- die Teilnehmer wissen, wann sie auf Sport verzichten sollten.
- die Teilnehmer kennen spät einsetzende Unterzuckerungen als Nachwirkung von Sport.
- die Teilnehmer kennen die Faustregel für Insulinreduktion bzw. KH-Zufuhr bei Kurz- und Langzeitbelastung.
- die Teilnehmer wissen, dass sie die Regeln für sich individuell anpassen müssen.
- die Teilnehmer kennen die Risiken der gewählten Sportart im Hinblick auf dessen Unterzuckerungsrisiko und der gesundheitliche Situation.

Vorgehen:

Erläutern Sie den Teilnehmern, wie der Körper seinen höheren Kohlehydratbedarf bei Bewegung abdeckt.

Folie: bei körperlicher Aktivität steigt der Energiebedarf

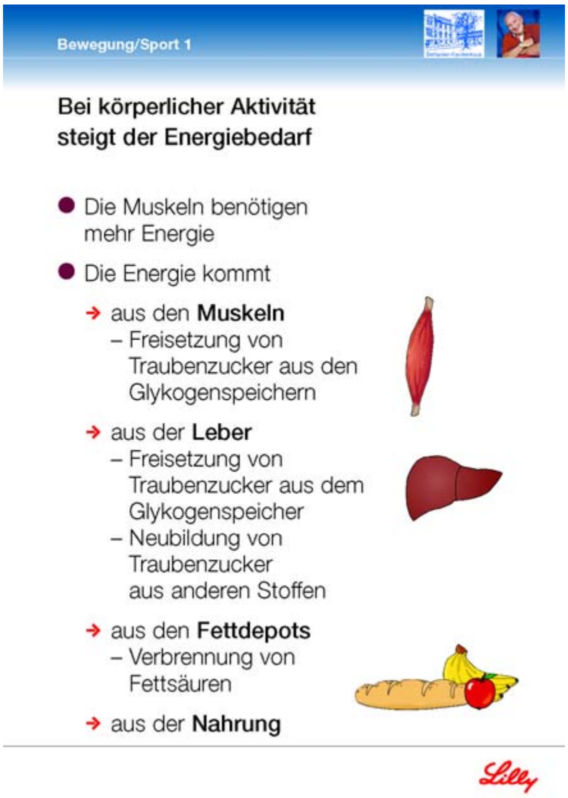

Erläutern Sie wieso der Organismus bei körperlicher Aktivität weniger Insulin benötigt.

Folie: Insulinausschüttung bei körperlicher Aktivität

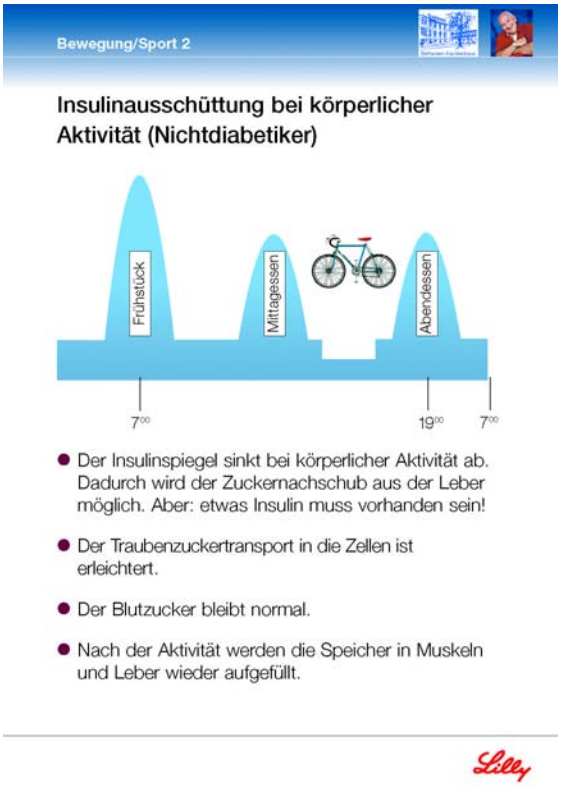

Erläutern Sie den Teilnehmern die Konsequenzen körperlicher Bewegung in Bezug auf ihren Insulinbedarf.

Folie: Konsequenzen für insulinbehandelte Diabetiker

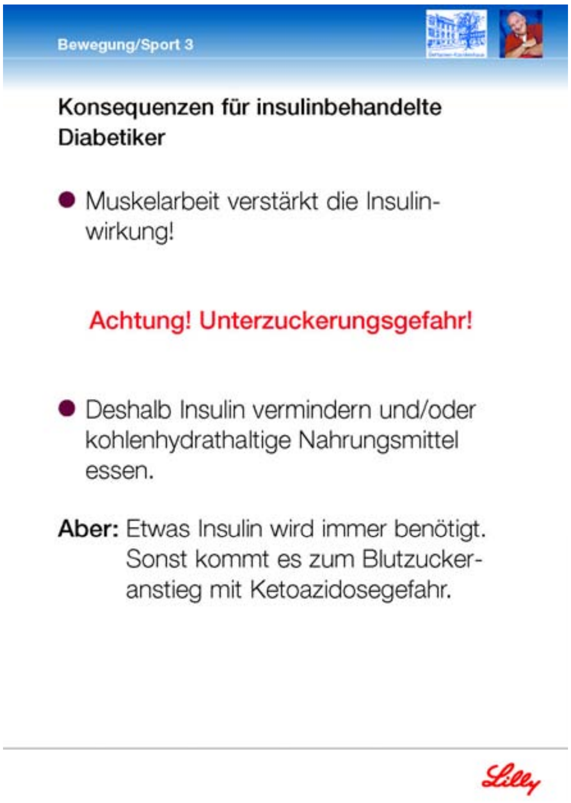

Besprechen Sie mit den Teilnehmern worauf sie bei körperlicher Aktivität achten sollten.

Folie: Allgemeine Hinweise

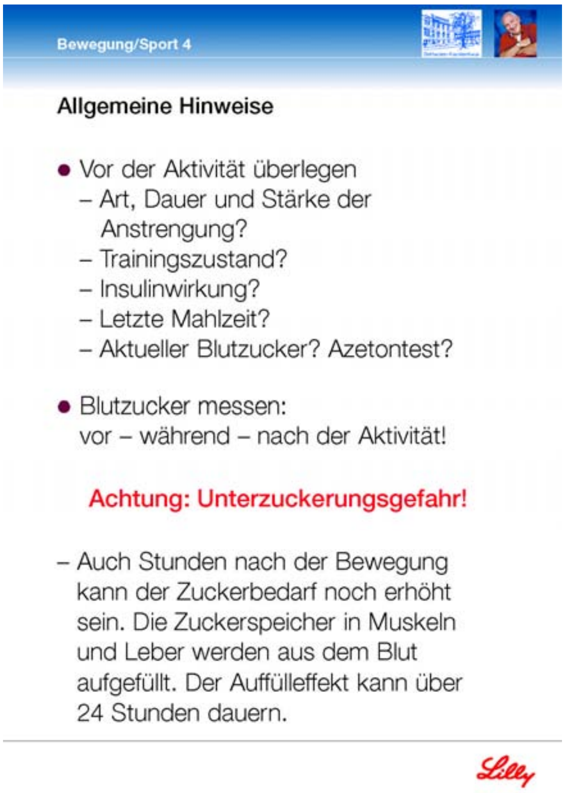

Besprechen Sie mit den Teilnehmern wie sie ihre Kohlehydratzufuhr auf die körperliche Aktivität anpassen können.

Folie: Zusatz-BE

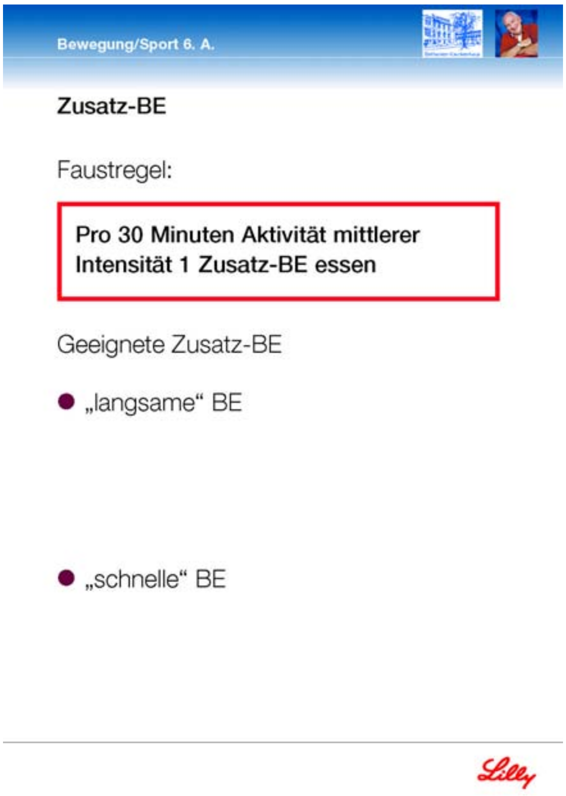

Folie: Zusatz-BE oder Insulinreduktion

Bewegung/Sport 8

Zusatz-BE oder Insulinreduktion bei Kurzzeitaktivität (1–2 Std.)

Maßnahme	Normalinsulin	Kurzwirkendes Insulinanalogon
	**Aktivitätsbeginn**	
50% Reduktion des Bolus	bis zu 3 Stunden nach dem Essensbolus	bis zu 2 Stunden nach dem Essensbolus
	vor Sport BZ testen (Zusatz BE?)	
	VI-Wirkung mitbedenken (Lücke? Maximum?)	
	**Aktivitätsbeginn**	
	später als 3 Std. nach dem Essensbolus	später als 2 Std. nach dem Essensbolus
	vor Sport BZ testen	
Zusatz-BE	VI-Wirkung mitbedenken (Lücke? Maximum?)	
	**Achtung:**	
	Normalinsulin ist evtl. noch wirksam	kurzwirkendes Insulinanalogon ist meist nicht mehr wirksam

Folie: Anpassung bei Langzeitaktivität

Bewegung/Sport 10

Anpassung bei Langzeitaktivität

- **Tagesaktivität**
  (z.B. Fahrradtour, Wandern, Renovieren, Umzug)

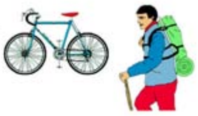

- – mehr Kohlenhydrate essen
- – Bolusinsulin zu den Mahlzeiten drastisch reduzieren (50%)
- – tagsüber und abends 30–50% weniger Verzögerungsinsulin
- – **Wichtig:** Blutzuckerkontrolle vor dem Schlafengehen. Zusatz-BE erforderlich?
- – evtl. 3.00 Uhr Blutzuckerkontrolle
- – vorsichtige Insulindosierung am nächsten Tag (Nachwirkung kann über 24 Stunden dauern! Muskelauffülleffekt)

Folie: Anpassung bei Langzeitaktivität

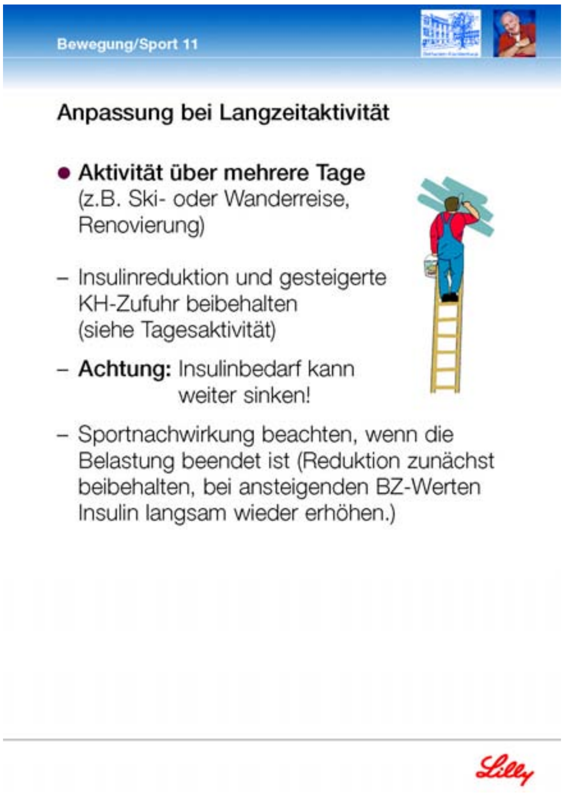

Erarbeiten Sie mit den Teilnehmern anhand eines Beispiels die Dosisanpassung. Verwenden Sie hierzu eine **Leerfolie** und lassen Sie die Teilnehmer Vorschläge machen.

Wenn noch Zeit ist oder bei Bedarf noch folgende beide Folien:

Folie: Blutzuckeranstieg nach Sport? Was tun?

bei Bedarf zusätzlich:

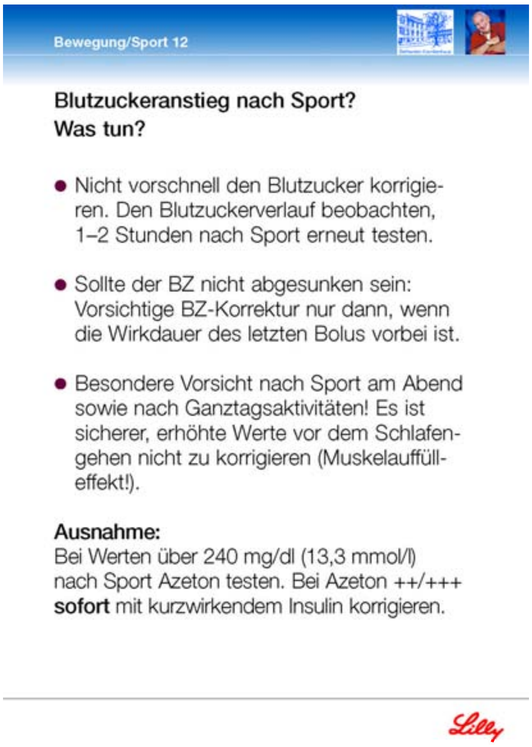

Folie: Ursachen für Blutzuckeranstieg nach Sport?

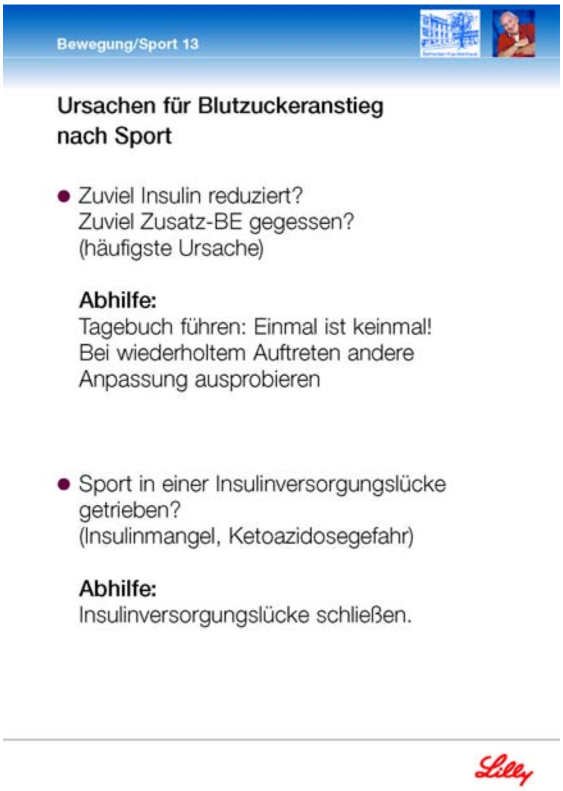

Kursstunde 6: Überblick

	Folgeerkrankungen	Seite
6.1	Risikofaktoren für Folgeerkrankungen	55
6.2	Rethinopathie	58
6.3.	Füße	59
6.4	Kontrolle	63

Kursstunde 6: Materialien

**Folgeerkrankungen**	**Folien:**
**6.1 Risikofaktoren für Folgeerkrankungen**	„Wie denken Sie darüber?“
	„Folgeerkrankungen betreffen den ganzen Körper“
	„Was gefährdet die Blutgefäße?“
	„Umgang mit Folgeerkrankungen“
**6.2 Rethinopathie**	„Augen“
**6.3 Füße**	„Unsere Füße“
	„Warum sind Füße bei Diabetes gefährdet?“
	„Anzeichen von Nervenstörungen“
	„Anzeichen von Durchblutungsstörungen“
	„Ein guter Schuh von außen betrachtet“
	„Ein guter Schuh von innen befühlt“
	„Die Füße täglich kontrollieren“
**6.4 Kontrollintervalle**	„Diabetes-Check-Up“
**6.5 Feedback**	
**Arbeitsblätter 16 und 17 mit nach Hause geben.**	

Kursstunde 6: Durchführung

6. Folgeerkrankungen des Diabetes

6.1. Risikofaktoren für Folgerkrankungen bei Diabetes

Die Teilnehmer beschäftigen sich mit wichtigen Risikofaktoren (Blutdruck, Störungen des Fettstoffwechsels, Rauchen), die für das Entstehen von Folgekomplikationen verantwortlich sind.

Ausdrücke wie „Folgeerkrankungen des Diabetes“ wie auch „Risikofaktoren“ stellen für die Betroffenen häufig eher abstrakte Begriffe dar. Ermöglichen Sie den Teilnehmern einen persönlichen Bezug: lassen Sie die Teilnehmer ihr persönliches Risikoprofil einschätzen.

Ziele

- Die Teilnehmer sollen verstehen, wie Folge- und Begleiterkrankungen des Diabetes zustande kommen.
- Die Inhalte sollen so dargestellt werden, dass die Teilnehmer einen persönlichen Bezug dazu herstellen: es geht nicht um das Risiko für Folgeerkrankungen im Allgemeinen, sondern um das individuelle Risiko eines Teilnehmers.
- Die Auseinandersetzung mit den möglichen Folgen dauerhaft erhöhter Blutzuckerwerte soll die Teilnehmer motivieren, sich möglichst gut um die Behandlung des Diabetes zu bemühen.
- Die wichtigste Botschaft dieser Einheit sollte daher lauten: „Der Diabetes kann sehr unangenehme Folgen für die Gesundheit mit sich bringen, aber diese sind durch eigenes Verhalten beeinflussbar bzw. zu verhindern.“

Vorgehen

Fragen Sie die Teilnehmer wie sie über Folgeerkrankungen denken.

Folie: Folgeerkrankungen des Diabetes: Wie denken Sie darüber?

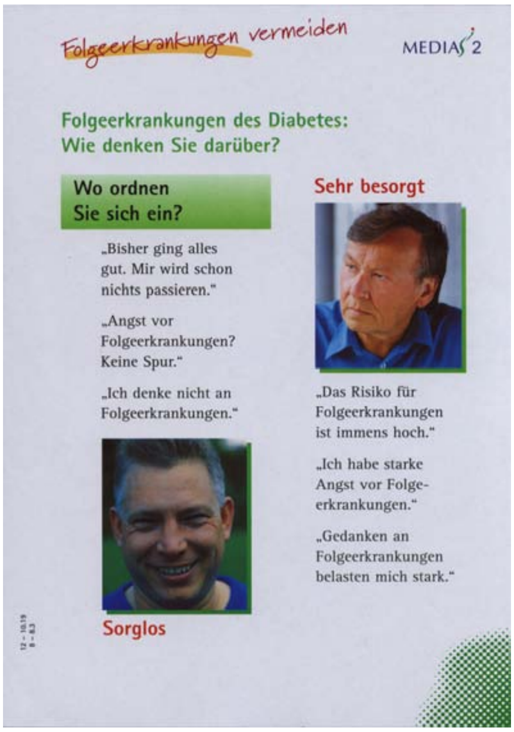

Erläutern Sie die wesentlichen Risikofaktoren für die Entstehung von Folge- und Begleiter-kankungen.

Folie: Folgeerkrankungen des Diabetes betreffen den ganzen Körper

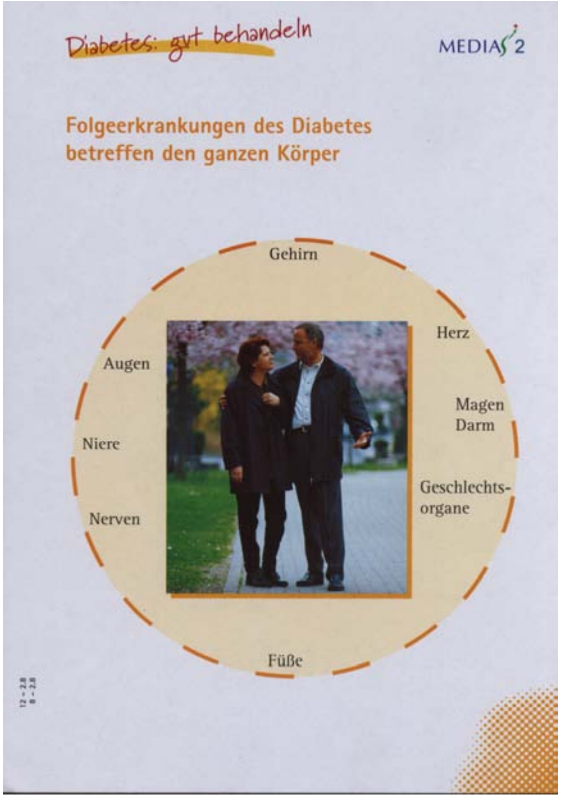

Gehen Sie nochmals auf die Konsequenzen eines Blutzuckerstaus vor den Zellen ein, wodurch sich Probleme an den Blutbahnen ergeben können. Durch Verengungen und Ablagerungen an den Blutgefäßen können verschiedene Organe in Mitleidenschaft gezogen werden. Solche Durchblutungsstörungen können sowohl an großen Blutgefäßen als auch an den kleinen Blutgefäßen auftreten.

Weisen Sie aber auch auf Schädigungen der Nerven hin, welche die Funktionsbeeinträchtigungen oder – Ausfall innervierter Organe zur Folge haben kann.

Folie: Was gefährdet Ihre Blutgefäße?

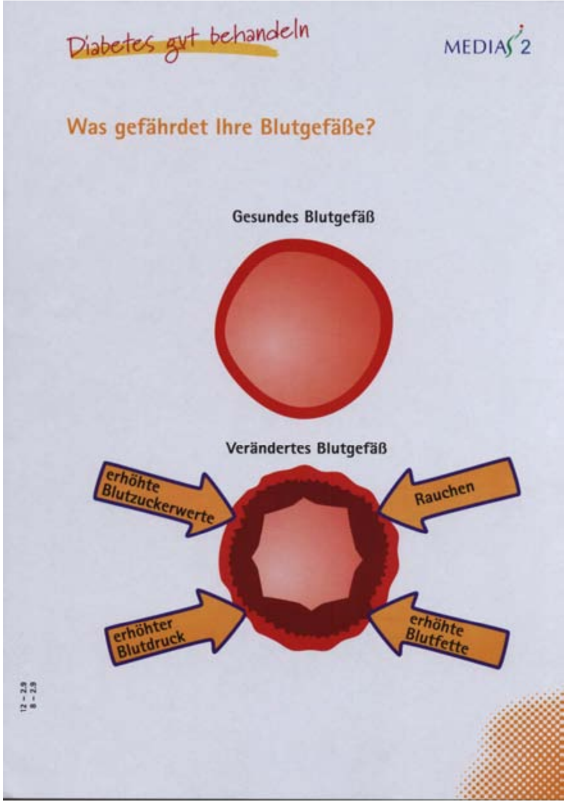

Folie: Umgang mit Folgeerkrankungen

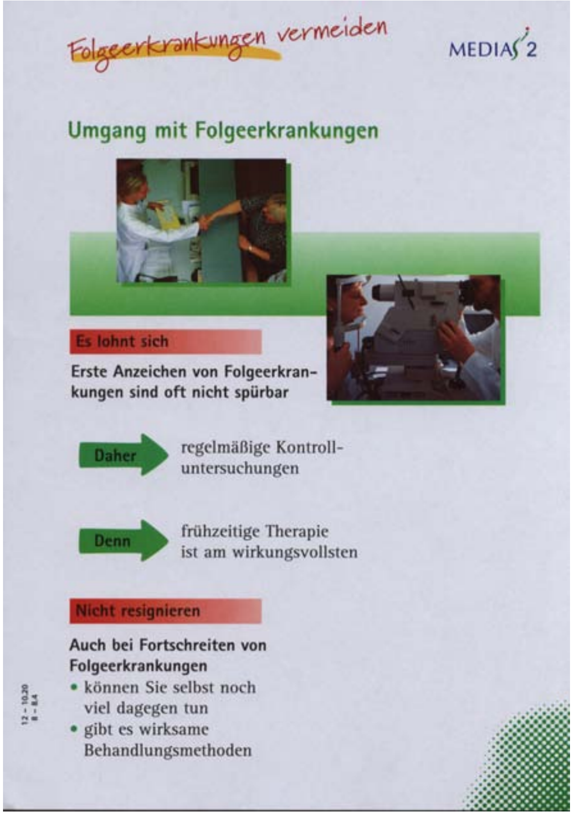

6.2 Rethinopathie

Ziel:

- Ziel ist es, den Teilnehmern ein Verständnis zur Ursache und Erkennung der Rethinopathie zu vermitteln.
- Die Teilnehmer sollen wissen welche Behandlungsmöglichkeiten es gibt.

Vorgehen:

Erläutern Sie den Teilnehmern, dass die Rhethinopathie eine häufige Folgeerkrankung des Diabetes ist, wie sie sie erkennen und behandeln können.

Folie: Folgeerkrankungen vermeiden: Augen

6.3 Füße

Ziele:

- Ziel dieser Einheit ist, dass die Teilnehmer ein Verständnis zur Entstehung und Vorbeugung von diabetischen Fußproblemen gewinnen.
- Die Teilnehmer lernen, ihre Schuhe und Strümpfe im Hinblick auf ihre Eignung zur Vermeidung von Fußproblemen anhand einfacher und verständlicher Kriterien zu beurteilen.
- Die Teilnehmer beschäftigen sich mit Auslösern von Fußkomplikationen, die zunächst kaum wahr- und ernst genommen werden, später jedoch zu schwerwiegenden Problemen führen können.
- Die Teilnehmer sollen in der Lage sein, regelmäßig und strukturiert ihre Füße zu kontrollieren

Vorgehen:

Fordern Sie die Teilnehmer auf, zu erzählen was deren Füße tagsüber leisten. Lassen Sie die Teilnehmer die Diskrepanz erkennen zwischen der Wichtigkeit der Füße und dem geringen Ausmaß an Aufmerksamkeit, die ihnen häufig entgegengebracht wird.

Folie: Warum sind die Füße bei Diabetes gefährdet?

Erarbeiten Sie mit den Teilnehmern die Gründe dafür, dass unscheinbare Druckstellen und Verletzungen am Fuß bei Menschen mit Diabetes ein großes Problem verursachen können.

Erläutern Sie die Gründe für das erhöhte Risiko:

- schlechtere Durchblutung
- Verletzung oder Druckstellen tun nicht weh
- Wunden heilen schlechter

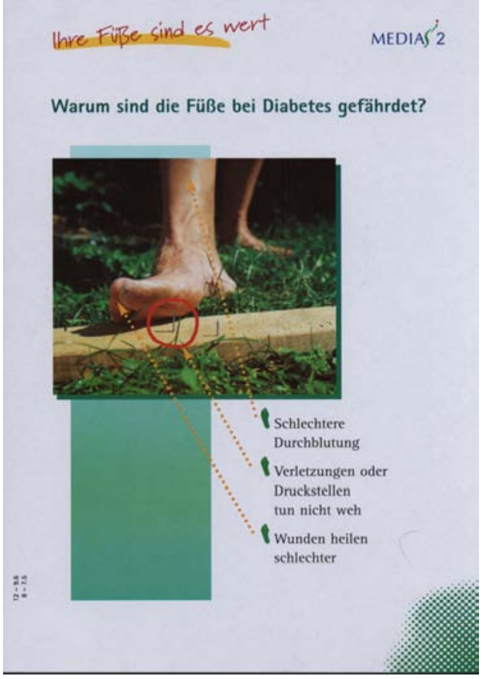

Beschreiben Sie, dass langfristig erhöhte Blutzuckerwerte Nerven und Gefäße schädigen und benennen Sie die entsprechenden Erkrankungen:

- Diabetesbedingte Nervenstörung (Neuropathie)
- Durchblutungsstörungen an den Beinen (arterielle Verschlusskrankheit)

Informieren Sie die Teilnehmer darüber:

- woran sie selbst diese Erkrankungen erkennen können
- wie der Arzt sie feststellen kann

Beschreiben Sie anhand praktischer Beispiele, dass bei einer Neuropathie Schmerzen nicht mehr wahrgenommen werden:

- eingeschränktes Temperaturempfinden
- Kribbeln, Ruheschmerz, Taubheitsgefühl
- Verluste des Schwitzens, rissige Haut

Folie Medias: Anzeichen von Nervenstörungen

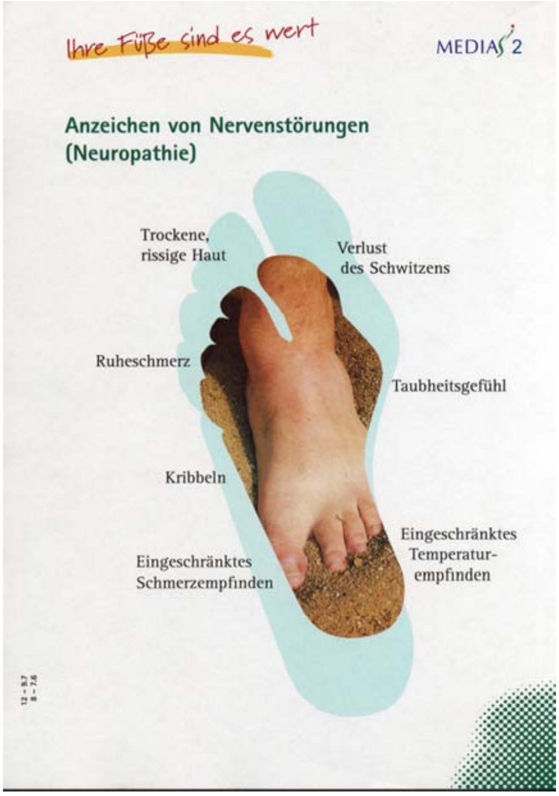

Erläutern Sie die Anzeichen von Durchblutungsstörungen an den Beinen, welche die Teilnehmer selbst registrieren können:

- Wadenkrämpfe und Schmerzen nach kurzen Gehstrecken; Linderung durch Stehen bleiben
- Blasse, dünne Haut
- Schwere, kraftlose Beine

Beschreiben Sie die möglichen Folgen von Durchblutungsstörungen an den Beinen.

Erläutern Sie, dass infolge von Durchblutungsstörungen die Nerven an den Beinen nicht mehr ausreichend mit Blut versorgt werden können – Wunden heilen somit schlechter ab. Bleibt eine rasche Behandlung aus, stirbt das Gewebe um die Wunde herum ab.

Zeigen Sie den Teilnehmern auf, dass Nerven- oder Durchblutungsstörungen sowohl einzeln, als auch gemeinsam auftreten können.

Wichtig ist, sich vor Augen zu führen, dass nicht der Diabetes Fußprobleme verursacht, sondern der unangemessene Umgang mit den Füßen! Wenn sich die Teilnehmer richtig um ihre Füße kümmern, sind Fußprobleme vermeidbar.

Folie Medias: Anzeichen von Durchblutungsstörungen an den Beinen

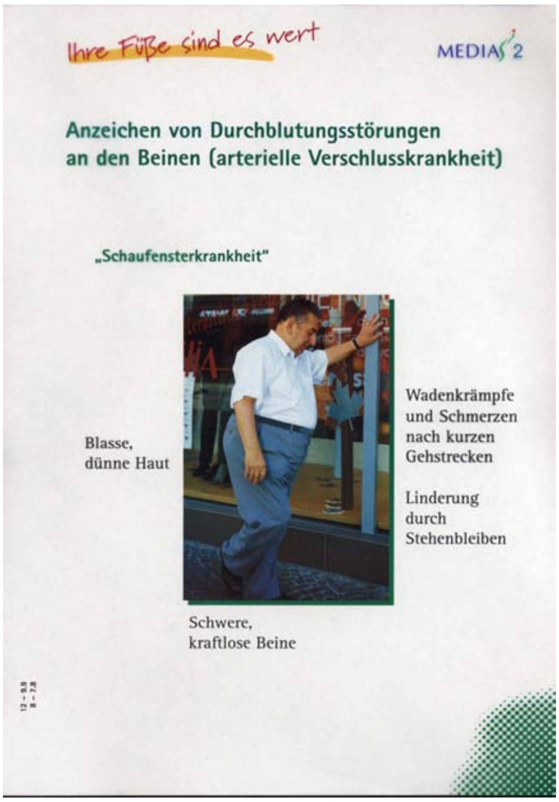

Begründen Sie den Teilnehmern, dass sich diabetische Fußprobleme zum Großteil vermeiden lassen, wenn Menschen mit Diabetes die geeigneten Schuhe und Strümpfe tragen. Regelmäßiges Kontrollieren der Füße macht es möglich, erste Anzeichen eines Fußproblems frühzeitig zu erkennen und rechtzeitig zu reagieren.

Informieren Sie die Teilnehmer über Merkmale geeigneter Schuhe. Begründen Sie jeweils gemeinsam mit den Teilnehmern warum die Beachtung dieser Merkmale sinnvoll ist.

Erläutern Sie anhand der Folie 12-9.13: Ein guter Schuh – von außen betrachtet

Ein guter Schuh:

- gibt der Ferse festen Halt
- hat keinen hohen Absatz
- lässt sich nicht leicht verdrehen
- hat weiches Obermaterial
- hat keine zu biegsame Schuhsohle
- hat keine Vorderkappe
- hat ausreichend Platz auch für die Zehen

Richten Sie nun mit den Teilnehmern das Augenmerk aufs Innere der Schuhe und stellen Sie Merkmale eines geeigneten Schuhs hervor. Begründen Sie auch hier, warum die Beachtung dieser Merkmale sinnvoll ist.

Ein guter Schuh:

- hat keine dicken Nähte oder Ösen
- hat keine hohen Absätze
- hat keine harten Stellen und Übergänge
- hat kein vorgefertigtes Fußbett
- hat kein zerrissenes Innenfutter

Folie Medias 12-9.15: Ein guter Schuh – von innen befühlt

Rufen Sie in Erinnerung, dass Personen mit Nervenstörungen Druckstellen und kleinere Verletzungen nicht richtig spüren können. Daher müssen die Augen und Hände die Aufgaben der Nerven übernehmen und Veränderungen an den Füßen registrieren.

Erläutern Sie im Einzelnen, worauf die Teilnehmer bei der Fußkontrolle achten sollen:

Folie: Die Füße täglich kontrollieren

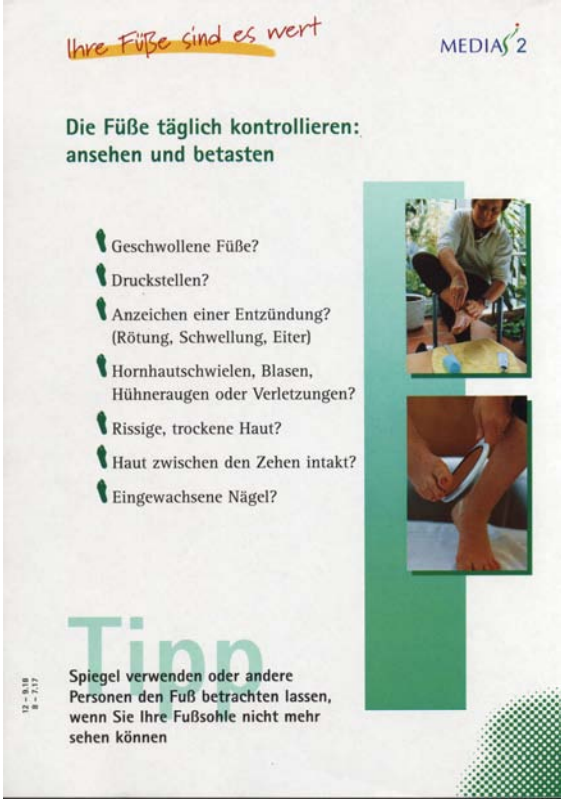

6.4 Kontrolluntersuchungen

Ziele

- Die Teilnehmer sollen erkennen, warum regelmäßige Kontrolluntersuchungen wichtig und notwendig sind: beginnende Veränderungen im Körper aufgrund von Diabetes sind nicht fühl- und spürbar und müssen deshalb regelmäßig kontrolliert werden.
- Auch können die Teilnehmer Risikofaktoren wie erhöhte Blutdruck- oder Blutfettwerte nicht wahrnehmen. Sie sind nur durch Kontrolluntersuchungen zu registrieren. Regelmäßige Kontrolluntersuchungen erlauben es, gezielt vorliegende Risikofaktoren und / oder Folgeerkrankungen zu behandeln.
- Die Einheit dient dazu, die Teilnehmer genauer über die Zielsetzung und den Gebrauch des Gesundheitspasses Diabetes zu informieren.

Vorgehen

Im Gesundheitspass Diabetes werden die Ergebnisse von regelmäßigen Kontrolluntersuchungen dokumentiert. Sie erläutern die wesentlichen Funktionen des Passes.

Anschließend erläutern Sie, welche Kontrolluntersuchungen wann und wie oft durchgeführt werden sollten.

Folie: Mein persönlicher Diabetes-Check-up

Geben Sie den Teilnehmern die Arbeitsblätter 16 und 17 mit nach Hause.

6.5 Feedback

Geben Sie den Kursteilnehmern die Möglichkeit noch Fragen zu stellen und eine Rückmeldung über den Kurs zu geben.

Verabschiedung.
