# Supplementary material for: Study protocol of the Diabetes and Depression Study (DAD): a multi-center randomized controlled trial to compare the efficacy of a diabetes-specific cognitive behavioral group therapy versus sertraline in patients with major depression and poorly controlled diabetes mellitus
Source: BMC Psychiatry. 2013 Aug 6;13:206. doi: 10.1186/1471-244X-13-206 (PMC3750698; doi:10.1186/1471-244X-13-206)
Supplement: Additional file 2 — Diabetes-specific CBT manual for therapists (in German). [file 1471-244X-13-206-S2.pdf]

Verhaltenstherapie

bei

Depression & Diabetes

Manual zur Gruppentherapie

(Das vorliegende Manual sowie alle weiteren Therapieunterlagen sind nur für den internen Gebrauch im Rahmen der DAD-Studie bestimmt.)

Einleitung

Das vorliegende Therapiemanual beschreibt überblicksartig die Inhalte und die therapeutische Vorgehensweise bei der Durchführung eines störungsspezifischen kognitiv-behavioralen Gruppentherapieprogramms zur Bewältigung der Depression und zur Verbesserung der Stoffwechseleinstellung (Senkung des HbA_{1c}) bei Menschen mit Diabetes (Typ 1 und Typ 2). Neben dem Einsatz verhaltenstherapeutischer Techniken und Therapiestrategien erfolgt eine gezielte Interaktionsgestaltung durch den Therapeuten, die sich an den Leitlinien einer verhaltenstherapeutischen Gruppenarbeit, wie sie von Fiedler (1996) formuliert wurden, orientiert. Im Anschluss an die Einleitung wird diese Interaktionsgestaltung gesondert beschrieben. Einzeltechniken (z. B. Techniken der kognitiven Umstrukturierung) werden aus Gründen der Ökonomie in ihrer praktischen Durchführung nicht gesondert beschrieben, da dies in der zitierten Originalliteratur nachzulesen ist. Die Durchführung des Therapieprogramms anhand des auf einer mittleren Detailebene verfassten Manuals setzt eine fundierte verhaltenstherapeutische Ausbildung voraus, und sollte nur von entsprechend qualifizierten Therapeuten durchgeführt werden.

Das Programm umfasst insgesamt 10 Sitzungen

Leitlinien der verhaltenstherapeutischen Gruppenarbeit

Die folgenden Überlegungen sind im Wesentlichen aus Ergebnissen zur Forschung klinisch-psychologischer Gruppentherapie abgeleitet (z. B. Liebermann et al., 1973; für eine Übersicht vgl. Fiedler, 1996).

Der nachfolgend beschriebene Einsatz interaktioneller Leitlinien dient vor allem zur gezielten Verbesserung der sog. instrumentellen Gruppenwirkfaktoren (Kohäsion, Vertrauen, Selbstöffnung, Arbeitsklima). Auch die Prävention häufige Therapeutenfehler, die sich ungünstig auf diese Faktoren auswirken können, soll durch sie erleichtert werden. Von zentraler Bedeutung ist dabei die Förderung der Eigenverantwortung der Patienten im Hinblick auf ihre Teilnahme an der Gruppentherapie im Sinne eines Selbstmanagement. Dies beinhaltet beispielsweise auch den Verzicht auf vorgegebene Gruppenregeln, wie sie in verhaltenstherapeutischen Gruppen häufig vorgesehen sind (zusammenfassend z. B. Fiedler, 1996).

Explizit formuliert wurden die interaktionellen Leitlinien, um die Reproduzierbarkeit dieser Aspekte des Gruppentherapiekonzepts, unabhängig von der Person des Behandlers zu erhöhen. Nicht zuletzt dient dies aber auch zur Klärung der Unterschiede bezüglich des Begriffs und der praktischen Umsetzung der „Interaktionsgestaltung“ durch den Therapeuten im Vergleich zu psychodynamischen Konzepten.

1 Allgemeine interaktionelle Leitlinien

Im folgenden werden die von Fiedler (1996) schlagwortartig „verdichteten“ Leitlinien kurz erläutert.

Gruppenverhaltenstherapie ist Einzeltherapie in der Gruppe!

Gruppentherapie wird dabei vorrangig als Einzelpsychotherapie für die beteiligten Patienten aufgefasst. Die persönlichen Probleme bzw. psychischen Störungen der einzelnen Patienten, die Anlass für die Teilnahme an der Therapie waren - im vorliegenden Fall Belastungen durch einen unzureichenden Umgang mit dem Diabetes - stehen im Vordergrund. Gruppengemeinsame Aktivitäten und Übungen stellen dabei förderliche Bedingungen für die „kollektive Einzeltherapie“ dar.

Patienten entscheiden selbst, ob, wann und wie sie mitarbeiten!

Jeder Patient entscheidet und verantwortet selbst, ob und wann und in welcher Weise er Gruppenangebote nutzt. Im Unterschied zum Einzelsetting, stellen Motivations- und Überzeugungsversuche in der Gruppentherapie stets eine Gefahr dar, die unter 3 genannten Therapeutenfehler zu begehen (z. B. Überforderung, ungefragtes Ansprechen von Problemen einzelner Patienten in Gruppe). Aus diesem Grund unterstützt der Therapeut Patienten, die aktuell nicht mitarbeiten wollen, wohlwollend in ihrer Zurückhaltung. Er schützt sie, auch um eine mögliche beginnende Außenseiterproblematik zu unterbinden, vor der Kritik anderer Gruppenteilnehmer an ihrer „unzureichenden Mitarbeit“.

Interessensunterschiede in der Gruppe haben Vorrang!

Nicht zuletzt um die Gruppenkohäsion zu erhöhen, unterbindet der Therapeut behutsam Versuche der Gruppenmehrheit, den Verlauf oder Eigenarten der Gruppenarbeit gegen die Interessen andere festlegen zu wollen. Interessensunterschiede haben immer dann bereits Vorrang, wenn

es im Prozess einer Entscheidungsfindung auch nur ansatzweise Uneinigkeit gibt. Der Therapeut unterstützt dabei eher Divergenzen und die Verbalisierung unterschiedlicher Interessen, um letztendlich nach Möglichkeit einen ***gruppengemeinsamen Konsens*** zu unterstützen. Im Hintergrund steht dabei das grundsätzliche Recht jedes Einzelnen, die Therapiegruppe nach seiner Art für sich zu nutzen. Der Therapeut übernimmt dabei die Aufgabe, das Recht auf Interessenunterschiede für alle zu unterstützen.

Interpersonelle Konflikte in der Gruppe löst jeder Patient für sich selbst!

Ergeben sich Konflikte der Patienten untereinander, wird die entstandene Konfliktsituation als jeweils individuelles interaktionelles Problem ***für jeden der Beteiligten getrennt*** aufgefasst und auch als solches behandelt. Es wird davon ausgegangen, dass die aktuellen Konflikte nicht selten auch Ähnlichkeiten zu Problemen aufweisen können, wie sie die Beteiligten auf unterschiedliche Weise auch außerhalb der Therapie haben. Die in der Einzelarbeit gefundenen individuellen Lösungen stellen möglicherweise auch eine Perspektive für die aktuelle Konfliktsituation in der Gruppe dar. Darüber hinaus steht es den Patienten frei, ihre Differenzen außerhalb der Therapiezeit auszutragen. Idealerweise können sie dabei in der Gruppe erlernte Problemlösestrategien unter Alltagsbedingungen einüben.

2 Funktion der Gruppe

Im Rahmen des vorliegenden Therapiekonzepts dient die Gruppe als Publikum oder Auditorium bei psychoedukativen Interventionen. Es handelt sich außerdem um ein „Übungsfeld“ zur Verbesserung zwischenmenschlicher Kompetenzen und Konfliktlösungsstrategien. Dabei ergeben sich auch Möglichkeiten der Reflexion persönlicher Probleme und Entwicklungsmöglichkeiten bezogen auf den Lebensalltag der Patienten.

3 Bedeutung und Prävention von Therapeutenfehlern

In den Forschungsergebnissen zur Wirksamkeit von Gruppenpsychotherapie lassen sich oft negative Wirkungen von Gruppentherapie nachweisen, wie sie sich im Varianzerweiterungseffekt und im Deteriorations-Effekt widerspiegeln.

Der Varianzerweiterungseffekt besagt dabei, dass auch wenn die Mittelwerte der Behandlungsgruppe denen der Kontrollgruppe überlegen sind, die Streuung von Erfolgsparametern vom Beginn bis zum Ende der Therapie in der Behandlungsgruppe zunimmt, während dies in den Kontrollgruppen oftmals nicht zutrifft. In diesem Fall profitieren die meisten Teilnehmer der aktiven Therapie, während es einigen anderen offensichtlich schlechter als vor der Behandlung geht (= Deteriorations-Effekt). Aufgrund der geringen Anzahl betroffener Patienten, ergibt sich dabei empirisch insgesamt ein positives Bild der Ergebnisse. Die Bedeutsamkeit des Varianzerweiterungseffektes zeigt sich beispielsweise in der Meta-Analyse von Grawe et al. (1994). Hier findet er sich in 40% der Untersuchungen, in denen entsprechende Angaben mitgeteilt werden.

In einer der empirisch am besten kontrollierten Studien (Liebermann et al., 1973) über Psychotherapiegruppen unterschiedlicher theoretischer Ausrichtungen wurde versucht, prädizierende Variablen für den Deteriorations-Effekt zu finden. Es ließen sich keine sicheren Prädiktoren aus der Teilnehmerstruktur oder den Interaktionsmustern und Verhaltensweisen der Teilnehmer untereinander ableiten. Die einzigen identifizierbaren Prädiktoren stellten spezifische Eigenarten und Verhaltens-

weisen der Therapeuten dar, unabhängig von deren theoretischer Orientierung.

Diese wurden von Liebermann, Yalom und Miles (1973) zu folgenden schädigenden Handlungsmustern zusammengefasst:

- Direkte oder unterschwellige Feindseligkeit der Gruppenleiter gegenüber einzelnen Patienten
- Zulassen wechselseitiger Aggressionen der Gruppenteilnehmer untereinander
- Interaktions- und Motivationsprobleme der Patienten direkt und ungefragt ansprechen
- Fehlende Solidarität des Therapeuten mit Außenseitern
- Überforderung einzelner Gruppenmitglieder
- Strikte Orientierung der Gruppenarbeit an vorgegebenen Gruppennormen und Interaktionszielen (z. B. die Erwartung, dass sich alle Patienten persönlich „öffnen" und beteiligen müssen)

Diese Handlungsmuster werden von Fiedler (1996) als „Therapeutenfehler" angesehen. Sie sollen im vorliegenden Therapiekonzept durch ihre explizite Thematisierung und Berücksichtigung, auch in Form der Leitlinien zur therapeutischen Interaktionsgestaltung, nach Möglichkeit vermieden werden.

4 Interaktionelle Probleme in der Therapie

Obwohl die Möglichkeiten besteht, anhand in der Gruppe auftretender interaktioneller Probleme exemplarisch neue Lernerfahrungen zu machen, die auch im Alltag der Patienten von Nutzen sein können, werden diese im Rahmen des hier dargestellten Therapiekonzepts eher als unerwünscht betrachtet. Angesichts der eingegrenzten Zielsetzung dieses Programms, wird der Behandlungsfokus vielmehr auf die Analyse und Bewältigung von Problemen im Lebensalltag der Patienten statt in der Therapiegruppe gelegt. Konsequenterweise erfolgen prophylaktische Maßnahmen des Therapeuten zur Vermeidung spezifischer Gruppenkonflikte. Treten diese dennoch auf, werden sie in jedem Fall beachtet und wie unter 2 beschrieben behandelt. Übersteigen diese Schwierigkeiten die Problemlösefähigkeiten der Beteiligten im Sinne einer krisenhaften Entwicklung, können bedarfsweise zwei bis drei kurze Einzelgespräche parallel zur Gruppentherapie angeboten werden. Dies gilt auch für den Fall anderer massiver Probleme einzelner Gruppenteilnehmer.

5 Leitlinien zur therapeutischen Interaktionsgestaltung

Aus den oben genannten Überlegungen lassen sich konkrete Leitlinien zur Interaktionsgestaltung durch den Therapeuten ableiten:

- Die generelle Haltung ist wohlwollend und unterstützend.
- Es wird kein Druck auf die Patienten bezüglich ihrer Mitarbeit ausgeübt.
- Offene oder unterschwellige Kritik am Verhalten des Patienten sollte nicht geäußert werden.
- Die Grundhaltung des Therapeuten beinhaltet eine hohe Akzeptanz bezüglich anderer Ansichten (z. B. auch über Krankheitsmodelle), Haltungen und Lebensstilen (auch wenn sie aus seiner Sicht problemverschärfend sind). Um Reaktanzphänomene zu minimieren, sollten Diskussionen im Sinne von „angestrengter Überzeugungsarbeit" vermieden werden. Angestrebt wird eine Erweiterung der Perspektiven und Veränderung der Gewichtungen dysfunktionaler Kognitionen, statt eine Ablehnung der Sichtweise des Patienten.

- Die Strategie zum Umgang mit offenen oder versteckten Vorwürfen, Angriffen und Entwertungen der Therapie bzw. des Behandlers durch Patienten lässt sich als „offensiv“ charakterisieren. Damit ist gemeint, dass der Therapeut gegebenenfalls selbst ansprechen sollte, dass Zweifel und Kritik spürbar sind. Er verstärkt offen formulierte Kritik als „engagierte, eigenverantwortliche Form der Mitarbeit“ und bemüht sich, gemeinsam mit der gesamten Gruppe, eine möglichst konstruktive Klärung herbeizuführen.
- Wiederholte, angemessen formulierte, theoretische Begründungen für die Vorgehensweise werden häufig eingebracht.
- Der Therapeut vermittelt klare und konkret formulierte Informationen über Veränderungsmöglichkeiten.
- Es sollte stets für alle Patienten nachvollziehbar sein, welche Ziele in der laufenden Gruppenarbeit anstrebt werden. Dabei dient die hohe Prozess-Transparenz der Begründung für die Interventionen, sie soll nicht mit dem Druck einhergehen, die zugrunde liegenden Ansichten und Modelle teilen zu müssen.

1. Sitzung

- Vorstellung des/r Therapeut/in
- Überblick über die Sitzung
- Vorstellung der Teilnehmer
- Vorstellung des Gruppenprogramms
- Vorkommen depressiver Störungen mit Bezug auf Diabetes
- Wie beeinflussen sich Aktivitäten und Stimmung?
- Positive Aktivitäten
- Übungsaufgaben
- Sitzungsbewertung

1.1 Vorstellung des Therapeuten (5´)

Kurze Angaben zum eigenen Hintergrund, bisherige Erfahrungen in den Bereichen Depression und Diabetes. Wichtig dabei ist, auf eine unaufdringliche Weise Kompetenz und Offenheit zu vermitteln.

1.2 Überblick Sitzung (5´)

Folie 1-2: Die Themen werden auf einer Powerpoint-Folie kurz dargelegt und erläutert.

1.3 Vorstellung der Gruppenteilnehmer (40´)

Folie 1-3: Das Kennen lernen erfolgt über **Paarinterviews**: Zwei Teilnehmer bilden jeweils ein Paar (ggf. eine Dreiergruppe).

Jeder macht dem Partner gegenüber einige Angaben zur eigenen Person (z.B. über den Diabetes, seine aktuelle Lebenssituation, etc.), wobei jeder nur das erzählt, was er auch wirklich mitteilen möchte.

Zusätzlich sollen die Erwartungen und Zielvorstellungen bezüglich der Behandlung erläutert werden. Die Teilnehmer werden instruiert, sich ausreichend Zeit pro Partner zu nehmen.

Jeder Gruppenteilnehmer wird anschließend durch seinen Partner in der Gruppe vorgestellt.

Nach jeder Vorstellung sollte gefragt werden, ob der Vorgestellte noch etwas ergänzen möchte.

1.4 Pause (10´)

1.5 Vorstellung des Gruppenprogramms (20´)

a) Ziele

Folie 1-4: Ziele der Behandlung kurz vorstellen, dabei erklären, dass insbesondere auf die konkreten Ziele (HbA_{1c} um einen Prozentpunkt senken) im Verlauf des Programms genauer eingegangen wird.

Wichtige Anmerkung für den/die Therapeut/in: Im optimalen Fall hat jeder Teilnehmer schon vorbereitend konkrete Empfehlungen zur Senkung des HbA_{1c} von seinem behandelnden Arzt eingeholt. Die entsprechenden Ärzte und Teilnehmer wurden zuvor von dem/der Studienmitarbeiter/in über diesen Therapiebaustein informiert und gebeten, die Ergebnisse der Arzt-Patientbesprechung zu protokollieren (AB 4). Diese sollen dann in der 2. Sitzung bei der Festlegung der Therapieziele berücksichtigt werden.

An dieser Stelle kann ggf. ein kurzer Hinweis auf die o.g. Vorarbeit (die individuellen Empfehlungen des Arztes zur Senkung des Hba_{1c}) und weitere Einbindung dieser Ergebnisse erfolgen. Dadurch wird den Teilnehmern das Ineinandergreifen der Diabetes- und der Verhaltenstherapie verdeutlicht (s.a. Übungsaufgaben der 1. Sitzung).

b) Grundprinzipien

Folie 1-5:

1. **Voraussetzungen**: Verbindlichkeit der Teilnahme an allen Sitzungen, Wichtigkeit der Übungen zwischen den Sitzungen und Schweigepflicht bezüglich personenbezogener Daten der Gruppenmitglieder erläutern bzw. vereinbaren. An dieser Stelle wird darauf hingewiesen, dass das Programm eine wirksame Behandlung darstellt, sie jedoch nur dann wirksam ist, wenn den Übungen zwischen den Sitzungen eine hohe Priorität im Alltag eingeräumt wird. Diese Bereitschaft sollte zumindest für die Dauer der Behandlung vorhanden sein, ansonsten ist eine Besserung nicht zu erwarten.
2. Ausgabe der **Terminpläne**.
3. Das **Persönliche Begleitbuch** verteilen. Es enthält alle wichtigen Informationen zum Nachlesen und -arbeiten. Die Teilnehmer werden darauf hingewiesen alle Unterlagen zu jeder Gruppensitzung mitzubringen.
4. **Telefonliste** (für alle diejenigen, die damit einverstanden sind, dass ihre Telefonnummer den anderen Gruppenmitgliedern gegeben wird). Festlegung eines Verantwortlichen bei Terminabsagen.

c) Überblick über Gliederung einer Sitzung

Folie 1-6: Hier wird die Gliederung/Struktur der einzelnen Stunden dargelegt.

d) Überblick über die Schwerpunktthemen der Sitzungen

Folien 1-7/1-8/1-9: Anhand der Powerpoint-Folien werden die Schwerpunktinhalte der 10 Sitzungen kurz vorgestellt. Besonderer Hinweis auf den leeren Block in der 9. Sitzung, den die Teilnehmer mit einem Wunschthema füllen können.

1.6 Vorkommen depressiver Störungen mit Bezug auf den Diabetes (10´)

Folie 1-10: Anhand der Grafik die Prävalenz von Depressionen erläutern, erhöhtes Risiko für Diabetespatienten nennen. Stoffwechseleinstellung bei depressiven Diabetikern im Vergleich zu nichtdepressiven Diabetikern erläutern. Das Rational der Therapie schlussfolgern.

Z.B. Depressionen kommen in Deutschland häufig vor, bei Diabetikern sind Depressionen sogar doppelt so häufig zu finden wie bei Menschen ohne Diabetes. Die Gründe dafür werden wir in der nächsten Stunde genauer besprechen. Der Diabetes kann eine Depression auslösen oder diese aufrechterhalten. Sehr häufig geht eine Depression bei Diabetikern mit einer schlechten Stoffwechseleinstellung einher. Haben Sie eine Idee, wieso das so sein könnte?

(Es können kurz die Vorschläge gesammelt werden, sollten aber nicht auf der Flipchart notiert werden, da in der 3. Sitzung ausführlich der Zusammenhang zwischen Depressionen und Diabetes behandelt wird.)

Depressionen gehen mit Freudlosigkeit und Interessenlosigkeit einher und depressive Diabetiker sehen oft nicht so viel Sinn darin, sich intensiv um das Messen und Spritzen zu kümmern. Kennen Sie das von sich?

Allein durch die Depressionen leidet die Lebensqualität sehr und der schlecht eingestellte Diabetes führt dann häufig noch zusätzlich zu Schuldgefühlen, so dass die Lebensqualität noch mehr leidet. Dies führt dann auch dazu, dass man insgesamt unsicher wird und sich von Freunden und Bekannten zurückzieht. Wir gehen auf

den Zusammenhang zwischen dem Diabetes und Depressionen in der 3. Sitzung genauer ein.

Unser Ansatzpunkt wird sein, Ihnen zunächst das nötige Wissen zu Diabetes und Depressionen zu vermitteln und dann Strategien zu finden, die helfen zunächst die Depression zu verbessern...

1.7 Wie beeinflussen sich Aktivitäten und Stimmung? (10´)

Folie 1- 11 (Abwärtsspirale) /Folie 1-12 (Aufwärtsspirale): Wechselwirkung von Stimmung und Aktivitäten erläutern, dabei das Rational des folgenden Therapieschrittes (Aktivitätenaufbau) einbinden.

...Dazu gehört, Ihnen dabei zu helfen, wieder mehr Positives zu erleben und auch mehr Positives in Ihrem Leben zu entdecken. Damit soll es Ihnen auch wieder möglich werden, sich besser um den Diabetes zu kümmern. Insgesamt soll damit Ihre Lebensqualität wieder deutlich verbessert werden. Das erscheint Ihnen zur Zeit vielleicht plausibel, aber nicht umsetzbar. Sie haben möglicherweise selbst schon ausprobiert, etwas zu unternehmen, das Ihnen gut tun sollte, ohne, dass es Ihnen Freude bereitet hat. Vielleicht können sie sich im Moment aber auch gar nicht vorstellen, dass sie überhaupt Spaß oder Freude an etwas empfinden. Das ist aus Ihrer Perspektive sehr verständlich, versuchen Sie zunächst einmal die nächsten Schritte auf sich zukommen zu lassen. Auch wenn sie es sich eventuell jetzt noch nicht vorstellen können, ist es sehr wahrscheinlich, dass sie schon bald profitieren werden.

Zunächst werden wir damit beginnen, einige für Sie positive Aktivitäten zu sammeln. Im weiteren Verlauf des Gruppenprogramms werden wir Ihnen vorschlagen, diese wieder in Ihren Alltag zu integrieren. Denn wenn Sie wenig Positives erleben, können Sie sich an wenigen Dingen erfreuen. Zudem verbringen Sie oft viel Zeit mit Grübeln. Dadurch wird man noch depressiver und man ist noch weniger aktiv. Man kann sich das folgendermaßen vorstellen... (Folie 1-11)***.***

Es gibt dementsprechend auch eine positive Spirale... (Folie 1-12).

Wer von Ihnen hat das schon mal so erlebt, wie es hier abgebildet ist?

1.8 Positive Aktivitäten (10´)

Folie 1-13: Anhand einiger Beispiele werden positive Aktivitäten oder Ereignisse identifiziert. Die Art der Aktivität kann individuell sehr unterschiedlich sein. Es geht darum, eine Art persönliches Menü zu erstellen, aus dem ab der 2. Sitzung jeder versuchen kann, sich öfter zu bedienen. Exemplarisch werden einige Beispiele in der Gruppe gesammelt. Es ist darauf zu achten, dass genügend realistische Aktivitäten dabei sind, deren Eintreten nicht zu sehr von exogenen Faktoren abhängt. Jeder bekommt dazu das AB 1 „Meine persönliche Liste positiver Aktivitäten“, welches dann als Übungsaufgabe (s. u.) komplettiert werden soll.

1.9 Übungsaufgaben (10´)

Folie 1-14:

1. Die persönliche Liste (AB 1) soll individuell zuhause komplettiert werden. Der Therapeut erklärt, dass es zunächst nur um die Erstellung eines Menüs geht, der zweite Teil des Arbeitsblattes (Protokollierung der tägl. Anzahl der jeweiligen Durchführung einer Aktivität) ist Übungsaufgabe ab der 2. Sitzung. Wer möchte, kann als Hilfestellung zur Generierung der Aktivitäten das AB 2 „Liste angenehmer Ereignisse“ benutzen.

2. Die Teilnehmer sollen außerdem auf dem AB 3 „Stimmungsbeobachtung" fortlaufend protokollieren, was sie ca. stundenweise gemacht haben und welche Stimmung sie dabei hatten. Dies stellt eine Baseline-Beobachtung dar.

3. Falls einer oder mehrere Teilnehmer mit seinem/ihrem Arzt, der den Diabetes behandelt, noch keine konkreten Verhaltensweisen zur Senkung des HbA_{1c} (AB 4 „Empfehlungen zur Senkung des HbA_{1c}") besprochen haben, werden diese ermutigt, das möglichst schnell nachzuholen. Es kann darauf hingewiesen werden, dass die Empfehlungen des Arztes insbesondere in der 2. Sitzung berücksichtigt werden sollen.

Der Therapeut betont, dass es wichtig ist, dass alle Unterlagen zum Gruppenprogramm zu jeder Sitzung mitgebracht werden!

1.10 Sitzungsbewertung (5´)

Jeder, der will, kann eine kurze Rückmeldung zur Sitzung geben. Die Beiträge werden nicht diskutiert, sondern jede Meinung annehmend stehen gelassen.

Formulierungsvorschläge: ***Wer möchte, kann jetzt gerne noch kurz etwas zur heutigen Sitzung sagen.***

Sind Sie mit der heutigen Sitzung zufrieden?

Jeder Teilnehmer füllt den **Rückmeldebogen** zur Sitzung aus.

Der Therapeut bewertet ebenfalls die Sitzung auf dem entsprechenden Bewertungsbogen.

2. Sitzung

- Rückblick auf die 1. Sitzung
- Überblick über die Sitzung
- Besprechung der Übungsaufgaben
- Wie entstehen Depressionen und wie werden sie aufrechterhalten?
  a) Was ist eine Depression?
  b) Entstehung einer Depression (prädisponierende, auslösende, aufrechterhaltende Faktoren)
  c) Aufrechterhaltung einer Depression
- Vereinbarung von (Therapie-) Zielen
- Übungsaufgaben
- Sitzungsbewertung

2.1 Rückblick auf die 1. Sitzung (5´)

Es wird eine kurze Zusammenfassung der Inhalte und Ergebnisse der ersten Sitzung gegeben.

Nachfrage, ob etwas aus der ersten Sitzung offen geblieben und besprochen werden müsste.

Überblick über die Themen der heutigen Sitzung (Folie 2-1).

2.2 Besprechung der Übungsaufgaben (15´)

Die zu Hause noch aufgelisteten positiven Aktivitäten können vorgestellt werden.

Sind Ihnen zuhause noch Aktivitäten eingefallen, die Ihnen Spaß machen könnten? (Gelegenheit geben, sich zu äußern.)

Vielleicht hat jemand noch etwas gefunden, was er früher einmal gerne unternommen hat und was er nun aufleben lassen will?

Was haben Sie bei der Stimmungsbeobachtung bemerkt? Ist Ihnen etwas aufgefallen?

An dieser Stelle der Therapiesitzung ist es sinnvoll, routinemäßig nach Problemen der Diabetestherapie zu fragen. Daraufhin sollte die/der Therapeut/in entweder auf entsprechende Übungsinhalte zukünftiger Sitzungen verweisen oder, falls dies erforderlich erscheint, den Teilnehmer ermutigen, seinen Arzt aufzusuchen. Zum Beispiel könnte ein Teilnehmer Schwierigkeiten beim Korrigieren seiner hohen Blutzuckerwerte durch Insulinspritzen benennen. Lassen sich diese Schwierigkeiten z.B. auf einen Antriebsmangel des Patienten zurückführen, sollte die Therapeutin auf die verhaltenstherapeutischen Techniken verweisen, die im Laufe des Gruppenprogramms erlernt werden. Ist die Ursache dieser Schwierigkeiten allerdings ein direktes diabetologisches Problem (z.B. mangelnde Kenntnis über die Berechnung von Insulineinheiten zur Blutzuckerkorrektur) sollte der Teilnehmer dringend ermutigt werden, sich in seiner Arztpraxis Unterstützung zu holen.

2.3 Wie entstehen Depressionen und wie werden sie aufrechterhalten? (40´)

a) Was ist eine Depression?

Rekapitulation der Äußerungen der Teilnehmer aus der letzten Stunde. Sammlung weiterer Symptome durch die Teilnehmer anhand der 6 Symptomebenen analog Hautzinger (somatische, motorische, emotionale, motivationale, kognitive und interaktive Beschwerden) sortieren. Es werden dazu die Folie 2-2 (6 Ebenen)

und Folien 2-3, 2-4, 2-5 genutzt. Der Therapeut gestaltet diesen Teil möglichst interaktiv mit Bezug zum eigenen Erleben der Teilnehmer.

b) Entstehung von Depression

Kurze Rekapitulation der epidemiologischen Zahlen.

Z.B.: ***Sie haben in der letzten Stunde gesehen, dass relativ viele Menschen an einer Depression leiden. Sie haben auch gesehen, dass ungefähr doppelt so viele Diabetiker unter Depressionen leiden wie Menschen ohne Diabetes.***

Nun stellt sich die Frage, wie es überhaupt zu einer Depression kommt. Man unterscheidet dabei genetische Faktoren, also Faktoren, die einem sozusagen in die Wiege gelegt werden, Faktoren, die einen Ausbruch der Krankheit begünstigen und Faktoren, die dafür verantwortlich sind, dass man z.T. über längere Zeit an der Depression leidet.

Folie 2-6:

„Prädisponierende" Faktoren: ***Damit sind Faktoren gemeint, die es wahrscheinlicher machen, dass man später im Verlauf seines Lebens eine Depression bekommt. Hierzu zählen Veränderungen bei einigen Genen, was dazu führt, dass man anfälliger für den Ausbruch von Depressionen wird. Es heißt nicht, dass man auf jeden Fall krank wird. Ein Indiz für eine genetische Belastung kann sein, dass in der Familie ungewöhnlich viele Verwandte depressiv geworden sind.***

Zu diesen „prädisponierenden" Faktoren zählen aber auch chronische, lang andauernde Belastungen und Überlastung. Z.B. können andauernde Erkrankungen während des Säuglingsalters oder belastende Erlebnisse in der Kindheit, wie z.B. Misshandlungen zu einer Störung der Stressverarbeitung führen, d.h. dass man noch im Erwachsenenalter empfindlicher auf Stress reagiert. Das wiederum begünstigt die Entstehung einer Depression.

Folie 2-7:

Auslösende Faktoren: ***Meist sind es Ereignisse, die wir als "Stress" oder Überforderung empfinden, z.B. der Tod eines nahe stehenden Menschen, Verlust des Arbeitsplatzes, Umzug an einen anderen Ort, oder auch eine Krankheit wie der Diabetes oder das Auftreten von Folgeerkrankungen, das heißt eine Verschlimmerung einer Erkrankung. Wie war das bei Ihnen, können Sie sich erinnern?***

Folie 2-8:

Aufrechterhaltende Faktoren: ***Bestimmte Faktoren können dazu führen, dass depressive Verstimmungen länger andauern, z.B. die ständige Sorge um die eigene Gesundheit oder das Erleben, dass das Bemühen um gute Blutzuckerwerte nicht zu den erhofften Werten führt. Dieses Gefühl von Hilflosigkeit kann man aber auch in anderen Bereichen erleben, z.B. bei der Arbeit, wo vielleicht die Anerkennung für viel Einsatz ausbleibt oder auch im Privatleben. Wie ist das bei Ihnen?***

Als Modell der Aufrechterhaltung wiederholt der Therapeut von der letzten Sitzung die Abwärtsspirale (Folie 1-11). Passivität und Antriebshemmung führen zu verminderter Aktivität, was wiederum dazu führt, dass man wenig Schönes/Positives erlebt. Dies wiederum macht einen noch depressiver und trauriger, was dann wieder dazu führt, dass man sich umso weniger überwinden kann, etwas Schönes zu unternehmen. Umgekehrt führen regelmäßige positive Aktivitäten zu einer Besserung der Depression.

(Im nächsten Schritt folgt die Darstellung des Zusammenhangs zwischen Verhalten, Emotionen und Kognitionen, der ebenfalls als aufrechterhaltender Mechanismus dargestellt wird.)

c) Aufrechterhaltung von Depressionen: Zusammenhang Denken, Fühlen und Handeln

Folie 2-9: Vorstellung des kognitionstheoretischen Ansatzes nach Beck.

Z.B. ***Ein Kennzeichen von Depressionen ist auch, das man verstärkt negative Gedanken hat. Die Gegenwart erscheint einem dunkel und schlecht, ebenso schätzt man auch die Zukunft ein. Depressive Menschen machen sich selbst große Vorwürfe wegen unterschiedlichster Dinge und denken schlecht über sich selbst. Auch ihre Umgebung erscheint ihnen in einem negativen Licht. Dies führt dazu, dass man sich auch schlecht fühlt, da Gedanken und Gefühle zusammenhängen. Und Gedanken und Gefühle haben wiederum einen Einfluss auf unser Verhalten. Da man Gefühle an sich nur schwer direkt verändern kann, setzt man bei der Therapie von Depressionen bei den Gedanken an. Darüber werden wir in der nächsten Stunde noch genauer reden.***

Den Teilnehmern soll damit ein grundlegendes Verständnis zur Depressionsentstehung und –aufrechterhaltung vermittelt werden, aus dem sich nachvollziehbar erste Ziele und die dazugehörigen folgenden Schritte ableiten lassen.

2.4 Pause (10´)

2.5 Vereinbarung von Therapiezielen (30´)

Kurze Einführung zur Wichtigkeit der Festlegung von Therapiezielen, ebenso zur Unterscheidung von kurz-, mittel- und langfristigen Zielen.

Z.B. ***Wir alle nehmen uns immer wieder Dinge vor, die wir erreichen wollen. Manche davon beziehen sich auf Dinge, die wir schnell erreichen können oder wollen, andere beziehen sich auf einen längeren Zeitraum oder sind sogar so genannte Lebensziele.***

In der Therapie ist es günstig, wenn Ziele einigermaßen realistisch sind, d.h., dass wir auch die Chance haben, sie erreichen zu können ("Ich möchte immer einen perfekten Blutzucker haben" oder "ich möchte, dass es mir immer gut geht" sind wahrscheinlich für die meisten Menschen nur schwer erreichbar). Manchmal nehmen wir uns auch Ziele vor, die so allgemein formuliert sind, dass wir gar nicht richtig überprüfen können, ob wir sie erreicht haben, z.B. "Ich will glücklich werden im Leben!". Oder wir wollen hauptsächlich Dinge erreichen, die erst in ferner Zukunft wahr werden können. Dabei kann einem auf dem Weg dahin der Atem ausgehen.

Wichtig ist uns, dass Sie sich über einige Therapieziele klar werden, die Sie in folgenden Zeiträumen erreichen können und wollen. Überlegen Sie sich ein Therapieziel, das Sie in den nächsten 4 Wochen erreichen können, eines, das Sie im Verlauf von Monaten und eines, das sie in einem längeren Zeitraum erreichen können und wollen.

Sie haben schon erfahren, dass es wichtig ist, einige Aktivitäten einzuplanen und regelmäßig durchzuführen. Bitte formulieren Sie dazu ein Therapieziel (z.B. ich möchte zwei Mal in der Woche eine halbe Stunde spazieren gehen).

Unser zweiter Behandlungsansatz ist eine Senkung Ihres HbA$_{1c}$. Es ist uns auch wichtig, dass sich Ihre Stoffwechsellage bessert. Man hat herausgefunden, dass sich die Senkung des HbA$_{1c}$ um ca. einen Prozentpunkt das Risiko für Komplikationen und Folgeerkrankungen drastisch reduziert.

Der Therapeut nennt anhand Folie 2-10 die Ergebnisse der Studie zur Senkung des HbA$_{1c}$.

Sie sehen also wie lohnenswert es ist, an der Verbesserung Ihres Stoffwechsels zu arbeiten. Wir empfehlen Ihnen daher ganz besonders, auch zu diesem Thema mindestens ein Therapieziel zu formulieren. Falls es Ihnen möglich war, zuvor mit Ihrem Arzt zu sprechen und sich Empfehlungen zu notieren, sollten sie diese nun hinzuziehen.

Nach 4 Wochen, also in der 6. Sitzung, werden wir dann gemeinsam schauen, inwieweit sie ihre kurzfristigen Ziele umsetzten konnten.

Auf AB 5 formuliert jeder Teilnehmer mindestens ein kurzfristiges, ein mittelfristiges und ein langfristiges Therapieziel.

Wichtig dabei ist, dass jeder ein Ziel zum Aktivitätenaufbau einplant und ein Ziel bzgl. des HbA_{1c} (unter Berücksichtigung des AB 4). Hier sollte der/die Therapeut/in darauf hinweisen, dass es ein wichtiger Bestandteil der therapeutischen Vorgehensweise ist, das AB 4 bei jedem Arztbesuch zu besprechen.

> **Zur Information für Therapeuten:** Therapieziele hinsichtlich der Senkung des HbA_{1c} können z.B. folgende sein: regelmäßige Selbstkontrolle des Blutzuckers (mit Tagebuch), gesunde Ernährung, regelmäßige Korrektur des BZ durch Insulinspritzen, körperliche Aktivität (regelmäßige Bewegung im Alltag (Richtwert: 0,5 Std./Tag oder 2,5 Std./Woche mäßig intensive Bewegung)).

Anhand der Regeln auf Folie 2-11 wird beispielhaft ein Therapieziel eines Teilnehmers besprochen.

Die kurzfristigen Ziele beziehen sich auf die nächsten 4 Wochen. In der 6. Stunde wird bzgl. dieser kurzfristigen Ziele gemeinsam eine Zwischenbilanz gezogen

2.6 Vorstellen der Übungsaufgaben (15´)

Folie 2-12:

1. Komplettieren der Therapieziele (AB 5).
2. Ab heute werden die pro Tag ausgeführten positiven Aktivitäten auf der persönlichen Liste (AB 1) protokolliert. Hinweis auf eine <u>angemessene</u> Erhöhung des Ausmaßes an positiven Aktivitäten.
3. Ausgabe und Erklärung des AB 6 „Protokoll positiver Aktivitäten und täglicher Stimmung“. An einem Beispiel wird die Handhabung der Arbeitsblattes verdeutlicht, zur Vereinfachung kann eine Tageslichtschreiber-Folie mit dem Arbeitsblatt hinzugezogen werden. Der Therapeut erklärt ebenfalls den Wegfall des bisherigen Stimmungsprotokolls (AB 3).

AB 1 und 6 werden bis zur 10. Sitzung weitergeführt (Tag 1 – 58)!

2.7 Sitzungsbewertung (5´)

Zur Sitzungsbewertung s. Sitzung 1 (1.10).

3. Sitzung

- Rückblick auf die 2. Sitzung
- Überblick über die Sitzung
- Besprechung der Übungsaufgaben
- Wie können sich sich Diabetes und Stimmung beeinflussen?
  a) Diabetes als möglicher ätiologischer Faktor
  b) Auswirkungen einer Depression auf den Diabetes
  c) Körperliche Wechselwirkung
- Wie beeinflussen sich Gedanken und Stimmung?
- Ungünstige Gedanken verändern
- Übungsaufgaben
- Sitzungsbewertung

3.1 Rückblick auf die 2. Sitzung (5´)

Es wird eine kurze Zusammenfassung der Inhalte und Ergebnisse der zweiten Sitzung gegeben.

Nachfrage, ob etwas offen geblieben ist und besprochen werden müsste.

Überblick über die Themen der heutigen Sitzung. Folie 3-1

3.2 Besprechung der Übungsaufgaben (30´)

1. AB 5 „Therapieziele". Falls hinsichtlich der diabetesbezogenen Ziele Klärungsbedarf mit dem Arzt besteht, werden die entsprechenden Teilnehmer ermutigt, dem nachzugehen.

   Hier ist es ebenfalls sinnvoll, nach Problemen der Diabetestherapie - unabhängig der festgelegten Ziele - zu fragen, und bei Bedarf ebenfalls zu einem Arztgespräch zu motivieren.

2. Intensive Besprechung des AB 6: Beobachtungen der Teilnehmer zu Auslösern depressiver Stimmung und Fluktuationen der Stimmung werden gesammelt (es kann auch noch einmal das AB 3 der ersten Woche hinzugenommen werden).

   Besonders ist darauf zu achten, welche Veränderungen sich durch Aktivitäten ergeben. Es soll herausgearbeitet werden, dass Aktivitäten in der Regel zu Stimmungsaufhellungen führen. Ein Fokus sollte ebenfalls sein, dass Aktivitäten im Sinne gesteigerter Bewegung zu einer Verbesserung der Blutzuckerwerte führen. Hinweis auf ein angemessenes Niveau der Aktivitätensteigerung.

3.3 Wie können sich Diabetes und Stimmung beeinflussen? (25´)

Kurze Rekapitulation der theoretischen Einheit der ersten Stunde mit Einbezug der Teilnehmer.

Kurze Darstellung der Interaktionen zwischen Depression und Diabetes. Dabei Wechselwirkung deutlich machen:

a) Diabetes als möglicher ätiologischer Faktor

Folie 3-2: Integration des Diabetes in das Depressionsmodell aus der vergangenen Sitzung. Diabetes könnte als kritisches Lebensereignis und damit als auslösender Faktor für die Entwicklung einer Depression oder als möglicher chronischer Stressfaktor eingeordnet werden.

Z.B. ***Wir haben uns in der letzten Stunde schon ein bisschen mit diesem Thema befasst. Wenn Sie sich zurückerinnern,***

wie war das denn bei Ihnen? Bei wem war der Diabetes schon bekannt, bevor die Depression entstanden ist? Haben Sie den Eindruck, der Diabetes hat etwas mit der Depression zu tun? Was genau?

Es soll dabei mit den Teilnehmern herausgearbeitet werden, dass die Diabetesdiagnose einen Umbruch im Leben bedeuten kann, der mit Stress einhergehen kann, vor allem bei einem akuten Beginn wie er beim Typ 1 Diabetes vorkommt. Die erste Zeit nach der Diagnose mit den Schulungen und den Versuchen der Diabeteseinstellung erfordern ein nicht zu unterschätzendes Adaptationsverhalten, das zu Überforderungen führen kann. Auch initiale Fehlversuche, den Diabetes zu kontrollieren, können im Sinne erlernter Hilflosigkeit depressive Gefühle auslösen. Die Erfahrungen der Teilnehmer sollen in diesem Sinne gesammelt und vor diesem Hintergrund erklärt werden.

Z.B. ***Wenn man eine schwere akute oder eine chronische Erkrankung bekommt, dann bedeutet das für jeden Menschen, dass er einige Zeit braucht, um sich auf diese Erkrankung einzustellen. Manche Menschen haben schon Verwandte mit Diabetes erlebt und mitbekommen, dass damit Folgeerkrankungen verbunden sein können und die Gedanken daran machen Angst. Man will das nicht erleben, was der Verwandte mitgemacht hat. Vielleicht erscheint es einem zunächst unausweichlich und dies kann dazu führen, dass man sich hilflos fühlt und das Gefühl bekommt, dass einem dieses Schicksal ereilen wird, ganz egal, was man tut. Solche Gedanken kommen Ihnen nun bekannt vor, denn wir haben in der ersten Stunde schon darüber gesprochen, dass dies typische depressive Gedanken sind.***

Andere haben vielleicht solche angst- und depressionsmachende Gedanken am Anfang gar nicht gehabt, haben aber vielleicht bald das Gefühl gehabt, mit dem Diabetes nicht leben zu können, weil man so viele Dinge bedenken und beachten muss. Und dann ist es natürlich so, dass gerade in der ersten Zeit nach der Diagnose viel auf einen einstürmt, Selbstkontrollmessungen, Medikamente nehmen oder Insulin spritzen, Schulungen mitmachen und anderes mehr. Da kann man den Eindruck bekommen, dass man das alles gar nicht schafft. Und vielleicht hatte der eine oder andere von Ihnen den Gedanken, dass er das nicht kann und vielleicht auch gar nicht will. Dabei kann schnell der Wunsch entstehen, die Bettdecke über sich zu ziehen und am liebsten nichts mehr zu tun. Auf so unterschiedliche Weise kann die Diabeteserkrankung zu einer Depression führen.

b) Auswirkungen einer Depression auf den Diabetes/die Diabeteseinstellung

Folie 3-3: Eine andere Art des Zusammenhangs ergibt sich, wenn zunächst die Depression vorhanden war und zeitlich danach eine Diabeteserkrankung festgestellt wurde. Fragen, die in diesem Zusammenhang zu klären sind:

Hat denn jemand von Ihnen schon eine Depression gehabt, bevor der Diabetes festgestellt wurde?

Wie war denn die Zeit vor der Diabeteserkrankung? Wie ging es Ihnen? Was haben Sie denn da gemacht?

Wie erging es Ihnen, als der Diabetes dann erkannt worden ist? Wie leicht bzw. schwer ist es Ihnen gefallen, die empfohlenen Verhaltensweisen zur Behandlung des Diabetes umzusetzen?

Anhand dieser Fragen sollen die Konsequenzen für den Umgang mit Diabetes aus dem Modell abgeleitet werden (z.B. Vernachlässigung von BZ-Messungen, Reduktion von Bewegung etc.).

Z.B. ***Wir haben schon besprochen, dass man bei einer Depression den Gedanken***

hat, dass alles sowieso keinen Sinn hat und man sich daher um viele Dinge natürlich auch nicht bemüht. Das kann dazu führen, dass man es aufgibt, die Blutzuckerwerte zu kontrollieren, da man glaubt, dass es sowieso nichts nützt. Oder dass einem die Gesundheit egal ist, weil das ganze Leben ja keinen Sinn hat. Dann ernährt man sich meist auch nicht gesund, sondern isst das, was schnell geht oder was man sich mal nebenher problemlos besorgen kann. Auch die Antriebslosigkeit, die zu einer Depression gehört, führt dazu, dass man sich nur schwer aufraffen kann, etwas zu tun, vor allem führt sie häufig dazu, dass man sich wenig oder gar nicht körperlich bewegt. Wenn dies über eine lange Zeit so geht, kann das die Entstehung eines Typ 2 Diabetes befördern, denn wenig Bewegung und ungünstiges Essverhalten sind Risikofaktoren für die Entstehung eines Typ 2 Diabetes.

Andererseits kann die Depression dazu führen, dass man die Selbstkontrolle unterlässt und sich wenig bewegt, obwohl der Arzt dies empfohlen hat und man selbst auch weiß, dass das gut wäre. Das heißt, eine Depression erschwert oder verhindert einen angemessenen Umgang mit dem Diabetes.

c) körperliche Wechselwirkung

Folie 3-4: ***Zwischen den beiden Erkrankungen scheint ebenfalls eine körperliche Wechselwirkung zu bestehen. Dabei geht es um eine hormonelle Interaktion. Diese organischen Prozesse sind noch nicht besonders gut erforscht. Wenn man aber bedenkt, dass man sich z.B. bei einem erhöhten Blutzucker schlapp fühlt und auch die Depression mit Erschöpfung einhergeht, kann man sich die körperlichen Bedingungsfaktoren in ihrer Wirkungsweise besser vorstellen.***

Folie 3-5: Gesamtes Bedingungsmodell zusammenfassend erklären.

Ableitung eines Behandlungskonzeptes, das sowohl die Depression als auch die Diabeteseinstellung verbessert (Rational für diese Gruppentherapie).

Z.B. ***Daher ist es uns wichtig, dass Sie einerseits die Depression verbessern, dabei aber den Diabetes nicht aus den Augen verlieren. Wir werden uns immer wieder darüber unterhalten, was Sie in Bezug auf den Diabetes machen können und wollen, welche Ziele Sie diesbezüglich haben. Wir werden Ihren Umgang mit dem Diabetes immer wieder thematisieren, Sie können Schwierigkeiten ansprechen und wir suchen nach Lösungsmöglichkeiten. Unser Ziel ist es, dass Sie nicht nur eine Verbesserung Ihrer Depression erleben, sondern, dass sich zusätzlich Ihr HbA_{1c} um mind. einen Prozentpunkt verringert***.

Zur Erinnerung: ***Man hat herausgefunden, dass sich die Senkung des HbA_{1c} um ca. einen Prozentpunkt das Risiko für Komplikationen und Folgeerkrankungen drastische reduziert.***

Der Therapeut nennt anhand Folie 2-10 die Ergebnisse der Studie zur Senkung des HbA_{1c}.

3.4 Pause (10´)

3.5 Wie beeinflussen sich Gedanken und Stimmung? (Übung: 20´)

Der Therapeut führt eine erlebnisbasierte Übung zum Erkennen des Einflusses von Gedanken auf die Gefühle durch („**Auswahlübung**“ in Anlehnung an Schelp et al.: Rational-Emotive-Therapie als Gruppentraining gegen Stress, Huber, 1997, S. 48):

Alle Teilnehmer werden gebeten, für einige Zeit die Augen zu schließen und sich ganz auf ihr inneres Erleben und Verhalten zu konzentrieren. Ihnen wird gesagt,

dass der Therapeut den Kreis der Teilnehmer abschreiten wird und ein oder zwei Teilnehmer auswählen wird, mit dem oder der er später etwas machen wird (hier kann auch angesagt werden, dass der Teilnehmer dann aufgefordert wird, etwas Persönliches aus Ihrer Biographie zu erzählen). Der Auswählvorgang wird erfolgen, indem der Therapeut dem ausgewählten Teilnehmer die Hand auf die Schulter legt. Die Teilnehmer werden gebeten während der Übung nicht zu reden und die Augen erst nach Aufforderung wieder zu öffnen. Der Therapeut geht nun ca. 1 Min. möglichst hörbar im Kreis herum. Sie legt aber erst in den letzten Sekunden einem, mehreren oder aber auch niemandem die Hand auf die Schulter. Die Teilnehmer werden aufgefordert ihre Augen wieder zu öffnen und ihre Erlebnisse zu äußern. An der Flipchart werden Gedanken, emotionale und körperliche Reaktionen gesammelt.

Ziel der Übung: Die Teilnehmer sollen erleben, dass sie sich in dieser Situation unterschiedliche Gedanken gemacht haben, die zu unterschiedlichen Gefühlen geführt haben, unabhängig von dem Ereignis, ob sie nun ausgewählt wurden oder nicht. Eventuell kann ergänzend gefragt werden, was sich abgespielt hätte, wenn der Auserwählte z.B. eine Tafel Schokolade bekommen hätte.

Anhand des an der Flipchart notierten ABC-Schemas kann ein depressions- oder diabetesbezogenes Beispiel aus dem Teilnehmerkreis besprochen werden. Falls die Teilnehmer keine persönlichen Beispiele bringen, wird auf Folie 3-6 zurückgegriffen.

Z.B. ***Sie haben gerade selbst erlebt, dass Gedanken und Gefühle zusammenhängen. Es ist schwierig, unmittelbar Gefühle zu verändern. Aber man kann über Gedanken durchaus Gefühle beeinflussen, was ich mit Ihnen gerne an einigen Beispielen besprechen möchte. Welche Gedanken gehen Ihnen öfter im Kopf herum? Welche Gedanken kennen Sie bezüglich des Diabetes?***

Den Teilnehmern soll es ermöglicht werden, eigene dysfunktionale Gedanken zu erkennen (welche Gedanken führen zu welchen Gefühlen?).

3.6 Ungünstige Gedanken verändern (20´)

Alternative, günstige Gedanken führen zu angenehmeren Gefühlen und sind damit hilfreicher. Anhand der persönlichen Beispiele oder mithilfe der Folien 3-7 und 3-8 wird der Einfluss angemessener und hilfreicher Gedanken erläutert.

Z.B. ***Was könnte nun in dieser Situation ein günstigerer Gedanke sein? Es geht nicht darum, nun alles positiv oder durch die rosa Brille zu sehen, sondern hilfreiche und angemessene Gedanken zu finden.*** Beispiel geben.

Wahrscheinlich stellen sie sich nun die Frage, wie Sie das selbst anstellen sollen, ihre Gedanken zu verändern. Wir wollen uns dies nun an Ihrem Beispiel einmal genauer anschauen.

Folie 3-9: Auf dem Flipchart wird die folgende **Übung** erarbeitet und protokolliert: Es werden nacheinander exemplarisch einzelne dysfunktionale Gedanken eines Teilnehmers identifiziert und anhand des **3-Stufen-Schemas** verändert, d.h. alternative funktionalere Gedanken erarbeitet. Dabei können Vorschläge der anderen Gruppenmitglieder miteinbezogen werden.

Der Therapeut erläutert die Wirksamkeit der kognitiven Umstrukturierung.

Z.B. ***Wahrscheinlich kommt einigen von Ihnen diese konkrete Auseinandersetzung mit einzelnen Ihrer Gedanken sehr künstlich und ungewohnt vor. Das erscheint auch zunächst einmal so. Sie werden si-***

cherlich in Kürze weniger Schwierigkeiten mit diesem Vorgehen haben. Es hat sich wissenschaftlich erwiesen, dass dieses eine therapeutische Technik ist, die äußerst erfolgreich ist.

3.7 Vorstellen der Übungsaufgaben (5´)

Folie 3-10:

1. Die Teilnehmer sollen zuhause wiederkehrende dysfunktionale Gedanken identifizieren und dazu auf dem AB 7 „3 Schritte zur Veränderung negativer Gedanken“ Alternativgedanken formulieren.

   Als Hilfestellung zur Gedankenidentifizierung dient das AB 8 „Ungünstige Einstellungen".

   Der Therapeut weist darauf hin, dass diese Technik am wirksamsten ist, je häufiger man sie anwendet.

2. Es kann bei Bedarf motivierend darauf hingewiesen werden, dass AB 1 und AB 6 bis zur 10. Sitzung weitergeführt werden, und dass das AB 4 bei anstehenden Arztbesuchen zur Hilfe genommen werden sollte.

3.8 Sitzungsbewertung (5´)

4. Sitzung

- Rückblick auf die 3. Sitzung
- Überblick über die Sitzung
- Besprechung der Übungsaufgaben
- Aufrechterhaltende Bedingungen der Depression: Hindernisse bei der Zielerreichung, Problemlösung in der Großgruppe
- Übungsaufgaben
- Sitzungsbewertung

4.1 Rückblick auf die 3. Sitzung (5´)

Es wird eine kurze Zusammenfassung der Inhalte und Ergebnisse der dritten Sitzung gegeben.

Nachfrage, ob etwas offen geblieben ist und besprochen werden müsste.

Überblick über die Themen der heutigen Sitzung Folie 4.1.

4.2 Besprechung der Übungsaufgaben (45´)

Besprechung der Erfahrungen mit den Übungsaufgaben, Beseitigung von Unklarheiten:

1. Zunächst werden die Erfahrungen mit dem **3-Stufen-Schema** (AB 7) besprochen. Bei der Besprechung ist darauf zu achten, dass der Zusammenhang von Gedanken und Gefühlen noch einmal klar wird, bzw. die positive Auswirkung alternativer Gedanken auf die Stimmung.

   Jeder Teilnehmer, der sich äußern will, sollte zu Wort kommen können.

   Sofern ein Teilnehmer zu einzelnen Gedanken keine Alternative finden konnte, können bedarfsweise in der Gruppe alternative Lösungen gesucht werden.

2. Danach soll nachgefragt werden, wie sich die Erfahrungen mit den **positiven Aktivitäten** (AB 1 /AB 6) entwickeln.

   Z.B. ***Wir haben zu Beginn unseres Programms festgestellt, dass es wichtig ist, auch immer wieder positive Aktivitäten in den Tag einzuplanen. Wie gelingt Ihnen dies inzwischen? Gibt es dabei Schwierigkeiten?***

   Besprechung dieser Erfahrungen. Größere Schwierigkeiten können im zweiten Teil der Stunde aufgegriffen werden.

3. Hier ist es sinnvoll, routinemäßig nach Problemen der Diabetestherapie zu fragen, auch unabhängig von den festgelegten Zielen. Dabei kann auf entsprechende Übungsinhalte des Programms verwiesen werden oder, falls dies erforderlich erscheint, den Teilnehmer zu ermutigen, seinen Arzt aufzusuchen.

4.3 Pause (10´)

4.4 Aufrechterhaltende Bedingungen der Depression: Wie können Sie Hindernisse bei der Umsetzung Ihrer Ziele überwinden? (45´)

Bezug zu den Zielen aus Sitzung 2 und Hindernissen, die dabei aufgetreten sind, herstellen.

Der Therapeut führt eine Problemlösung nach 6 Schritten in der **Großgruppe** für 1 bis max. 2 Patienten durch:

a) ggf. allgemein (z.B. Probleme beim Aktivitätsaufbau) und /oder

b) diabetesspezifisch (z.B. Messen, Spritzen, usw.)

Z.B. ***Jeder von Ihnen hat sich in der 2. Stunde einige Ziele vorgenommen, die er erreichen will. Manchmal gestaltet sich das ja schwieriger, als man sich das vorgestellt hat. Wie sind denn Ihre Erfahrungen bisher? Hat jemand größere Schwierigkeiten, seine Ziele aus der 2. Stunde zu erreichen? Wir haben hier die Gelegenheit, gemeinsam nach einer Lösung zu suchen.***

Der Teilnehmer stellt sein Problem kurz dar (Problemanalyse = Schritt 1). Er wird dann aufgefordert eigene Lösungsvorschläge zu generieren. Dabei wird er angeleitet, alle Lösungen (z.B. auch unrealistische) zu nennen, die ihm einfallen. Es wird betont, dass es an dieser Stelle noch keinen Sinn macht, Lösungen zu bewerten. Unter Erlaubnis des Teilnehmers werden ggf. weitere Lösungsvorschläge per Brainstorming in der Gruppe gesammelt, die wiederum nicht kommentiert oder bewertet werden. Alle Lösungsvorschläge werden am Flipchart notiert (Schritt 2). Danach kann sich der Teilnehmer zu den Vorschlägen äußern. Er soll sich einen Vorschlag heraussuchen, den er als realistisch umsetzbar bewertet (Schritt 3). Die konkrete Planung und Umsetzung wird mit dem Teilnehmer durchgesprochen (Schritt 4).

Z.B. ***Was ist der nächste Schritt? Wann werden Sie das machen?***

Die Umsetzung soll wenn möglich in der nächsten Woche stattfinden (Schritt 5). Als Abschluss wird eine Erfolgsbewertung (Schritt 6) durchgesprochen. Der Teilnehmer bekommt seine Flipchartbögen mit nach Hause. Jeder Teilnehmer kann sich zur Gruppenarbeit persönliche Notizen im Persönlichen Begleitbuch machen.

Erst im Anschluss an die Erarbeitung in der Großgruppe sollen die 6 Problemlöseschritte mithilfe der Folie 4.2 transparent gemacht und genau erläutert werden.

4.5 Vorstellen der Übungsaufgaben (15´)

Folie 4-3

Aus den erörterten Beispielen sollen sich Übungsaufgaben für jeden Teilnehmer ergeben, insofern, als sich bei jedem wahrscheinlich in Bezug auf mindestens eines der Ziele Hindernisse ergeben haben. Anhand der in der Stunde gemachten Erfahrungen sollen Hindernisse noch einmal klar definiert und beschrieben und anhand der 6 Schritte (AB 9) bearbeitet werden.

Es kann bei Bedarf motivierend darauf hingewiesen werden, dass AB 1 und AB 6 bis zur 10. Sitzung weitergeführt werden, und dass das AB 4 bei anstehenden Arztbesuchen zur Hilfe genommen werden sollte.

4.6 Sitzungsbewertung (5´)

5. Sitzung

- Überblick über die Sitzung
- Rückblick auf die 4. Sitzung
- Besprechung der Übungsaufgaben
- Aufrechterhaltende Bedingungen der Depression: Welche Gedanken können bei der Problemlösung helfen?
- Übungsaufgaben
- Sitzungsbewertung

5.1 Überblick über die heutige Sitzung (5´)

Folie 5-1

5.2 Besprechung der Übungsaufgaben (45´)

Besprechung der Erfahrungen mit den Übungsaufgaben:

1. Aktivitätenaufbau weiter auswerten.
2. Kurze Zusammenfassung der Problemlösung in der Großgruppe der letzten Sitzung und Nachfrage, ob die Teilnehmer sich äußern möchten, wie es Ihnen mit der Umsetzung ergangen ist.

   Erfahrung mit Problemlösung (AB 9) der gesamten Gruppe:

   Welche Hindernisse haben sich ergeben? Konnten Sie aus dem Weg geräumt werden? Wie? Konnte jemand dabei helfen?

Sinnvoll ist den hierauf folgenden Behandlungsschritt mit zu bedenken und diesen bei Bedarf einzuleiten.

5.3 Pause (10´)

5.4 Aufrechterhaltende Bedingungen der Depression: Welche Gedanken/Einstellungen können bei der Problemlösung helfen? (Gruppenarbeit: 50´)

Kurze Rekapitulation zum Zusammenhang von Gedanken, Gefühlen und Diabetes und Überleitung zur Gruppenarbeit. Je nachdem was für Ziele/Hindernisse in der letzten Sitzung im Vordergrund standen, sollte darauf geachtet werden, dass diabetes- und depressionsspezifische Ziele ausgewogen behandelt werden. Es ist anzunehmen, dass sich die Hindernisse zu beiden Zielsetzungen zuordnen lassen, vielleicht können beide Zielsetzungen noch einmal betont werden.

Z.B. ***Wir haben uns in der vorletzten Sitzung mit dem Zusammenhang von Gedanken und Gefühlen beschäftigt. Wir haben festgestellt, dass negative Gedanken zu negativen Gefühlen führen und wir haben gesehen, dass die Suche nach alternativen Gedanken manchmal gar nicht leicht ist. Wenn man aber alternative, positivere Gedanken hat, dann ändern sich auch die Gefühle in eine positivere Richtung. Wir wollen uns in der heutigen Sitzung noch einmal mit den Hindernissen und Schwierigkeiten bei der Umsetzung Ihrer Ziele auseinandersetzen. Das ganze aber aus einer weiteren Perspektive, der***

der Gedanken. Welche Gedanken treten bei der Umsetzung Ihrer Ziele auf?

Partnerübung: ***Zum Verständnis der nächsten Übung möchte ich mit Ihnen das Vorgehen einmal an einer Beispielsituation durchgehen. Stellen Sie sich vor, eines Ihrer Ziele sei gewesen, an einem bestimmten Tag spazieren zu gehen. Nun haben Sie es leider nicht geschafft.***

(Therapeut notiert auf der Flipchart: Situation: Wollte spazieren gehen, habe es nicht geschafft.)

Ein typischer negativer Gedanke in dieser Situation könnte so etwas gewesen sein, wie „Bringt doch alles nichts! Ich kann mich nicht aufraffen, ich habe keine Energie!“

Der Therapeut notiert diesen Gedanken auf der Flipchart.

Hat jemand von Ihnen einen alternativen Gedanken parat? Z.B.: „Wenn ich mich jetzt aufraffe, geht es mir hinterher besser!“ Ich möchte Sie bitten, zu zweit zu besprechen, welche Gedanken Ihnen Schwierigkeiten bei der Umsetzung Ihrer Ziele machen. Versuchen Sie dann genauso, wie wir es gerade gemacht haben, hilfreiche Gedanken zu finden.

Im Anschluss können Sie diese auf einer Karteikarte notieren. (wird ausgeteilt)

Wir empfehlen Ihnen, die Karteikarte als eine Erinnerungsstütze für den nächsten Versuch zu nutzen. Wenn Sie sich also wieder vorgenommen haben, spazieren zu gehen, können Sie in der Situation auf Ihre Karteikarte schauen und sich selbst motivieren mit Ihrem eigenen, hilfreichen Gedanken, ihr Ziel zu verfolgen....

Wir beschriften mal eine Karteikarte gemeinsam, damit Sie sich das besser vorstellen können:: Zu dem alternativen Gedanken aus unserem ersten Beispiel „Wenn ich mich jetzt aufraffe, geht es mir hinterher besser!“ könnte ich mir notieren „Jetzt fange ich an!“ oder „Hinterher geht es mir besser“

Der Therapeut schreibt die Gedanken auf die Karteikarte und zeigt sie, so dass jeder eine Vorstellung bekommt, wie sie aussehen und verwendet werden können.

Folie 5-2: Pro Teilnehmer stehen ca. 20 Min. zur Verfügung. Der Therapeut bietet Hilfe an, wenn dies gewünscht wird.

Im Anschluss an die Kleingruppenarbeit findet je nach Zeit eine **Besprechung in der Großgruppe** statt. Man kann ein Teilnehmerpaar, welches Schwierigkeiten bei der Gedankengenerierung und anschließenden Problemlösung hatte, bitten, das Problem und die bisher gefundenen, nicht ausreichenden alternativen Gedanken kurz vorzustellen, dann können weitere Lösungen durch die Großgruppe erarbeit werden.

5.5 Übungsaufgaben (5´)

Folie 5-3

Aus den erörterten Beispielen sollen sich Übungsaufgaben für jeden Teilnehmer ergeben, insofern, als sich bei jedem wahrscheinlich in Bezug auf mindestens eines der Ziele weitere Hindernisse sowie das Auftreten dysfunktionaler Kognitionen ergeben haben. Anhand der in der Stunde gemachten Erfahrungen sollen nun zusätzlich unter Berücksichtigung ungünstiger Gedanken die 6 Problemlösestufen bearbeitet werden. Das AB 10 enthält alle Stufen wie auch AB 9, zusätzlich sind Fragen zu Kognitionen aufgeführt.

Zur Gedankenveränderung kann unterstützend das AB 7 verwendet werden.

Es kann bei Bedarf motivierend darauf hingewiesen werden, dass AB 1 und AB 6 bis zur 10. Sitzung weitergeführt werden, und dass das AB 4 bei anstehenden Arztbesuchen zur Hilfe genommen werden sollte.

5.6 Sitzungsbewertung

6. Sitzung

- Rückblick auf die 5. Sitzung
- Überblick über die Sitzung
- Besprechung der Übungsaufgaben
- Zwischenbilanz
- Wie können Sie Kraft für die Umsetzung Ihrer Ziele tanken?
- Übungsaufgaben
- Sitzungsbewertung

6.1 Rückblick auf die 5. Sitzung (5´)

Es wird eine kurze Zusammenfassung der Inhalte und Ergebnisse der fünften Sitzung gegeben.

Nachfrage, ob etwas offen geblieben ist und besprochen werden müsste.

Überblick über die Themen der heutigen Sitzung (Folie 6-1).

6.2 Besprechung der Übungsaufgaben (15´)

Besprechung der Übungsaufgaben zur Problemlösung und zum Aktivitätenaufbau.

Bei Problemen der Diabetestherapie kann auf entsprechende Übungsinhalte des Programms verwiesen werden (in dieser Sitzung eventuell der nächste Therapiebaustein eingeleitet werden) oder, falls dies erforderlich erscheint, der Teilnehmer ermutigt werden, seinen Arzt aufzusuchen.

6.3 Zwischenbilanz (60´)

Erfahrungen der Teilnehmer bis zum jetzigen Zeitpunkt:

1. Das bereits ausgefüllte AB 5 „Ziele" hinzuziehen und **kurzfristige Ziele überprüfen**:

   Wie gut wurden die kurzfristigen Ziele erreicht? Müssen sie modifiziert werden? Wenn ja, wie? Werden ausreichend Aktivitäten geplant und ausgeführt? Wie steht es um die Ausgewogenheit von positiven Aktivitäten und Pflichten?

   Ggf. werden Ziele angepasst oder erneuert.

2. **Erfolge/Misserfolge bei der Anwendung der neu gelernten Strategien:**

   Wie gut ist es Ihnen gelungen, Ihre Denkgewohnheiten zu verändern? Was fällt Ihnen schwer, bzw. was gelingt nicht?

   Der Therapeut geht flexibel auf die Beiträge der Teilnehmer ein, dabei werden sowohl depressions- und diabetesbezogene Erfolge und Misserfolge berücksichtigt. Bei der Bewertung der diabetesbezogenen Ziele ist es sinnvoll, die Empfehlungen des Arztes (AB 4) hinzu zu ziehen. Ggf. sollten die Teilnehmer ermutigt werden, erneute Rücksprache mit ihrem Arzt mithilfe des AB 4 zu halten.

   Bei interaktionellen Aspekten wird auf die nächste Sitzung hingewiesen.

6.4 Pause (10´)

6.5 Wie können Sie Kraft für die Umsetzung Ihrer Ziele tanken? (20´)

Einzelarbeit:

Sie haben bisher versucht, sich Ziele zu setzen und diese auch zu verfolgen. Dabei kommen häufig persönliche Bedürfnisse, die mit Genuss zu tun haben, zu kurz. Das

zu tun, wonach einem gerade ist, gönnt man sich manchmal gar nicht, oder man kann es gar nicht genießen, weil einen das schlechte Gewissen plagt. Die Verfolgung Ihrer Ziele bedeutet harte Arbeit, umso wichtiger ist ein Ausgleich... Was können Sie tun, um aufzutanken und Kraft zur Umsetzung Ihrer Ziele zu bekommen?

Folie 6-2:

Suchen Sie sich einen ruhigen Ort und notieren sie sich, wie Sie in der nächsten Woche Kraft tanken möchten. (AB 11)

6.6 Hinweis auf die 9. Sitzung

Der Therapeut macht darauf aufmerksam, dass für die 9. Sitzung ein Schwerpunktthema gewählt werden wird. Es können dann Techniken wiederholt, Schwierigkeiten besprochen, etc. werden.

6.7 Übungsaufgaben (5´)

Folie 6-3:

Die Teilnehmer werden motiviert, in der nächsten Woche mithilfe Ihrer Notizen Kraft zu tanken.

Es kann bei Bedarf motivierend darauf hingewiesen werden, dass AB 1 und AB 5 bis zur 10. Sitzung weitergeführt werden, und dass das AB 4 bei anstehenden Arztbesuchen zur Hilfe genommen werden sollte.

6.8 Sitzungsbewertung (5´)

7. Sitzung

- Rückblick auf die 6. Sitzung
- Überblick über die Sitzung
- Besprechung der Übungsaufgaben
- Schwierige Situationen im Umgang mit anderen Menschen: Bedürfnisse umsetzen
- Übungsaufgaben
- Sitzungsbewertung

7.1 Rückblick auf die 6. Sitzung (5´)

Es wird eine kurze Zusammenfassung der Inhalte und Ergebnisse der sechsten Sitzung gegeben.

Nachfrage, ob etwas offen geblieben ist und besprochen werden müsste.

Überblick über die Themen der heutigen Sitzung Folie 7-1.

7.2 Besprechung der Übungsaufgaben (20´)

1. Besprechen der Protokolle positiver Aktivitäten (AB 6/AB 3) mit dem Ziel, einen Vergleich zum Beginn des Programms heraus zu arbeiten.
2. Besprechen, ob und wie die Teilnehmer Kraft getankt haben (AB 11).
3. Hier ist es sinnvoll, routinemäßig nach Problemen der Diabetestherapie zu fragen - unabhängig von den festgelegten Zielen. Dabei kann auf entsprechende Übungsinhalte des Programms verwiesen werden oder, falls dies erforderlich erscheint, der Teilnehmer ermutigt werden, seinen Arzt aufzusuchen.

7.3 Schwierige Situationen im Umgang mit anderen Menschen: Bedürfnisse umsetzen (Übungen zur sozialen Kompetenz oder Gruppenberatung: 55´)

1. Es können einige oder alle Teilnehmer Bedürfnisse nennen, deren Umsetzung schwierig ist, da soziale Kommunikation erforderlich ist, oder das eigene Bedürfnis sogar mit dem von anderen kollidiert. („Bedürfnis“ wird hier als Sammelbegriff für Forderung, Wunsch, Recht, etc. verwendet. Es wird nicht zwischen Situationstypen (wie in Hintsch & Pfingsten, 2002) unterschieden. In dieser Übung werden nur solche Bedürfnisse thematisiert, die die Teilnehmer gerne umsetzen möchten, es aber aufgrund mangelnder sozialer Kompetenz nicht können.).

   Die Situationen sollen wenn möglich im Zusammenhang mit der Diabeteserkrankung stehen. Sie werden zusammengetragen und auf dem Flipchart notiert.

   Sofern keine Beispielsituationen von den Teilnehmern genannt werden, kann die Folie 7-2 mit diabetesbezogenen Beispielsituationen gezeigt und nach Erfahrungen der Teilnehmer in solchen Situationen gefragt werden.
2. Anhand einer Beispielsituation sollen an der Flipchart **Kriterien für selbstsicheres Äußern des Bedürfnisses**

gesammelt werden. Folgende Fragen sollen dazu an die Flipchart geschrieben und von den Teilnehmern beantwortet werden:

Was sollte er/sie beim Äußern dieses Bedürfnisses beachten?

Was sollte er/sie vermeiden?

Falls keine oder wenige Vorschläge kommen, kann an dieser Stelle auf die komplexere Tabelle im Persönlichen Begleitbuch (S. 20) zurückgegriffen werden.

3. Eine Beispielsequenz wird im **Rollenspiel** unter der Berücksichtigung der aufgestellten Kriterien geübt. Nach Möglichkeit sollen die Rollenspiele nicht als solche angekündigt werden, um die Teilnehmer nicht zu verschrecken, sondern die Rollenspiele sollen ganz unverbindlich eingeleitet werden.

   Wenn z.B. eine Teilnehmerin erzählt, kann der Leiter zur Konkretisierung auffordern: ***Herr/Frau XY, erzählen Sie doch einmal, wie man sich Ihre angesprochene Situation vorstellen kann. Vielleicht erinnern Sie sich an das letzte Mal, als es Ihnen so ergangen ist.***

   Der Therapeut übernimmt dabei einfach eine Rolle in der geschilderten Episode. Sie bleibt in allen Rollenspielen Interaktionspartner und stellt sich je nach Teilnehmer auf den Schweregrad der Übung ein.

   Nach dem Rollenspiel leitet der Therapeut ein **Feedback** an, indem

   a) zunächst der Bedürfniseinforderer zu seinem Erleben befragt wird.

   Wie haben Sie sich dabei gefühlt? Hat Ihr Gegenüber verstanden, worum es Ihnen ging?

   a) Dann wird der Teilnehmer gefragt, ob er Rückmeldung aus der Gruppe bekommen möchte. Wenn ja, wird zunächst gefragt, was dem Rollenspieler gut gelungen ist.

   b) Als zweites können Verbesserungsvorschläge gemacht werden.

   Der Therapeut beachtet dementsprechend, dass das Feedback ressourcenorientiert gehalten wird.

Als Alternative in einer Gruppe oder bei einem Patienten, der sich nicht auf das aktive Rollenspiel einlässt, wird eine **Gruppenberatung** nach Fiedler (2005) durchgeführt.

Zunächst werden die „Spielregeln“ der Gruppenberatung erläutert.

Ein Teilnehmer benennt eine schwierige soziale Situation aus den o.g. Bereichen und rückt damit in die „Einzeltherapie in der Gruppe“. Unter wiederholter Absicherung des Therapeuten, dass der Teilnehmer sich beraten lassen möchte bzw. die Beratung weiter fortgeführt werden soll, stellen ihm die anderen Teilnehmer so viele Fragen zur Situation, bis alle glauben, die Schwierigkeit und deren Begleitumstände verstanden zu haben.

In einer zweiten Runde geben die anderen Teilnehmer Ratschläge und Lösungsvorschläge, die von dem Betroffenen ohne Bewertung angehört und notiert werden. Auch hier sichert sich der Therapeut zwischendurch ab, ob die Beratungssituation für den entsprechenden noch angenehm ist, ob er weitermachen möchte.

Z.B.: ***Möchten Sie sich noch einen Ratschlag anhören? Möchten Sie das so stehen lassen oder möchten Sie noch einen Ratschlag?***

Wenn der Teilnehmer es wünscht, gibt der Therapeut ebenfalls eine Rückmeldung. Dabei ist darauf zu achten, dass diese die Beiträge der anderen Teilnehmer nicht entwertet.

Zum Abschluss ist ein Statement des Beratenen möglich.

Die **Pause** von 10 min soll an geeigneter Stelle eingeschoben werden, danach werden die Rollenspiele bzw. die Gruppenberatung weitergeführt.

7.4 Übungsaufgaben

Folie 7-3:

Zu Hause werden zwei Bedürfnisse, die mit denen anderer kollidieren, herausgesucht und das in den Rollenspielen Gelernte in der Realität umgesetzt. Die Erfahrungen, insbesondere das als hilfreich Erlebte, sollen auf dem AB 12 protokolliert werden.

Es kann bei Bedarf motivierend darauf hingewiesen werden, dass AB 1 und AB 6 bis zur 10. Sitzung weitergeführt werden, und dass das AB 4 bei anstehenden Arztbesuchen zur Hilfe genommen werden sollte.

7.5 Sitzungsbewertung (5´)

8. Sitzung

- Rückblick auf die 7. Sitzung
- Überblick über die Sitzung
- Besprechung der Übungsaufgaben
- Wie beeinflussen sich Ängste vor Folgeerkrankungen, Stimmung und der Umgang mit dem Diabetes?
  a) Ängste vor Diabetesfolgen
  b) Umgang mit Ängsten
- Reduktion der Ängste
- Festlegung des Themas für die 9. Sitzung
- Übungsaufgaben
- Sitzungsbewertung

8.1 Rückblick auf die 7. Sitzung (5´)

Es wird eine kurze Zusammenfassung der Inhalte und Ergebnisse der siebten Sitzung gegeben.

Nachfrage, ob etwas offen geblieben ist und besprochen werden müsste.

Überblick über die Themen der heutigen Sitzung (Folie 8-1).

8.2 Besprechung der Übungsaufgaben (15´)

Besprechung der Realisierung von Bedürfnissen. Welche Schritte wurden mit welchem Erfolg umgesetzt? (AB 12). Sofern jemand ein Scheitern berichtet, können Alternativen in einem Rollenspiel geübt werden.

Hier ist es sinnvoll, routinemäßig nach Problemen der Diabetestherapie zu fragen, auch unabhängig von den festgelegten Zielen. Dabei kann auf entsprechende Übungsinhalte des Programms verwiesen werden oder, falls dies erforderlich erscheint, der Teilnehmer ermutigt werden, seinen Arzt aufzusuchen.

8.3 Wie beeinflussen sich Ängste vor Folgeerkrankungen, Stimmung und der Umgang mit dem Diabetes?

a) Ängste vor Diabetesfolgen (20´)

Folie 8-2:

In der **Großgruppe** wird erarbeitet, ob Ängste bzgl. Folgeerkrankungen bestehen und wie die Teilnehmer mit diesen umgehen.

Sammeln der Antworten auf dem Flipchart.

b) Umgang mit Ängsten bzgl. der diabetischen Folgeerkrankungen (Psychoedukation: 15´)

Ziel der Psychoedukation soll sein, Ängste einerseits als sinnvoll und zum Leben gehörig anzuerkennen, aber auch die Grenzen zur pathologischen Angst zu erkennen. Selbstverständlich ist das Risiko für bestimmte Erkrankungen bei Diabetes erhöht, damit sollte auch eine "gesunde" Angst einhergehen, die zu Vorsichtsmaßnahmen führt, z.B. dem Achten auf eine gute Stoffwechseleinstellung. Eine zu große Angst, die dysfunktionale Konsequenzen hat (z.B. große Angst, welche die Lebensqualität deutlich beeinträchtigt oder die zu extensiven Vorsorgemaßnahmen führt) sollte abgebaut werden. Dazu müs-

sen häufig angewandte Vermeidungsstrategien ("am besten nicht darüber nachdenken", zu viel sorgen) aufgegeben werden. Eine Konfrontation mit den angstmachenden Gedanken und deren Veränderung sind daher wesentlich, um übertriebene Ängste zu reduzieren.

Die Psychoedukation sollte möglichst interaktiv gestaltet werden, indem der Bezug zum Erleben der Teilnehmer hergestellt wird und persönliche Beispiele aufgenommen werden!

Z.B. ***Kennt jemand von Ihnen einen Menschen, der noch nie in seinem Leben Angst gehabt hat? Wohl nicht, denn Angst gehört zum Leben. Schon ganz kleine Kinder haben Angst vor bestimmten Dingen, z.B. vor Höhe. Man kann sogar sagen, dass es vorteilhaft ist, Angst empfinden zu können. Stellen Sie sich vor, Sie hätten absolut keine Angst vor Höhe: wahrscheinlich würden Sie ziemlich leichtsinnig auf Berge klettern und auf Fenstersimsen herumturnen, denn Sie hätten ja keine Angst hinunterzufallen. Die Wahrscheinlichkeit, dass Sie dann tatsächlich hinunterfallen, ist dann natürlich relativ groß. Angst führt dazu, dass Sie vorsichtig sind und sich in Acht nehmen und helfen letztlich dabei zu überleben. Ähnlich ist es mit der Angst vor Feuer. Angst ist also eigentlich ein sinnvolles Gefühl. Aber Angst kann auch so groß werden, dass sie Sie von Dingen abhalten kann, die Sie eigentlich tun wollen oder sollen. Man kann solche Angst vor dem Zahnarzt entwickeln, dass man da nicht hingeht und am Ende nur noch kaputte Zähne hat, die Zahnschmerzen verursachen. Oder man kann so große Ängste vor Menschen entwickeln, dass man nicht mehr einkaufen geht. Solche Ängste sind in den allermeisten Fällen nicht angemessen und beeinträchtigen das Leben und die Lebensqualität immens.***

Diabetes ist eine Erkrankung mit einem erhöhten Risiko für Folgeerkrankungen, welche die Augen, Nieren und Nerven in Mitleidenschaft ziehen. Ängste in Bezug auf diese Folgeerkrankungen sind nicht unrealistisch, die Risiken sind ja nicht wegzudiskutieren. Dass sorgenvolle Gedanken immer einmal wieder auftreten, z.B. wenn der HbA_{1c} einmal ziemlich schlecht war oder erste Anzeichen für Folgeerkrankungen gefunden werden, ist sicherlich normal.

Wenn aber die Ängste so groß werden, dass Sie Ihre Lebensqualität oder Ihre Stoffwechseleinstellung massiv im negativen Sinn beeinflussen, dann wird das selbst zu einem Problem. Auf der anderen Seite ist es auch keine Lösung, den Gedanken an Folgeerkrankungen und die Befürchtungen bei Seite zu schieben und einfach nicht mehr daran zu denken.

Folie 8-3:

Es muss also eine Balance, ein Gleichgewicht zwischen Angst und Sorglosigkeit ***gefunden werden. Was also tun?***

Folie 8-4:

1. *Nicht weiter drüber nachdenken, sich ablenken.* ***Wer von Ihnen kennt das? Was ist die Folge davon? Oft genug lassen sich solche Gedanken nicht verscheuchen oder kommen ziemlich schnell wieder. Und jedes mal verursachen sie Angst. Wenn Sie versuchen so zu tun, als gäbe es die Risiken dieser Komplikationen nicht und den Gedanken immer beiseite schieben, bedeutet das aber, dass Sie die Realität verleugnen. Oft bedeutet das auch, dass man mögliche Vorsorgestrategien nicht anwendet.***

2. *Oder Sie machen sich so oft wie möglich Gedanken darüber und sorgen sich die ganze Zeit.* ***Gibt es***

jemanden von Ihnen, der sich eher zu viel sorgt? Damit kommt man schnell zu der Überzeugung, dass es sowieso so kommen wird und dass alles, was Sie tun, sowieso nichts daran ändern kann. Oder Sie sind dermaßen angespannt, dass sie sich auf nichts anderes mehr konzentrieren können. Auch hier werden Sie möglicherweise keine Vorsorge treffen, da sie dem Schicksal eh nicht entrinnen können.

3. *Oder Sie denken diese Gedanken einmal zu Ende.* ***Beantworten Sie sich die Frage, wie realistisch der Gedanke und die Angst sind. Sie können versuchen, eine Lösung dafür zu finden, wenn der Gedanke einmal Realität werden sollte. Wenn Sie eine Lösung haben, verliert der Gedanke viel von seiner Bedrohung und sie haben weniger Angst. Ein hilfreicher Gedanke ist: Wenn es soweit kommen sollte, weiß ich, was ich tun kann.***

8.4 Pause (10´)

8.5 Bewältigung der Ängste bzgl. der Folgeerkrankungen (40´)

1. **Gruppenarbeit**: Die Gesamtgruppe wird in zwei Teilgruppen geteilt.

   Folie 8-5: Zunächst soll jede Gruppe so viele typische Angstgedanken bzgl. der Folgeerkrankungen wie möglich generieren (5 Min.). Im Anschluss sollen alternative, konstruktive Gedanken formuliert werden (10 Min.). Der Gruppenleiter kann insbesondere in der "konstruktiven Phase" die Gruppen unterstützen.

2. Anhand einiger Beispielsituationen werden **Strategien zur Angstreduktion** an der Flip-Chart **vermittelt** (s. Begleitbuch): Realitätsüberprüfung, Entkatastrophisieren, Zu-Ende-Denken der Vorstellungen, 3-Stufen-Schema.

   Beispiel:
   Es wird auf eine Angstsituation eines Teilnehmers eingegangen und dazu ein katastrophisierender Gedanke an der Flip-Chart notiert, gemeinsam können Argumente für und gegen den Gedanken gesammelt und notiert werden.

8.6 Festlegung des Themas für die nächste Stunde (5´)

Es können Themen und Techniken wiederholt werden. Falls ein spezielles Thema (z.B. Beantragung eines Schwerbehindertenausweises) gewünscht wird, gibt es vielleicht Freiwillige, die dazu Informationen einholen und die Sitzung mit vorbereiten.

8.7 Übungsaufgaben (5´)

Folie 8-6:

Erinnern Sie sich an eine Situation, in der das Gleichgewicht zwischen Angst und Sorglosigkeit bei Ihnen nicht optimal war. Notieren Sie sich drei Gedanken, die in dieser Situation aufgetreten sind. Bearbeiten Sie diese anhand des Arbeitsblattes „3-Stufen-Schema“ (AB 7).

Es kann bei Bedarf motivierend darauf hingewiesen werden, dass AB 1 und AB 6 bis zur 10. Sitzung weitergeführt werden, und dass das AB 4 bei anstehenden Arztbesuchen zur Hilfe genommen werden sollte.

8.8 Sitzungsbewertung (5´)

9. Sitzung

- Rückblick auf die 8. Sitzung
- Überblick über die Sitzung
- Besprechung der Übungsaufgaben
- Freies Thema
- Übungsaufgaben
- Sitzungsbewertung

9.1 Rückblick auf die 8. Sitzung (5´)

Es wird eine kurze Zusammenfassung der Inhalte und Ergebnisse der achten Sitzung gegeben.

Nachfrage, ob etwas offen geblieben ist und besprochen werden müsste.

Überblick über die Themen der heutigen Sitzung.

9.2 Besprechung der Übungsaufgaben (20´)

Besprechung der Aufgaben zur Angstreduktion vor Folgeerkrankungen.

Besprechung von Schwierigkeiten, ggf. kurze Gruppenberatung, sofern ein Teilnehmer keine Änderung herbeiführen konnte.

Hier ist es sinnvoll, routinemäßig nach Problemen der Diabetestherapie zu fragen, auch unabhängig von den festgelegten Zielen. Dabei kann auf entsprechende Übungsinhalte des Programms verwiesen werden oder, falls dies erforderlich erscheint, der Teilnehmer ermutigt werden, seinen Arzt aufzusuchen.

9.3 Leerer Block: Bearbeitung des in der letzten Stunde festgelegten Themas nach Wunsch der Teilnehmer (70´)

9.4 Pause an geeigneter Stelle (10´)

9.5 Übungsaufgaben (10´)

Folie 9-1:

Die Teilnehmer bewerten das Gruppenprogramm, indem sie ihre drei wichtigsten Erkenntnisse auf dem AB 13 auflisten.

Es kann bei Bedarf motivierend darauf hingewiesen werden, dass AB 1 und AB 6 bis zur 10. Sitzung weitergeführt werden, und dass das AB 4 bei anstehenden Arztbesuchen zur Hilfe genommen werden sollte.

9.6 Sitzungsbewertung (5´)

10. Sitzung

- Rückblick auf die 9. Sitzung
- Überblick über die Sitzung
- Besprechung der Übungsaufgaben
- Wie können Sie mit Rückschritten positiv umgehen und Ihre Erfolge aufrechterhalten?
- Was ist ein Rückschritt für mich? Was möchte ich dem aufgetretenen Rückschritt entgegensetzen?
- Weitere Arbeit mit dem Persönlichen Begleitbuch
- Verabschiedung
- Sitzungs- und Gesamtbewertung

10.1 Rückblick auf die 9. Sitzung (5´)

Es wird eine kurze Zusammenfassung der Inhalte und Ergebnisse der neunten Sitzung gegeben.

Nachfrage, ob etwas offen geblieben ist und besprochen werden müsste.

Überblick über die Themen der heutigen Sitzung (Folie 10-1)

10.2 Besprechung der Übungsaufgaben (20´)

Die Teilnehmer berichten über die Themen, die bisher für sie am wichtigsten oder hilfreichsten waren (AB 13).

Es wird wiederholt AB 1, AB 6 (und evtl. AB 3) besprochen. Hier kann auf Veränderungen im Verlauf des Programms geschaut werden.

Hier ist es sinnvoll, routinemäßig nach Problemen der Diabetestherapie zu fragen, auch unabhängig von den festgelegten Zielen. Dabei kann auf entsprechende Übungsinhalte des Programms verwiesen werden oder, falls dies erforderlich erscheint, der Teilnehmer ermutigt werden, seinen Arzt aufzusuchen.

10.3 Wie können Sie mit Rückschritten positiv umgehen und Ihre Erfolge aufrechterhalten? (Psychoedukation: 10´)

Es wird die Wichtigkeit von Rückfallprophylaxe dargestellt. Hinweis, dass Rückschritte normal sind und kein Zeichen des Versagens darstellen. Es wird auf die Notwendigkeit der Analyse eines geschehenen Rückschrittes hingewiesen.

Z.B. Rückschritte können immer wieder auftauchen. Jedes Ziel, das wir erreichen wollen ist mit Schwierigkeiten verbunden. Seine Depression dauerhaft zu verbessern und den Stoffwechsel langfristig auf einem angemessenen Niveau zu halten, heißt ebenfalls Schwankungen in Kauf zu nehmen. Gerade stressvolle Zeiten, die natürlicherweise vorkommen, begünstigen eine depressive Stimmung. Dennoch ist ein Rückschritt noch kein Rückfall in die Depression. Ein Anstieg des HbA_{1c} muss auch keine dauerhafte Verschlechterung des Stoffwechsels bedeuten. Die Art und Weise wie wir mit Rückschritten umgehen, hat eine große Bedeutung für unser anschließendes Wohlbefinden und den weiteren Verlauf einer Erkrankung. Das Erkennen erster Rückschritte und entsprechendes Gegenwirken kann einen Rückfall verhindern. Es ist an jeder Stelle möglich, die depressive Spirale zu unterbrechen. Es lohnt sich auch immer wieder zu versuchen, den HbA_{1c} konstant

auf einem angemessenen Niveau zu halten. Lassen Sie sich nicht entmutigen!

Wichtig ist

1. ***nach Ursachen für geschehene Rückschritte zu suchen. Wir empfehlen die Analyse eines Rückschrittes mit folgenden Fragen (Folie 10-2):***

   War der Rückschritt vermeidbar?

   Haben Sie sich vielleicht ein zu hohes Ziel gesteckt?

   Haben Sie vielleicht Faktoren, die Sie nicht beeinflussen können, unterschätzt?

   Wäre es vielleicht hilfreich, sich zusätzliche Hilfe von Freunden/ Partnern einzuholen?

   Im Falle eines Rückschrittes können diese Fragen im Persönlichen Begleitbuch nachgelesen werden!

2. ***Wichtig ist allerdings auch, sich genau zu überlegen, wann man überhaupt von einem Rückschritt spricht und was dann weiterhin zu tun ist. Bei einem HbA_{1c} von 7,8% achtsam zu sein und die Selbstkontrolle wieder etwas zu verstärkten ist einfacher, als abzuwarten und später dann einen HbA_{1c} von 9% wieder herunter auf 7,5% zu bekommen. Bereits bei beginnender depressiver Stimmung, fühlt man sich meist antriebsloser und unsicherer.***

   Am besten ist es also, sich schon jetzt auf einen möglichen Rückschritt vorzubereiten, so dass es Ihnen dann in solch einer Situation leichter fällt, Ihre Ziele wieder aufzunehmen.

10.4 Was ist ein Rückschritt für mich? Was möchte ich aufgetretenen Rückschritten entgegen setzen? (Partnerübung: 40´)

Folie 10-3: In Zweiergruppen sollen die Teilnehmer sammeln:

1. **woran jeder merkt, dass er sich von seinem Ziel entfernt hat, einen Rückschritt macht.**

   Jeder Teilnehmer trägt die Definition seiner möglichen Rückschritte in sein Begleitbuch (S. 39 und 40) ein. Dabei sollen sowohl depressionsbezogene wie auch diabetesbezogene Rückschritte formuliert werden (z.B. HbA_{1c} überschreitet einen festgelegten Wert, Rückzugsverhalten, den wöchentlichen Sport vermeiden u.ä.).

   Der Therapeut weist darauf hin, dass die Rückschritte **konkret und klar** beschrieben werden (nicht: "wenn es mir schlecht geht" oder "wenn der HbA_{1c} schlechter wird" oder "wenn ich mich weniger bewege") und erinnert dabei an die Regeln zur Zielformulierung, die auch hier sinnvoll sind.

2. **was jeder Teilnehmer bei Bemerken dieser Rückschritte machen möchte.**

   Hier sollen im Gruppenprogramm vermittelte Strategien gefunden werden, die bei einem Rückschritt helfen können, sein Ziel weiter zu verfolgen, und die Rückentwicklung somit frühzeitig aufhalten zu können.

Pro Partner stehen ca. 15 Min. zur Verfügung.

Anschließend findet eine **Nachbesprechung** in der Großgruppe statt.

Jeder Teilnehmer soll am Ende der Übung wissen, was für ihn ein Rückschritt ist (sowohl bzgl. der Depression als auch des Diabetes) und eine Idee haben, was er

dem entgegnen möchte. Falls Teilnehmer nicht fertig geworden sind, werden sie motiviert Ihre Eintragungen zuhause zu vervollständigen.

10.5 Pause (10´)

10.6 Weitere Arbeit mit dem Begleitbuch und das persönliche Gespräch in einem Jahr (15´)

Die Teilnehmer werden motiviert, depressions- und diabetesbezogene **Ziele hinsichtlich eines Jahreszeitraumes** zu generieren und zuhause ins Persönliche Begleitbuch (S. 42) einzutragen. Die Zielerreichung wird dann in der Jahreskatamnese mit einem Studienmitarbeiter im Einzelkontakt besprochen. Dieses Gespräch soll als Motivationsstütze dienen.

Die Teilnehmer werden darüber hinaus ermutigt, die zusammengetragenen Arbeitsblätter und Notizen nicht nur zur Rückfallprophylaxe, sondern auch zur weiteren Zielverfolgung und zum Generieren neuer Ziele heran zu ziehen.

An dieser Stelle wird noch einmal betont, dass ein grundsätzlicher Bestandteil dieses Programms die optimierte Zusammenarbeit mit dem Arzt hinsichtlich der Senkung des Hba_{1c} ist. Diese optimale Zusammenarbeit kann über das Fortsetzen der regelmäßigen Arztgespräche über mögliche Verhaltensweisen zur Verbesserung der Stoffwechseleinstellung gewährleistet werden (AB 4).

Es wird auf die hohe Wirksamkeit von Bibliotherapie hingewiesen und motiviert die erlernten Techniken mithilfe des Persönlichen Begleitbuches weiter zu üben.

Z.B. ...es ist sinnvoll, sich vielleicht einmal im Monat die Ziele, die man sich vorgenommen hat, zu überprüfen. Schauen Sie regelmäßig, wie weit Sie sie umgesetzt haben. Wenn man dies zu bestimmten Zeitpunkten macht, kann man verhindern, dass man sie aus den Augen verliert. Bei Erreichen der Ziele kann man sich analog der Aufzeichnungen neue Ziele setzen. Ebenso kann man mit der Überprüfung von Denkmustern verfahren. Auch hier empfehlen wir, die Techniken, wie das 3-Stufen-Schema zur Veränderung unangemessener Gedanken weiter zuüben, da sich alte Gewohnheiten schnell einschleichen können. Das Persönliche Begleitbuch können Sie also als einen ständigen Begleiter nutzen, den Sie immer wieder als Hilfestellung heranziehen können...

…Nehmen Sie zu Ihren Arztterminen das Arbeitsblatt 4 mit, so können Sie mit Ihrem Arzt über weitere mögliche Schritte zur Verbesserung Ihrer Stoffwechseleinstellung sprechen…

10.7 Verabschiedung (10´)

10.8 Sitzungs- und Gesamtbewertung (10´)

Jeder Teilnehmer, der will, gibt kurz ein Feedback zum Programm.

Was hat besonders gefallen/geholfen, was eher nicht?

Ausfüllen des Rückmeldebogens.

Literaturverzeichnis

Anderson, R.J., Freedland, K.E., Clouse, R.E. & Lustman, P.J. (2001). The Prevalence of Comorbid Depression in Adults With Diabetes: A meta-analysis. ***Diabetes Care***, 24, 1069-1078.

Fiedler, P. (1996). ***Verhaltenstherapie in und mit Gruppen. Psychologische Psychotherapie in der Praxis.*** Weinheim: Psychologie Verlags Union.

Grawe, K., Donati, R. & Bernauer, F. (1994) ***Psychotherapie im Wandel - Von der Konfession zur Profession***. Göttingen: Hogrefe-Verlag.

Hautzinger, M. (1997). ***Kognitive Verhaltenstherapie bei Depressionen***. Weinheim: Psychologie Verlags Union.

Jacobi, F., Hoyer, J. & Wittchen, H.-U. (2004). Seelische Gesundheit in Ost- und Westdeutschland. ***Zeitschrift für Klinische Psychologie und Psychotherapie, 33,*** 251-260.

Hintsch, R. & Pfingsten, U. (1983***). Gruppentraining sozialer Kompetenzen***. Weinheim: Psychologie Verlags Union.

Kühner, C. & Weber, I. (2001). ***Depression vorbeugen. Ein Gruppenprogramm nach R.F. Muňos***. Göttingen: Hogrefe-Verlag.

Liebermann, M.A., Yalom, I.D. & Miles, M.B. (1973). ***Encounter groups: First facts***. New York: Basic Books.

Schelp, T., Maluck, D. & Gravemeier, R. (1997). ***Rational-Emotive Therapie als Gruppentraining gegen Stress***. Bern: Hans Huber Verlag.

UK Prospective Diabetes Study, UKPDS (1998). ***The Lancet***, 352, 837-858.
