# Supplementary material for: Study protocol of the Diabetes and Depression Study (DAD): a multi-center randomized controlled trial to compare the efficacy of a diabetes-specific cognitive behavioral group therapy versus sertraline in patients with major depression and poorly controlled diabetes mellitus
Source: BMC Psychiatry. 2013 Aug 6;13:206. doi: 10.1186/1471-244X-13-206 (PMC3750698; doi:10.1186/1471-244X-13-206)
Supplement: Additional file 3 — Working sheet for diabetologists and patients: strategies to improve glycemic control’ (in German). [file 1471-244X-13-206-S3.pdf]

Diabetes-Depressions-Studie

Vorbereitung für die Verhaltenstherapie

Liebe Patientin, lieber Patient,

Sie werden nun bald im Rahmen der DAD-Studie an der Verhaltenstherapie teilnehmen. In jeder Gruppensitzung werden unter anderem auch Themen bearbeitet, die mit dem Diabetes zusammenhängen und auch mindestens eines Ihrer Therapieziele sollte direkt auf den Diabetes bezogen sein. Im Rahmen der Verhaltenstherapie wird immer wieder auf diese Ziele eingegangen und mögliche Schwierigkeiten bei der praktischen Umsetzung können besprochen werden.

Welche Ziele werden in der DAD-Studie angestrebt?

- Als kurzfristiges Ziel der Verhaltenstherapie streben wir zunächst eine Verbesserung Ihrer Depression und eine Verbesserung Ihrer Lebensqualität an.
- Als langfristiges Ziel möchten wir Ihnen zusätzlich helfen, die Qualität Ihrer Blutzuckereinstellung zu verbessern, um einen besseren Verlauf Ihres Diabetes wahrscheinlicher zu machen. Konkret heißt das, dass wir eine langfristige Verbesserung Ihres $HbA1_c$-Wertes um mindestens 1%-Punkt erreichen möchten.

Wie können Sie mit Ihrem Arzt zusammenarbeiten, um diese Ziele zu erreichen?

Um Ihnen zu helfen, Ihren Umgang mit dem Diabetes möglichst so zu gestalten, dass Ihre Blutzuckerwerte besser werden, möchten wir Sie bitten, sich an Ihren Arzt, der den Diabetes behandelt, zu wenden. Er sollte ein bis drei konkrete Verhaltensweisen mit Ihnen besprechen, die aus seiner Sicht für Sie persönlich entscheidend wären, um eine Verbesserung Ihrer Stoffwechseleinstellung zu erreichen.

Diese Verhaltensweisen sollten auf dem beiliegenden Arbeitsblatt eingetragen werden.

Beispiel für eine konkrete Verhaltensweise: Wenn nach der Blutzuckermessung die Blutzuckerwerte deutlich zu hoch waren, sollten Sie die passende Menge an Insulin zur Korrektur spritzen.

Durch dieses Vorgehen erhalten nicht nur Sie konkrete Empfehlungen, sondern auch Ihre Verhaltenstherapeutin bekommt eine Möglichkeit, die Empfehlungen Ihres Prüfarztes zu berücksichtigen.

Bitte besprechen Sie zu Beginn der Therapie und bei jeder Untersuchung bei Ihrem Arzt diese konkreten diabetesbezogenen Verhaltensweisen sowie die Möglichkeiten zu deren Umsetzung (möglicherweise ändern sich auch im Laufe der Zeit die Empfehlungen).

Protokollieren Sie das Ergebnis dieser Besprechungen bitte in Ihrem Persönlichen Begleitbuch zum Gruppenprogramm, welches Sie in der ersten Gruppensitzung erhalten. Es ist sinnvoll dies auch nach Beendigung der Verhaltenstherapie weiter fortzuführen, mindestens bis zum Zeitpunkt der Abschlussuntersuchung ein Jahr nach Beendigung der Verhaltenstherapie. Dies kann Ihnen helfen, auch langfristig Ihre diabetesbezogenen Therapieziele nicht aus den Augen zu verlieren.

ARBEITSBLATT
EMPFEHLUNGEN ZUR SENKUNG DES HbA_{1C}

Patient:________________________ **Datum:** _____________ **ggf. Visit:**____

Vom Arzt auszufüllen:

Folgende Verhaltensweisen könnten Ihnen helfen, Ihre Stoffwechseleinstellung langfristig zu verbessern (= $HbA1_c$ um mindestens 1% zu senken), und dadurch die Wahrscheinlichkeit von Spätschäden durch den Diabetes zu verringern:

A) ____

B) ____

C) ____

Vom Patienten auszufüllen:

Bitte tragen Sie in die Kästchen für jede Empfehlung (A bis C) ihre persönliche Bewertung auf einer Skala von 0-10 ein.
0 = gar nicht wichtig/schwierig bis 10 = maximal wichtig/schwierig

	A	B	C
Wie wichtig ist **mir persönlich** die Umsetzung dieser Empfehlung?			
Wie schwierig erscheint mir die Umsetzung dieser Empfehlung?			

Folgende **Lösungsmöglichkeiten** zur Umsetzung der Empfehlungen habe **ich mit meinem Arzt** besprochen:

Zu A) ____

Zu B) ____

Zu C) ____
