# Supplementary material for: Study protocol of the Diabetes and Depression Study (DAD): a multi-center randomized controlled trial to compare the efficacy of a diabetes-specific cognitive behavioral group therapy versus sertraline in patients with major depression and poorly controlled diabetes mellitus
Source: BMC Psychiatry. 2013 Aug 6;13:206. doi: 10.1186/1471-244X-13-206 (PMC3750698; doi:10.1186/1471-244X-13-206)
Supplement: Additional file 4 — Working book for patients of the diabetes-specific CBT group (in German). [file 1471-244X-13-206-S4.pdf]

Persönliches
Begleitbuch

zum

Gruppenprogramm
bei Diabetes und Depression

Name: ....

TherapeutIn: ....

Wichtige Telefonnummern:

Wenn Sie zu einer Gruppensitzung nicht kommen können, melden Sie sich bitte bei

.... ab.
(Name) (Telefonnummer)

Informationen zur verpassten Gruppensitzung erteilt Ihnen

....
(Name) (Telefonnummer)

Einleitung

Herzlich Willkommen zu unserem Gruppenprogramm für Patientinnen und Patienten mit Diabetes und Depression!

Wir haben zu jeder Sitzung die wichtigsten Informationen für Sie zusammengestellt, damit Sie sich die Inhalte jeder Zeit noch mal vergegenwärtigen können. Hier finden Sie auch die Aufgabenstellungen und die Arbeitsblätter für die Übungen zwischen den Sitzungen. Am Ende jedes Kapitels haben Sie die Gelegenheit, das Wichtigste jeder Sitzung für sich zu notieren. Wir empfehlen Ihnen, dies zu tun; so können Sie wichtige Erkenntnisse festhalten und auch diese zu einem späteren Zeitpunkt noch einmal nachlesen. Diese Unterlagen dienen Ihnen, wenn Sie möchten, auch über die 10 Gruppensitzungen hinaus als ein Begleiter und Ratgeber.

Sie haben sich dafür entschieden, an diesem Gruppenprogramm teilzunehmen. Damit es für Sie auch tatsächlich effektiv wird, sollten Sie bereit sein, sich auch außerhalb der gemeinsamen Gruppensitzungen mit dem Material zu beschäftigen, und die gestellten Aufgaben - so vollständig wie es Ihnen möglich ist - auszuführen. Je mehr Sie sich bemühen, desto größer wird Ihre Chance, von diesem Programm in Ihrem Alltag zu profitieren.
In der Verhaltenstherapie, zu der dieses Programm zählt, hat sich herausgestellt, dass insbesondere die Übungen zuhause für die erfolgreichsten Therapiefortschritte verantwortlich sind. Auch die Arbeit mit solch einem Patientenbuch hat sich in wissenschaftlichen Studien als therapeutisch sehr wirksam erwiesen.
Wir möchten Sie ermutigen, diese Chance zu nutzen!

Zu Sitzung 1

1. Einige Informationen zu Depressionen und Diabetes

Depressionen kommen in Deutschland häufig vor. Das Risiko, im Laufe seines Lebens an einer Depression zu erkranken, liegt bei ca. 17% (Jakobi et al, 2004).
In einer Studie (Anderson et al, 2001) wurde festgestellt, dass über 11% der Menschen, die an Diabetes erkrankt sind, an Depressionen leiden. Bei Menschen ohne Diabetes sind es laut Andersons Untersuchung dagegen nur 5%. Das hieße also, dass Depressionen bei Diabetikern doppelt so häufig zu finden sind wie bei Nicht-Diabetikern.
Die Gründe dafür werden wir in den folgenden Sitzungen/Kapiteln genauer beschreiben. Der Diabetes kann eine Depression auslösen oder diese aufrechterhalten. Sehr häufig geht eine Depression bei Diabetikern mit einer schlechten Stoffwechseleinstellung einher. Symptome einer Depression sind Freudlosigkeit und Interessenlosigkeit; demnach sehen depressive Diabetiker oft nicht so viel Sinn darin, sich intensiv um das Messen und Spritzen zu kümmern. Allein durch die Depressionen leidet die Lebensqualität sehr. Häufig führt der schlecht eingestellte Diabetes dann zusätzlich zu Schuldgefühlen, so dass die Lebensqualität noch mehr leidet. Meist wird man insgesamt unsicher und zieht sich von Freunden und Bekannten mehr und mehr zurück. Unser Ansatzpunkt wird daher sein, Ihnen zunächst das nötige Wissen zu Diabetes und Depressionen zu vermitteln und dann Strategien zu finden, die helfen, zunächst die Depression und damit auch den HbA_{1c} zu verbessern.

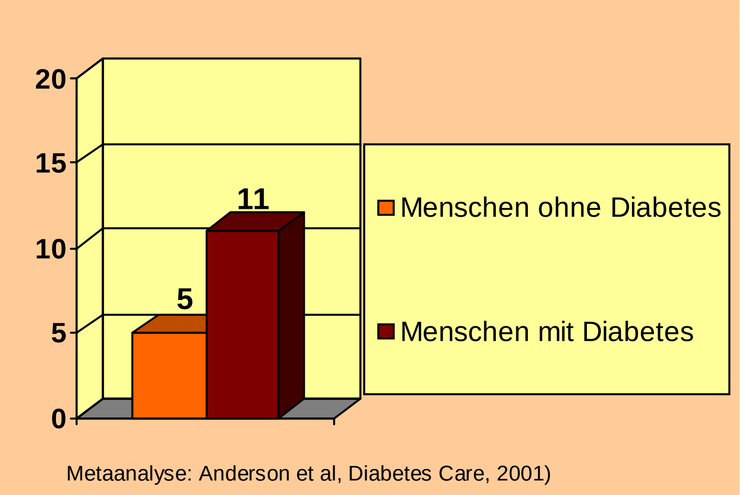

Abbildung 1: Häufigkeit von Depressionen bei Menschen mit Diabetes (Typ 1 und Typ 2) im Vergleich zu Menschen ohne Diabetes

2. Wie beeinflussen sich Aktivitäten und Stimmung?

2.1 Der Zusammenhang zwischen Stimmung und positiven Aktivitäten

Je weniger positive Aktivitäten man durchführt, desto schlechter oder gar depressiver fühlt man sich. Viele Menschen unternehmen, wenn sie sich depressiv fühlen, weniger Dinge, die normalerweise angenehm für sie sind. Was die meisten Menschen dabei jedoch nicht bemerken ist, dass dies die Stimmung nur noch weiter verschlechtert. Durch den Rückzug nimmt man sich die Möglichkeit, Positives zu erleben, das sich positiv auf die Stimmung auswirkt. In der Psychologie nennt man diesen Vorgang „Verstärkerverlust“, da der zurückgezogenen Person die Gelegenheiten fehlen, Positives überhaupt zu erfahren, was ihnen Lebensfreude und Energie zurückgeben könnte.

Wir stellen uns also folgende wichtige Frage: Lässt eine niedrige Zahl positiver Aktivitäten depressive Stimmung entstehen – oder bewirkt depressive Stimmung, dass man weniger unternimmt? Vermutlich ist beides zutreffend. Vieles beeinflusst sich gegenseitig.

Man kann sich diesen Prozess wie einen Teufelskreis oder auch wie eine „Abwärts-Spirale“ vorstellen: Je weniger Sie unternehmen, desto depressiver fühlen Sie sich, und je depressiver Sie sich fühlen, desto weniger tun Sie, was Sie nur noch depressiver macht, usw.

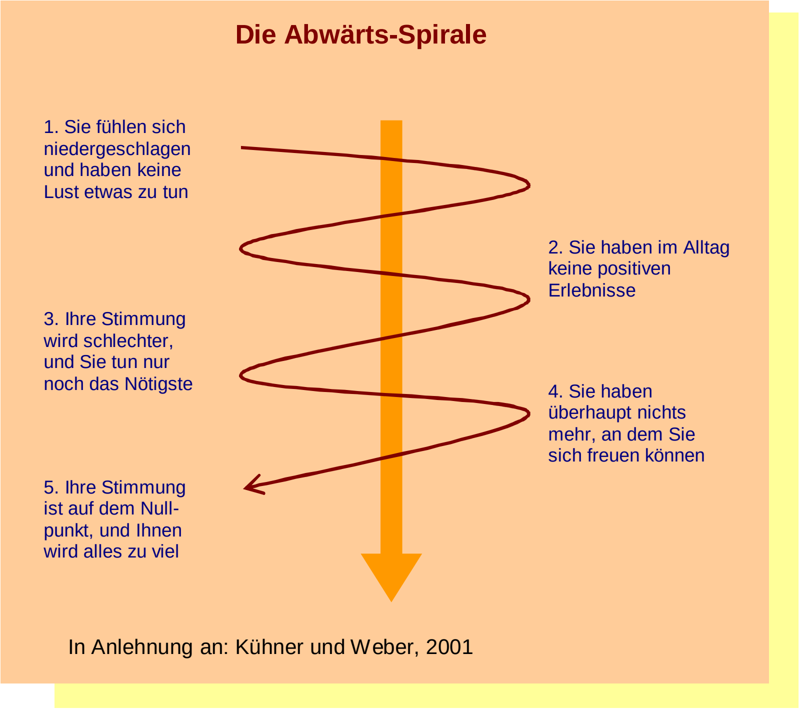

Doch das Erkennen dieses Teufelskreises kann uns dazu führen, die Spirale umzudrehen, denn sie funktioniert auch in die andere Richtung: Je mehr man unternimmt, desto besser fühlt man sich, und je besser man sich fühlt, desto eher unternimmt man etwas.

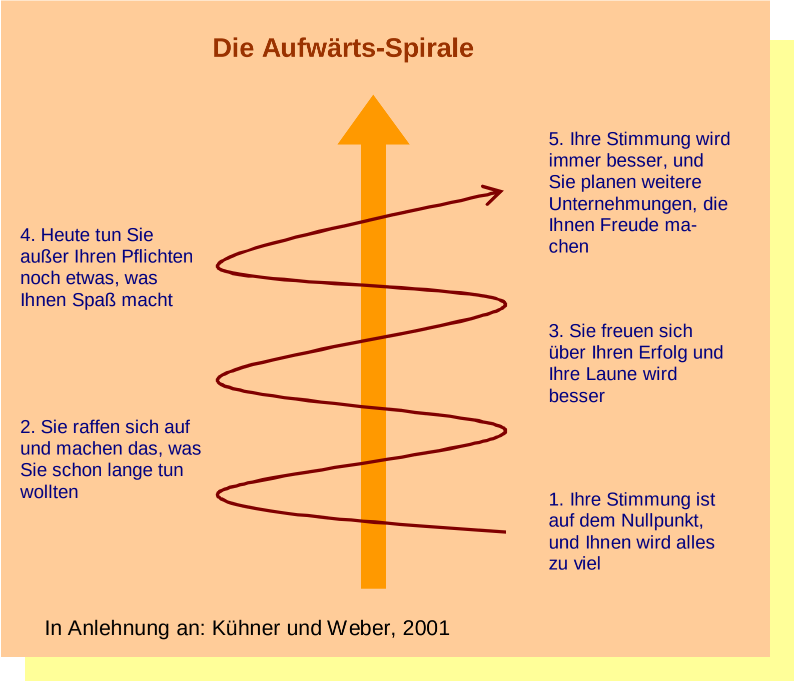

Wir können die „Aufwärts-Spirale“ also dazu nutzen, Ihre Stimmung positiv zu beeinflussen: Es ist insbesondere dann, wenn man sich depressiv fühlt, nicht ganz einfach, sich zu ermutigen, etwas Positives zu tun. Möglicherweise können Sie sich gar nicht vorstellen, an irgendetwas Freude zu empfinden. Sie glauben vielleicht gar nicht, dass es etwas Angenehmes gibt, das Ihre Stimmung tatsächlich positiv beeinflussen könnte. Versuchen Sie sich trotzdem einmal auf die nächsten Schritte einzulassen. Positive Aktivitäten erscheinen häufig nicht sofort als angenehm, manchmal dauert es eine Weile, bis sie sich tatsächlich positiv auf Ihr Wohlbefinden auswirken. Sie werden wahrscheinlich schon sehr bald bemerken, dass es Ihnen ein wenig besser geht.

2.2 Was sind positive Aktivitäten?

Positive Aktivitäten sind für jeden von uns ganz verschieden. Etwas, das von jemandem positiv empfunden wird, mag für Sie oder jemand anderen neutral oder negativ sein. Beispiele lassen sich in allen Lebensbereichen finden:

- Einen Ausflug machen
- Gemütlich eine Tasse Tee trinken
- Sport treiben
- Ins Kino gehen
- Zu einer Familienfeier gehen
- Einen guten Film anschauen
- Mit Freunden im Café sitzen
- Einkaufen
- Etwas Leckeres essen
- Usw.

Positive Aktivitäten müssen nicht unbedingt besondere oder außergewöhnliche Erlebnisse sein. Häufig sind es sogar ganz alltägliche Aktivitäten.

Positive Aktivitäten können sowohl Aktivitäten sein, bei denen Sie sich bewegen oder die Sie mit anderen Menschen gemeinsam erleben (z.B. irgendetwas unternehmen). Es können aber auch Aktivitäten sein, die Sie eher innerlich oder gedanklich machen (z.B. sich an etwas Schönes erinnern, Träumen).

2.3 Persönliche positive Aktivitäten finden

Menschen, die sich depressiv fühlen haben meist Schwierigkeiten, sich positive Aktivitäten vorzustellen.
Eventuell können Sie sich auch nicht mehr gut erinnern, welche Aktivitäten Ihnen Freude bereiten könnten. Vielleicht haben Sie sie schon lange nicht mehr durchgeführt.
Auf dem Arbeitsblatt 2 ist eine ganze Reihe positiver Ereignisse aufgelistet, vielleicht nützen Ihnen diese Anregungen, um für sich selbst herauszufinden, welche Aktivitäten für Sie positiv sind oder sein könnten.

2.4 Wie können Sie sich selbst beobachten?

Damit Sie entdecken können, ob es einen Zusammenhang zwischen Ihrer Stimmung und der Durchführung verschiedener Aktivitäten gibt, ist es sinnvoll, sich selbst einmal im Alltag zu beobachten.
Vielleicht fallen Ihnen Stimmungsschwankungen auf, die zuvor nicht unbedingt bemerkbar waren. Solche gewonnenen Informationen über das eigene Erleben werden wir dann in den nächsten Sitzungen nutzen, um Ihre Stimmung verbessern zu können.
Eine solche Selbstbeobachtung können Sie mithilfe des Arbeitsblattes 3 durchführen (s. Übungsaufgaben).

Persönliche Notizen zu dieser Sitzung

....
....
....
....
....
....
....
....
....
....
....
....
....
....
....
....
....
....

Übungsaufgaben der 1. Sitzung:

1. Vervollständigen Sie Ihre persönliche Liste positiver Aktivitäten auf dem Arbeitsblatt 1. Wenn Sie möchten, können Sie als Hilfestellung das Arbeitsblatt 2 “Liste Positiver Ereignisse” hinzuziehen.

2. Wir empfehlen Ihnen, in der nächsten Woche Ihre Stimmung einmal selbst zu beobachten. Tragen Sie das, was sie tagsüber machen, sowie Stimmungswerte auf dem Arbeitsblatt 3 ein.

3. Falls Sie in Vorbereitung auf dieses Programm noch nicht mit Ihrem Arzt, der Ihre Diabetesbehandlung betreut, besprochen haben, wodurch Sie Ihre Stoffwechseleinstellung verbessern können, empfehlen wir Ihnen dies dringend zu tun. Vereinbaren Sie dazu möglichst kurzfristig einen Termin bei Ihrem Arzt, und besprechen Sie mit ihm anhand des Arbeitsblattes 4, welche diabetesbezogenen Therapieziele er Ihnen empfiehlt.

Bitte bringen Sie alle Unterlagen des Gruppenprogramms zu allen Sitzungen mit!

ARBEITSBLATT 1

MEINE PERSÖNLICHE LISTE POSITIVER AKTIVITÄTEN (TAG 1 – 29)

1.																													
2.																													
3.																													
4.																													
5.																													
6.																													
7.																													
8.																													
9.																													
10.																													
11.																													
12.																													
13.																													
14.																													
15.																													
	1	2	3	4	5	6	7	8	9	10	11	12	13	14	15	16	17	18	19	20	21	22	23	24	25	26	27	28	29

Tage

MEINE PERSÖNLICHE LISTE POSITIVER AKTIVITÄTEN (TAG 1 – 29)

16.																													
17.																													
18.																													
19.																													
20.																													
21.																													
22.																													
23.																													
24.																													
25.																													
26.																													
27.																													
28.																													
29.																													
30.																													
	1	2	3	4	5	6	7	8	9	10	11	12	13	14	15	16	17	18	19	20	21	22	23	24	25	26	27	28	29

Tage

MEINE PERSÖNLICHE LISTE POSITIVER AKTIVITÄTEN (TAG 30 – 58)

1.																													
2.																													
3.																													
4.																													
5.																													
6.																													
7.																													
8.																													
9.																													
10.																													
11.																													
12.																													
13.																													
14.																													
15.																													
	30	31	32	33	34	35	36	37	38	39	40	41	42	43	44	45	46	47	48	49	50	51	52	53	54	55	56	57	58

Tage

MEINE PERSÖNLICHE LISTE POSITIVER AKTIVITÄTEN (TAG 30 – 58)

16.																													
17.																													
18.																													
19.																													
20.																													
21.																													
22.																													
23.																													
24.																													
25.																													
26.																													
27.																													
28.																													
29.																													
30.																													
	30	31	32	33	34	35	36	37	38	39	40	41	42	43	44	45	46	47	48	49	50	51	52	53	54	55	56	57	58

Tage

ARBEITSBLATT 2
LISTE POSITIVER EREIGNISSE

Im folgenden finden Sie eine lange Liste von Ereignissen, die für manche Menschen positiv sind. Diese Liste können Sie benutzen, um Ihre persönliche Liste positiver Aktivitäten (Arbeitsblatt 1) zu erstellen oder zu vervollständigen.

1. Ins Grüne fahren
2. Für einen guten Zweck spenden
3. Sich über Sport unterhalten
4. Eine neue Bekanntschaft machen (gleiches Geschlecht)
5. Gut vorbereitet eine Prüfung bestehen
6. Zu einem Pop-Konzert gehen
7. Federball spielen
8. Ausflüge oder Urlaubsfahrten planen
9. Für sich selbst Dinge einkaufen
10. Am Strand sein
11. Sich künstlerisch betätigen (Malerei, Bildhauerei, Zeichnen, Filme drehen, usw.)
12. Kletterfahrten oder Bergtouren machen
13. Die Bibel oder andere religiöse Schriften lesen
14. Golf oder Minigolf spielen
15. Zimmer oder Haus auf- oder umräumen
16. Nackt herumlaufen
17. Zu einer Sportveranstaltung gehen
18. Zu Rennveranstaltungen gehen (Pferde-, Auto-, Bootsrennen, usw.)
19. Tipps und Ratschläge zur Selbsthilfe lesen
20. Romane, Erzählungen, Theaterstücke oder Gedichte lesen
21. In ein Lokal gehen
22. Zu Vorträgen gehen
23. Autofahren
24. Ein Lied oder ein Musikstück texten oder komponieren
25. Eine Sache klipp und klar sagen
26. Segeln, Motorboot oder Kanu fahren
27. Seinen Eltern Freude bereiten
28. Antiquitäten restaurieren, Möbel aufarbeiten (restaurieren)
29. Fernsehen
30. Selbstgespräche führen
31. Zelten
32. Sich politisch betätigen
33. An technischen Dingen arbeiten (Autos, Fahrräder, Motorräder, Hausgeräte, usw.)
34. Positive Zukunftspläne schmieden
35. Teure oder exklusive Kleidung tragen
36. Karten spielen
37. Eine schwierige Aufgabe meistern
38. Lachen
39. Puzzle, Kreuzworträtsel usw. lösen
40. An Hochzeiten, Taufen, Konfirmationen, usw. teilnehmen
41. Jemanden kritisieren
42. Mit Freunden oder Bekannten zusammen essen
43. Tennis spielen
44. Eine Dusche nehmen
45. Lange Strecken fahren
46. Holz- oder Schreinerarbeiten ausführen
47. Romane, Erzählungen, Theaterstücke oder Gedichte schreiben
48. Sich mit Tieren beschäftigen
49. Mit einem Flugzeug fliegen
50. Erkundigungsgänge machen (von gewohnten Straßen abweichen, unbekannte Gegenden erforschen usw.)

51. Eine offene und ehrliche Unterhaltung führen
52. In einem Chor singen
53. Über sich selbst oder seine Probleme nachdenken
54. Sich beruflich engagieren
55. Zu einer Party gehen
56. Eine Fremdsprache sprechen
57. Zu kirchlichen Veranstaltungen gehen (Wohltätigkeitsveranstaltungen, Vorträge, Bazare, usw.)
58. Zu Versammlungen von gemeinnützigen oder sozialen Vereinen gehen
59. An einer Tagung teilnehmen
60. Einen Luxus- oder Sportwagen fahren
61. Ein Musikinstrument spielen
62. Skilaufen
63. Geholfen bekommen
64. Leger gekleidet zu sein
65. Sein Haar kämmen oder bürsten
66. Schauspielerisch tätig sein
67. Ein Nickerchen machen
68. Mit Freunden zusammen sein
69. Lebensmittel einmachen, einfrieren, Vorräte anlegen
70. Schnell fahren
71. Ein persönliches Problem lösen
72. Ein Bad nehmen
73. Vor sich hinsingen
74. Billard spielen
75. Mit seinen Enkelkindern zusammen sein
76. Schach oder Dame spielen
77. Mit künstlerischen Materialien arbeiten (Ton, Schmuck, Leder, Perlen, Weben, usw.)
78. Zum Zirkus oder Zoo gehen
79. Sich kratzen
80. Make-up auflegen, sein Haar richten, usw.
81. Etwas entwerfen oder zeichnen
82. Leute besuchen, die krank, inhaftiert oder sonst wie in Schwierigkeiten sind
83. Fröhlich sein, gute Stimmung verbreiten
84. Bowling spielen gehen
85. Tiere beobachten
86. Eine originelle Idee haben
87. Garten-, Landschafts- oder Hofarbeit verrichten
88. Fachliteratur oder Sachbuch lesen
89. Neue Kleidung tragen
90. Tanzen
91. In der Sonne sitzen
92. Motorrad fahren
93. Nur so herumsitzen und nachdenken
94. In geselliger Runde etwas trinken
95. Miterleben, wie seiner Familie oder seinen Freunden etwas Positives widerfährt
96. Einen Vergnügungspark besuchen
97. Sich über Philosophie oder Religion unterhalten
98. Um Geld spielen
99. Etwas planen oder organisieren
100. Friedhof besuchen
101. Alleine etwas trinken
102. Den Geräuschen der Natur zuhören
103. Verabredungen treffen, um mit jemandem zu flirten
104. Eine lebhafte Unterhaltung führen
105. Rennen fahren
106. Radio hören
107. Besuch von Freunden bekommen
108. An einem sportlichen Wettbewerb teilnehmen
109. Leute einander vorstellen, von denen man annimmt, sie könnten einander gut verstehen
110. Geschenke machen
111. Zu Gerichtsverhandlungen gehen
112. Massiert werden
113. Briefe erhalten

114. Den Himmel, Wolken oder einen Sturm beobachten
115. Sich im Freien aufhalten (in einen Park, Garten, zum Picknick, Grillen, usw. gehen)
116. Basketball oder Volleyball spielen
117. Seiner Familie etwas kaufen
118. Fotografieren
119. Eine Rede oder einen Vortrag halten
120. Landkarten studieren
121. Dinge aus der Natur sammeln (wilde Früchte oder Beeren, Steine, Treibholz, usw.)
122. Seine finanziellen Angelegenheiten regeln
123. Saubere Kleidung tragen
124. Eine Anschaffung oder Investition tätigen (Auto, Geräte, Haus- oder Vorratsgegenstände)
125. Jemandem helfen
126. Sich um neue Arbeit bewerben
127. Witze anhören
128. Eine Wette gewinnen
129. Über seine Kinder oder Enkel sprechen
130. Jemand neuen vom anderen Geschlecht kennen lernen
131. Über seine Gesundheit sprechen
132. Gut essen
133. Etwas für seine Gesundheit tun (die Zähne in Ordnung bringen lassen, eine neue Brille bekommen, usw.)
134. In der Stadt herumbummeln
135. Ringen oder boxen
136. Schießsport betreiben
137. In einer Musikgruppe mitspielen
138. Wandern
139. Ein Museum oder eine Ausstellung besuchen
140. Tagebuch schreiben
141. Eine Aufgabe gut ausführen
142. Freizeit haben
143. Angeln gehen
144. Etwas verleihen
145. Als sexuell attraktiv bemerkt werden
146. Arbeitgebern, Lehrern, usw. eine Freude bereiten
147. Jemanden beraten
148. In ein Fitness-Center, eine Sauna usw. gehen
149. Etwas Neues lernen
150. Jemandem Komplimente machen oder ihn loben
151. Über Leute nachdenken, die man mag
152. An jemandem Rache nehmen
153. Mit seinen Eltern zusammen sein
154. Reiten
155. Telefongespräche führen
156. Tagträumen
157. Blätter, Sand, Kieselsteine, usw. herumkicken
158. Boccia spielen
159. Zu Klassen-, Alterstreffen und der gleichen gehen
160. Berühmte Leute sehen
161. Ins Kino gehen
162. Küssen
163. Allein sein
164. Essen kochen
165. Einen Neunmalklugen hereinlegen
166. "Gelegenheitsarbeiten" im Hause verrichten
167. Weinen
168. Gesagt bekommen, dass man gebraucht wird
169. An einem Treffen oder einer Feier der Familie teilnehmen
170. Eine Party oder ein gemütliches Beisammensein veranstalten
171. Seine Haare waschen
172. Jemandem Anweisungen erteilen
173. Eine Blume oder Pflanze sehen oder riechen
174. Zum Ausgehen eingeladen werden
175. Kölnischwasser, Parfüm benutzen
176. Mit jemandem derselben Meinung sein

177. In Erinnerungen schwelgen, von früheren Zeiten sprechen
178. Morgens früh aufstehen
179. Ruhe finden
180. Experimente oder andere wissenschaftliche Versuche durchführen
181. Freunde besuchen
182. Sich beraten lassen, einen Rat erteilt bekommen
183. Beten
184. Jemanden massieren
185. Per Anhalter reisen
186. Meditation oder Yoga betreiben
187. Einem Kampf zusehen
188. Mit Arbeits- oder Klassenkameraden sprechen
189. Entspannt sein
190. Um Rat oder Hilfe gebeten werden
191. Über anderer Leute Probleme nachdenken
192. Gesellschaftsspiele spielen
193. Nachts tief und fest schlafen
194. Schwere Arbeiten im Freien verrichten (Bäume fällen oder Holz hacken, Gartenarbeiten)
195. Zeitung lesen
196. Leute schockieren, fluchen, obszöne Gesten machen, usw.
197. An einer Selbsterfahrungsgruppe teilnehmen
198. Tischtennis spielen
199. Sich die Zähne putzen
200. Schwimmen
201. An einem Kampf beteiligt sein
202. Laufen, Jogging, Gymnastik, Fitness oder Freiluftübungen betreiben
203. Barfuss laufen
204. Ein Wurfspiel oder Fangen spielen
205. Hausarbeit oder Wäsche erledigen; saubermachen
206. Musik hören
207. Sexuelle Befriedigung haben
208. Stricken, Häkeln, Sticken oder phantasievolle Näharbeiten ausführen
209. Schmusen
210. Leute erheitern
211. Über Sexualität sprechen
212. Zu einem Friseur oder Kosmetiker gehen
213. Gäste im Haus haben
214. Mit jemandem zusammen sein, den man mag
215. Zeitschriften lesen
216. Ausschlafen
217. Ein neues Vorhaben beginnen
218. Eigensinnig sein
219. Diskutieren
220. In eine Bibliothek gehen
221. Fußball oder Handball spielen
222. Ein neues oder spezielles Gericht zubereiten
223. Vögel beobachten
224. Einen Einkaufsbummel machen
225. Leute beobachten
226. Ein Feuer anzünden oder beobachten
227. Eine Auseinandersetzung erfolgreich bestehen
228. Etwas verkaufen oder mit etwas handeln
229. Ein Vorhaben oder eine Aufgabe zu Ende bringen
230. Etwas beichten oder für etwas Abbitte leisten
231. Gegenstände reparieren
232. Radfahren
233. Leuten sagen, was sie zu tun haben
234. Mit fröhlichen Menschen zusammen sein
235. Partyspiele spielen
236. Briefe, Karten schreiben
237. Über Politik oder öffentliche Angelegenheiten reden
238. Um Hilfe oder Ratschläge bitten
239. Über sein Hobby oder spezielles Interessensgebiet reden

240. Attraktive Frauen oder Männer beobachten
241. Leuten zulächeln
242. Im Sand, Gras oder an einem Fluss spielen
243. Über andere Leute reden
244. Mit seinem Ehepartner zusammen sein
245. Von anderen Leuten Interesse an seinen Äußerungen gezeigt bekommen
246. Tabak rauchen
247. Sich um Zimmerpflanzen kümmern
248. Mit Freunden Kaffee, Tee trinken
249. Einen Spaziergang machen
250. Verschiedene Dinge sammeln
251. Nähen
252. Sich an einen verstorbenen Freund erinnern
253. Mit Kindern gemeinsam etwas unternehmen
254. Komplimente erhalten oder hören, dass man etwas gut gemacht hat
255. Gesagt bekommen, dass man geliebt wird
256. Imbisse zu sich nehmen
257. Abends lange aufbleiben
258. Mit seinen Kindern zusammen sein
259. Zu Auktionen, Versteigerungen, usw. gehen
260. Über eine interessante Frage nachdenken
261. Freiwillige Arbeit tun, an gemeinnützigen Projekten mitarbeiten
262. Wasserski laufen, Surfen, Tauchen
263. Jemanden verteidigen oder in Schutz nehmen; gegen Betrug und Missbrauch einschreiten
264. Einen Anhalter mitnehmen
265. Einen Wettbewerb gewinnen
266. Über Beruf oder Schule sprechen
267. Cartoons, Comic-Hefte lesen
268. Etwas ausleihen
269. An einer Gruppenreise teilnehmen
270. Alte Freunde wiedertreffen
271. Reisen
272. Ein Konzert, eine Opern- oder Ballettaufführung besuchen
273. Mit Haustieren spielen
274. Ein Theaterstück besuchen
275. Die Sterne oder den Mond betrachten

(in Anlehnung an Hautzinger, 1997)

ARBEITSBLATT 3
STIMMUNGSBEOBACHTUNG

Einschätzung der Stimmung
Tragen Sie hier folgende Werte für Ihre Stimmung ein:
1 für sehr gute
2 für gute
3 für mittelmäßige
4 für weniger gute
5 für schlechte
6 für sehr schlechte Stimmung

Datum:

	Situation, Tätigkeit im Augenblick	Stimmung
7- 8 Uhr		
8- 9 Uhr		
9-10 Uhr		
10-11 Uhr		
11-12 Uhr		
12-13 Uhr		
13-14 Uhr		
14-15 Uhr		
15-16 Uhr		
16-17 Uhr		
17-18 Uhr		
18-19 Uhr		
19-20 Uhr		
20-21 Uhr		
21-22 Uhr		
22-23 Uhr		
23-24 Uhr		

Datum:

	Situation, Tätigkeit im Augenblick	Stimmung
7- 8 Uhr		
8- 9 Uhr		
9-10 Uhr		
10-11 Uhr		
11-12 Uhr		
12-13 Uhr		
13-14 Uhr		
14-15 Uhr		
15-16 Uhr		
16-17 Uhr		
17-18 Uhr		
18-19 Uhr		
19-20 Uhr		
20-21 Uhr		
21-22 Uhr		
22-23 Uhr		
23-24 Uhr		

STIMMUNGSBEOBACHTUNG

Einschätzung der Stimmung
Tragen Sie hier folgende Werte für Ihre Stimmung ein:
1 für sehr gute
2 für gute
3 für mittelmäßige
4 für weniger gute
5 für schlechte
6 für sehr schlechte Stimmung

Datum:

	Situation, Tätigkeit im Augenblick	Stimmung
7- 8 Uhr		
8- 9 Uhr		
9-10 Uhr		
10-11 Uhr		
11-12 Uhr		
12-13 Uhr		
13-14 Uhr		
14-15 Uhr		
15-16 Uhr		
16-17 Uhr		
17-18 Uhr		
18-19 Uhr		
19-20 Uhr		
20-21 Uhr		
21-22 Uhr		
22-23 Uhr		
23-24 Uhr		

Datum:

	Situation, Tätigkeit im Augenblick	Stimmung
7- 8 Uhr		
8- 9 Uhr		
9-10 Uhr		
10-11 Uhr		
11-12 Uhr		
12-13 Uhr		
13-14 Uhr		
14-15 Uhr		
15-16 Uhr		
16-17 Uhr		
17-18 Uhr		
18-19 Uhr		
19-20 Uhr		
20-21 Uhr		
21-22 Uhr		
22-23 Uhr		
23-24 Uhr		

STIMMUNGSBEOBACHTUNG

Einschätzung der Stimmung
Tragen Sie hier folgende Werte für Ihre Stimmung ein:
- **1** für sehr gute
- **2** für gute
- **3** für mittelmäßige
- **4** für weniger gute
- **5** für schlechte
- **6** für sehr schlechte Stimmung

Datum:

	Situation, Tätigkeit im Augenblick	Stimmung
7- 8 Uhr		
8- 9 Uhr		
9-10 Uhr		
10-11 Uhr		
11-12 Uhr		
12-13 Uhr		
13-14 Uhr		
14-15 Uhr		
15-16 Uhr		
16-17 Uhr		
17-18 Uhr		
18-19 Uhr		
19-20 Uhr		
20-21 Uhr		
21-22 Uhr		
22-23 Uhr		
23-24 Uhr		

Datum:

	Situation, Tätigkeit im Augenblick	Stimmung
7- 8 Uhr		
8- 9 Uhr		
9-10 Uhr		
10-11 Uhr		
11-12 Uhr		
12-13 Uhr		
13-14 Uhr		
14-15 Uhr		
15-16 Uhr		
16-17 Uhr		
17-18 Uhr		
18-19 Uhr		
19-20 Uhr		
20-21 Uhr		
21-22 Uhr		
22-23 Uhr		

ARBEITSBLATT 4
EMPFEHLUNGEN ZUR SENKUNG DES HbA_{1C}

Patient:________________________ **Datum:** _____________ **ggf. Visit:**____

Vom Arzt auszufüllen:

Folgende Verhaltensweisen könnten Ihnen helfen, Ihre Stoffwechseleinstellung langfristig zu verbessern (= $HbA1_c$ um mindestens 1% zu senken), und dadurch die Wahrscheinlichkeit von Spätschäden durch den Diabetes zu verringern:

A) ____

B) ____

C) ____

Vom Patienten auszufüllen:

Bitte tragen Sie in die Kästchen für jede Empfehlung (A bis C) ihre persönliche Bewertung auf einer Skala von 0-10 ein.
0 = gar nicht wichtig/schwierig bis 10 = maximal wichtig/schwierig

	A	B	C
Wie wichtig ist **mir persönlich** die Umsetzung dieser Empfehlung?			
Wie schwierig erscheint mir die Umsetzung dieser Empfehlung?			

Folgende **Lösungsmöglichkeiten** zur Umsetzung der Empfehlungen habe **ich mit meinem Arzt** besprochen:

Zu A) ____

Zu B) ____

Zu C) ____

Zu Sitzung 2

1. Wie entstehen Depressionen und wie werden sie aufrechterhalten?

1.1 Was ist eine Depression?

Depression kommt aus dem Lateinischen, ***deprimere*** bedeutet niederdrücken. Als Depression beschreibt man ein seelisches Leiden, dass u.a. folgende Symptome einschließt:

- Sich niedergeschlagen, traurig, bedrückt oder gereizt zu fühlen
- Kein Interesse und keine Freude mehr zu empfinden
- Erschöpft und antriebslos zu sein
- Sich nicht mehr konzentrieren oder entscheiden zu können
- Keinen Appetit mehr zu haben und Gewicht verloren zu haben, ohne eine Diät eingehalten zu haben, oder aber vermehrten Appetit zu haben und deutlich an Gewicht zugenommen zu haben
- Nicht mehr schlafen zu können oder vermehrt schlafen zu müssen
- Sich schuldig oder wertlos zu fühlen
- Lebensmüde Gedanken zu haben, Gedanken an den Tod zu haben
- U.a.

Von einer Depression spricht man dann, wenn eine Mehrzahl dieser Symptome über einen längeren Zeitraum (mindestens zwei Wochen) so stark ausgeprägt ist, dass man von ihnen deutlich beeinträchtigt wird.

Wenn Sie möchten können Sie hier **persönliche Symptome**, die Sie an sich festgestellt haben, benennen:

______________________	______________________
______________________	______________________
______________________	______________________
______________________	______________________
______________________	______________________
______________________	______________________
______________________	______________________
______________________	______________________

1.2 Wie entsteht eine Depression?

Wenn man verstehen möchte, wie Depressionen entstehen, ist es sinnvoll den Blick auf eine Reihe von Ursachen zu richten, die sich gegenseitig bedingen. Heutzutage geht man davon aus, dass eine Depression durch das Zusammenwirken folgender Faktoren zu erklären ist:

1. **“Prädisponierende” Faktoren**: Damit sind Faktoren gemeint, die es wahrscheinlicher machen, dass man später im Verlauf seines Lebens eine Depression bekommt. Hierzu zählen Veränderungen bei einigen Genen, was dazu führt, dass man anfälliger für den Ausbruch von Depressionen wird. Ein Indiz für eine genetische Belastung ist, dass in der Familie viele Verwandte depressiv geworden sind.

   Allein diese Faktoren erklären nicht das Auftreten einer Depression, sie erhöhen nur die Anfälligkeit für eine solche Erkrankung.

   Zu diesen “prädisponierenden” Faktoren zählen aber auch ständige, lang andauernde Belastungen, die zu einer chronischen Belastung oder Überlastung führen. Z.B. können andauernde Erkrankungen während des Säuglingsalters oder belastende Erlebnisse in der Kindheit, wie z.B Misshandlungen, zu einer Störung der Stressverarbeitung führen, d.h. dass man noch im Erwachsenenalter empfindlicher auf Stress reagiert. Das wiederum begünstigt die Entstehung einer Depression.

   Damit eine Depression tatsächlich auftritt, müssen meistens noch weitere, auslösende Faktoren hinzukommen.

2. **Auslösende Faktoren**: Meist sind diese Faktoren Ereignisse, die als "Stress" oder Überforderung empfunden werden, z.B. der Tod eines nahe stehenden Menschen, der Verlust des Arbeitsplatzes, ein Umzug an einen anderen Ort, oder auch eine Krankheit wie der Diabetes oder das Auftreten von Folgeerkrankungen, das heißt eine Verschlimmerung einer Erkrankung.

3. **Aufrechterhaltende Faktoren**: Bestimmte Faktoren können dazu führen, dass depressive Verstimmungen länger andauern und zu einer Depression werden, z.B. die ständige Sorge um die eigene Gesundheit oder die von anderen. Oder das Erleben, dass das Bemühen um gute Blutzuckerwerte nicht zu den erhofften Werten führt. Solch ein Gefühl von Hilflosigkeit kann man aber auch in anderen Bereichen erleben, z.B. im Beruf, wo vielleicht die Anerkennung für viel Arbeit ausbleibt. Oder auch Schwierigkeiten im Privatleben, dass jemand sich z.B. hinsichtlich seiner Umgehensweise mit dem Diabetes nicht verstanden fühlt.

1.3 Wie werden Depressionen aufrechterhalten? Denken, Fühlen, Handeln

Menschliches Leben und Erleben findet auf unterschiedlichen Ebenen statt, die sich gegenseitig beeinflussen. In unterschiedlichen Lebenssituationen denken, fühlen und handeln wir auf unterschiedliche Art und Weise. Bestimmt hat jeder schon einmal erlebt, dass er in schlechter Stimmung auch negativere Gedanken hat und weniger Lust, etwas Positives zu unternehmen. Oder dass eine unangenehme Tätigkeit ihn in schlechtere Stimmung versetzt und zu pessimistischen Gedanken angeregt hat. Nicht nur Stimmung und Aktivitäten beeinflussen sich gegenseitig, sondern zusätzlich spielen Gedanken eine wichtige Rolle. Durch die gegenseitige Beeinflussung dieser drei Ebenen wird eine Depression aufrechterhalten oder sogar verstärkt.

Besonders in Zeiten, in denen man sich depressiv fühlt, kann es einem von Nutzen sein, einmal den Zusammenhang zwischen dem eigenen Denken, Fühlen und Handeln genauer zu betrachten und Ansatzpunkte kennen zu lernen, wie man das eigene Erleben des Alltags beeinflussen kann.

Von allen drei Ebenen ist die des Fühlens am schwersten direkt zu beeinflussen. Kaum jemand ist in der Lage, einfach zu beschließen, sich gut zu fühlen, und diesen Vorsatz umzusetzen. Im Vergleich dazu ist es einfacher, sich die Ebene des Handelns einmal genauer anzuschauen, zu beobachten und nach Möglichkeiten der Veränderung zu suchen. Dies haben wir bereits in der ersten und dieser zweiten Sitzungen thematisiert.

In der dritten Sitzung werden wir uns noch genauer der dritte Ebene, der des Denkens widmen.

Sicherlich haben Sie schon beim Lesen bemerkt, dass einige der möglichen Risikofaktoren zur Entstehung und Aufrechterhaltung einer Depression auch für Sie zutreffend sind. Es lassen sich aber sicherlich noch mehrere, nicht genannte Bedingungen finden:

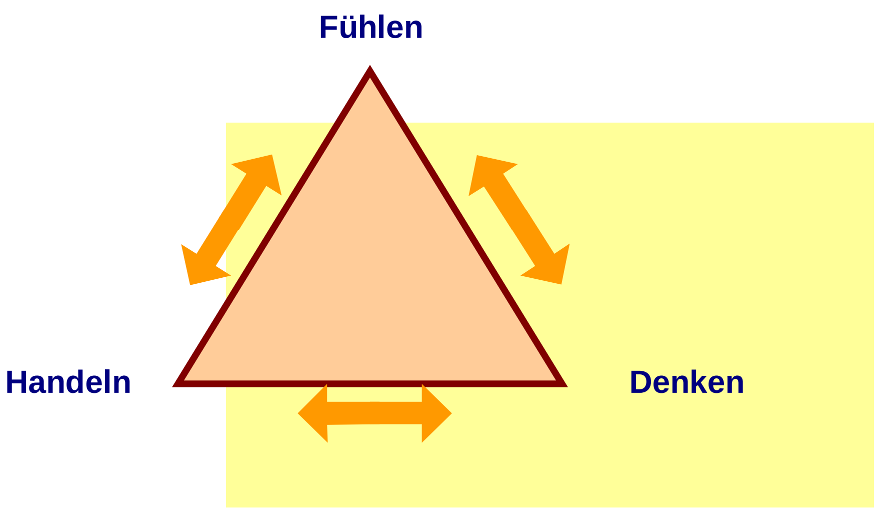

Versuchen Sie für sich **persönlich** “prädisponierende” Faktoren, die die spätere Entstehung Ihrer Depression vielleicht schon im Vorfeld wahrscheinlicher gemacht haben, Faktoren, die bei Ihnen Auslöser gewesen sein könnten, und Bedingungen, die zur Aufrechterhaltung depressiver Verstimmung beigetragen haben, zu sammeln:

„Prädisponierende“ Faktoren
Auslösende Bedingungen
Aufrechterhaltende Faktoren

2. Festlegen von Therapiezielen

Wir alle nehmen uns immer wieder Dinge vor, die wir erreichen wollen. Manche davon beziehen sich auf Dinge, die schnell erreicht werden können oder wollen, andere beziehen sich auf einen längeren Zeitraum oder sind sogar so genannte Lebensziele.

Leider müssen wir immer wieder feststellen, dass wir einige unserer Vorhaben nicht erreichen. Haben Sie sich z.B. schon zu Neujahr einen Vorsatz gemacht und diesen nicht umgesetzt? Das passiert sehr häufig.

Im Rahmen unseres Programms ist es sinnvoll, sich zu überlegen, was Sie hinsichtlich Ihrer Stimmung und Ihres Diabetes überhaupt erreichen möchten. D.h. wir legen Therapieziele fest. Folgende Regeln können helfen, damit der Erfolg bei der Umsetzung dieser Ziele wahrscheinlicher ist:

1. Günstig ist es, wenn Therapieziele **realistisch** sind, d.h. dass Sie auch eine Chance haben, sie zu erreichen.
2. Allgemein formulierte Therapieziele sind hinsichtlich Ihres Erfolges meist nicht überprüfbar. Sie bleiben abstrakt. Hilfreich ist es, dass Sie versuchen, Ihre Therapieziele möglichst **konkret** zu formulieren. Sinnvoll kann dabei auch sein, Teilziele auszuwählen.
3. **Positiv** formulierte Therapieziele drücken genauer aus, was Sie möchten und sind motivierender.
4. Wichtig ist es, dass Sie sich klar machen, dass jedes Ziel in einem **bestimmten Zeitraum** erreicht werden kann. Dadurch lässt sich die Zielerreichung einfacher im Auge behalten. Versuchen Sie zu Ihrem Therapieziel einen Zeitraum festzulegen, indem Sie es erreichen können und wollen.

Ziele wie "Ich möchte immer einen perfekten Blutzucker haben“ oder "Ich möchte, dass es mir immer gut geht" sind wahrscheinlich für die meisten Menschen nur schwer erreichbar. Schwierig ist auch ein allgemein formuliertes Ziel wie "Ich will zufrieden sein im Leben!".

Beispiele, die sich vom letzten Ziel nach den oben genannten Regeln ableiten lassen, sind:

- ***Ich möchte wieder ein Wochenende an die Nordsee fahren.***
- ***Ich möchte zwei Mal in der Woche eine Stunde spazieren gehen.***
- ***Ich werde drei Mal täglich meinen Blutzucker kontrollieren.***

Persönliche Notizen zu dieser Sitzung

Übungsaufgaben der 2. Sitzung:

1. Versuchen Sie anhand des Arbeitsblattes 5 mindestens
   - ein Therapieziel, welches Sie in den nächsten 4 Wochen erreichen möchten,
   - ein weiteres, welches Sie im Verlauf von Monaten, und
   - eins, das Sie in einem längeren Zeitraum erreichen möchten,

   festzulegen.
   Dabei ist es sinnvoll, wenn sich mindestens eins der Therapieziele auf die **Durchführung positiver Aktivitäten** und mindestens eins auf die **Verbesserung Ihres HbA_{1c}-Wertes** bezieht. Zur Festlegung eines diabetesbezogenen Therapiezieles sollten Sie die Ergebnisse der vergangenen Besprechung mit Ihrem Arzt (Arbeitsblatt 4) einbeziehen.

2. Notieren Sie in Ihrer persönlichen Liste positiver Aktivitäten (Arbeitsblatt 1), welche Aktivitäten Sie pro Tag wie oft durchgeführt haben.

3. Protokollieren Sie weiter ihre Stimmung und die Durchführung positiver Aktivitäten - auf dem Arbeitsblatt 6.

Wir empfehlen Ihnen, das Arbeitsblatt 1 und 6 ab der 2. Sitzung (Tag 1) bis zur 10. Sitzung (Tag 58) weiterzuführen!

Sie sollten bei jedem Arztbesuch Empfehlungen zur Senkung Ihres HbA_{1c} mit Ihrem Arzt besprechen und diese auf dem Arbeitsblatt 4 protokollieren!

ARBEITSBLATT 5
THERAPIEZIELE

Legen Sie bitte fest, welche konkreten Ziele Sie erreichen wollen, bezogen auf die Bewältigung Ihrer Depressionen und den Umgang mit Ihrem Diabetes.

Siehe dazu auch Patientenbuch S. 9-10:

Wie Therapieziele formuliert werden sollten:

1. **Realistisch**
2. **Konkret**
3. **Positiv**
4. **Festgelegter Zeitraum,** indem das Ziel erreicht werden kann.

Beispiel für ein allgemeines Ziel:

- Ich will zufrieden sein im Leben.

Beispiele für daraus abgeleitete konkrete, positive Ziele:

- Ich möchte wieder ein Wochenende an die Nordsee fahren.
- Ich möchte zwei Mal in der Woche eine Stunde spazieren gehen.
- Ich werde drei Mal täglich meinen Blutzucker kontrollieren.

Es hat sich als erfolgreich erwiesen, nicht zu viele Therapieziele auf einmal erreichen zu wollen.
Wir empfehlen Ihnen, mindestens ein Ziel hinsichtlich der Durchführung positiver Aktivitäten und ein Ziel bezüglich der Besserung Ihres HbA_{1c} zu formulieren.

**Datum: ____________** **Meine Therapieziele lauten:**	**Datum der Überprüfung**	**Ziel erreicht?** Kreuzen Sie bitte auf folgender Skala an, wie weit Sie sich Ihrem Ziel angenähert haben:
In den nächsten 4 Wochen möchte ich folgende Ziele erreichen:		
1.	*in der 6. Sitzung*	1-2-3-4-5-6-7-8-9-10 gar nicht … vollkommen
2.	*in der 6. Sitzung*	1-2-3-4-5-6-7-8-9-10 gar nicht … vollkommen
3.	*in der 6. Sitzung*	1-2-3-4-5-6-7-8-9-10 gar nicht … vollkommen
In den nächsten 2 Monaten möchte ich folgende Ziele erreichen:		
4.	*im Verlauf von 2 Monaten*	1-2-3-4-5-6-7-8-9-10 gar nicht … vollkommen
5.	*im Verlauf von 2 Monaten*	1-2-3-4-5-6-7-8-9-10 gar nicht … vollkommen
In den nächsten 6 Monaten möchte ich folgende Ziele erreichen:		
6.	*im Verlauf von 6 Monaten*	1-2-3-4-5-6-7-8-9-10 gar nicht … vollkommen
7.	*im Verlauf von 6 Monaten*	1-2-3-4-5-6-7-8-9-10 gar nicht … vollkommen

ARBEITSBLATT 6 BEISPIEL

PROTOKOLL POSITIVER AKTIVITÄTEN UND TÄGLICHER STIMMUNG

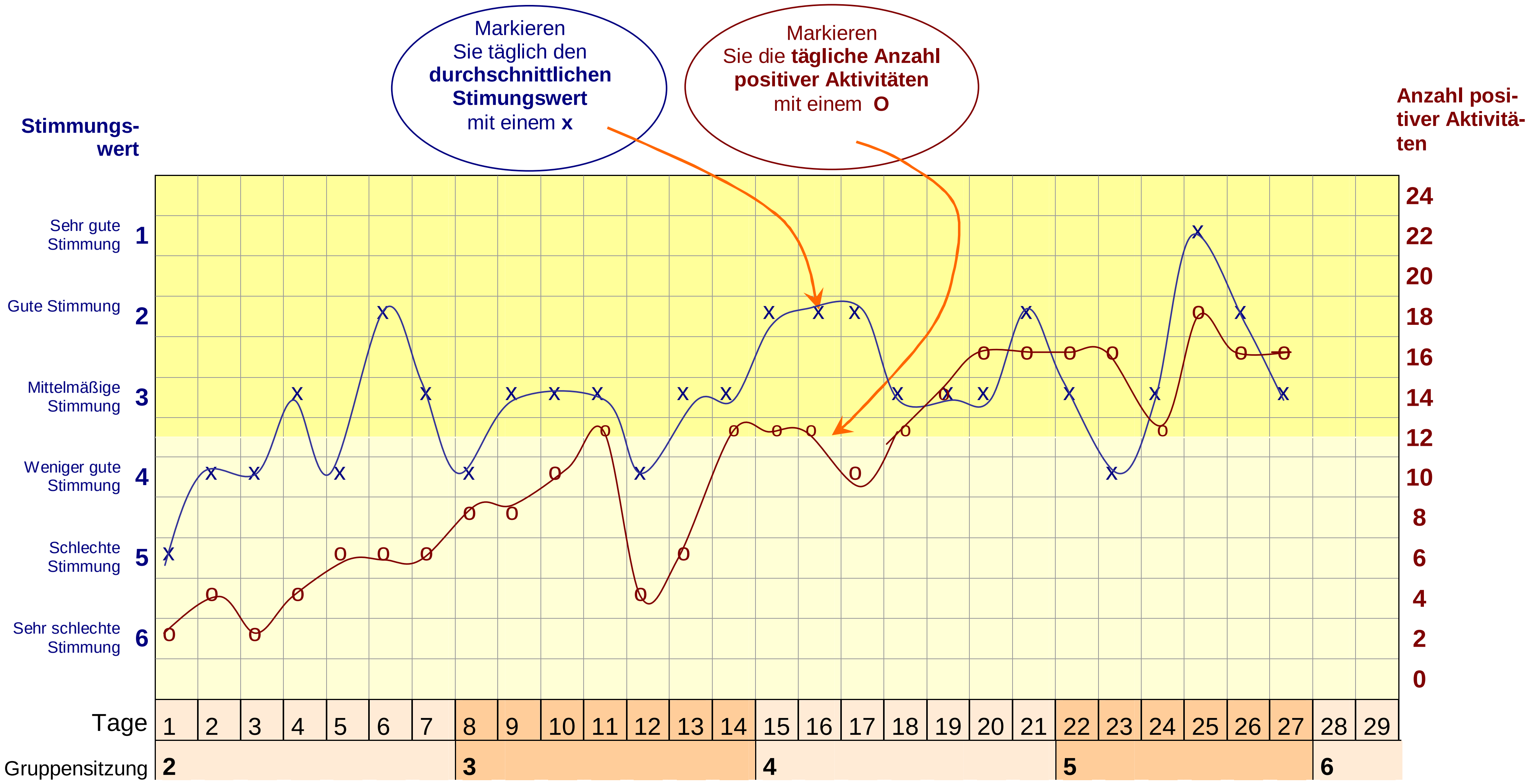

ARBEITSBLATT 6
PROTOKOLL POSITIVER AKTIVITÄTEN UND TÄGLICHER STIMMUNG (TAG 1-30)

Markieren Sie täglich den **durchschnittlichen Stimmungswert** mit einem **x**
Markieren Sie die **tägliche Anzahl positiver Aktivitäten** mit einem **O**

Stimmungs-wert

Sehr gute Stimmung **1**
Gute Stimmung **2**
Mittelmäßige Stimmung **3**
Weniger gute Stimmung **4**
Schlechte Stimmung **5**
Sehr schlechte Stimmung **6**

Anzahl positiver Aktivitäten

24
22
20
18
16
14
12
10
8
6
4
2
0

Tage	30	31	32	33	34	35	36	37	38	39	40	41	42	43	44	45	46	47	48	49	50	51	52	53	54	55	56	57	58	59
Gruppensitzung	**2**							**3**							**4**							**5**							**6**	

ARBEITSBLATT 6

PROTOKOLL POSITIVER AKTIVITÄTEN UND TÄGLICHER STIMMUNG (TAG 31-58)

Markieren Sie den **täglichen Stimmungswert** mit einem **x**

Markieren Sie die **tägliche Anzahl positiver Aktivitäten** mit einem **O**

Stimmungs-wert

Anzahl positiver Aktivitäten

Stimmungswert		Anzahl positiver Aktivitäten
		24
Sehr gute Stimmung	1	22
		20
Gute Stimmung	2	18
		16
Mittelmäßige Stimmung	3	14
		12
Weniger gute Stimmung	4	10
		8
Schlechte Stimmung	5	6
		4
Sehr schlechte Stimmung	6	2
		0

Tage	60	61	62	63	64	65	66	67	68	69	70	71	72	73	74	75	76	77	78	79	80	81	82	83	84	85	86	87	
Gruppensitzung						**7**							**8**								**9**							**10**	

Zu Sitzung 3

1. Wie beeinflussen sich Diabetes und Stimmung?

Wie schon in den ersten Sitzungen deutlich wurde, scheint es einen Zusammenhang zwischen Depressionen und Diabetes zu geben. Die Auftretenshäufigkeit von Depressionen bei Diabetikern ist doppelt so hoch wie in der Allgemeinbevölkerung. Wir wollen uns diesen Zusammenhang einmal genauer anschauen.
Wenn wir den Diabetes in das aus Sitzung 2 bekannte Entstehungsmodell einfügen, könnte die Diabeteserkrankung zum einen eine Rolle als auslösender Faktor bekommen. Die Diabetesdiagnose bedeutet für die meisten Menschen Veränderung und Umbruch im Leben. Diabeteseinstellungen, häufige Arztbesuche, evtl. ein Klinikaufenthalt, Schulungen und Fehlversuche bei der Diabeteskontrolle sind Ihnen sicherlich in Erinnerung. Meist kommen ein veränderter Alltag, Ängste vor Folgeerkrankungen, sowie die Enttäuschung chronisch erkrankt zu sein hinzu. All dies bedeutet Stress und kann zu Überforderung führen. Damit kann der Diabetes zu einem auslösenden Risikofaktor bei der Entstehung einer depressiven Erkrankung werden.

Nun kann es aber auch sein, dass die Depression schon vor der Diabetesdiagnose da war, oder aber die Depression sich ungünstig auf den Diabetes auswirkt. Wie in der vergangenen Sitzung beschrieben, geht eine depressive Symptomatik mit Interessenlosigkeit und Antriebsmangel einher. Vielleicht kennen Sie den Gedanken “Das hat doch alles keinen Sinn!” oder “Mir wird alles zu viel, ich schaffe es sowieso nicht!”? Depressive Stimmung kann dazu führen, dass man es aufgibt, die Blutzuckerwerte zu kontrollieren, da man meint, dass es sowieso nichts nützt. Oder die Gesundheit wird einem egal, weil “das ganze Leben ja keinen Sinn hat”. Dann ernährt man sich meist auch nicht gesund, sondern isst das, was schnell geht oder was einfach zu besorgen ist. Man kann sich nur schwer aufraffen, etwas zu tun. Körperliche Bewegung empfindet man meist als zu anstrengend. Wenn dies über eine lange Zeit so geht, kann das die Entstehung eines Typ 2 Diabetes fördern, denn wenig Bewegung und ungünstiges Essverhalten sind Risikofaktoren für die Entstehung eines Typ 2 Diabetes. Andererseits kann die Depression bei einer bekannten Diabeteserkrankung dazu führen,

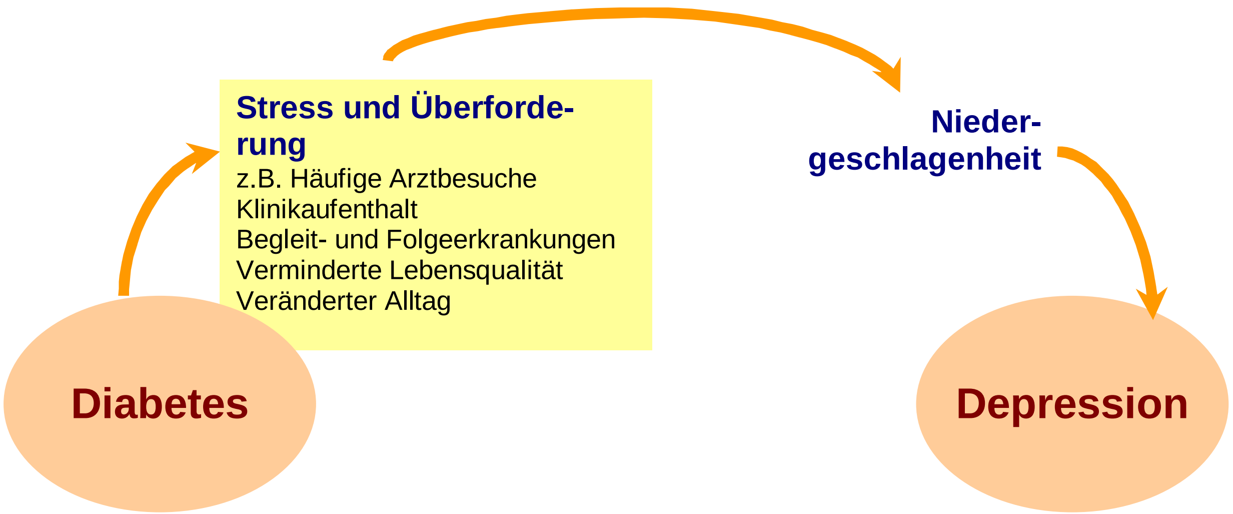

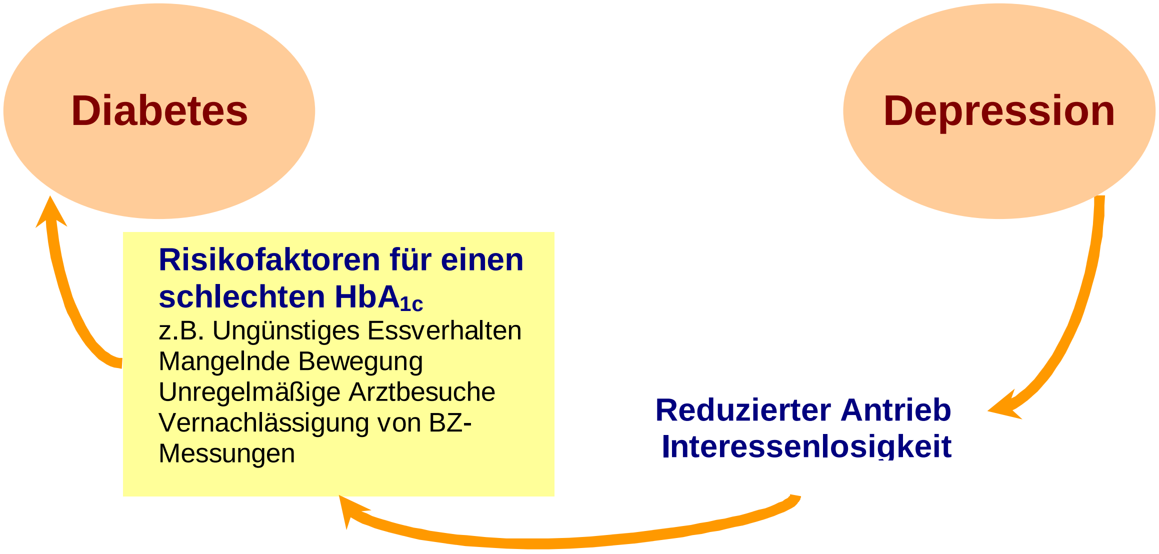

dass man den Empfehlungen des Arztes nicht nachkommt und, obwohl man weiß, was gut für die Diabeteseinstellung und damit für die eigene Gesundheit wäre, es nicht umsetzt.

1.1 Wechselwirkung von Diabetes und Depression

Zwischen den beiden Erkrankungen scheint ebenfalls eine körperliche Wechselwirkung zu bestehen. Dabei geht es um hormonelle Prozesse, die jedoch nicht besonders gut erforscht sind.

Zusammenfassend kann man also feststellen, dass sich Diabeteserkrankung und Depression gegenseitig ungünstig bedingen und sich verstärken können. Dementsprechend ist es wichtig, dass einerseits die depressive Symptomatik verbessert wird und diese Verbesserung auch die Stoffwechseleinstellung begünstigt. Andererseits soll aber auch der Diabetes im Auge bleiben, so dass Stress und Überforderung durch den Diabetes reduziert werden und somit auch eine positive Wirkung auf die depressive Erkrankung entsteht. Genau darauf zielt unser Gruppenprogramm ab.

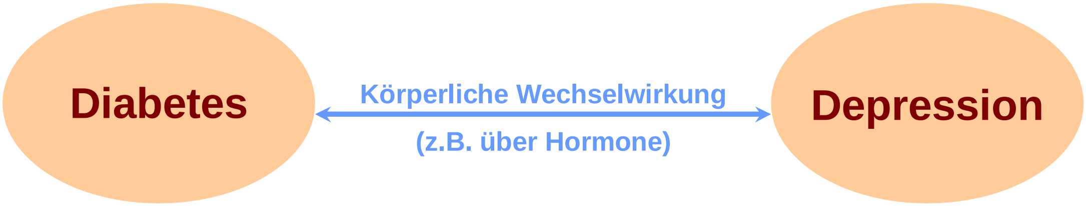

2. Wie beeinflussen sich Gedanken und Stimmung?

Gedanken, Handlungen und Gefühle beeinflussen sich wie in Sitzung 2 dargestellt gegenseitig. Wir werden in dieser Sitzung bei den Gedanken ansetzten und Möglichkeiten ausprobieren, wie Gedanken eine günstige Wirkung auf Ihre Stimmung ausüben können.
Gedanken haben einen beträchtlichen Einfluss auf unsere Stimmung. Meist sind es die Gedanken, die bestimmen, ob wir uns z.B. traurig, ängstlich oder ärgerlich fühlen.

Zur Verdeutlichung dessen, machen wir eine ***kleine Übung***:

Versuchen Sie sich einmal an ein sehr peinliches Ereignis aus Ihrem Leben zu erinnern! Es kann schon weit zurückliegen, oder aber gerade erst passiert sein. Führen Sie sich die Situation noch einmal kurz vor Augen!

Wahrscheinlich fühlen Sie sich in diesem Moment peinlich berührt. Vielleicht erröten Sie sogar. Obwohl gerade gar nichts Peinliches passiert. Nur der Gedanke an diese Situation löst das Gefühl aus.

Nun stellen Sie sich einmal folgende Situation vor:

Sie würden Ihren Blutzucker messen und hätten einen Wert von 240 mg/dl. Ein typischer Gedanke in dieser Situation könnte sein: “Schon wieder zu hoch. Ich hätte das Stück Kuchen nicht essen sollen. Um gute Werte zu haben muss ich auf alles Gute verzichten.“ In diesem Moment träten vielleicht Schuldgefühle auf. Möglicherweise würden Sie sich verzweifelt und hoffnungslos fühlen. Um das ganze übersichtlich zu gestalten, bringen wir diese Abfolge von Situation, den dabei aufkommenden Gedanken und dem daraus resultierenden Gefühl einmal in Tabellenform:

Situation	→	Gedanken	→	Gefühl
Messe den Blutzucker und habe einen Wert von 240 mg/dl.	→	„Schon wieder zu hoch. Ich hätte das Stück Kuchen nicht essen sollen. Um gute Werte zu haben muss ich auf alles Gute verzichten.“	→	Schuldgefühle<br><br>Verzweifelt<br>Hoffnungslos<br><br>Lebensüberdruss

**Situation**		**Gedanken**		**Gefühl**
Liege morgens im Bett und denke über all das nach, was an dem Tag zu erledigen ist	→	„Wie soll ich das nur schaffen? Das kriege ich nie hin.“	→	Niedergeschlagen<br>Hoffnungslos<br>Deprimiert

Deutlich wird auch beim oben aufgeführten Beispiel, dass die Gedanken das nachfolgende Gefühl auslösen.
Demnach sind die bewertenden Gedanken, die scheinbar automatisch entstehen, in den o.g. Beispielen nicht besonders hilfreich, was die Verbesserung der Stimmung betrifft. Eher im Gegenteil, sie verstärken die depressive Stimmung, machen traurig und passiv. Gerade dann, wenn Menschen sich niedergeschlagen oder depressiv fühlen neigen sie dazu automatische, ungünstige Gedanken zu generieren. Manchmal verzerren diese Gedanken den Eindruck in eine negative Richtung oder sind übertrieben. Auf dem Arbeitsblatt 8 sind typische Gedanken, die einem das Leben erschweren können, aufgelistet.

2.1 Ungünstige Gedanken verändern

Wir haben festgestellt, dass sich insbesondere in depressiver Stimmungslage Gedanken und Stimmung ungünstig beeinflussen. Wir wollen nun eine Möglichkeit aufzeigen, wie Sie unangemessene Gedanken so verändern können, dass sie Ihnen helfen, Ihre Therapieziele zu erreichen und Ihre Stimmung zu verbessern.

Dazu sind die **drei** folgenden **Schritte** (s. auch Arbeitsblatt 7) sinnvoll:

1. **Formulieren Sie den negativen Gedanken** als Aussage, wenn möglich nicht als Frage. Notieren Sie ihn.

2. **Begründen Sie**, **ob** der Gedanke **zutreffend** ist. Nur wenn Sie diese Frage nicht eindeutig beantworten können, fragen Sie sich stattdessen inwiefern der Gedanke **hilfreich** für Ihr Wohlbefinden ist.
   a) Ist der Gedanke zutreffend/ hilfreich, handelt es sich um einen angemessenen Gedanken, der nicht verändert werden muss!
   b) Der Gedanke ist nicht zutreffend oder aber nicht hilfreich: Gehen Sie zu Schritt 3.

3. **Formulieren Sie einen alternativen Gedanken** der in dieser Situation hilfreicher oder angemessener wäre.

2.2 Angemessene Gedanken finden

Bei der Formulierung alternativer Gedanken, geht es nicht darum eine “rosa Brille aufzusetzen”, also nur noch positiv zu denken und dabei Gefahr zu laufen, die Realität zu verkennen. Vielmehr sollen Gedanken entstehen, die den Umständen der Situation angemessen sind, die zu konstruktiven Lösungen führen, die eventuell motivieren und helfen.
Für die zuvor genannten Beispiele kann man ebenfalls verschiedene angemessene Gedanken finden:

Situation	Negative Gedanken	Gefühl	Alternative Gedanken	Alternatives Gefühl
Messe den Blutzucker und habe einen Wert von 240 mg/dl.	„Schon wieder zu hoch. Ich hätte das Stück Kuchen nicht essen sollen. Um gute Werte zu haben muss ich auf alles Gute verzichten.“ →	Schuldgefühle<br><br>Verzweifelt<br>Hoffnungslos<br><br>Lebens-überdruss	“Ich könnte ein wenig spazieren gehen, dann bessern sich die Werte.”<br>“Ich spritze Insulin nach, dann wird das schon besser.” →	Erleichterung<br><br>Ein bisschen hoffnungsfroh<br><br>Weniger Schuldgefühle

Situation	Negative Gedanken	Gefühl	Alternative Gedanken	Alternatives Gefühl
Liege morgens im Bett und denke über all das nach, was an dem Tag zu erledigen ist.	„Wie soll ich das nur schaffen? Das kriege ich nie hin.“ →	Niedergeschlagen<br><br>Hoffnungslos<br><br>Deprimiert	“Früher habe ich das auch geschafft. “<br>“Eins nach dem anderen.” →	Nicht mehr ganz so hoffnungslos<br><br>Ein wenig zuversichtlich

Persönliche Notizen zu dieser Sitzung

Übungsaufgaben der 3. Sitzung:

1. Versuchen Sie **eigene wiederkehrende ungünstige Gedanken** zu **verändern**:
   Nutzen Sie dazu die drei Schritte auf dem Arbeitsblatt 7.
   Als Hilfestellung zum Erkennen ungünstiger Gedanken dient Ihnen das Arbeitsblatt 8.

ARBEITSBLATT 7
3 SCHRITTE ZUR VERÄNDERUNG NEGATIVER, UNANGEMESSENER GEDANKEN

1. Negativer automatischer Gedanke (als Aussage formulieren, keine Fragen)

2. Ist der Gedanke zutreffend / hilfreich? (Begründung)

Wenn ja: = ist kein irrationaler Gedanke
Wenn nein: ⇓

3. Was wäre ein hilfreicher, angemessener Gedanke?

ARBEITSBLATT 7
3 SCHRITTE ZUR VERÄNDERUNG NEGATIVER, UNANGEMESSENER GEDANKEN

1. Negativer automatischer Gedanke (als Aussage formulieren, keine Fragen)

2. Ist der Gedanke zutreffend / hilfreich? (Begründung)

Wenn ja: = ist kein irrationaler Gedanke
Wenn nein: ⇓

3. Was wäre ein hilfreicher, angemessener Gedanke?

ARBEITSBLATT 7
3 SCHRITTE ZUR VERÄNDERUNG NEGATIVER, UNANGEMESSENER GEDANKEN

1. Negativer automatischer Gedanke (als Aussage formulieren, keine Fragen)

2. Ist der Gedanke zutreffend / hilfreich? (Begründung)

Wenn ja: = ist kein irrationaler Gedanke
Wenn nein: ⇓

3. Was wäre ein hilfreicher, angemessener Gedanke?

ARBEITSBLATT 7
3 SCHRITTE ZUR VERÄNDERUNG NEGATIVER, UNANGEMESSENER GEDANKEN

1. Negativer automatischer Gedanke (als Aussage formulieren, keine Fragen)

2. Ist der Gedanke zutreffend / hilfreich? (Begründung)

Wenn ja: = ist kein irrationaler Gedanke
Wenn nein: ⇓

3. Was wäre ein hilfreicher, angemessener Gedanke?

ARBEITSBLATT 7 KOPIERVORLAGE

3 SCHRITTE ZUR VERÄNDERUNG NEGATIVER, UNANGEMESSENER GEDANKEN

1. Negativer automatischer Gedanke (als Aussage formulieren, keine Fragen)

2. Ist der Gedanke zutreffend / hilfreich? (Begründung)

Wenn ja: = ist kein irrationaler Gedanke
Wenn nein: ⇓

3. Was wäre ein hilfreicher, angemessener Gedanke?

ARBEITSBLATT 8
UNGÜNSTIGE EINSTELLUNGEN

Einige Einstellungen und Annahmen,
mit denen Menschen sich das Leben schwer machen können

1. Wenn ich nicht alles perfekt mache, bin ich ein Versager.
2. Jemanden um Hilfe zu bitten ist ein Zeichen von Schwäche.
3. Ich muss unbedingt von allen Menschen, die mir etwas bedeuten, geliebt und akzeptiert werden.
4. Es ist besser, gar nichts zu tun, als etwas zu versuchen, was fehlschlagen könnte.
5. Wenn mir ein Fehler unterläuft, dann ist das furchtbar und katastrophal.
6. Es wäre unerträglich, als unzulänglich bloßgestellt zu werden.
7. Ich sollte unangenehme Situationen um jeden Preis vermeiden.
8. Wenn ich an mich denke oder etwas für mich tue, bin ich ein Egoist.
9. Nur wenn ich absolut sicher bin, kann ich Entscheidungen treffen.
10. Ich bin für alles verantwortlich.
11. Wenn man Problemen und unangenehmen Situationen aus dem Weg geht, verschwinden sie mit der Zeit von selbst.
12. Es gibt immer eine perfekte Lösung.
13. Ich darf niemandem wehtun.
14. Man kann sich auf niemanden verlassen.

Zu Sitzung 4

1. Wie werden depressive Stimmungen aufrechterhalten?

1.1 Wie können Hindernisse die Umsetzung der Therapieziele erschweren?

Therapieziele, wie z.B. positive Aktivitäten ausführen oder bestimmte Vorkehrungen wegen des Diabetes zu treffen, sind eventuell gar nicht so einfach durchzuführen. Vielleicht haben Sie auch bei den Zielen, die Sie in der 2. Sitzung formuliert haben, Hindernisse entdeckt. Vielleicht sind Schwierigkeiten aufgetaucht, die Ihnen die Umsetzung der ersten Schritte erschweren oder gar als unmöglich erscheinen lassen.

Vielleicht wird die Zielerreichung auch durch die Symptome oder durch aufrechterhaltende Faktoren Ihrer Depression erschwert. Über solche Hindernisse zu stolpern ist sicherlich unangenehm, vielleicht auch entmutigend. Besonders dann, wenn Menschen in depressiver Stimmung sind, geben sie schneller auf.

Wir wollen uns deswegen einmal ganz konkret mit Schwierigkeiten, die bei der Umsetzung Ihrer Therapieziele aufgekommen sind, auseinandersetzen.

Hier haben Sie die Gelegenheit, die **Therapieziele, bei denen Schwierigkeiten aufgetaucht sind**, zu benennen:

....

....

....

....

....

....

....

....

....

....

....

1.2 Wie können Sie Hindernisse überwinden?

Zum Bearbeiten von Hindernissen hat sich folgendes Vorgehen als sehr hilfreich bewährt:

6 Stufen zur Problemlösung

1. **Formulieren Sie möglichst konkret Ziel und Hindernis**. Versuchen Sie die Situation, die schwierig war, genau zu beschreiben.
2. **Notieren Sie alle (!) Lösungsvorschläge**, die Ihnen spontan einfallen, ohne sie zu bewerten. Vielleicht können Sie auch andere Teilnehmer oder eine Vertrauensperson bitten, mitzudenken!
3. **Bewerten Sie die Lösungsvorschläge**, indem Sie festlegen, welcher Ihnen am besten gefällt. Sie können die Vorschläge z.B. in eine Rangreihe bringen, indem Sie entsprechende Platznummern hinter den Vorschlag schreiben. Platz 1 gewinnt!
4. **Planen Sie den nächsten Schritt**, den die Umsetzung dieses Vorschlags erfordert. Hilfreiche Fragen dazu sind: Was ist das nächste, was ich tun könnte? Wie kann das genau aussehen?
5. **Führen Sie den geplanten Schritt durch** und planen Sie anschließend den darauf folgenden (diesen Vorgang wiederholen Sie solange, bis sie ihr Ziel erreicht haben).
6. **Bewerten Sie Ihren Erfolg**. Nutzen Sie dazu die Vorgaben auf dem Arbeitsblatt 9.

Persönliche Notizen zu dieser Sitzung

Welche Vorschläge oder Anmerkungen sind Ihnen aufgefallen, die Ihnen wichtig oder hilfreich erscheinen?

....

....

....

....

....

....

....

....

....

Persönliche Notizen zu dieser Sitzung

....

....

....

....

....

....

....

....

....

....

Übungsaufgaben der 4. Sitzung:

Versuchen Sie mithilfe der oben beschriebenen 6 Stufen zur Problemlösung ein **Hindernis**, das Sie von der Erreichung Ihrer Ziele fern hält, zu **bearbeiten**. Wir empfehlen Ihnen dazu das Arbeitsblatt 9.

ARBEITSBLATT 9
6 STUFEN DER PROBLEMLÖSUNG

Wie können Sie Hindernisse überwinden?

Stufe 1

Formulieren Sie möglichst konkret Ziel und Hindernis:

Stufe 2

Notieren Sie alle (!) Lösungsvorschläge (nutzen Sie ggf. die Rückseite des Blattes):

Stufe 3

Bewerten Sie die Lösungsvorschläge! Vergeben Sie Rangplätze und markieren sie sich den, der Ihnen am besten gefällt!

Stufe 4

Was ist der nächste Schritt?

Stufe 5

Führen Sie den geplanten Schritt aus und bewerten Sie das Ergebnis in Stufe 6.

Stufe 6

Wie weit habe ich mich meinem Ziel angenähert?

Kreuzen Sie an: 0 – 1 – 2 – 3 – 4 – 5 – 6 – 7 – 8 – 9 – 10

gar nicht ... vollkommen

Wie zufrieden bin ich mit der bisherigen Umsetzung meines Ziels?

Kreuzen Sie an: 0 – 1 – 2 – 3 – 4 – 5 – 6 – 7 – 8 – 9 – 10

gar nicht ... vollkommen

Was hat mir bisher geholfen?

ARBEITSBLATT 8
6 STUFEN DER PROBLEMLÖSUNG

Wie können Sie Hindernisse überwinden?

Stufe 1

Formulieren Sie möglichst konkret Ziel und Hindernis:

Stufe 2

Notieren Sie alle (!) Lösungsvorschläge (nutzen Sie ggf. die Rückseite des Blattes):

Stufe 3

Bewerten Sie die Lösungsvorschläge! Vergeben Sie Rangplätze und markieren sie sich den, der Ihnen am besten gefällt!

Stufe 4

Was ist der nächste Schritt?

Stufe 5

Führen Sie den geplanten Schritt aus und bewerten Sie das Ergebnis in Stufe 6.

Stufe 6

Wie weit habe ich mich meinem Ziel angenähert?

Kreuzen Sie an: 0 – 1 – 2 – 3 – 4 – 5 – 6 – 7 – 8 – 9 – 10

gar nicht ... vollkommen

Wie zufrieden bin ich mit der bisherigen Umsetzung meines Ziels?

Kreuzen Sie an: 0 – 1 – 2 – 3 – 4 – 5 – 6 – 7 – 8 – 9 – 10

gar nicht ... vollkommen

Was hat mir bisher geholfen?

Zu Sitzung 5

1. Wie wird depressive Stimmung aufrechterhalten?

1.1 Wie können ungünstige Gedanken die Problemlösung behindern?

Wir haben uns in der 3. Sitzung mit dem Zusammenhang von Gedanken und Gefühlen beschäftigt. Wir haben festgestellt, dass negative Gedanken zu negativen Gefühlen führen. Wir haben gesehen, dass die Suche nach alternativen Gedanken manchmal gar nicht leicht ist. Wenn man aber alternative, positivere Gedanken hat, dann ändern sich meist auch die Gefühle in eine positivere Richtung.

In der 4. Sitzung haben wir festgestellt, dass Hindernisse immer wieder auftauchen können und dass sie häufig entmutigend sind. Häufig sind negative Gedanken, mit denen missglückte Situationen automatisch bewerten werden, verantwortlich für die nachfolgende schlechte Stimmung und Mutlosigkeit. Jeder kennt Gedanken wie "Jetzt reicht es aber, ich habe keine Lust mehr!", "Das wird sowieso wieder unangenehm!" oder "Jetzt lohnt es sich auch nicht mehr, los zu gehen!". Solche Gedanken erschweren Ihnen vielleicht, auftretende Hindernisse zu überwinden. Statt Schwierigkeiten zu lösen, verleiten sie, vorschnell aufzugeben.

1.2 Welche hilfreichen Gedanken und Einstellungen können bei der Problemlösung helfen?

Haben Sie eigene ungünstige Gedanken in einer schwierigen Situation bemerkt? Wenn Sie sich nicht sicher sind, können Sie das Arbeitsblatt 7 zur Hilfe nehmen und negative Gedanken identifizieren. Im Anschluss empfehlen wir Ihnen auch alternative, hilfreiche Gedanken zu formulieren. Wenn Sie diese z.B. auf Karteikarten schreiben, können Sie sie bei sich tragen und sich selbst in einer ähnlichen Situation mit Ihrem hilfreichen Gedanken ermutigen.

Hier haben Sie Platz, Gedanken, die Sie beim Auftauchen von Hindernissen als **hilfreich und nützlich** betrachten, fest zu halten:

....

....

....

....

....

....

....

....

....

Persönliche Notizen zu dieser Sitzung

Übungsaufgaben der 5. Sitzung:

Bearbeiten Sie zur **Problemlösung** mit hilfreichen Gedanken das Arbeitsblatt 10. Sie können zur **Veränderung der ungünstigen Gedanken** Arbeitsblatt 7 zur Hilfe nehmen.
Wir empfehlen Ihnen, Ihre **Karteikarten** als Stütze vor oder in der entsprechenden Situation zu nutzen!

ARBEITSBLATT 10
6 STUFEN DER PROBLEMLÖSUNG MIT BERÜCKSICHTIGUNG NEGATIVER GEDANKEN

Wie können Sie Hindernisse überwinden?

Stufe 1

Formulieren Sie möglichst konkret Ziel und Hindernis:

Welche negativen, ungünstigen Gedanken sind dabei aufgetreten?

Stufe 2

Notieren Sie alle (!) Lösungsvorschläge (nutzen Sie ggf. die Rückseite des Blattes):

Stufe 3

Bewerten Sie die Lösungsvorschläge! Vergeben Sie Rangplätze und markieren sie sich den, der Ihnen am besten gefällt!

Stufe 4

Was ist der nächste Schritt?

Welche alternativen Gedanken können bei der Umsetzung hilfreich sein?

Stufe 5

Führen Sie den geplanten Schritt aus und bewerten Sie das Ergebnis im Stufe 6.

Stufe 6

Wie weit habe ich mich meinem Ziel angenähert?

Kreuzen Sie an: 0 – 1 – 2 – 3 – 4 – 5 – 6 – 7 – 8 – 9 – 10

gar nicht ... vollkommen

Wie zufrieden bin ich mit der bisherigen Umsetzung meines Ziels?

Kreuzen Sie an: 0 – 1 – 2 – 3 – 4 – 5 – 6 – 7 – 8 – 9 – 10

gar nicht ... vollkommen

Was hat mir bisher geholfen?

Welcher Gedanke war besonders hilfreich?

ARBEITSBLATT 10
6 STUFEN DER PROBLEMLÖSUNG MIT BERÜCKSICHTIGUNG NEGATIVER GEDANKEN

Wie können Sie Hindernisse überwinden?

Stufe 1

Formulieren Sie möglichst konkret Ziel und Hindernis:

Welche negativen, ungünstigen Gedanken sind dabei aufgetreten?

Stufe 2

Notieren Sie alle (!) Lösungsvorschläge (nutzen Sie ggf. die Rückseite des Blattes):

Stufe 3

Bewerten Sie die Lösungsvorschläge! Vergeben Sie Rangplätze und markieren sie sich den, der Ihnen am besten gefällt!

Stufe 4

Was ist der nächste Schritt?

Welche alternativen Gedanken können bei der Umsetzung hilfreich sein?

Stufe 5

Führen Sie den geplanten Schritt aus und bewerten Sie das Ergebnis im Stufe 6.

Stufe 6

Wie weit habe ich mich meinem Ziel angenähert?

Kreuzen Sie an: 0 – 1 – 2 – 3 – 4 – 5 – 6 – 7 – 8 – 9 – 10

gar nicht ... vollkommen

Wie zufrieden bin ich mit der bisherigen Umsetzung meines Ziels?

Kreuzen Sie an: 0 – 1 – 2 – 3 – 4 – 5 – 6 – 7 – 8 – 9 – 10

gar nicht ... vollkommen

Was hat mir bisher geholfen?

Welcher Gedanke war besonders hilfreich?

ARBEITSBLATT 7
3 SCHRITTE ZUR VERÄNDERUNG NEGATIVER, UNANGEMESSENER GEDANKEN

1. Negativer automatischer Gedanke (als Aussage formulieren, keine Fragen)

2. Ist der Gedanke zutreffend / hilfreich? (Begründung)

Wenn ja: = ist kein irrationaler Gedanke
Wenn nein: ⇓

3. Was wäre ein hilfreicher, angemessener Gedanke?

Zu Sitzung 6

1. Zwischenbilanz

Sie haben sich in der 2. Sitzung Therapieziele gesetzt, die innerhalb von 4 Wochen erreichbar sein sollten. Nun bietet es sich an, zu überprüfen, wieweit es Ihnen gelungen ist, Ihre Ziele zu erreichen. Sie können sich das Arbeitsblatt 5, auf dem Sie Ihre Vorhaben notiert hatten, zur Hand nehmen. Folgende Fragen sind zur Überprüfung jedes der Ziele hilfreich:

1. **Wie weit habe ich mich meinem Ziel angenähert?**

   0 – 1 – 2 – 3 – 4 – 5 – 6 – 7 – 8 – 9 – 10
   gar nicht vollkommen

2. **Wie zufrieden bin ich mit der Umsetzung meines Ziels?**

   0 – 1 – 2 – 3 – 4 – 5 – 6 – 7 – 8 – 9 – 10
   gar nicht vollkommen

3. **Was hat mir geholfen, meinem Ziel näher zu kommen?**

4. **Möchte ich mein Ziel weiter verfolgen?**

5. **Möchte ich ein neues Ziel formulieren? Wenn ja, welches?**

Falls Sie zu dem Ergebnis kommen, Therapieziele neu zu formulieren, konnen Sie das Arbeitsblatt 5 wiederholt zur Hilfe nehmen. Wenn Sie bemerken, dass Sie hinsichtlich eines Ziels zur Verbesserung Ihrer Stoffwechseleinstellung Ihren Arzt hinzuziehen möchten, vereinbaren Sie möglichst kurzfristig einen Termin bei ihm. Das Arbeitsblatt 4 kann Ihnen bei so einem Arztgespräch behilflich sein.

Persönliche Notizen zur Zwischenbilanz dieser Sitzung

....
....
....
....
....
....
....
....
....
....
....
....
....
....
....

2. Wie können Sie Kraft für die Umsetzung Ihrer Ziele tanken?

Sie haben bisher versucht, sich Ziele zu setzen und diese auch zu verfolgen. Dabei kommen häufig persönliche Bedürfnisse, die mit Genuss zu tun haben, zu kurz. Das zu tun, wonach einem gerade ist, gönnt man sich manchmal gar nicht. Vielleicht ist das, wonach Ihnen gerade ist, nicht zu vereinbaren mit Ihren Therapiezielen. Vielleicht können Sie Erholung gar nicht genießen, weil Sie das schlechte Gewissen plagt. Die Verfolgung Ihrer Ziele bedeutet harte Arbeit. Gerade dann, wenn man hart an sich arbeitet, ist ein Ausgleich wichtig.

Wenn man sich depressiv und niedergeschlagen fühlt, ist es meist schwieriger wahrzunehmen, welche Bedürfnisse man gerade hat, was man in einer Situation braucht, um Kraft zu tanken. Zum anderen fällt es vielen schwer, die Erfüllung solcher Bedürfnisse zu genießen.

Häufig ist es Ruhe oder Erholung, die wir bräuchten, manchmal sind es auch Kleinigkeiten, wie eine aufmunternde Geste, ein tiefer Atemzug oder aber auch eine duftende Tasse Kaffee, die uns seelische Kraft geben würden.

Solche "Kraftspender" kann man nicht immer vorausplanen, manchmal ist es notwendig im Augenblick achtsam zu sein und heraus zu finden, was einem im jeweiligen Moment gut tun würde.

Was können Sie tun, um aufzutanken und Kraft zur Umsetzung Ihrer Ziele zu bekommen? Wenn Sie möchten, können sie Ihre Ideen auf dem Arbeitsblatt 11 notieren.

Übungsaufgaben der 6. Sitzung:

Versuchen Sie, in der nächsten Woche ganz bewusst und geplant Kraft zu tanken!

ARBEITSBLATT 11
KRAFT TANKEN

Wie ich in der nächsten Woche

Kraft tanken möchte...

- ....
  ....
- ....
  ....
- ....
  ....
- ....
  ....
- ....
  ....

ARBEITSBLATT 5
THERAPIEZIELE

Legen Sie bitte fest, welche konkreten Ziele Sie erreichen wollen, bezogen auf die Bewältigung Ihrer Depressionen und den Umgang mit Ihrem Diabetes.

Siehe dazu auch Patientenbuch S. 9-10:

Wie Therapieziele formuliert werden sollten:

1. **Realistisch**
2. **Konkret**
3. **Positiv**
4. **Festgelegter Zeitraum**, indem das Ziel erreicht werden kann.

Beispiel für ein allgemeines Ziel:

- Ich will zufrieden sein im Leben.

Beispiele für daraus abgeleitete konkrete, positive Ziele:

- Ich möchte wieder ein Wochenende an die Nordsee fahren.
- Ich möchte zwei Mal in der Woche eine Stunde spazieren gehen.
- Ich werde drei Mal täglich meinen Blutzucker kontrollieren.

Es hat sich als erfolgreich erwiesen, nicht zu viele Therapieziele auf einmal erreichen zu wollen.
Wir empfehlen Ihnen, mindestens ein Ziel hinsichtlich der Durchführung positiver Aktivitäten und ein Ziel bezüglich der Besserung Ihres HbA_{1c} zu formulieren.

**Datum:** ____________<br>**Meine Therapieziele lauten:**	**Datum der Überprüfung**	**Ziel erreicht?**<br>Kreuzen Sie bitte auf folgender Skala an, wie weit Sie sich Ihrem Ziel angenähert haben:
In den nächsten 4 Wochen möchte ich folgende Ziele erreichen:		
1.	*in der 6. Sitzung*	1-2-3-4-5-6-7-8-9-10<br>gar nicht … vollkommen
2.	*in der 6. Sitzung*	1-2-3-4-5-6-7-8-9-10<br>gar nicht … vollkommen
3.	*in der 6. Sitzung*	1-2-3-4-5-6-7-8-9-10<br>gar nicht … vollkommen
In den nächsten 2 Monaten möchte ich folgende Ziele erreichen:		
4.	*im Verlauf von 2 Monaten*	1-2-3-4-5-6-7-8-9-10<br>gar nicht … vollkommen
5.	*im Verlauf von 2 Monaten*	1-2-3-4-5-6-7-8-9-10<br>gar nicht … vollkommen
In den nächsten 6 Monaten möchte ich folgende Ziele erreichen:		
6.	*im Verlauf von 6 Monaten*	1-2-3-4-5-6-7-8-9-10<br>gar nicht … vollkommen
7.	*im Verlauf von 6 Monaten*	1-2-3-4-5-6-7-8-9-10<br>gar nicht … vollkommen

ARBEITSBLATT 5 KOPIERVORLAGE
THERAPIEZIELE

Legen Sie bitte fest, welche konkreten Ziele Sie erreichen wollen, bezogen auf die Bewältigung Ihrer Depressionen und den Umgang mit Ihrem Diabetes.

Siehe dazu auch Patientenbuch S. 9-10:

Wie Therapieziele formuliert werden sollten:

1. **Realistisch**
2. **Konkret**
3. **Positiv**
4. **Festgelegter Zeitraum**, indem das Ziel erreicht werden kann.

Beispiel für ein allgemeines Ziel:

- Ich will zufrieden sein im Leben.

Beispiele für daraus abgeleitete konkrete, positive Ziele:

- Ich möchte wieder ein Wochenende an die Nordsee fahren.
- Ich möchte zwei Mal in der Woche eine Stunde spazieren gehen.
- Ich werde drei Mal täglich meinen Blutzucker kontrollieren.

Es hat sich als erfolgreich erwiesen, nicht zu viele Therapieziele auf einmal erreichen zu wollen.
Wir empfehlen Ihnen, mindestens ein Ziel hinsichtlich der Durchführung positiver Aktivitäten und ein Ziel bezüglich der Besserung Ihres HbA_{1c} zu formulieren.

**Datum: ____________**<br>**Meine Therapieziele lauten:**	**Datum der Überprüfung**	**Ziel erreicht?**<br>Kreuzen Sie bitte auf folgender Skala an, wie weit Sie sich Ihrem Ziel angenähert haben:
In den nächsten 4 Wochen möchte ich folgende Ziele erreichen:		
1.	*in der 6. Sitzung*	1-2-3-4-5-6-7-8-9-10<br>gar nicht … vollkommen
2.	*in der 6. Sitzung*	1-2-3-4-5-6-7-8-9-10<br>gar nicht … vollkommen
3.	*in der 6. Sitzung*	1-2-3-4-5-6-7-8-9-10<br>gar nicht … vollkommen
In den nächsten 2 Monaten möchte ich folgende Ziele erreichen:		
4.	*im Verlauf von 2 Monaten*	1-2-3-4-5-6-7-8-9-10<br>gar nicht … vollkommen
5.	*im Verlauf von 2 Monaten*	1-2-3-4-5-6-7-8-9-10<br>gar nicht … vollkommen
In den nächsten 6 Monaten möchte ich folgende Ziele erreichen:		
6.	*im Verlauf von 6 Monaten*	1-2-3-4-5-6-7-8-9-10<br>gar nicht … vollkommen
7.	*im Verlauf von 6 Monaten*	1-2-3-4-5-6-7-8-9-10<br>gar nicht … vollkommen

ARBEITSBLATT 4
EMPFEHLUNGEN ZUR SENKUNG DES HbA_{1C}

Patient:________________________ **Datum:** _____________ **ggf. Visit:**____

Vom Arzt auszufüllen:

Folgende Verhaltensweisen könnten Ihnen helfen, Ihre Stoffwechseleinstellung langfristig zu verbessern (= $HbA1_c$ um mindestens 1% zu senken), und dadurch die Wahrscheinlichkeit von Spätschäden durch den Diabetes zu verringern:

A) ____

B) ____

C) ____

Vom Patienten auszufüllen:

Bitte tragen Sie in die Kästchen für jede Empfehlung (A bis C) ihre persönliche Bewertung auf einer Skala von 0-10 ein.
0 = gar nicht wichtig/schwierig bis 10 = maximal wichtig/schwierig

	A	B	C
Wie wichtig ist **mir persönlich** die Umsetzung dieser Empfehlung?			
Wie schwierig erscheint mir die Umsetzung dieser Empfehlung?			

Folgende Lösungsmöglichkeiten zur Umsetzung der Empfehlungen habe **ich mit meinem Arzt** besprochen:

Zu A) ____

Zu B) ____

Zu C) ____

ARBEITSBLATT 4 KOPIERVORLAGE

EMPFEHLUNGEN ZUR SENKUNG DES HbA_{1C}

Patient:________________________ **Datum:** _____________ **ggf. Visit:**____

Vom Arzt auszufüllen:

Folgende Verhaltensweisen könnten Ihnen helfen, Ihre Stoffwechseleinstellung langfristig zu verbessern (= $HbA1_c$ um mindestens 1% zu senken), und dadurch die Wahrscheinlichkeit von Spätschäden durch den Diabetes zu verringern:

A) ____

B) ____

C) ____

Vom Patienten auszufüllen:

Bitte tragen Sie in die Kästchen für jede Empfehlung (A bis C) ihre persönliche Bewertung auf einer Skala von 0-10 ein.
0 = gar nicht wichtig/schwierig bis 10 = maximal wichtig/schwierig

	A	B	C
Wie wichtig ist **mir persönlich** die Umsetzung dieser Empfehlung?			
Wie schwierig erscheint mir die Umsetzung dieser Empfehlung?			

Folgende Lösungsmöglichkeiten zur Umsetzung der Empfehlungen habe **ich mit meinem Arzt** besprochen:

Zu A) ____

Zu B) ____

Zu C) ____

Zu Sitzung 7

1. Schwierige Situationen im Umgang mit anderen Menschen: Bedürfnisse umsetzen

Schwierige Situationen mit anderen Menschen tauchen immer wieder auf, insbesondere dann, wenn man eigene Bedürfnisse umsetzen möchte. Vielleicht ist Ihnen beim Krafttanken oder bei der Umsetzung Ihrer Therapieziele aufgefallen, dass Sie andere Menschen um etwas bitten wollten, sie Wünsche oder Forderungen an Ihre Mitmenschen hatten.
Gerade dann, wenn man eine chronische Erkrankung hat, entstehen solche Situationen. Z.B. ist es nicht immer ganz einfach andere zu bitten, Rücksicht auf einen zu nehmen, da man zunächst noch seinen Blutzucker kontrollieren muss.

Insbesondere in depressiver Stimmung kann es schwierig sein, sich für seine eigenen Belange einzusetzen. Am liebsten würde man sich zurückziehen, der Kontakt mit Anderen wird schnell zu viel. In solch einer Stimmungslage neigen die meisten im Kontakt mit Mitmenschen zu einem **passiven und wenig selbstsicheren Stil**. Merkmale dieses Stils sind Zurückhaltung und Ängstlichkeit, die dazu führen, dass eigene Bedürfnisse und Wünsche gar nicht oder undeutlich geäußert werden. Manchmal reagieren Menschen auch eher gereizt auf andere, wenn sie in einer depressiven Stimmung sind. Dadurch können ebenfalls schwierige Situationen entstehen.

Sich **selbstsicher oder sozial kompetent** zu verhalten bedeutet, eigene Wünsche und Bedürfnisse offen zu äußern, dabei aber auch Wünsche und Bedürfnisse von anderen zu berücksichtigen.

Damit unterscheidet sich dieses Verhalten von einem **aggressiven Stil**, bei dem nur die eigenen Interessen, ohne Rücksicht auf andere, verfolgt werden.

Kriterien für unsicheres, sicheres und aggressives Verhalten sind:

Merkmal	Unsicher	Sicher	Aggressiv
**Stimme**	Leise<br>Zaghaft	Angemessen laut<br>Klar<br>Deutlich	Brüllend<br>Schreiend<br>Impulsiv
**Formulierung**	Unklar oder vage<br>Umständlich<br>“Überhöflich”	Eindeutig<br>Höflich	Drohend<br>Beleidigend<br>Unhöflich
**Inhalt**	Überflüssige Erklärungen<br><br>Verleugnen eigener Bedürfnisse/ Schnelles Nachgeben<br><br>Gebrauch von “man”<br>Gefühle werden nicht oder nur indirekt ausgedrückt	Präzise Begründung<br><br>Ausdrücken eigener Bedürfnisse /Zu Kompromissen bereit<br><br>Gebrauch von “ich”<br>Gefühle werden ausgedrückt	Keine Erklärungen und Begründungen<br><br>Kompromisslosigkeit/ Rechte anderer werden ignoriert<br><br>Drohungen<br><br>Beleidigungen
**Gestik und Mimik**	Kaum vorhanden oder verkrampft<br><br>Kein Blickkontakt	Lebhaft<br><br>Blickkontakt<br><br>Entspannte Körperhaltung	Unkontrolliert, wild gestikulierend<br><br>Kein Blickkontakt oder “anstarrend”<br><br>Drohend

In Anlehnung an: Hintsch, R. & Pfingsten, U. (1983).

Stellen Sie sich vor, Sie säßen abends mit Ihrem Mann oder einem Freund vorm Fernseher. Dieser schaltet ganz selbstverständlich die Live-Übertragung eines Fußballspiels ein. Sie hatten sich schon am Vormittag das Fernsehprogramm durchgelesen und sind dabei auf einen interessanten Film gestoßen, den Sie gerne mit Ihrem Mann/ Freund sehen würden.
Denken Sie einmal kurz darüber nach, wie Sie sich fühlen würden und was Ihnen durch den Kopf gehen würde.

Ein Beispiel für das selbstsichere Äußern dieses Wunsches ist:

“Ich wünsche mir, dass wir heute den Film auf dem anderen Programm sehen. Ich habe in der Fernsehzeitung darüber gelesen, er hört sich sehr interessant an.”

Persönliche Notizen zu dieser Sitzung

....
....
....
....
....
....
....
....
....
....
....
....
....
....
....
....
....
....

Übungsaufgaben der 7. Sitzung:

Versuchen Sie, die in dieser Sitzung erprobten oder besprochenen Verhaltensbeispiele zuhause in der Realität durchzuführen:

- ❖ Versuchen Sie, in zwei Situationen mit anderen Menschen eigene Bedürfnisse umzusetzen.
- ❖ Wir empfehlen Ihnen, die beiden Übungen auf dem Arbeitsblatt 12 zu protokollieren.

ARBEITSBLATT 12
ÜBUNGSPROTOKOLL „EIGENE BEDÜRFNISSE UMSETZEN“

Inhalt der Übung:

Gesprächspartner:

Datum: **Ort:**

Worauf ich bei der Durchführung der Übung **achten wollte**...

- ....
- ....
- ....
- ....
- ....

Meine Stimmung: 1 Sehr gut – 2 gut – 3 mittelmäßig – 4 weniger gut – 5 schlecht – 6 sehr schlecht

Vor der Situation: **Während** der Situation: **Nach** der Situation:

Was ich **gut** gemacht habe...

- ....
- ....
- ....
- ....
- ....

Hilfreiche Gedanken während der Übung waren...

- ....
- ....
- ....
- ....
- ....

Was ich beim nächsten Mal **besser** machen möchte...

- ....
- ....
- ....
- ....
- ....

ARBEITSBLATT 12
ÜBUNGSPROTOKOLL „EIGENE BEDÜRFNISSE VERWIRKLICHEN“

Inhalt der Übung:

Gesprächspartner:
Datum: **Ort:**

Worauf ich bei der Durchführung der Übung **achten wollte**...

- ....
- ....
- ....
- ....
- ....

Meine Stimmung: 1 Sehr gut – 2 gut – 3 mittelmäßig – 4 weniger gut – 5 schlecht – 6 sehr schlecht

Vor der Situation: **Während** der Situation: **Nach** der Situation:

Was ich **gut** gemacht habe...

- ....
- ....
- ....
- ....
- ....

Hilfreiche Gedanken während der Übung waren...

- ....
- ....
- ....
- ....
- ....

Was ich beim nächsten Mal **besser** machen möchte...

- ....
- ....
- ....
- ....
- ....

Zu Sitzung 8

1. Wie beeinflussen sich Ängste vor Folgeerkrankungen, Stimmung und der Umgang mit dem Diabetes?

1.1 Diabetesfolgeerkrankungen

Folgeerkrankungen treten in Abhängigkeit von Dauer und Ausmaß der Blutzuckerhöhung sowie von bestehenden Begleiterkrankungen auf. Man kann sagen, je länger ein Diabetes schlecht oder gar nicht eingestellt ist, desto höher ist das Risiko für das Entstehen von Folgeerkrankungen.

Zu den Folgeerkrankungen des Diabetes zählen vor allem Nierenschäden (Nephropathie), Augenschäden (Retinopathie), Nervenschäden (Neuropathie) und Durchblutungsstörungen mit dem Risiko für ein diabetisches Fußsyndrom und Amputationen. Außerdem entsteht ein erhöhtes Risiko für eine Arteriosklerose, speziell für die koronare Herzkrankheit, Schlaganfall und die periphere arterielle Verschlusskrankheit.

Ist man nun diesen Risiken durch die Diabeteserkrankung ausgesetzt, können Ängste entstehen. Das merken Sie vielleicht dadurch, dass Sie diese Thematik am liebsten aussparen, also vermeiden würden oder daran, dass Sie nervös, unruhig oder angespannt beim Lesen der ersten zwei Absätze dieses Kapitels geworden sind.

1.2 Was ist Angst?

Jeder Mensch hat in seinem Leben schon einmal Angst gehabt. Angst ist demnach etwas natürliches. Schon kleine Kinder haben Angst vor bestimmten Dingen, z.B. vor Höhe. Das ist auch gut so. Stellen Sie sich einmal vor, ein Kind oder auch Sie selbst hätten keine Angst vor Höhe: Sie würden wahrscheinlich leichtsinnig auf Berge klettern und dabei auf Felskanten zugehen. Oder Sie würden auf Fenstersimsen herumturnen, denn Sie hätten ja keine Angst hinunterzufallen. Die Wahrscheinlichkeit, dass Sie allerdings dabei herunterfallen und sich dabei das Genick brechen würden, wäre natürlich trotzdem relativ groß. Angst führt dazu, dass Sie vorsichtig sind und sich bei Gefahr in Acht nehmen. Angst ist eine sinnvolle Reaktion und verhilft letztendlich zu überleben.
Nun kann Angst aber auch so groß werden, dass sie Sie von Dingen oder vor Situationen abhält, die Sie eigentlich tun möchten oder sollten. Man kann z.B. eine solche Angst vor Zahnarztbehandlungen entwickeln, dass man den Arzt nicht mehr aufsucht, am Ende kaputte Zähne und Zahnschmerzen hat. Oder man kann so große Angste vor Menschen entwickeln, dass man nicht mehr einkaufen geht. Solche Ängste sind in den allermeisten Fällen nicht angemessen und beeinträchtigen das Leben und die Lebensqualität immens.

1.3 Angst vor Folgeerkrankungen

Wie wir oben bereits festgestellt haben, ist eine Diabeteserkrankung mit dem Risiko für Folgeerkrankungen behaftet. Ängste in Bezug auf diese Folgeerkrankungen sind nicht unrealistisch, die Risiken sind nicht wegzudiskutieren. Dass sorgenvolle Gedanken auftreten, z.B. wenn der HbA_{1c} einmal ziemlich schlecht war oder erste Anzeichen für Folgeerkrankungen gefunden werden, ist sinnvoll, denn dadurch treffen Sie bestimmte Vorsorge- oder Vor-

sichtsmaßnahmen. Diese Ängste können aber auch so groß werden, dass Ihnen nichts übrig bleibt, als solche Gedanken schnell zu verdrängen und das Thema zu übergehen oder ihre Lebensqualität ist zusätzlich belastet.

Es gibt also verschiedene Möglichkeiten mit Ängsten umzugehen:

1. Nicht weiter drüber nachdenken, sich ablenken.

Vorteil: Zunächst einmal sind Sie erleichtert, es geht Ihnen besser.

Nachteil: Oft genug lassen sich solche Gedanken nicht verscheuchen oder kommen ziemlich schnell wieder, die Realität lässt sich nur schwer verleugnen. Wahrscheinlich wenden Sie wenige oder keine Vorsorgestrategien an.

2. Sich so oft wie möglich Gedanken darüber machen und sich die ganze Zeit sorgen.

Vorteil: Kurzzeitig sind Sie beruhigt, da sie das Gefühl haben sich bestens auf schlimme Zeiten vorzubereiten.

Nachteil: Sie entwickeln kreisende Gedanken und finden keine Lösungsmöglichkeiten, Sie sind angespannt und Ihre Stimmung wird schlechter. Sie gelangen wahrscheinlich zu der Überzeugung, dass es sowieso so kommen wird und dass alles, was Sie tun, nichts daran ändern kann. Auch hier werden Sie möglicherweise keine Vorsorge treffen, da sie glauben, dem Schicksal nicht entrinnen zu können.

3. Automatische, angstauslösende Gedanken zu Ende denken. Überprüfen, wie realistisch der Gedanke und die Angst sind. Eine Lösung für die Möglichkeit finden, dass der Gedanke Realität wird.

Vorteil: Wenn Sie sich angemessen mit den sorgenvollen Gedanken auseinandergesetzt haben und dadurch leichter eine Lösungsmöglichkeit gefunden haben, verliert der Gedanke viel von seiner Bedrohung und sie haben langfristig eine angemessene Portion Angst oder Sorge, die Sie zu Vorsorgemaßnahmen motiviert.

Nachteil: Die konkrete Auseinandersetzung mit einem sorgenvollen Gedanken bedeutet, dass vielleicht kurzfristig mehr Angst entsteht oder es unangenehm ist.

1.4 Gleichgewicht zwischen Angst und Sorglosigkeit

Zuviel Angst oder eine Sorglosigkeit wirken sich ungünstig auf den Umgang mit der Diabeteserkrankung aus. Sinnvoll ist eine “gesunde” Angst, die Sie dazu motiviert, z.B. auf Ihren Stoffwechsel zu achten.

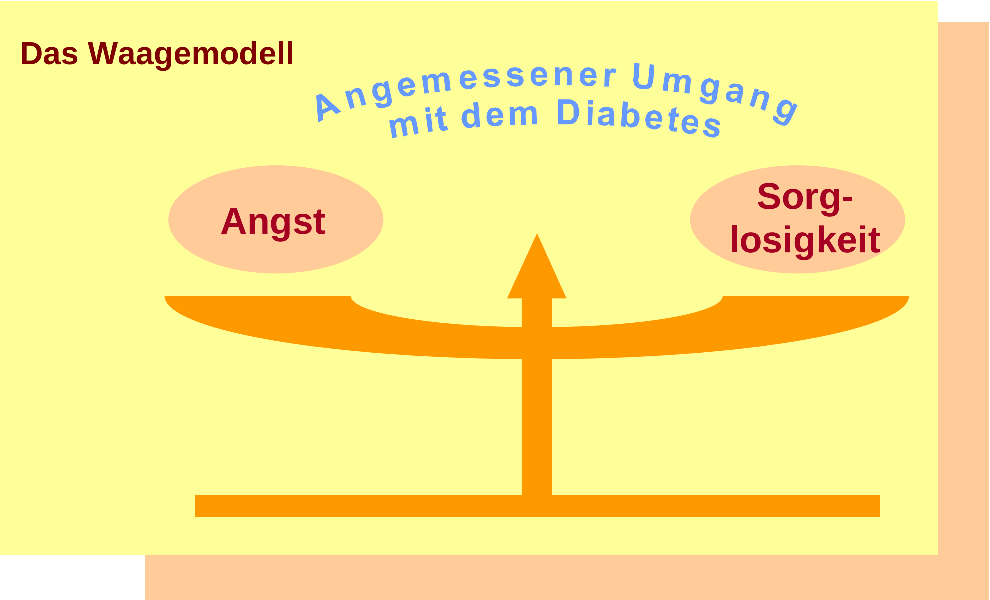

2. Bewältigung der Angst vor Folgeerkrankungen – Wie Sie Waage halten können

Angst entsteht wie in Sitzung 3 beschrieben als Reaktion auf Gedanken. Dabei handelt es sich um negative, sorgenvolle oder katastrophisierende Gedanken. Ebenso wie bei der Verbesserung der depressiven Stimmung ist es auch zur Balancierung der Angst sinnvoll, bei den Gedanken anzusetzen.

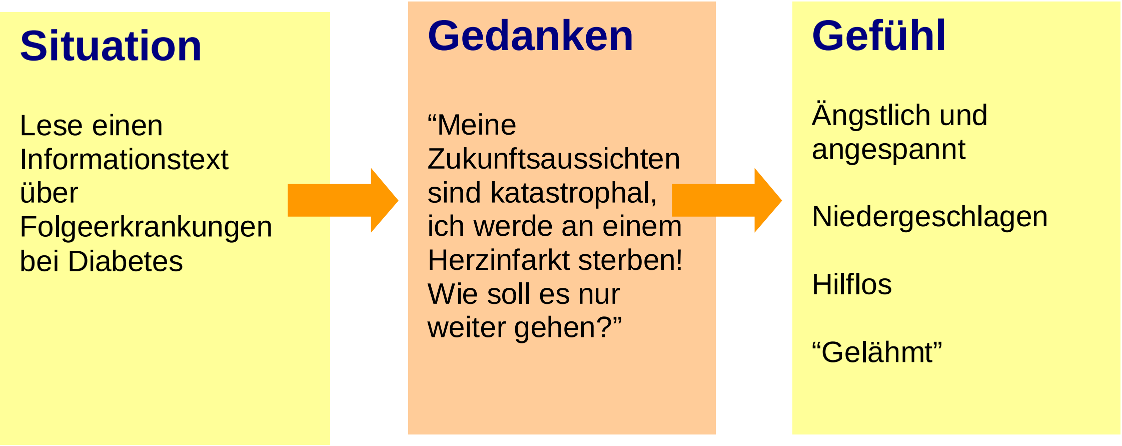

Wir möchten Ihnen verschiedene **Gedankentechniken** vorstellen, die Ihnen dabei helfen sollen die Angst zu reduzieren und in einem motivationsauslösenden Ausmaß zu halten:

Realitätsüberprüfung: Häufig treten, wie auch bei depressiver Stimmung, in Verbindung mit dem Erleben von Angst, automatische Gedanken auf, die wir zunächst einmal als Tatsachen hinnehmen. Bei genauerer Betrachtung fällt auf, das die Gedanken inhaltlich nur Spekulationen, Hypothesen oder eine bestimmte Möglichkeit in der Zukunft darstellen. Da wir es aber gewohnt sind, so zu denken oder aus einer bestimmten Perspektive, in diesem Falle der Angstperspektive, auf die Dinge schauen, fallen uns solche Verzerrungen nicht mehr auf.

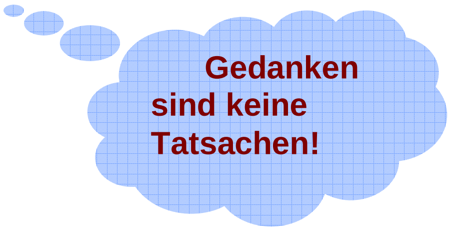

Versuchen Sie folgende Fragen zur Realitätsprüfung eines Gedankens zu beantworten:

- Ist mein Gedanke Tatsache oder Spekulation?
- Wie wahrscheinlich ist, das der Gedanke Wahrheit wird?
- Wie überzeugend ist der Gedanke zwischen 0 und 100%?
- Was spricht für den Gedanken, was spricht dagegen?

Entkatastrophisieren: Ein weiteres Merkmal von angstbezogenen automatischen Gedanken, ist, dass sie häufig katastrophisierende Erwartungen beinhalten.

Versuchen Sie folgende drei Fragen nacheinander zur Entkatastrophisierung eines Gedankens zu beantworten:

1. Was ist das **Schlimmste**, was passieren könnte?
2. Was ist das **Bestmöglichste**, was passieren könnte?
3. Was ist das **Wahrscheinlichste**, was passieren könnte?

Zu-Ende-Denken der Vorstellung: Vielleicht haben Sie selbst schon beobachtet, dass wir häufig Dinge nicht mehr zu Ende denken, da wir der Überzeugung sind zu wissen, was wir damit meinen oder weil es aus Gewohnheit nicht mehr notwendig ist. Ein Gedanke wie „Oh nein, wahrscheinlich ein diabetisches Fußsyndrom" löst bereits Angst und Niedergeschlagenheit aus, Sie fühlen sich angespannt und unwohl, versuchen sich abzulenken. Denn Sie wissen ja, was damit gemeint ist.

Jedoch ist es sinnvoll, sich auch hier einmal Fragen zu stellen, denn mit der konkreten Auseinandersetzung dieser Vorstellung sinkt meist ihre scheinbare Unüberwindbarkeit:

- Wird es schnell passieren (z.B. innerhalb von Tagen oder wenigen Wochen) oder wird einige Zeit vergehen, bis es richtig schlimm ist?
- Was werden Sie in dieser Zeit tun?
- Welche Umstände gehen damit einher?
- Was/wer kann Ihnen helfen, wenn das Schlimmste eintritt?

3-Stufen-Schema zur Veränderung negativer Gedanken: Viele angstauslösende Gedanken sind nicht hilfreich. Wenn Sie extrem katastrophisierend oder realitätsverkennend sind, lösen sie Unruhe oder Passivität aus, das ist nicht hilfreich. Sie können das Arbeitsblatt 7 anwenden, um Ihre Gedanken zu überprüfen und sie in konstruktive, lösungsorientierte Gedanken zu verändern.

Persönliche Notizen zur Gruppenarbeit dieser Sitzung

Was waren auf mich zutreffende katastrophisierende Gedanken und welche waren die für mich sinnvollen, alternativen, hilfreichen Gedanken?

Meine Katastrophengedanken	Meine hilfreichen Gedanken
....	....
....	....
....	....
....	....
....	....
....	....
....	....
....	....
....	....
....	....
....	....

Übungsaufgaben der 8. Sitzung:

Erinnern Sie sich an eine persönliche Situation, in der das Gleichgewicht zwischen Angst und Sorglosigkeit bei Ihnen nicht optimal war.
Notieren Sie sich **drei Gedanken**, die in dieser Situation aufgetreten sind.
Bearbeiten Sie diese anhand des Arbeitsblattes 7 .”3-Stufen-Schema zur Veränderung negativer Gedanken”.

ARBEITSBLATT 7
3 SCHRITTE ZUR VERÄNDERUNG NEGATIVER, UNANGEMESSENER GEDANKEN

1. Negativer automatischer Gedanke (als Aussage formulieren, keine Fragen)

2. Ist der Gedanke zutreffend / hilfreich? (Begründung)

Wenn ja: = ist kein irrationaler Gedanke
Wenn nein: ⇓

3. Was wäre ein hilfreicher, angemessener Gedanke?

ARBEITSBLATT 7
3 SCHRITTE ZUR VERÄNDERUNG NEGATIVER, UNANGEMESSENER GEDANKEN

1. Negativer automatischer Gedanke (als Aussage formulieren, keine Fragen)

2. Ist der Gedanke zutreffend / hilfreich? (Begründung)

Wenn ja: = ist kein irrationaler Gedanke
Wenn nein: ⇓

3. Was wäre ein hilfreicher, angemessener Gedanke?

ARBEITSBLATT 7
3 SCHRITTE ZUR VERÄNDERUNG NEGATIVER, UNANGEMESSENER GEDANKEN

1. Negativer automatischer Gedanke (als Aussage formulieren, keine Fragen)

2. Ist der Gedanke zutreffend / hilfreich? (Begründung)

Wenn ja: = ist kein irrationaler Gedanke
Wenn nein: ⇓

3. Was wäre ein hilfreicher, angemessener Gedanke?

Zu Sitzung 9

In dieser Sitzung haben Sie und Ihre Gruppe das Thema selbst gewählt. Falls es zu dieser Sitzung Unterlagen oder Arbeitsblätter gibt, werden sie Ihnen bei der entsprechenden Gruppensitzung ausgehändigt.

Persönliche Notizen zu dieser Sitzung

....

....

....

....

....

....

....

....

....

....

....

....

....

....

....

....

....

....

Übungsaufgaben der 9. Sitzung:

Bitte benennen Sie Ihre **drei wichtigsten Erkenntnisse**, die Ihnen im Verlauf des gesamten Gruppenprogramms gekommen sind, auf dem Arbeitsblatt 13.

ARBEITSBLATT 13
BEWERTUNG DES GRUPPENTRAININGS

Die 3 wichtigsten Erkenntnisse
dieses Gruppenprogramms
waren für mich...

- ....
  ....
- ....
  ....
- ....
  ....
- ....
  ....
- ....
  ....

Zu Sitzung 10

1. Wie können Sie mit Rückschritten positiv umgehen und ihre Erfolge aufrechterhalten?

1.1 Wie beeinflusst der Umgang mit Rückschritten die Stimmung?

Rückschritte können immer wieder auftauchen. Jedes Ziel, das wir erreichen wollen ist mit Schwierigkeiten verbunden. Seine Depression dauerhaft zu verbessern und den Stoffwechsel langfristig auf einem angemessenen Niveau zu halten, heißt Schwankungen in Kauf zu nehmen. Gerade stressvolle Zeiten, die natürlicherweise vorkommen, begünstigen eine depressive Stimmung. Dennoch ist ein Rückschritt noch kein Rückfall in die Depression. Ein Anstieg des HbA_{1c} muss auch keine dauerhafte Verschlechterung des Stoffwechsels werden. Die Art und Weise wie wir mit Rückschritten umgehen hat eine große Bedeutung für unser anschließendes Wohlbefinden und den weiteren Verlauf einer Erkrankung. Das Erkennen erster Rückschritte und entsprechendes Gegenwirken kann einen Rückfall verhindern. Es ist an jeder Stelle möglich, die depressive Spirale zu unterbrechen. Es lohnt sich auch, wiederholt zu versuchen den HbA_{1c} konstant auf einem angemessenen Niveau zu halten. Lassen Sie sich nicht entmutigen!

Wichtig ist allerdings, sich genau zu überlegen, was wann zu tun ist. Bei einem HbA_{1c} von 7,8% achtsam zu sein und die Selbstkontrolle wieder etwas zu verstärken ist einfacher, als abzuwarten und später dann einen HbA_{1c} von 9% wieder herunter auf 7,5% zu bekommen. Bereits bei beginnender depressiver Stimmung, fühlen wir uns antriebsloser und unsicherer.

Wir können uns am besten schon jetzt auf einen möglichen Rückschritt vorbereiten, so dass es uns dann in solch einer Situation leichter fällt, unsere Ziele wieder aufzunehmen.

1.1 Rückschritt definieren

Zunächst einmal ist es wichtig, zu wissen, woran Sie persönlich einen Rückschritt erkennen würden. Jeder befindet sich in einer speziellen Ausgangslage, definiert individuelle Ziele, so dass auch ein Rückschritt für jeden etwas anderes bedeutet. Überlegen Sie, woran Sie merken würden, dass Sie einen Rückschritt gemacht haben.

Denken Sie bei der Formulierung an die Regeln, die bei der Zielformulierung (Sitzung 2) wichtig waren. So können Sie einen Rückschritt klar umschreiben und ihn dadurch schneller und eindeutiger erkennen.

Beispiele könnten sein:

- Ein Rückschritt für mich ist, wenn ich einen HbA_{1c} größer 7% habe.
- Ein Rückschritt ist für mich, wenn ich 2 mal meinen wöchentlichen Besuch im Fitness-Studio aus Antriebslosigkeit habe ausfallen lassen.

1.2 Der optimale Umgang mit Rückschritten

Sinnvoll ist es, nach Ursachen für geschehene Rückschritte zu suchen. Wir empfehlen die Analyse eines Rückschrittes mithilfe der im rechten Kasten aufgeführten Fragen.
Wir empfehlen Ihnen bereits im Voraus zu planen, was Sie beim Bemerken eines Rückschrittes, diesem entgegensetzen möchten. Vielleicht haben Sie Ideen, was Ihnen helfen könnte, welche der im Gruppenprogramm gelernten Strategien Sie anwenden möchten.

Die Analyse eines Rückschrittes

- Haben Sie sich vielleicht ein zu hohes Ziel gesteckt?
- Haben Sie vielleicht Faktoren, die Sie nicht beeinflussen können, unterschätzt?
- Wäre es vielleicht hilfreich, sich zusätzliche Hilfe von Freunden/ Partnern einzuholen (oder mit meinem Arzt Rücksprache zu halten)?
- Gibt es etwas, das sie anders hätten tun können?

Was möchte ich tun, wenn ich einen Rückschritt feststelle?

Ein Rückschritt hinsichtlich meiner **Depression** ist für mich...	Wenn das auftritt, werde ich dem ... entgegen setzen

Ein Rückschritt hinsichtlich des Umgangs mit meinem **Diabetes** ist für mich...	Wenn das auftritt, werde ich dem ... entgegen setzen

Greifen Sie Ihre Ziele bald wieder auf!

Denken Sie an Ihre Erfolge, die Sie schon erreicht haben!

1.3 Was sollten Sie tun, wenn Ihre Stimmung oder Ihr Antrieb sich über einen längeren Zeitraum (mindestens 2 Wochen) deutlich verschlechtert?

Bemühen Sie sich, diesem Befinden aktiv entgenzutreten, indem Sie sich daran erinnern, was Ihnen früher geholfen hat.
Lesen Sie in diesem Buch nach und wenden Sie die Methoden an, die Ihnen bisher am besten geholfen haben.

Wenn Sie merken, dass sich Ihr Befinden dadurch nicht wieder allmählich bessert, dann sprechen Sie mit Ihrem Arzt darüber. Er wird mit Ihnen besprechen, welche Schritte dann zu unternehmen sind, damit es Ihnen wieder besser geht.

1.4 Wie können Sie mit diesem Programm fortfahren?

Die Gruppensitzungen finden nun nicht mehr statt, Sie können das Programm aber trotzdem weiter fortführen. Wir empfehlen Ihnen, Ihr Persönliches Begleitbuch noch für mindestens 12 Monate weiter zu nutzen!

Sinnvoll ist es, sich nun noch einmal das Arbeitsblatt 5 “Therapieziele” anzuschauen. Sie haben sich dort Ziele notiert, die Sie innerhalb eines zweimonatigen und eines 6monatigen Zeitraumes erreichen wollten. Überprüfen Sie regelmäßig, z.B. alle 2 Wochen, wie weit sie sich dem angenähert haben. Um sich Ihren Zielen weiter anzunähern, ist es sehr wahrscheinlich hilfreich, die Übungen zur Durchführung positiver Aktivitäten, zur Veränderung unangemessener und negativer Gedanken, zur Problemlösung, zur Umsetzung Ihrer Bedürfnisse, zur Balancierung Ihrer Sorge um Folgeerkrankungen zu wiederholen.

Wir empfehlen Ihnen ebenfalls, mithilfe der Aufzeichnungen neue Ziele zu stecken! Eine gute Möglichkeit an der Verbesserung der Stoffwechseleinstellung weiter zu arbeiten, bietet Ihnen das Arbeitsblatt 4. Sie sollten ihre ausgefüllten Arbeitsbeitsblätter zum Thema “Empfehlungen zur Senkung des HbA_{1c}” mit zu jedem Arztbesuch nehmen. So können Sie mit Ihrem Arzt weiter besprechen, welche konkreten Verhaltensweisen er Ihnen empfiehlt.

Gewohnheiten schleichen sich schnell wieder ein. Deswegen lohnt es sich, Ihren Umgang mit dem Diabetes sowie die Depression weiter im Auge zu behalten und sich für eine langfristige Verbesserung weiter zu engagieren!

In einem Jahr wird ein Gespräch mit einem Studienmitarbeiter statt finden. Es wird dann nicht nur um die Messung der Langzeitergebnisse Ihrer Bemühungen gehen, sondern Sie werden außerdem die Möglichkeit haben, sich über Ihre Erfahrung mit dem Persönlichen Begleitbuch auszutauschen. Überlegen Sie sich, was Sie bis dahin tun möchten, um Ihren HbA_{1c} weiter zu senken und Ihre Stimmung weiter zu verbessern.

Wenn Sie möchten können Sie das Gespräch in einem Jahr nutzen, um Schwierigkeiten anzusprechen.

Was ich tun werde, um meinen **HbA_{1c}** innerhalb des nächsten Jahres weiter zu **senken**...

1. ....
....
....

2. ....
....
....

3. ....
....
....

4. ....
....
....

5. ....
....
....

Was ich tun werde, um meine **Stimmung** innerhalb des nächsten Jahres weiter zu **verbessern**...

1. ....
....
....

2. ....
....
....

3. ....
....
....

4. ....
....
....

5. ....
....
....

Wir wünschen Ihnen viel Erfolg und alles Gute!
