# Supplementary material for: Study protocol of the Diabetes and Depression Study (DAD): a multi-center randomized controlled trial to compare the efficacy of a diabetes-specific cognitive behavioral group therapy versus sertraline in patients with major depression and poorly controlled diabetes mellitus
Source: BMC Psychiatry. 2013 Aug 6;13:206. doi: 10.1186/1471-244X-13-206 (PMC3750698; doi:10.1186/1471-244X-13-206)
Supplement: Additional file 5 — ‘Safety manual’ (in German); and ‘Definitions and assessment of adverse events’. [file 1471-244X-13-206-S5.pdf]

Safety Manual:

DAD Trial

Cognitive behavioural therapy vs. sertraline in patients with depression and poorly controlled diabetes mellitus: A randomized controlled trial

Short title: Diabetes and Depression Study (DAD Study)

Eudra CT No: 2005-004525-26

Finale Version 1.0, 20. April 2006

Verantwortlichkeiten:

**Aufgabe**	**Institution**	**Name**
SAE-Meldung	Prüfzentren, Studienzentren	Prüfer, Psychologen
SAE-Registrierung	KKS Mainz	
SAE-Zweitbewertung	Sponsor, LKP	PD Dr. Petrak<br>PD Dr. Müller
Nutzen-Risiko-Bewertung	Sponsor, LKP, Advisory Board	PD Dr. Petrak<br>PD Dr. Müller
Amendment<br>Abbruch der Studie	Sponsor	PD Dr. Petrak
Entblindung von SUSARs	entfällt	
Meldung (SUSAR, Todesfälle)	Sponsor	PD Dr. Petrak
Dokumentation aller SAEs	KKS Mainz	Verantwortlicher Mitarbeiter
Jahresbericht (EK, BfArM)	Sponsor	PD Dr. Petrak
Bericht an Advisory Board	Sponsor	PD Dr. Petrak

Meldewege für SAE und SUSAR – DAD Studie

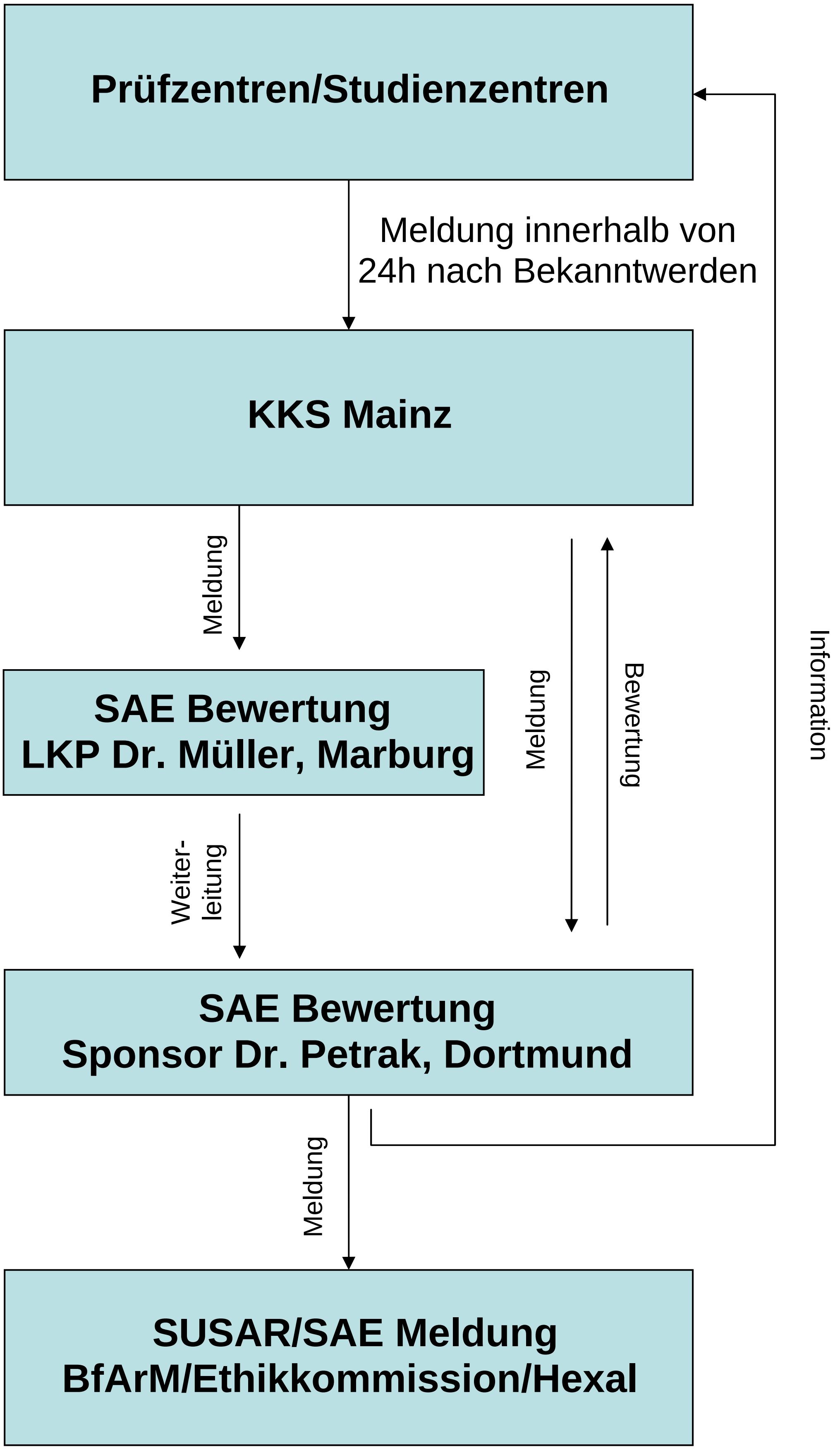

SAE Assessment by LKP and Sponsor

**Protocol Identification**				
Protocol: DAD (Diabetes and Depression Study)				
Protocol Code: DAD-Study		EUDRACT No.: 2005-004525-26		
**SAE Identification**				
Patient (Random) No.:	Site No.:	SAE No.:	Date of onset of event:	
☐ Initial report ☐ Follow-up report<br><br>**SAE (medical term, details see enclosed SAE report):**				

**SAE Assessment**			
	**Serious?**	**Related?**<br>(1=related, 2=probably, 3=possibly, 4=unlikely, 5=not related, 6=not assessable, 0=not assessed)	**Unexpected?**
**Investigator**	☐ yes	I__I	not applicable
**LKP**	☐ yes ☐ no	I__I	☐ yes ☐ no

Review by the sponsor:
Do you agree with the assessment made by LKP? ☐ yes ☐ no
Comment:

Sponsor assessment of risk-benefit ratio:

☐ Safety issue that might alter the current benefit-risk assessment

☐ Safety issue that is sufficient to consider changes in the conduct of the trial

☐ further information will be required

Date (DD/MM/YY) Signature LKP (Dr. Müller)

Date (DD/MM/YY) Signature Sponsor (Dr. Petrak)

PLEASE FAX TO KKS Mainz: FAX No.

EUDRACT Nr.: 2005-004525-26 SAE-BERICHT Seite 1 von 2

PROTOKOLL TITEL: DAD Diabetes –Depressions-Studie
INDIKATION: Depression und schlecht eingestellter Diabetes mellitus
SPONSOR: Dr. rer. soc. Dipl.-Psych. Frank Petrak

Bitte alle Informationen vollständig und wahrheitsgemäß mit schwarzem Kugelschreiber eintragen

PATIENTEN IDENTIFIZIERUNG

Patienten Nr. (Random Nr.)	Geschlecht: ☐ M ☐ W	Alter (Jahre):	Geburtsdatum:	SAE Nr.:

INFORMATIONEN ZUM SAE-BERICHT

☐ INITIAL Bericht	Name des Prüfarztes:	
Datum:	Zentrums Nr.:	Land: Deutschland
☐ FOLLOW-UP Bericht	Institution:	Telefon Nr.:
Datum:	E-mail:	FAX Nr.:

Schweregrad und Grund des SAE

☐ Tod
☐ führt zu bleibenden/signifikanten Schäden/Behinderungen
☐ lebendsbedrohlich
☐ stellt eine angeborene Missbildung oder Geburtsfehler dar
☐ stationäre Behandlung oder eine Verlängerung des stationären Aufenthaltes erforderlich
☐ andere wichtige medizinische Gründe

SERIOUS ADVERSE EVENT (SAE)

SAE: Verdachtsdiagnose und Symptome (wenn möglich)

____

Beginn des SAE (TT/MM/JJJJ) __________

Ende des SAE (TT/MM/JJJJ) __________

Todesdatum (falls zutreffend) (TT/MM/JJJJ) __________

SCHWEREGRAD/INTENSITÄT des SAE

Kausalzusammenhang zur Studie : ☐ sicher ☐ wahrscheinlich ☐ möglich ☐ unwahrscheinlich ☐ nicht sicher ☐ ungeklärt

☐ Zusammenhang mit der kognitiven Verhaltenstherapie
☐ Zusammenhang mit der Studienmedikation (Sertraline)
☐ Andere Zusammenhänge zu studienrelevanten Prozeduren

STUDIENMEDIKATION

Sertralin® / Sertralinhydrochlorid Chargennumer.	Datum der ersten Einnahme	Zeit in Stunden zwischen der letzten Einnahme und Beginn des Ereignisses	Tägliche Dosis in mg Art der Einnahme

Zusammenhang mit der Studienmedikation: ☐ sicher ☐ wahrscheinlich ☐ möglich ☐ unwahrscheinlich ☐ nicht sicher ☐ ungeklärt

EUDRACT Nr.: 2005-004525-26 SAE-BERICHT Seite 2 von 2

Bitte alle Informationen vollständig und wahrheitsgemäß mit schwarzem Kugelschreiber eintragen

PATIENTEN INFORMATION

Patient- Nr. (Random Nr.)	Alter (Jahre)	SAE Nr.	☐ INITIALER BERICHT Datum:<br>☐ FOLLOW-UP Datum:

RELEVANTE MEDIZINISCHE ANAMNESE	Start Datum	End Datum
1.		
2.		
3.		

RELEVANTE BEGLEITMEDIKATION

	Indikation	Tägliche Dosierung, Einheit, Art der Einnahme	Datum der ersten Einnahme	Datum der letzten Einnahme
1.				
2.				
3.				

RELEVANTE LABOR- ODER ANDERE UNTERSUCHUNGSERGEBNISSE

	Normalwert	Datum	Ergebnis
1.			
2.			
3.			
4.			

BEHANDLUNG DES SAE	ÄNDERUNG DER STUDIENMEDIKATION	AUSGANG DES SAE
☐ keine<br>☐ Medikamentengabe<br>☐ andere<br>spezifizieren:<br>__________<br>__________<br>__________<br>__________	☐ Dosis nicht geändert<br>☐ Dosis wurde reduziert<br>☐ Dosis wurde erhöht<br>☐ Medikation wurde abgesetzt:<br>Datum: ________<br>Ist eine Reexposition erfogt?<br>☐ Ja ☐ Nein ☐ Unbekannt<br>Kam es zu einer erneuten Reaktion?<br>☐ Ja ☐ Nein ☐ Unbekannt<br>☐ unbekannt<br>☐ nicht zutreffend	☐ wiederhergestellt<br>☐ Besserung<br>☐ nicht wiederhergestellt<br>☐ wiederhergestellt mit Folge-erscheinungen<br>☐ tödlich<br>Im Fall des Todes:<br>Autopsie? ☐ Ja ☐ Nein<br>☐ unbekannt

KOMMENTAR:

UNTERSCHRIFT DES PRÜFARZTES

Name	Unterschrift	Datum (TT/MM/JJJJ)

FAX INNERHALB 24 STUNDEN AN: **FAX No.**

ADVERSE EVENTS

1 Definitions

1.1 Adverse Event

According to GCP, an adverse event (AE) is defined as follows: Any untoward medical occurrence in a subject administered a pharmaceutical product and which does not necessarily have a causal relationship with this treatment. An AE can therefore be any unfavourable and unintended sign (including an abnormal laboratory finding), symptom, or disease temporally associated with the use of a medicinal investigational product, whether or not related to the medicinal investigational product.

An AE may be:

- New symptoms/medical conditions
- New diagnosis
- Changes of laboratory parameters
- Intercurrent diseases and accidents
- Worsening of medical conditions/diseases existing before clinical trial start
- Recurrence of disease
- Increase of frequency or intensity of episodically diseases.

Surgical procedures themselves are not AEs; they are therapeutic measures for conditions that require surgery. The condition for which the surgery is required may be an AE. Planned surgical measures permitted by the clinical trial protocol and the condition(s) leading to these measures are not AEs, if the condition leading to the measure was present prior to inclusion into the trial.

AEs fall into the categories "non-serious" and "serious".

1.2 Serious Adverse Event

A serious adverse event (SAE) is one that at any dose:

- Results in death
- Is life-threatening
- Requires subject hospitalization or prolongation of existing hospitalization
- Results in persistent or significant disability/incapacity or
- Is a congenital anomaly/birth defect.

1.2.1 Suspected unexpected serious adverse reactions (SUSARs)

All adverse drug reactions that are both, serious and unexpected.

2 Period of Observation and Documentation

All AEs reported by the subject or detected by the investigator, will be collected during the trial and must be documented on the appropriate pages of the CRF. AEs must also be documented in the subject's medical records.

In this trial, all AEs that occur after the subject has signed the informed consent document will be documented on the pages provided in the CRF (see Flowchart and chapters 6.1.5, 6.2.17 and 6.4). All subjects who have AEs, whether considered associated with the use of the trial medication or not, must be monitored to determine the outcome. The clinical course of the AE will be followed up by the time of resolve or normalization of changed laboratory parameters or until it has changed to a stable condition.

SAE will be followed up even after trial closure until the AE is resolved or normalization of changed laboratory parameters or until it has changed to a stable condition.

2.1 Assessment of AE intensity

The intensity of an AE should be assessed by the investigator as follows:

mild:	temporary event which is tolerated well by the subject and does not interfere with normal daily activities
moderate:	event which results in discomfort for the subject and impairs his/her normal daily activity.
severe:	event which results in substantial impairment of normal activities of subject.

2.2 Relation to trial participation

The investigator will evaluate each AE occurred after administration of investigational medicinal product regarding the coherency with the administration of the investigational medicinal product possibly exists:

certain:	if there is a reasonable possibility that the event may have been caused by trial participation. A certain event has a **strong temporal relationship** and an alternative cause is unlikely. E.g. the AE abates upon discontinuation of the trial participation and reappears when the trial participation is continued.
probable:	An AE that has a reasonable possibility that the event is likely to have been caused by trial participation. The AE has a **timely relationship** to the trial procedure(s) and **follows a known pattern of response**, but a potential alternative cause may be present.
possible:	An AE that has a reasonable possibility that the event may have been caused by trial participation. The AE has a **timely relationship** to the trial procedure(s); **however, follows no known pattern of response**, and an alternative cause seems more likely, or there is significant uncertainty about the cause of the event.

improbable:	An AE that does not follow a reasonable temporal sequence from trial participation and that is **likely to have been produced** by the subject's clinical state, other modes of therapy or other known etiology.
unrelated:	An AE that does not follow a reasonable temporal sequence from trial participation and that is **definitely caused by** the subject's clinical state, other modes of therapy or other known etiology.
Unknown	**inadequate data** for assessment, **other data** may be expected
not assessable:	**inadequate data** for assessment, **no other data** may be expected

2.3 Action taken

The investigator will record the action taken for each event according to the following:

- None
- Treatment required
- Hospitalization
- Patient withdrawn
- Other (specify)

2.4 Outcome of AE

The investigator will record the outcome for each event according to the following:

Recovered:	The patient has completely recovered without any ongoing disadvantage.
Improved:	The patient recovers from the AE, but it is still present.
Ongoing:	The patient has an ongoing disadvantage which is unlikely to disappear in the future.
Death	
lost to follow up	

3 Reporting of Serious Adverse Events by Investigator

SAEs must be reported within 24 hours after the SAE becomes known using the "Serious Adverse Event" form.

SAE from the diabetological trial sites will be reported to:

KKS Mainz

Langenbeckstr. 2

55131 Mainz

FAX 06131/39-34634

SAE from the psychologists in the coordinating institutions will be reported to:

Westfälische Klinik Dortmund/

Ruhr-Universität Bochum

FAX

(number is requested and will be notified
before start of patient enrolment)

The initial report must be as complete as possible including details of the current illness and (serious) adverse event and an assessment of the causal relationship between the event and the trial medication. The investigator must also inform the site monitor in all cases.

4 Safety evaluation by the sponsor

The investigator will give all informations to the sponsor that he needs for keeping the requirements without delay.

The trial sponsor is responsible for SAE management and reporting as required by German Drug Law (AMG) and GCP regulation (GCP-V).

SUSARs and safety issues as defined by GCP-V are subject of expedited reporting:

The competent authorities and the Ethics Committee should be notified as soon as possible but not later than 15 calendar days (7 days if fatal or life-threatening). All investigators should be informed too.

Work flow and procedures concerning pharmacovigilance are described in SOPs/Manuals.

5 Emergency Unblinding

The trial is unblinded concerning the study treatment.

6 Emergency Treatment

During and following a subject’s participation in the trial, the investigator should ensure that adequate medical care is provided to a subject for any AEs including clinically significant laboratory values. The investigator should inform a subject when medical care is needed for intercurrent illness(es) of which the investigator becomes aware.
