# Supplementary material for: Study protocol of the Diabetes and Depression Study (DAD): a multi-center randomized controlled trial to compare the efficacy of a diabetes-specific cognitive behavioral group therapy versus sertraline in patients with major depression and poorly controlled diabetes mellitus
Source: BMC Psychiatry. 2013 Aug 6;13:206. doi: 10.1186/1471-244X-13-206 (PMC3750698; doi:10.1186/1471-244X-13-206)
Supplement: Additional file 6 — Detailed statistical analysis plan. [file 1471-244X-13-206-S6.pdf]

Statistical Analysis Plan

of the study

Cognitive behavioural therapy vs. sertraline in patients with depression and poorly controlled diabetes mellitus: A randomized controlled trial

Date: 13SEP2011

Status: Final

Author: Christian Ruckes
Interdisciplinary Center for Clinical Trials
University Medical Center Mainz
(Interdisziplinäres Zentrum Klinische Studien (IZKS)
Universitätsmedizin Mainz)

Table of contents

1	Background	4
1.1	Study Objectives	4
1.2	Study Design	4
**FLOW CHART**		5
2	Analysis Populations	6
2.1	Definitions	6
2.2	Scope	7
2.3	Major Protocol Violations	7
3	Study Centres	8
4	Analysis Variables	9
5	Treatment of Missing Values and Outliers	10
6	Statistical Analyses	11
6.1	Disposition of Patients	11
6.2	Demographics	11
6.3	Baseline Characteristics	12
6.3.1	Psychosocial scores	12
6.4	Concomitant diseases	16
6.5	Concomitant medication	16
6.6	Extent of Exposure to Study Treatment	16
6.7	Adherence to Therapy	18
6.8	Primary Analysis	19
6.9	Secondary analyses	20
6.9.1	Efficacy	20
6.9.2	Safety	34
6.10	Subgroup Analyses	35
6.11	Interim Analysis	38
7	Software	39
8	Appendix 1 – List of Variables	40
8.1	Disposition of Patients	40
8.2	Demographics	41
8.3	Baseline Characteristics	42
8.4	Primary Variable	48
8.5	Secondary Variables	48
8.5.1	Efficacy	48
8.5.2	Safety	52
9	Appendix 2 – Recoded Values of the SF-36	53
10	Appendix 3 – Population means and Standard deviations for calculation of SF-36 z-scores	55
11	Appendix 4 – Calculation of SER-Adherence	60

Signatures

PD Dr. Matthias J. Müller<br>Coordinating Investigator	25.1.2012 [signature]<br>Date, signature
Prof. Dr. F. Petrak,<br>Sponsor Representative	30.1.2012 Petrak<br>Date, signature
C. Ruckes<br>Trial Statistician	17.1.2012 Christian Ruckes<br>Date, signature

1 BACKGROUND

1.1 Study Objectives

To compare the efficacy of diabetes-specific cognitive behavioural therapy vs. antidepressive medication, specifically the selective serotonin reuptake inhibitor (SSRI) sertraline, regarding improvement of glycaemic control in patients with poorly controlled diabetes and depression.

The primary outcome variable is improvement of the $HbA1_c$ at the one-year follow-up; the most important secondary outcome variable is remission of depression.

1.2 Study Design

Multicentre randomized controlled trial (RCT) comparing sertraline vs. CBT. After 12 weeks of open-label therapy, only the treatment-responders (50% improvement of depression) of both groups will be included in the one-year long-term phase of the study. In the long-term phase, diabetological treatment as usual will be given to both groups. CBT-responders will receive no further treatment, while SSRI-responders will be given a sustained sertraline regimen as relapse prevention.

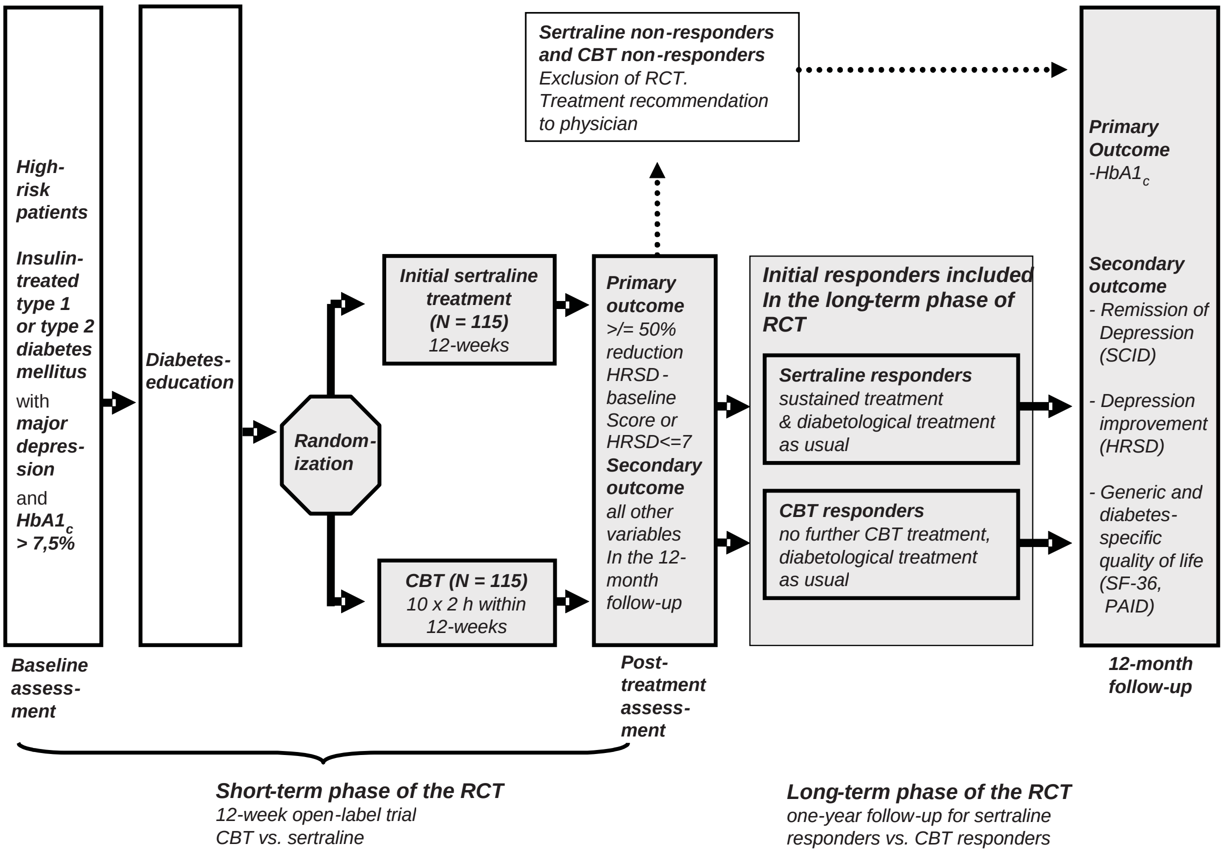

Figure 1: Trial schedule with planned/expected patient numbers

All analyses described contribute to the final study report of the study.

FLOW CHART

	baseline phase			***12 week short-term phase (S)***					***12 months long-term phase (L)***				***End of Study Visit***
Planned Study Day	*-42 to -16*	*-15*	*- 14 to -1*	*1*	*15*	*56*	*83*	*84*	*175*	*267*	*358*	*449*	*450*
Visit	*Screening*	*P0*	***2 sessions of diabetes education***	*S1*	*S2**	*S3*	*S4*	*P1*	*L1*	*L2*	*L3*	*L4*	*P2*
Screening information, informed consent	*D*												
Screening, inclusion/exclusion criteria	*D*												
Blood-sample (HbA1c)	*D*			*D*			*D*		*D*	*D*	*D*	*D*	
Blood-sample (liver enzymes, other endocr. parameters)	*D*			*D*					*D*			*D*	
Treatment preference		*P*											
CES-D questionnaire	*D*			*D*			*D*		*D*	*D*	*D*	*D*	
Diagnosis information, informed consent	*D*												
Other questionnaires		*P*						*P*					*P*
Hamilton Rating Scale (HRSD-17)		*P*						*P*					*P*
SCID-Interview		*P*											*P*
Review mental disorder related inclusion/exclusion criteria		*P*											
Treatment information, informed consent		*P*		*D*									
Randomisation				*D*									
CBT group: Cognitive behavioural therapy				***10 sessions P***									
Sertraline group: Delivery of medication				*D*	*D*	*D*	*D*		*D*	*D*	*D*	*D*	
Sertraline group: Blood-sample (plasma concentrations of sertraline)						*D*	*D*		*D*	*D*	*D*	*D*	
Diabetological treatment as usual	*D*			*D*			*D*		*D*	*D*	*D*	*D*	
Dose adjustment insulin/sertraline				*optional supplementary visits in case of problems with diabetological treatment or side-effects of sertraline D*									
Recording and monitoring of adverse events				*continuously D, P*									
Recording of concomitant medication				*continuously D*									
Review inclusion/exclusion criteria				*continuously D, P*									

P = visit to the coordinating institutions were questionnaire and interviews will be administered by members of the research team (research assistants, psychologists)
*D = visit to the treating physician or research assistants in the diabetologic trial centres. *Visit S2 is optional by telephone or personally*

2 ANALYSIS POPULATIONS

The study population is defined by the following key inclusion and exclusion criteria:

Key inclusion criteria

- Type 1 or type 2 diabetes mellitus diagnosed at least 12 months beforehand
- Insulin treatment for at least the past 6 months
- 21 to 69 years of age
- Poor glycaemic control: $HbA1_c$ level > 7,5 % within the nine preceding months
- Current major depression (DSM-IV-TR criteria)
- Living near the coordination institution where CBT treatment will take place (<1 hour access)
- Able to understand study and individual consequences

Key exclusion criteria

- Clinically significant suicide risk or history of attempted suicide in the past 12 months
- History of schizophrenia or psychotic symptoms
- Bipolar disorder
- Organic brain syndrome or dementia
- Alcohol or substance abuse or dependence in the past 6 months

2.1 Definitions

Randomized Population [Randomized]: All randomized subjects.

Safety Population [Safety]: All randomized subjects with at least one therapy session (CBT) or at least one sertraline (SER) intake and at least one safety assessment after therapy.

Intent-to-Treat Population (ITT Population) [ITT]: The subset of the randomized population of the therapy responders after the short-term phase (HRSD-17 reduction of at least 50% or HRSD-17 less than or equal to 7). The analyses for this study might be omitted, if too few responders will be observed.

Per Protocol Population (PP Population) [PP]: The subset of the ITT Population and the Safety Population without any major protocol violation as defined in 2.3.

For analyses of the Randomized or ITT Population the subjects will be assigned to the treatment allocated at randomization. For analyses employing the Safety or PP Population the subjects will be assigned to the treatment they actually received.

2.2 Scope

Demographic analyses will be performed for all populations mentioned above. The primary population for efficacy will be the ITT Population [ITT]. Randomized Population [Randomized] and PP Population [PP] analyses will be employed as sensitivity analyses of the primary outcomes of interest to check the primary analysis results for robustness. The primary population for analysis might be switched from the ITT Population to the Randomized Population, when the absolute difference of responders between the treatment groups deviates by more than 10%.

2.3 Major Protocol Violations

The following criteria will be regarded as major protocol violations and the subjects will be excluded from the PP Population:

- Not fulfilling all inclusion criteria
- Fulfilling at least one exclusion criterion
- No documented sertraline intake at all or never participate in a CBT session
- Missing values of the HbA1c at baseline or at study day 445 (Visit L4)
- Deviation of the visit schedule of more than 90 days at Visit L4
- Unallowed concomitant medications will be discussed and decided by the coordinating investigator and the sponsor representative on an individual basis

3 STUDY CENTRES

Four trial coordinating psychological institutions (Mainz, Bochum/Dortmund, Düsseldorf/Köln and Bad Mergentheim) coordinated the recruitment in 70 diabetologic trial centres located in the vicinity of the four coordinating institutions.

4 ANALYSIS VARIABLES

All variables (including all derived variables) are listed in Appendix 1

5 TREATMENT OF MISSING VALUES AND OUTLIERS

The primary analyses will be focussed on observed cases. For HbA1c and the HRSD-17 score the analysis will be repeated after employing the last observation carried forward method. If more than 10% but less than 40% of the values are missing, multiple imputation methods could be considered. An appropriate method has to be chosen on an individual basis when the missing value pattern becomes known.

Outliers will not be investigated. If outliers become apparent, they will be set to missing, if they bias the analysis.

6 STATISTICAL ANALYSES

The default summary statistics for quantitative variables will be the number of observations (n), mean, standard deviation (SD), median, interquartile-range (IQA), minimum and maximum for those patients with data available.

For qualitative variables, the number (n) and percentage (%) of patients with non-missing data per category will be the default summary presentation. If appropriate, the number of missing values will be displayed as a “Missing” category. Unless otherwise stated percentages will be calculated using a denominator of all patients in the specified population or treatment group.

Exploratory p-values of the t-test and Chi-Square test between treatment groups will be shown for quantitative and qualitative variables respectively. For quantitative variables the standardized effect size (ES) will be presented for absolute values and changes to baseline. It will be defined as ES = (mean visit y CBT – mean visit y SER) / (pooled SD at baseline) for the quantitative values itself or ES = (mean change visit y and baseline CBT – mean change visit y and baseline SER) / (pooled SD for the quantitative variable at baseline) for the changes to baseline. For effect sizes derived from analysis models (ANCOVA or Mixed models the least square mean instead of the mean will be taken). For dichotomous variables the Odds Ratio (OR) from the analysis models will be taken as a measure of effect size. For other categorical variables no reliable effect size measures are available.

All variables will be displayed by treatment group and with a total column.

6.1 Disposition of Patients

The following patient disposition variables will be presented by numbers and percentages: Informed consent given to questionnaires [SC_2] and diagnostics [SC_46, P0_2], inclusion [SC_4 – SC_9] and exclusion criteria [SC_10 – SC_13] (questionnaires, psychologist, diagnostics), size of analysis populations (Randomized, Safety, ITT, PP), completion of diagnostics part [ED_2] and psychological part [EP_2], reasons for discontinuation of diagnostics part [ED_3 – ED_19] and psychological part [EP_3 – EP_19], study completion according to protocol [ED_2,EP_2], reasons for discontinuation [ED_3 – ED_19, EP_3 – EP_19], participation on every study visit (if the visit date [S1_1, S2_1, S3_5, S4_1, L1_31, L2_1, L3_1, L4_1, P0_1, P1_1, P2_11] is available, participation at the visit is assumed), on every short-term visit [S1_1, S2_1, S3_5, S4_1, P0_1, P1_1] and on every long-term visit [L1_31, L2_1, L3_1, L4_1, P2_11] and number and type of protocol violations ([ProtViolTyp] for definition see section 2.3).

A logistic regression for the Randomized population will be done with responder after the short term phase as dependent variable [ITT] and will be explored for treatment [S1_12] differences. The analysis will be controlled for baseline HbA1c [s1_77] and HRSD-17 [p0_hrsdsc]. If the HbA1c value at baseline is missing, the HbA1c at visit SC [SC_118_1/2] will be used. HRSD-17 Baseline is the latest value before treatment start at visit P0 [p0_hrsdsc].

Tables: 6.1.1, 6.1.2, 6.1.3.1/2, 6.1.4.1.1

6.2 Demographics

The following variables will be derived:

Age [AGE] [years] = year of randomisation minus year of birth

Education years [Education]:

<10: "Hauptschulabschluss" from the school education variable
10-14: "Mittlere Reife", "Abitur or Fachhochschulreife" from the school education variable
>14: "Hochschulabschluss" from the school education variable

Continuous income [€] [Income]:

<750:	0.4
750-1500:	1.125
1500-2000:	1.75
2000-2500:	2.25
2500-3000:	2.75
3000-4000:	3.5
4000-5000:	4.5
>5000:	5.5

Age [AGE], number of children [P0_5] and continuous income [Income] will be summarised by the descriptive statistics n, mean, standard deviation, median, interquartile-range (IQA), minimum and maximum. Exploratory p-values of the t-test between treatment groups [S1_12] will be shown.

The demographics gender [Sex], marital status [SC_16], school education [SC_17], education years [Education], employment status [P0_6], employment group [P0_7], income categories [P0_8] and nationality [P0_9] will be presented by number and percentage. Exploratory p-values of the Chi-Square test between treatment groups [S1_12] will be shown.

The results will be displayed for the Randomized Population [Randomized], Safety Population [Safety], ITT Population [ITT] and PP Population [PP].

Tables: 6.2.1.1/2/3/4, 6.2.2.1/2/3/4, 6.2.3.1/2/3/4

6.3 Baseline Characteristics

6.3.1 Psychosocial scores

Scores of HRSD-17 [P0_hrsdsc], SF-36 [P0_1_SF_PF, P0_1_SF_RP, P0_1_SF_BP, P0_1_SF_GH, P0_1_SF_V, P0_1_SF_SF, P0_1_SF_RE, P0_1_SF_MH, P0_SF_sMH, P0_SF_sPH], PAID [P0_1_x_paisc], ADS [S1_1_x_adssc_kategorial, S1_1_x_adssc_final], SCL-K-9 [P0_1_x_sclk9sc], CTQ [P0_1_x_easc, P0_1_x_pansc, P0_1_x_sasc, P0_1_x_ea2sc, P0_1_x_phnsc, P0_1_x_edsc], DWT type 1 [P0_1_x_dt1sc], DWT type 2 [P0_1_x_dt2sc, P0_356, P0_357, P0_358, P0_359, P0_360], IPC-D1 [P0_1_x_intSC, P0_1_x_PhySC, P0_1_x_UnpSC, P0_1_x_FoChSC], FKV-15 [P0_1_x_acsc, P0_1_x_dhsc, P0_1_x_disc, P0_1_x_exsc, P0_1_x_cosc], SE [P0_1_x_sesc], BIT [P0_1_x_fitsc, P0_1_x_rosc, P0_1_x_ehsc, P0_1_x_stsc, P0_1_x_hysc, P0_1_x_bitsc], SDSCA [P0_1_x_gdsc, P0_1_x_sdsc, P0_1_x_bgsc, P0_1_x_fcsc], PFUK-R [P0_1_emos_v, P0_1_emos_a, P0_1_entl_v, P0_1_entl_a, P0_1_glob_v, P0_1_glob_a], H-Skala [P0_1_x_hrasc] and K-INK [P0_1_x_apasc, P0_1_x_avasc, P0_x_tssc] will be presented by appropriate descriptive statistics (mean, standard deviation, median, interquartile-range (IQA), minimum and maximum for quantitative variables and absolute and relative frequencies for qualitative statistics).

Exploratory p-values of the t-test and Chi-Square test between treatment groups [S1_12] will be shown.

The results will be displayed for the Randomized Population [Randomized], Safety Population [Safety], ITT Population [ITT] and PP Population [PP].

Tables:

HRSD 6.3.1.1.1/2/3/4,

SF-36 6.3.1.2.1/2/3/4,

PAID 6.3.1.3.1/2/3/4,

ADS 6.3.1.4.1/2/3/4,

SCLK-9 6.3.1.5.1/2/3/4,

CTQ 6.3.1.6.1.1/2/3/4,

DWT1 6.3.1.7.1/2/3/4,

DWT2 6.3.1.8.1/2/3/4,

IPC-D1 6.3.1.9.1/2/3/4,

FKV-15 6.3.1.10.1/2/3/4,

SE 6.3.1.11.1/2/3/4,

BIT 6.3.1.12.1/2/3/4,

SDSCA 6.3.1.13.1/2/3/4,

PFUK-R 6.3.1.14.1/2/3/4,

H-Skala 6.3.1.15.1/2/3/4,

K-INK 6.3.1.16.1/2/3/4

Childhood Trauma Questionnaire (CTQ)

The CTQ is a 28-item questionnaire (1=”not at all” to 5=”very often”) assessing the adverse childhood experiences.

There are 6 subscales: Emotional Neglect (items 5, 7, 13, 19, and 28 (all items are reversed and summed), range 5 to 25) [P0_1_x_easc], Physical Abuse (items 9, 11, 12, 15, and 17 (sumscore), range 5 to 25) [P0_1_x_pansc], Physical Neglect (items 1, 2, 4, 6, and 26 (items 2 and 26 reversed, sumscore), range 5 to 25) [P0_1_x_phnsc], Sexual Abuse (items 20, 21, 23, 24, and 27 (sumscore), range 5 to 25) [P0_1_x_sasc], Emotional Abuse (items 3, 8, 14, 18, and 25 (sumscore), range 5 to 25) [P0_1_x_ea2sc], and Extenuation/Denial (items 10, 16, and 22 (1, if any of the items is 5, else 0), range 0 to 3) [P0_1_x_edsc]. All subscores are computed using the mean of all non-missing single items for the subscore multiplied by 5. A subscore will be set to missing, if more than 1 item is missing. For the Denial score [P0_1_x_edsc] all three items (10, 16, 22) are summed up. No missing items are allowed.

For calculation of HRSD-17, SF-36, PAID, ADS, SCL-K-9, DWT type 1, DWT type 2, IPC-D1, FKV-15, SE, BIT, SDSCA, PFUK-R, H-Skala and K-INK see section 6.9.1.

6.3.2 Medical Data

The following variables will be derived:

Any late complications [DiabComp]: At least one of the following categories for diabetes related concomitant diseases (neuropathy [SC_63], nephropathy [SC_64], retinopathy [SC_65]) is ticked “Yes”.

Number of late complications [NumberDiabComp]: The number of ticked ”Yes” categories for diabetes related concomitant diseases (neuropathy [SC_63], nephropathy [SC_64], retinopathy [SC_65]) from the CRF.

KHK [KHK]: At least one of the following categories for diabetes related concomitant diseases (coronary heart disease [SC_69], myocardial infarction [SC_70], Bypass-OP [SC_71]) is ticked “Yes”.

Any macrovascular complications [Comorb]: At least one of the following categories for diabetes related concomitant diseases (PAVK [SC_72], Apoplex [SC_76], KHK [KHK]) is ticked “Yes”.

Number of macrovascular complications [NumComorb]: The number of ”Yes” categories for diabetes related concomitant diseases (PAVK [SC_72], Apoplex [SC_76], KHK [KHK]) .

Systolic blood pressure [SC_60], diastolic blood pressure [SC_59], pulse [SC_61], height [SC_55], abdominal girth [SC_56], weight [SC_57] and hip size [SC_58] will be summarised by the descriptive statistics n, mean, standard deviation, median, interquartile-range (IQA), minimum and maximum.

The parameters type of diabetes mellitus [SC_54], treatment with hypertensive medication [SC_62], existence of diabetes related diseases (at least one of [SC_63 – SC_77] ticked “yes”), diabetes related diseases by type of disease [SC_63 – SC_77], other relevant diseases [SC_79] and current concomitant medication [SC_80] will be presented by number and percentages.

Exploratory p-values of the t-test and Chi-Square test between treatment groups [S1_12] will be shown.

The results will be displayed for the Randomized Population [Randomized], Safety Population [Safety], ITT Population [ITT] and PP Population [PP].

Tables: 6.3.2.1.1/2/3/4, 6.3.2.2.1/2/3/4, 6.3.2.3.1/2/3/4, 6.3.2.4.1/2/3/4, 6.3.2.5.1/2/3/4, 6.3.2.6.1/2/3/4

6.3.3 Current diabetes treatment

The following variables will be derived:

Diabetes duration [years] [DUR_1] = year of randomisation [S1_9] minus year of first manifestation of diabetes [year of SC_81]

Duration of oral antidiabetic medication [years] [DUR_3] = year of randomisation [S1_9] minus year of intake of oral antidiabetic medication [year of SC_100]

Total daily dose of insulin [units] = sum of the daily basal and daily prandial insulin doses [SC_91, SC_93, SC_95, SC_97]

Diabetes duration [DUR_1], duration of oral antidiabetic medication [DUR_3], duration of insulin treatment [DUR_2], daily basal dose [SC_93, SC_97], daily prandial dose [SC_91, SC_95] and total daily dose of insulin [SC_91, SC_93, SC_95, SC_97] will be summarised by the descriptive statistics n, mean, standard deviation, median, interquartile-range (IQA), minimum and maximum. (Comment: Doses will be displayed for each medication type separately).

Current diabetes treatment_[SC_83, SC_84, SC_85], application form of insulin [SC_87, SC_88, SC_89], type of insulin [SC_90, SC_92, SC_94, SC_96] and type of oral anti-diabetics [SC_101, SC_103, SC_105, SC_107] will be presented by number and percentages.

Exploratory p-values of the t-test and Chi-Square test between treatment groups [S1_12] will be shown.

The results will be displayed for the Randomized Population [Randomized], Safety Population [Safety], ITT Population [ITT] and PP Population [PP].

Tables: 6.3.3.1.1/2/3/4, 6.3.3.2.1/2/3/4

6.3.4 Laboratory values

The parameters HbA1c [SC_118_1,SC_118_2, S1_77], ASAT/SGOT[SC_119_1, SC_119_2] and ALAT/SGPT [SC_120_1, SC_120_2] will be summarised by the descriptive statistics n, mean, standard deviation, median, interquartile-range (IQA), minimum and maximum. Exploratory p-values of the t-test between treatment groups [S1_12] will be shown.

The results will be displayed for the Randomized Population [Randomized], Safety Population [Safety], ITT Population [ITT] and PP Population [PP].

Tables 6.3.4.1/2/3/4

6.3.5 Concomitant psychological disorders according to SKID

The SKID is an instrument for the diagnosis of mental disorders. It assesses the more commonly occurring psychiatric disorders described in the DSM-IV.

The number of previous episodes [P0_37] and age at onset of first depressive episode [P0_36] will be summarised by the descriptive statistics n, mean, standard deviation, median, interquartile-range (IQA), minimum and maximum.

The parameters current bipolar I (yes/no) [P0_14], current bipolar II (yes/no) [P0_16], current other bipolar disorder (yes/no) [P0_18], current major depression (yes/no) [P0_19], course of the major depression (single episode, recurrent episode, with seasonal pattern) [P0_20, P0_21, P0_22], dysthymia (yes/no) [P0_44], current depression not otherwise specified (yes/no) [P0_50] will be presented by numbers and percentages.

Exploratory p-values of the t-test and Chi-Square test between treatment groups will be shown.

The results will be displayed for the Randomized Population [Randomized], Safety Population [Safety], ITT Population [ITT] and PP Population [PP].

Tables 6.3.5.1.1/2/3/4, 6.3.5.2.1/2/3/4, 6.3.5.3.1/2/3/4, 6.3.5.4.1/2/3/4

6.3.6 Sertraline specific items

Plasma concentrations of sertraline [SER_9_1] and desmethylsertraline [SER_11_1] and daily dose [SER_2_1] will be summarised by the descriptive statistics n, mean, standard deviation, median, interquartile-range (IQA), minimum and maximum.

Intensity of depression according to CGI [S1_44] will be presented by numbers and percentages.

The results will be displayed for the Randomized Population [Randomized], Safety Population [Safety], ITT Population [ITT] and PP Population [PP].

Tables 6.3.6.1.1/2/3/4, 6.3.6.2.1/2/3/4, 6.3.6.3.1/2/3/4

6.4 Concomitant diseases

The following variables will be derived:

Duration of concomitant disease [DUR_5_1 – DUR_5_20] [years] = year of randomisation [S1_9] minus year of start of concomitant disease [MH_3_1 – MH_3_20]

The average duration of concomitant disease [DUR_5_1 – DUR_5_20] per patient [ConDisDur] will be summarised by the descriptive statistics n, mean, standard deviation, median, interquartile-range (IQA), minimum and maximum.

Concomitant diseases [MH_1_1 – MH_1_20] will be coded by MedDRA (Medical Dictionary for Regulatory Activities) terminology and presented by number and percentages within preferred term and system organ class.

The results will be displayed for the Randomized Population [Randomized], Safety Population [Safety], ITT Population [ITT] and PP Population [PP].

Tables 6.4.1.1/2/3/4, 6.4.2.1/2/3/4

6.5 Concomitant medication

The following variable will be derived:

Duration of concomitant medications (for ongoing medications) [DUR_6_1 – DUR_6_14] [years] = year of start of study minus year of start of concomitant medications [CMS4_1-CMS_4_15]

The average duration of concomitant medications [DUR_6_1 – DUR_6_14] per patient [ConMedDur] will be summarised by the descriptive statistics n, mean, standard deviation, median, interquartile-range (IQA), minimum and maximum.

Current concomitant medication [SC_80] and treatment with antihypertensive medication [SC_62] will be presented by numbers and percentages. Medication name [SC_80 and SC_62] will be coded according to Deutsche Rote Liste. CRF entries with a stop date before study start will be removed from the table. Concomitant medication [SC_80 and SC_62] will be provided by “Hauptgruppe” and “Standardisierter Medikationsname” within “Hauptgruppe”. Patients with at least one concomitant medication will be displayed by number and percentages.

The results will be displayed for the Randomized Population [Randomized], Safety Population [Safety], ITT Population [ITT] and PP Population [PP].

Tables 6.5.1.1/2/3/4, 6.5.2.1/2/3/4

6.6 Extent of Exposure to Study Treatment

6.6.1 **Study completion**

Duration of study participation [days] [DUR_4] will be summarised by the descriptive statistics n, mean, standard deviation, median, interquartile-range (IQA), minimum and

maximum. Subjects participating in each study visit (if the visit date [S1_1, S2_1, S3_5, S4_1, L1_31, L2_1, L3_1, L4_1, P0_1, P1_1, P2_11] is available, participation at the visit is assumed) will be presented by numbers and percentages.

The results will be displayed for the Randomized Population [Randomized], Safety Population [Safety], Per Protocol Population [PP] and ITT Population [ITT].

There will be an overall summary for the short-term and long-term phase. It will be presented by numbers and percentages together with the patient disposition for the Randomized Population [Randomized].

The following variable will be derived:

Duration of study participation [days] [DUR_4] = date of end of study (latest of the dates [ep_1, ed_1]) minus date of randomisation [S1_9]

Tables 6.6.1.1/2/3/4

6.6.2 Drop-Outs

Drop-outs are defined as patients with at least one reason for study termination (from either diagnostician or psychologist i. e. at least one of the following variables is ticked [ED_3-ED_8, ED_10, ED_12-ED_18, EP_3- EP _8, EP _10, EP_12- EP _18).

For analysis the following more specific criteria for comparing drop-outs were defined:

Starter vs. Non-Starter [Starter]:
Starter: at least one documented treatment (one session CBT/ 1 tablet sertraline)
Non-Starter: no documented treatment (no session CBT/ no tablet sertraline)
Patients who did not receive treatment and discontinued due to medical reasons were excluded from the comparison.

Completer Short-term phase vs. patients discontinued therapy [KZG]:
Completer: Patients with at least one documented treatment (one session CBT/ 1 tablet sertraline) completing the short-term phase (appearance at Visit S4/P1)
Discontinued: Patients with at least one documented treatment (one session CBT/ 1 tablet sertraline) discontinuing the short-term-phase sertraline
Patients discontinued due to medical reasons and therefore did not complete the short-term phase are excluded from the comparison.

Completer Long-term phase vs. patients discontinued therapy [LZG]:
Completer Long-term phase: *All patients with at least one documented treatment (CBT/sertraline) completing the study according to protocol* (i. e. non-responder according to HRSD-17 score completed the study, if they completed the study until visit S4/P1; responders completed the study, if they completed the study until visit L4/P2)
Discontinued: *All patients with at least one documented treatment (CBT/sertraline)* discontinuing the study in short-term phase or long-term phase of the study..

Patients discontinued due to medical reasons and therefore did not complete the study are excluded from the comparison.

Exploratory p-values of the Chi-Square test between completer classification [Starter, KZG, LZG] and treatment groups [S1_12] within completer classification [Starter, KZG, LZG] will be shown. For drop-outs exploratory p-values of the t-test and Chi-Square test for the

following baseline characteristics between completer classification [Starter, KZG, LZG] and treatment groups [S1_12] within completer classification [Starter, KZG, LZG] will be shown: gender [SC_14], age [AGE], marital status [SC_16], school education [SC_17], education years [Education], employment status [P0_6], employment group [P0_7], continuous income [Income], nationality [P0_9], number of children [P0_5], type of diabetes [SC_54], medical data (see section 6.3.2), current diabetes treatment (see section 6.3.3), baseline (HbA1c) [S1_77 or SC_118_1/2 respectively], SKID (course of major depression [P0_20, P0_21, P0_22], dysthymia [P0_44], number of depressive episodes [P0_37], age at onset of first depression [P0_36] and baseline HRSD-17 score [P0_hrsdsc, P0_hrsdsc_kategorial_final].

Tables:

Demographic: 6.6.2.1.1.1/2/3, 6.6.2.1.2.1/2/3, 6.6.2.1.3.1/2/3, 6.6.2.2.1.1/2/3, 6.6.2.2.2.1/2/3, 6.6.2.2.3.1/2/3, 6.6.2.3.1.1/2/3, 6.6.2.3.2.1/2/3, 6.6.2.3.3.1/2/3

Medical data: 6.6.2.4.1.1/2/3, 6.6.2.4.2.1/2/3, 6.6.2.4.3.1/2/3

Current diabetes treatment: 6.6.2.5.1.1/2/3, 6.6.2.5.2.1/2/3, 6.6.2.5.3.1/2/3, 6.6.2.6.1.1/2/3, 6.6.2.6.2.1/2/3, 6.6.2.6.3.1/2/3

Baseline HbA1c: 6.6.2.7.1.1/2/3, 6.6.2.7.2.1/2/3, 6.6.2.7.3.1/2/3

SKID: 6.6.2.8.1.1/2/3, 6.6.2.8.2.1/2/3, 6.6.2.8.3.1/2/3, 6.6.2.9.1.1/2/3, 6.6.2.9.2.1/2/3, 6.6.2.9.3.1/2/3, 6.6.2.10.1.1/2/3, 6.6.2.10.2.1/2/3, 6.6.2.10.3.1/2/3, 6.6.2.11.1.1/2/3, 6.6.2.11.2.1/2/3, 6.6.2.11.3.1/2/3

Baseline HRSD-17 score: 6.6.2.12.1.1/2/3, 6.6.2.12.2.1/2/3, 6.6.2.12.3.1/2/3

6.7 Adherence to Therapy

The following variables will be derived:

Adherence full ITT sample [Adherence_ITT]: 0= non-adherent/partially non-adherent, 1= adherent.

Patients are categorized as non-adherent/partially non-adherent if

a) Participation in 0-7 CBT sessions [CB_2, CB_5, CB_8, CB_11, CB_14, CB_17, CB_20, CB_23, CB_26, CB_29] **or**
b) Categorized as non-adherent or partially non-adherent with regard to SER-adherence ITT-variable [Adherence_ITT]

Patients are categorized as adherent if

a) Participation in at least 8 session CBT [CB_2, CB_5, CB_8, CB_11, CB_14, CB_17, CB_20, CB_23, CB_26, CB_29] **or**
b) Categorized as adherent with regard to SER-adherence ITT-variable. [Adherence_ITT]

Adherence full Random sample [Adherence_Randomised]: 0= non-adherent/partially non-adherent, 1= adherent.

Patients are categorized as non-adherent/partially non-adherent if

c) Participation in 0-7 CBT sessions [CB_2, CB_5, CB_8, CB_11, CB_14, CB_17, CB_20, CB_23, CB_26, CB_29] **or**
d) Categorized as non-adherent or partially non-adherent with regard to SER-adherence- Random-variable [Adherence_Randomized]

Patients are categorized as adherent if

c) Participation in at least 8 session CBT [CB_2, CB_5, CB_8, CB_11, CB_14, CB_17, CB_20, CB_23, CB_26, CB_29] **or**

d) Categorized as adherent with regard to SER-adherence-Random-variable. [Adherence_Randomized]

CBT-adherence [Adherence_CBT] categories: 1=adherent, patient attended to at least 8 CBT-sessions [CB_2, CB_5, CB_8, CB_11, CB_14, CB_17, CB_20, CB_23, CB_26, CB_29], 2= partially non-adherent, patient attended to 1-7 CBT sessions [CB_2, CB_5, CB_8, CB_11, CB_14, CB_17, CB_20, CB_23, CB_26, CB_29], 3=non-adherent, patients participated in 0 CBT-sessions [CB_2, CB_5, CB_8, CB_11, CB_14, CB_17, CB_20, CB_23, CB_26, CB_29]

SER-adherence1 [Adherence1_SER_ITT] categories: “1_adherent”, “2_partially non-adherent”, “3 non-adherent”

SER-adherence2 [Adherence2_SER_ITT] categories: “1 adherent”, “2 partially non-adherent”, “3 hidden non-adherent”, “4 open non-adherent”.

Adherence1_SER_Ran: 1 = adherent, 2 = partially non-adherent, 3 = non-adherent

Adherence2_SER_Ran: 1 = adherent, 2 = partially non-adherent, 3 = hidden non-adherent, 4 = open Non-adherent.

For calculation of CBT- and SER-adherence see Appendix 4.

CBT-adherence [Adherence_CBT] and SER-adherence [Adherence1_SER_ITT, Adherence2_SER_ITT; Adherence1_SER_RAN, Adherence2_SER_RAN] will be presented by numbers and percentages. Number of CBT sessions [NumberCbtTs] will be summarised by the descriptive statistics n, mean, standard deviation, median, interquartile-range (IQA), minimum and maximum.

The results will be displayed for the Randomized Population [Randomized] and ITT Population [ITT].

Tables 6.7.1.2, 6.7.2.1/2, 6.7.3.1/2, 6.7.4.1/2, 6.7.5.2, 6.7.6.1/2/3/4

6.8 Primary Analysis

The primary variable is the HbA1c plasma level.

The primary endpoint is the difference of the HbA1c value at the end of the long-term phase (visit L4) [L4_69] and the baseline visit ("change of glycaemic control") [S1_77].

Primary outcome: The primary outcome of the study is "change of glycaemic control", compared between the different treatment groups [S1_12] using an Analysis of Covariance (ANCOVA).

The variables HbA1c and HRSD-17 at baseline serve as predefined control variables (motivated by literature). HbA1c baseline is the value at visit S1 [S1_77]. If the HbA1c value at baseline is missing, the HbA1c at visit SC [SC_118_1/2] will be used. HRSD-17 baseline is the latest value before treatment start at visit P0 [p0_hrsdsc].

The following hypothesis will be tested:

H_0: $\mu_{CBT} = \mu_{SER}$

versus

H_1: $\mu_{CBT} \neq \mu_{SER}$

where μ_{CBT} and μ_{SER} are the expected mean values of the differences in HbA1c values belonging to the CBT treatment group [S1_12] and the SER treatment group [S1_12] respectively. The hypothesis will be tested on a two-sided level of significance α=0.05. The results will be displayed by means of p-value, estimate and 95%-confidence interval.

The primary analysis will be conducted for the ITT Population [ITT]. The analyses for the Randomized Population [Randomized], Safety Population [Safety] and PP Population [PP] will be considered as secondary.

The primary analysis will be repeated by employing the last observation carried forward (LOCF) method to missing data in HbA1c [HbA1c_S1_L, HbA1c_S4_L, HbA1c_L1_L, HbA1c_L2_L, HbA1c_L3_L, HbA1c_L4_L.

The HbA1c values over time [SC_118_1/2, S1_77, S4_67, I1_69, I2_67, I3_67, I4_69] will be displayed by descriptive statistics (n, mean, standard deviation, median, interquartile-range (IQA), minimum and maximum) and exploratory p-values of the t-test and the effect size between treatment groups [S1_12] will be shown. Descriptive statistics will also be done by coordinating institution [K_Zentrum] to explore centre effect. Moreover, the HbA1c values [SC_118_1/2, S1_77, S4_67, I1_69, I2_67, I3_67, I4_69] over time will be displayed by Box-Whisker-plots.

Tables 6.8.1.1.1/2/3/4, 6.8.1.2.1/2/3/4, 6.8.2.1.1/2/3/4, 6.8.2.2.1/2/3/4

Box-Whisker-Plots: 6.8.3.

6.9 Secondary analyses

6.9.1 Efficacy

6.9.1.1 Change of glycaemic control after adjustment for potential confounders.
The "change of glycaemic control" [L4_69 minus S1_77] will additionally be analysed by means of an ANCOVA with additional control variables. A correlation analysis with following control variables will be performed:

- Age [years] [Age]
- Sex (male/female) [SC_14]
- Coordinating institution (Bad Mergentheim, Dortmund/Bochum, Düsseldorf/Köln, Mainz) [K_Zentrum]
- Diabetes type (type 1/type 2) [SC_54]
- Late complications (Retinopathie [SC_65], Nephropathie [SC_64], Neuropathie [SC_63]) [DiabComp]
- Macrovascular complications (KHK [KHK], Apoplex [SC_76], PAVK [SC_72]) [Comorb]
- Education years (<10/10-14/>14) [Education]

- o Continuous income [€] [Income]
- o Single/recurrent episode(s) (Note: recurrent episodes include the category “with seasonal pattern”) [p0_20/(p0_21,p0_22)]
- o Comorbidity of other mental disorders (yes (any concomitant psychological disorders according to SKID)/no) [CoMenDis]

Note: The coordinating institution Bad Mergentheim will be set to missing.

The resulting p-values will be checked. If Pearson’s correlation coefficient has a p-value <.10, the control variable will be used in a subsequent ANCOVA. The subsequent ANCOVA will consist of treatment group [S1_12], HbA1c baseline, HRSD-17 baseline, and the selected control variables. HbA1c baseline is the value at visit S1 [S1_77]. If the HbA1c value at baseline is missing, the HbA1c at screening visit [SC_118_1/2] will be used. HRSD-17 baseline is the latest value before treatment start at visit P0 [p0_hrsdsc].

The results will be displayed by means of p-value, estimate, 95%-confidence interval and effect size.

The results will be displayed for the Randomized Population [Randomized], Safety Population [Safety] and ITT Population [ITT].

Tables 6.9.1.1.1.1/2/3, 6.9.1.1.2.1/2/3

6.9.1.2 Change of glycaemic control with repeated measurements

Another analysis of the outcome change of glycaemic control [L4_69 minus S1_77] will be done by a mixed model with repeated measurements incorporating all HbA1c measurements [S4_67, I1_69, I2_67, I3_67, I4_69] with visit as a fixed effect and AR(1) (first order auto-regressive) as covariance structure. The HbA1c baseline and HRSD-17 baseline serve as control variables. HbA1c baseline is the value at visit S1 [S1_77]. If the HbA1c value at baseline is missing, the HbA1c at visit SC [SC_118_1/2] will be used. HRSD-17 baseline is the latest value before treatment start at visit P0 [p0_hrsdsc].

The results will be displayed by means of p-value, estimate, 95%-confidence interval and effect size.

The results will be displayed for the Randomized Population [Randomized], Safety Population [Safety] and ITT Population [ITT].

Tables 6.9.1.2.1/2/3

6.9.1.3 Improvement of glycaemic control [Imp_hba1c_L4], i.e. decrease of at least 1% in HbA1c value compared to baseline (visit S1) [S1_77], corresponding to the former primary parameter (before the protocol was amended).

Analyses:

1. A logistic regression model will be performed controlled for HRSD-17 baseline and HbA1c baseline. HbA1c baseline is the value at visit S1 [S1_77]. If the HbA1c value at baseline is missing, the HbA1c at screening visit [SC_118_1/2] will be used. HRSD-17 baseline is the latest value before treatment start at visit P0 [p0_hrsdsc].
2. Correlation analyses (Pearson) will be performed to identify further covariates (those with a p-value < .10 will be included in the logistic regression model) with the variables stated above: age [Age], sex [SC_14], coordinating institution [K_Zentrum], diabetes type [SC_54], Late complications [DiabComp], Macrovascular complications [Comorb], education years [Education], continuous income [Income], single/recurrent

episode(s) [p0_20/(p0_21,p0_22)] and comorbidity of other mental disorders [CoMenDis].

The results are displayed by estimates, p-values and 95% confidence intervals.

The differences in HbA1c values to baseline [SC_118_1/2 or S1_77 respectively] over time [S4_67, I1_69, I2_67, I3_67, I4_69] will be displayed by descriptive statistics (n, mean, standard deviation, median, interquartile-range (IQA), minimum and maximum), and exploratory p-values of the t-test between treatment groups [S1_12] and effect sizes will be shown.

The results will be displayed for the Randomized Population [Randomized], Safety Population [Safety] and ITT Population [ITT].

Tables 6.9.1.3.1.1/2/3, 6.9.1.3.2.1/2/3, 6.9.1.3.3.1/2/3, 6.9.1.3.4.1/2/3

6.9.1.4 Remission of depression [p2_1_hrsdRem], i.e. not fulfilling the DSM-IV-TR criteria for depression according to the SKID [P2_19] and depression score Hamilton Rating Scale - Interview ≤ 7 [P2_hrsdsc].

Analyses for Remission:

1. A logistic regression model will be performed controlled for HRSD-17 baseline and HbA1c baseline. HbA1c baseline is the value at visit S1 [S1_77]. If the HbA1c value at baseline is missing, the HbA1c at visit SC [SC_118_1/2] will be used. HRSD-17 baseline is the latest value before treatment start at visit P0 [p0_hrsdsc].
2. Correlation analyses (Pearson) will be performed to identify further covariates (those with a p-value < .10 will be included in the logistic regression model) with the variables stated above: age [Age], sex [SC_14], coordinating institution [K_Zentrum], diabetes type [SC_54], Late complications [DiabComp], Macrovascular complications [Comorb], education years [Education], continuous income [Income], single/recurrent episode(s) [p0_20/(p0_21,p0_22)] and comorbidity of other mental disorders [CoMenDis].

The results are displayed by estimates, p-values, 95% confidence intervals and effect sizes (odds ratios).

The remission of depression at Visit P2 [p2_1_hrsdRem] will be displayed by descriptive statistics (n, percentage) and exploratory p-values of the Chi-Square test between treatment groups [S1_12] will be shown.

The results will be displayed for the Randomized Population [Randomized], Safety Population [Safety] and ITT Population [ITT].

Tables 6.9.1.4.1.1/2/3, 6.9.1.4.2.1/2/3, 6.9.1.4.3.1/2/3, 6.9.1.4.4.1/2/3

6.9.1.5 Improvement of depression [P2_1_hrsdImp], i.e. a reduction of the HRSD-17-score compared to baseline [p0_hrsdsc] by at least 50%.

Analyses for Improvement:

1. A logistic regression model will be performed controlled for HRSD-17 baseline and HbA1c baseline. HbA1c baseline is the value at visit S1 [S1_77]. If the HbA1c value at baseline is missing, the HbA1c at visit SC [SC_118_1/2] will be used. HRSD-17 baseline is the latest value before treatment start at visit P0 [p0_hrsdsc].
2. Correlation analyses (Pearson) will be performed to identify further covariates (those with a p-value < .10 will be included in the logistic regression model) with the variables stated above: age [Age], sex [SC_14], coordinating institution

[K_Zentrum], diabetes type [SC_54], Late complications [DiabComp], Macrovascular complications [Comorb], education years [Education], continuous income [Income], single/recurrent episode(s) [p0_20/(p0_21,p0_22)] and comorbidity of other mental disorders [CoMenDis].

The results are displayed by estimates, p-values, 95% confidence intervals and effect sizes (odds ratios).

The improvement of depression over time [P1_1_hrsdImp, P2_1_hrsdImp] and the responders over time [P1_1_hrsdtre, P2_1_hrsdtre] (definition see below) will be displayed by descriptive statistics (n, percentage) and exploratory p-values of the Chi-Square test between treatment groups [S1_12] will be shown.

The results will be displayed for the Randomized Population [Randomized], Safety Population [Safety] and ITT Population [ITT].

Tables 6.9.1.5.1.1/2/3, 6.9.1.5.2.1/2/3, 6.9.1.5.3.1/2/3, 6.9.1.5.4.1/2/3

Responder after the long term phase: [P2_1_hrsdtre], i.e. a reduction of the HRSD-17-score compared to baseline [p0_hrsdsc] by at least 50% or depression score Hamilton Rating Scale -Interview ≤ 7 [P2_hrsdsc].

Analyses:

1. A logistic regression will be done with responder after the long term phase [P2_1_hrsdtre] as dependent variable will be explored for treatment differences [S1_12]. The analysis will be controlled for baseline HbA1c [s1_77] and HRSD-17 [p0_hrsdsc]. If the HbA1c value at baseline is missing, the HbA1c at visit SC [SC_118_1/2] will be used. HRSD-17 Baseline is the latest value before treatment start at visit P0 [p0_hrsdsc].
2. Correlation analyses (Pearson) will be performed to identify further covariates (those with a p-value < .10 will be included in the logistic regression model) with the variables stated above: age [Age], sex [SC_14], coordinating institution [K_Zentrum], diabetes type [SC_54], Late complications [DiabComp], Macrovascular complications [Comorb], education years [Education], continuous income [Income], single/recurrent episode(s) [p0_20/(p0_21,p0_22)] and comorbidity of other mental disorders [CoMenDis].

The results are displayed by estimates, p-values, 95% confidence intervals and effect sizes (odds ratios) for the Randomized population [Randomized].

Tables 6.9.1.5.5.1.1, 6.9.1.5.5.2.1

6.9.1.6 Improvement of Health related Quality of Life, i.e. change in generic HRQoL as assessed per SF-36 from baseline (visit P0) to end of the long-term-phase (visit P2) (mental component: [P2_SF_sMH minus P0_SF_sMH], physical component: P2_SF_sPH minus P0_SF_sPH]). Both scores will be analysed separately.

Analyses:

1. An ANCOVA will be performed controlled for the baseline HbA1c and baseline SF-36 scores (visit P0) [P0_SF_sMH or P0_SF_sPH respectively]. HbA1c baseline is the value at visit S1 [S1_77]. If the HbA1c value at baseline is missing, the HbA1c at visit SC [SC_118_1/2] will be used.

2. Correlation analyses (Pearson) will be performed to identify further covariates (those with a p-value < .10 will be included in the regression model) with the variables stated above: HRSD-17 baseline, age [Age], sex [SC_14], coordinating institution [K_Zentrum], diabetes type [SC_54], Late complications [DiabComp], Macrovascular complications [Comorb], education years [Education], continuous income [Income], single/recurrent episode(s) [p0_20/(p0_21,p0_22)] and comorbidity of other mental disorders [CoMenDis]. HRSD-17 baseline is the latest value before treatment start at visit P0 [p0_hrsdsc].

The results are displayed by estimates, p-values, 95% confidence intervals and effect sizes.

The results will be displayed for the Randomized Population [Randomized], Safety Population [Safety] and ITT Population [ITT].

Tables 6.9.1.6.1.1.1/2/3, 6.9.1.6.1.2.1/2/3, 6.9.1.6.1.3.1/2/3, 6.9.1.6.2.1.1/2/3, 6.9.1.6.2.2.1/2/3, 6.9.1.6.2.3.1/2/3

6.9.1.7 Improvement of Diabetes related distress, i.e. change regarding problems in daily living with diabetes as assessed per PAID from baseline (Visit P0) to end of the long-term-phase (visit P2) [P2_1_x_paisc minus P0_1_x_paisc].

Analyses:

1. An ANCOVA will be performed controlled for baseline HbA1c-value [S1_77] and baseline PAID score (visit P0) [P0_1_x_paisc]. If the HbA1c value at baseline is missing, the HbA1c at visit SC [SC_118_1/2] will be used.
2. Correlation analyses (Pearson) will be performed to identify further covariates (those with a p-value < .10 will be included in the regression model) with the variables stated above: HRSD-17 baseline [p0_hrsdsc], age [Age], sex [SC_14], coordinating institution [K_Zentrum], diabetes type [SC_54], Late complications [DiabComp], Macrovascular complications [Comorb], education years [Education], continuous income [Income], single/recurrent episode(s) [p0_20/(p0_21,p0_22)] and comorbidity of other mental disorders [CoMenDis]. HRSD-17 baseline is the latest value before treatment start at visit P0 [p0_hrsdsc].

The results are displayed by estimates, p-values, 95% confidence intervals and effect sizes.

The PAID scores over time [P0_1_x_paisc, P1_1_x_paisc, P2_1_x_paisc] as well as changes versus Visit P0 will be displayed by descriptive statistics (n, mean, standard deviation, median, interquartile-range (IQA), minimum and maximum) and exploratory p-values of the t-test and the effect sizes for the absolute values and the changes to baseline at Visits P1 and P2 between treatment groups [S1_12] will be shown.

The results will be displayed for the Randomized Population [Randomized], Safety Population [Safety] and ITT Population [ITT].

Tables 6.9.1.7.1.1/2/3, 6.9.1.7.2.1/2/3, 6.9.1.7.3.1/2/3, 6.9.1.7.4.1.1/2/3, 6.9.1.7.4.2.1/2/3

6.9.1.8 Change of depression scores with repeated measurements

Another analysis of the outcome change of depression score compared to HRSD-17 baseline [p0_hrsdsc] will be done by a mixed model with repeated measurements incorporating all HRSD-17 measurements with visit [p1_hrsdsc, p2_hrsdsc] as a fixed effect and AR(1) (first order auto-regressive) as covariance structure. The HbA1c and HRSD-17 at baseline serve as control variables. HbA1c baseline is the value at visit S1 [S1_77]. If the HbA1c value at baseline is missing, the HbA1c at visit SC [SC_118_1/2] will be used. HRSD-17 baseline is the latest value before treatment start at visit P0 [p0_hrsdsc].

The results are displayed by estimates, p-values and 95% confidence intervals.

The results will be displayed for the Randomized Population [Randomized], Safety Population [Safety] and ITT Population [ITT].

Tables 6.9.1.8.1/2/3

6.9.1.9 Differences in further psychosocial variables

For the scores of HRSD-17 [P0_hrsdsc, P1_hrsdsc, P2_hrsdsc], SF-36 [P0/P1/P2_1_SF_PF, P0/P1/P2_1_SF_RP, P0/P1/P2_1_SF_BP, P0/P1/P2_1_SF_GH, P0/P1/P2_1_SF_V, P0/P1/P2_1_SF_SF, P0/P1/P2_1_SF_RE, P0/P1/P2_1_SF_MH, P0/P1/P2_SF_sMH, P0/P1/P2_SF_sPH], PAID [P0_1_x_paisc, P1_1_xpaisc, P2_1_x_paisc], ADS [Screening_1_x_adssc_final, S1_1_x_adssc_final, S4_1_x_adssc_final, L1_1_x_adssc_final, L2_1_x_adssc_final, L3_1_x_adssc_final, L4_1_x_adssc_final], SCL-K-9 [P0_1_x_sclk9sc], DWT-1 [P0_1_x_dt1sc, P1_1_x_dt1sc, P2_1_x_dt1sc] & 2 [P0_1_x_dt2sc, P1_1_x_dt2sc, P2_1_x_dt2sc], IPC-D1 [P0/P1/P2_1_x_intSC, P0/P1/P2_1_x_PhySC, P0/P1/P2_1_x_UnpSC, P0/P1/P2_1_x_FoChSC], IPC-D1 [P0/P1/P2_1_x_intSC, P0/P1/P2_1_x_PhySC, P0/P1/P2_1_x_UnpSC, P0/P1/P2_1_x_FoChSC], FKV-15 [P0/P1/P2_1_x_acsc, P0/P1/P2_1_x_dhsc, P0/P1/P2_1_x_disc, P0/P1/P2_1_x_exsc, P0/P1/P2_1_x_cosc], SE [P0_1_x_sesc, P1_1_x_sesc, P2_1_x_sesc], BIT [P0/P1/P2_1_x_fitsc, P0/P1/P2_1_x_rosc, P0/P1/P2_1_x_ehsc, P0/P1/P2_1_x_stsc, P0/P1/P2_1_x_hysc, P0/P1/P2_1_x_bitsc], SDSCA [P0/P1/P2_1_x_gdsc, P0/P1/P2_1_x_sdsc, P0/P1/P2_1_x_bgsc, P0/P1/P2_1_x_fcsc], PFUK-R [P0/P1/P2_1_emos_v, P0/P1/P2_1_emos_a, P0/P1/P2_1_entl_v, P0/P1/P2_1_entl_a, P0/P1/P2_1_glob_v, P0/P1/P2_1_glob_a], H-Skala [P0_1_x_hrasc, P1_1_x_hrasc, P2_1_x_hrasc] and K-INK [P0/P1/P2_1_x_apasc, P0/P1/P2_1_x_avasc, P0/P1/P2_x_tssc] descriptive statistics (n, mean, standard deviation, median, interquartile-range (IQA), minimum and maximum) of the absolute values and descriptive statistics (n, mean, standard deviation, median, interquartile-range (IQA), minimum and maximum) of the changes to baseline will be displayed by treatment group [S1_12].

Exploratory p-values of t-tests and ANCOVA will be provided and displayed together with the descriptive statistics per visit. Absolute and relative frequency counts of categories will be presented for each visit when there is a categorisation of the score. Effect sizes will be provided whenever appropriate.

The results will be displayed for the Randomized Population [Randomized], Safety Population [Safety] and ITT Population [ITT].

6.9.1.9.1 Hamilton Depression Scale (HRSD-17)

The HRSD-17 [P0_hrsdsc, P1_hrsdsc, P2_hrsdsc] is a sumscore of 17 items to assess the severity of depressive symptoms. It is calculated as the sumscore of all items and ranges from 0 to 52. If single items are missing, the score will be adjusted by multiplying the score by 52 divided by the maximum achievable score without the missing item. Afterwards the score will be rounded to the next integer number. The score will be set to missing, if more than 2 items are missing. The total score can be classified as none (0 to <8), mild (8 to <14), moderate (14 to <20), severe (20 to <26), and very severe (26 to 52) [P0_hrsdsc_kategorial_final, P1_hrsdsc_kategorial_final, P2_hrsdsc_kategorial_final].

The score will be summarised by the descriptive statistics n, mean, standard deviation, median, interquartile-range (IQA), minimum and maximum.

Exploratory p-values of baseline-adjusted ANCOVA (omitted for the changes) and t-test, effect sizes for the absolute values and the changes to baseline at visits P1 and P2 and Chi-Square tests between treatment groups [S1_12] will be presented per visit.

An ANCOVA for the HRSD-17 at Visit P2 [P2_hrsdsc] will be performed controlled for the HRSD-17 baseline [p0_hrsdsc] and HbA1c baseline. HbA1c baseline is the value at visit S1 [S1_77]. If the HbA1c value at baseline is missing, the HbA1c at visit SC [SC_118_1/2] will be used. HRSD-17 baseline is the latest value before treatment start at visit P0 [p0_hrsdsc]. The results are displayed by estimates, p-values, 95% confidence intervals and effect sizes.

The results will be displayed for the Randomized Population [Randomized], Safety Population [Safety] and ITT Population [ITT].

Tables 6.9.1.9.1.1.1/2/3, 6.9.1.9.1.2.1/2/3, 6.9.1.9.1.3.1/2/3 6.9.1.9.1.4.1/2/3

6.9.1.9.2 Short Form 36 (SF-36, German version, Bullinger et al., 1998)

The SF-36 is a 36-item score assessing the subject's quality of life. The SF 36 comprises eight scales covering physical functioning [P0/P1/P2_1_SF_PF], role-physical [P0/P1/P2_1_SF_RP], bodily pain [P0/P1/P2_1_SF_BP], general health [P0/P1/P2_1_SF_GH], vitality [P0/P1/P2_1_SF_V], social functioning [P0/P1/P2_1_SF_SF], role-emotional [P0/P1/P2_1_SF_RE] and mental health [P0/P1/P2_1_SF_MH].

In addition, two summary measures (Physical Health component score (PCS) [P0/P1/P2_SF_sPH] and Mental Health component score (MCS) [P0/P1/P2_SF_sMH]) can be computed. Missing items will be treated as recommended in the manual: If missing items occur, the subscore is calculated without the missing item. If more than 50% of the items of a subscore are missing, the subscore is set to missing (Bullinger et al., 1998).

Calculation of the Scores

First, the items will be recoded according to the table displayed in appendix 2.

Second, the eight subscales (Physical Functioning (Q3a to Q3j) [P0/P1/P2_1_SF_PF], Role Physical (Q4a to Q4d) [P0/P1/P2_1_SF_RP], Role Emotional (Q5a to Q5c) [P0/P1/P2_1_SF_RE], Vitality (Q9a, Q9e, Q9g and Q9i) [P0/P1/P2_1_SF_V], Mental Health (Q9b, Q9c, Q9d, Q9f and Q9h) [P0/P1/P2_1_SF_MH], Social Functioning (Q6, Q10) [P0/P1/P2_1_SF_SF], Bodily Pain (Q7, Q8) [P0/P1/P2_1_SF_BP], General Health (Q1, Q11a to Q11d)) [P0/P1/P2_1_SF_GH] are calculated by means of the recoded values. The resulting subscales vary from worst health (0) to best health (100).

The (interim) subscales are defined as follows:

**(Interim) Subscale**	**Calculation**
Physical Functioning	Mean (Q3a to Q3j) * 50
Role Physical	Mean (Q4a to Q4d) * 100
Role Emotional	Mean (Q5a to Q5c) * 100
Vitality	Mean (Q9a, Q9e, Q9g and Q9i) * 20
Mental Health	Mean (Q9b, Q9c, Q9d, Q9f and Q9h) * 20
Social Functioning	Mean (Q6, Q10) * 25

Bodily Pain	Mean (Q7, Q8) * 20
General Health	Mean (Q1, Q11a to Q11d) * 25

These scale values were transformed into z-scores based on German population means and standard deviations considering age and gender. This approach was chosen due to the following reasons: First, HrQoL decreases with increasing age at different rates for men and women. Second, as population means and standard deviations differ between the subscale scores, the comparisons of the eight subscales were facilitated by the use of z-scores.

The two summary measures (Physical and Mental Component Scores, PCS/MCS) will be computed according to Ware, Kosinski, Bayliss, McHorney, Rogers, & Raczek (1994). To facilitate the comparability with the SF-36 subscales, for both components z-scores will be used instead of T-values. The summary measures will only be computed, if all subscales are available, i.e. no subscale is missing.

The Physical Health score [P0/P1/P2_SF_sPH] will be calculated by

Physical Health = (z-score of Physical Functioning [P0/P1/P2_1_SF_PF_Z]*0.42402) + (z-score of Role Physical [P0/P1/P2_1_SF_RP_Z]*0.35119) + (z-score of Bodily Pain [P0/P1/P2_1_SF_BP_Z]* 0.31754) + (z-score of Social Functioning [P0/P1/P2_1_SF_SF_Z]*-0.00753) + (z-score of Mental Health [P0/P1/P2_1_SF_MH_Z]*-0.22069) + (z-score of Role Emotional [P0/P1/P2_1_SF_RE_Z]*-0.19206) + (z-score of Vitality [P0/P1/P2_1_SF_V_Z]*0.02877) + (z-score of General Health [P0/P1/P2_1_SF_GH_Z]*0.24954)

The Mental Healthscore [P0/P1/P2_SF_sMH] will be calculated by

Mental Health = (z-score of Physical Functioning [P0/P1/P2_1_SF_PF_Z]*-0.22999) + (z-score of Role Physical [P0/P1/P2_1_SF_RP_Z]*-0.12329) + (z-score of Bodily Pain [P0/P1/P2_1_SF_BP_Z]*-0.09731) + (z-score of Social Functioning [P0/P1/P2_1_SF_SF_Z]*0.26876) + (z-score of Mental Health [P0/P1/P2_1_SF_MH_Z]*0.48581) + (z-score of Role Emotional [P0/P1/P2_1_SF_RE_Z]*0.43407) + (z-score of Vitality [P0/P1/P2_1_SF_V_Z]*0.23407) + (z-score of General Health [P0/P1/P2_1_SF_GH_Z]*-0.01571)

The scores as well as changes versus Visit P0 will be summarised by the descriptive statistics n, mean, standard deviation, median, interquartile-range (IQA), minimum and maximum.

Exploratory p-values of baseline-adjusted ANCOVA (omitted for the changes) and t-test per visit and the effect size for the absolute values and the changes to baseline at Visits P1 and P2 between treatment groups will be presented.

The results will be displayed for the Randomized Population [Randomized], Safety Population [Safety] and ITT Population [ITT].

Tables 6.9.1.9.2.1.1/2/3, 6.9.1.9.2.2.1/2/3

6.9.1.9.3 Problem Areas in Diabetes Survey (PAID)

The PAID [P0_1_x_paisc, P1_1_x_paisc, P2_1_x_paisc] is composed of 20 items (0=no problem, 1=rather small problem, 2=moderate problem, 3=rather big problem, 4=big problem) measuring diabetes related distress. The total score is the mean of all non-missing single items multiplied by 25. This means that the total score ranges from 0 to 100. The score will be set to missing, if more than 3 items are missing.

The score as well as changes versus Visit P0 will be summarised by the descriptive statistics n, mean, standard deviation, median, interquartile-range (IQA), minimum and maximum.

Exploratory p-values of baseline-adjusted ANCOVA (omitted for the changes) and t-test per visit and the effect size for the absolute values and the changes to baseline at Visits P1 and P2 between treatment groups will be presented.

The results will be displayed for the Randomized Population [Randomized], Safety Population [Safety] and ITT Population [ITT].

Tables 6.9.1.9.3.1.1/2/3, 6.9.1.9.3.2.1/2/3

6.1.9.1.4 Allgemeine Depressions Skala (ADS, German translation of the CES-D)

The ADS-score [Screening_1_x_adssc_final, S1_1_x_adssc_final, S4_1_x_adssc_final, L1_1_x_adssc_final, L2_1_x_adssc_final, L3_1_x_adssc_final, L4_1_x_adssc_final] is a 20-item score (0=”rarely or never”, 1=”sometimes”, 2=”often”, 3=”mostly”) that assesses symptoms of depression and ranges from 0 to 60. For calculating the total score the items 4, 8, 12 and 16 will be reversed. The total score is the mean of all non-missing single items multiplied by 20. The total score will be categorised as follows 0 – 17: Healthy subjects, 18-22: Subclinical, >22: Clinically relevant depression. [Screening_1_x_adssc_kategorial, S1_1_x_adssc_kategorial, S4_1_x_adssc_kategorial, L1_1_x_adssc_kategorial, L2_1_x_adssc_kategorial, L3_1_x_adssc_kategorial, L4_1_x_adssc_kategorial]. The score will be set to missing, if more than 3 items are missing.

The continuous ADS score as well as changes versus Visit S1 will be presented by mean, SD, median, interquartile-range (IQA), minimum and maximum.

The ADS score categories will be presented by number and percentages.

Exploratory p-values of the baseline-adjusted ANCOVA (omitted for the changes) and t-test and Chi-Square test per visit and the effect size for the continuous score for the absolute values and the changes to baseline at Visits S4, L1, L2, L3 and L4 between treatment [S1_12] groups will be presented.

The baseline results will be displayed for the Randomized Population [Randomized], Safety Population [Safety], ITT Population [ITT] and PP Population [PP]. Post-baseline results will be displayed for the Randomized Population [Randomized], Safety Population [Safety] and ITT Population [ITT].

Tables 6.9.1.9.4.1.1/2/3, 6.9.1.9.4.2.1/2/3, 6.9.1.9.4.3.1/2/3

6.9.1.9.5 SCL-K-9

The SCL-K-9 [P0/P1/P2_1_x_sclk9sc] is a 9-item score (0=”not at all” to 4=”very strong”) assessing the symptoms of psychopathology. The total score is the mean of all non-missing single items. The score will be set to missing, if more than 1 item is missing.

The score as well as changes versus Visit P0 will be summarised by the descriptive statistics n, mean, standard deviation, median, interquartile-range (IQA), minimum and maximum.

Exploratory p-values of the baseline-adjusted ANCOVA (omitted for the changes) and t-test per visit and the effect size for the absolute values and the changes to baseline at Visits P1 and P2 between treatment groups [S1_12] will be presented.

The results will be displayed for the Randomized Population [Randomized], Safety Population [Safety] and ITT Population [ITT].

Tables 6.9.1.9.5.1.1/2/3, 6.9.1.9.5.2.1/2/3

6.1.9.1.6 Diabetes Wissens Test: Typ 1 (DWT: Type 1)

The DWT: Type 1 [P0_1_x_dt1sc, P1_1_x_dt1sc, P2_1_x_dt1sc] consists of 30-item scores testing the subjects' knowledge of diabetes type1. The total score is the sum of all 30 items. If an item is missing, it will be considered as filled out wrongly.

The following tables display the correct answers for the DWT type 1:

Item Number	Correct Answer
2	a,b,c
3	a,b,c
4	a
5	c
6	b,c
7	a,b
8	a,b
9	a,b
10	a,b
11	b
12	b,c
13	a,b,c
14	a,b,c
15	a,b
16	a,b
17	b,c
18	a,b,c
19	b,c
20	a,,c
21	b,c
22	b,c
23	b,c
24	a,b
25	b,c
26	b,c
27	a,b
28	a,b
29	a,b
30	a,c
31	a,b

The test results as well as changes versus Visit P0 will be summarised by the descriptive statistics n, mean, standard deviation, median, interquartile-range (IQA), minimum and maximum.

Exploratory p-values of the baseline-adjusted ANCOVA (omitted for the changes) and t-test per visit and the effect size for the absolute values and the changes to baseline at the Visits P1 and P2 between treatment groups [S1_12] will be presented.

The results will be displayed for the Randomized Population [Randomized], Safety Population [Safety] and ITT Population [ITT].

Tables 6.9.1.9.6.1.1/2/3, 6.9.1.9.6.2.1/2/3

6.9.1.9.7 Diabetes Wissens Test: Typ 2 (DWT: Type 2)

The DWT: Type 2 [P0_1_x_dt2sc, P1_1_x_dt2sc, P2_1_x_dt2sc] consists of 17-items testing the subjects' knowledge of diabetes type 2. The total score is the sum of the first 14 items. If an item is missing, it will be considered as filled out wrongly. The last 3 items (2 of the three items consist of 2 questions) [P0_356, P0_357, P0_358, P0_359, P0_360, P1_193, P1_194, P1_195, P1_196, P1_197, P2_246, P2_247, P2_248, P2_249, P2_250] are dismissed when calculating the total score and are interpreted separately.

The following tables display the correct answers for the DWT type 2:

Item Number	Correct Answer
1	b,c
2	a,b,c,d
3	b,d
4	a,c,d
5	a,d
6	a,c
7	b,d
8	b,d
9	a,b
10	c,d
11	a,b,c
12	a,c,d
13	b,d
14	a,c

The test results as well as changes versus Visit P0 for the total score will be summarised by the descriptive statistics n, mean, standard deviation, median, interquartile-range (IQA), minimum and maximum.

Exploratory p-values of the baseline-adjusted ANCOVA (omitted for the changes) and t-test per visit and the effect size for the absolute values and the changes to baseline at the Visits P1 and P2 between treatment groups [S1_12] will be presented for the total score. For the last 3 items changes will not be analysed.

The results will be displayed for the Randomized Population [Randomized], Safety Population [Safety] and ITT Population [ITT].

Tables 6.9.1.9.7.1.1.1/2/3, 6.9.1.9.7.1.2.1/2/3, 6.9.1.9.7.2.1/2/3

6.9.1.9.8 IPC Diabetes-Fragebogen (IPC-D1)

The IPC-D1 is a 29-item score (1="not at all" to 6="very exact") assessing disease specific control convictions. There exist 4 subscores (Internality (items 2, 3, 6, 15, 17, 20, 22, and 27) [P0/P1/P2_1_x_intSC], Physician related externality (items 5, 9, 10, 11, 12, 16, and 29) [P0/P1/P2_1_x_PhySC], Unpredictability (items 1, 4, 7, 13, 14, 19, 25, 26, and 28) [P0/P1/P2_1_x_UnpSC], Fortune and Chance (items 8, 18, 21, 23, and 24) [P0/P1/P2_1_x_FoChSC]. The subscore is the mean of all non-missing single items

multiplied by the number of items of each subscore. Each subscore will be set to missing, if more than 1 of the items is missing.

The scores as well as changes versus Visit P0 will be summarised by the descriptive statistics n, mean, standard deviation, median, interquartile-range (IQA), minimum and maximum.

Exploratory p-values of the baseline-adjusted ANCOVA (omitted for the changes) and t-test per visit and the effect sizes for the absolute values and the changes to baseline at Visits P1 and P2 between treatment groups [S1_12] will be presented.

The results will be displayed for the Randomized Population [Randomized], Safety Population [Safety] and ITT Population [ITT].

Tables 6.9.1.9.8.1.1/2/3, 6.9.1.9.8.2.1/2/3

6.9.1.9.9 Freiburger Fragebogen zur Krankheitsverarbeitung (FKV-15)

The FKV-15 consists of 15 items scored from 1 to 5 (1=”completely disagree” to 5=”completely agree”) and assesses the patient’s coping with disease. The FKV-15 has 5 subscores: Active problem-focused coping (Items 4,9,11,15) [P0/P1/P2_1_x_acsc], Depressive coping (items 2,3,6,10,14) [P0/P1/P2_1_x_dhsc], Distraction (items 5,8) [P0/P1/P2_1_x_disc],Trivializing (items 7,13) [P0/P1/P2_1_x_exsc], Doctor-centered coping (items 1,12) [P0/P1/P2_1_x_cosc]. The subscores are computed using the mean of the respective non-missing single items. The subscores for Active problem-focused coping and Depressive coping will be set to missing, if more than 1 item is missing. For the other subscores no missing items are allowed.

The scores as well as changes versus Visit P0 will be summarised by the descriptive statistics n, mean, standard deviation, median, interquartile-range (IQA), minimum and maximum.

Exploratory p-values of the baseline-adjusted ANCOVA (omitted for the changes) and t-test per visit and the effect size for the absolute values and the changes to baseline at Visits P1 and P2 between treatment groups [S1_12] will be presented.

The results will be displayed for the Randomized Population [Randomized], Safety Population [Safety] and ITT Population [ITT].

Tables 6.9.1.9.9.1.1/2/3, 6.9.1.9.9.2.1/2/3

6.9.1.9.10 Rosenberg-Scale (SE)

The SE [P0_1_x_sesc, P1_1_x_sesc, P2_1_x_sesc] is composed of 10 questions assessing the patients’ self-esteem. The single items will be coded from 0=”does not apply” to 3=”completely applies”. For calculating the total score the items 2, 5, 6, 8 and 9 will be reversed. The total score is the mean of all non-missing single items multiplied by 10. The total score will be set to missing, if more than 2 items are missing.

The score as well as changes versus Visit P0 will be summarised by the descriptive statistics n, mean, standard deviation, median, interquartile-range (IQA), minimum and maximum.

Exploratory p-values of the baseline-adjusted ANCOVA (omitted for the changes) and t-test per visit and the effect size for the absolute values and the changes to baseline at Visits P1 and P2 between treatment groups [S1_12] will be presented.

The results will be displayed for the Randomized Population [Randomized], Safety

Population [Safety] and ITT Population [ITT].

Tables 6.9.1.9.10.1.1/2/3, 6.9.1.9.10.2.1/2/3

6.9.1.9.11 Barriers to Insulin Therapy Questionnaire (BIT)

The BIT consists of 14 items coded from 1="completely disagree" to 10=" completely agree".

There exist 5 subscores (Fear of injections and self-testing (item 1 to 3) [P0/P1/P2_1_x_fitsc], Expectations regarding positive insulin related outcomes (item 4 to 6) [P0/P1/P2_1_x_rosc], Expected hardship from insulin therapy (item 7 to 9) [P0/P1/P2_1_x_ehsc], Stigmatization by insulin injections (item 10 to 12) [P0/P1/P2_1_x_stsc], Fear of hypoglycaemia (item 13 and 14) [P0/P1/P2_1_x_hysc]) and a total score [P0/P1/P2_1_x_bitsc]. For the total score items 4, 5, and 6 will be reversed. The scores will be calculated taking the mean of the non-missing items. The total score will be set to missing, if more than 2 items are missing. The subscores Fear of injections and self-testing, Expectations regarding positive insulin related outcomes, Expected hardship from insulin therapy, Stigmatization by insulin injections will be set to missing, if more than 1 item is missing. For Fear of hypoglycaemia no missing items are allowed.

The scores as well as changes versus Visit P0 will be summarised by the descriptive statistics n, mean, standard deviation, median, interquartile-range (IQA), minimum and maximum.

Exploratory p-values of the baseline-adjusted ANCOVA (omitted for the changes) and t-test per visit and the effect sizes for the absolute values and the changes to baseline at the Visits P1 and P2 between treatment groups [S1_12] will be presented.

The results will be displayed for the Randomized Population [Randomized], Safety Population [Safety] and ITT Population [ITT].

Tables 6.9.1.9.11.1.1/2/3, 6.9.1.9.11.2.1/2/3

6.9.1.9.12 Summary of Diabetes Self Care Activities Measure (SDSCA)

A correlation analysis (Pearson) of the improvement in depression at Visit P2 and the HbA1c value at Visit L4 will be performed for each treatment group separately. If this is the case, the analysis of the SDSCA will be investigated from a different point of view. If the SDSCA will increase more in the CBT than in the SER group, the SDSCA will be considered as a mediator variable between the HRSD-17 and the HbA1c.

The results will be displayed for the Randomized Population [Randomized], Safety Population [Safety] and ITT Population [ITT].

Table 6.9.1.9.12.1

The SDSCA is composed of 10 items measuring (item 1 to 9: 0 to 7, item 10: Yes/No and the number of cigarettes) measuring diabetes self-care measurements. There is no total score. However, there exist 6 subscores (General Diet, Specific Diet, Exercise, Blood-Glucose Testing, Foot-Care, Smoking Status).

The General Diet Subscore [P0/P1/P2_1_x_gdsc] is the mean value of the first two items.

The Specific Diet Subscore [P0/P1/P2_1_x_sdsc] is the mean value of the items 3 and the reversed item 4.

The Exercise Subscore [P0_366, P1_203, P2_256] is the value of the fifth item.

The Blood-Glucose Testing Subscore [P0/P1/P2_1_x_bgsc] is the mean value of the sixth and seventh item.

The Foot-Care Subscore [P0/P1/P2_1_x_fcsc] is the mean value of item 8 and item 9.

The Smoking Status is smoking yes/no (no=0, yes=1) [P0_371, P1_208, P2_261] and the smoked number of cigarettes [P0_372, P1_209, P2_262] analysed separately.

Each subscore will be set to missing, if one or both items are missing.

The scores as well as changes versus Visit P0 will be summarised by the descriptive statistics n, mean, standard deviation, median, interquartile-range (IQA), minimum and maximum.

Exploratory p-values of the baseline-adjusted ANCOVA (omitted for the changes) and t-test per visit and the effect size for the absolute values and the changes to baseline at the Visits P1 and P2 between treatment groups [S1_12] will be presented.

The results will be displayed for the Randomized Population [Randomized], Safety Population [Safety] and ITT Population [ITT].

Tables 6.9.1.9.12.2.1.1/2/3, 6.9.1.9.12.2.2.1/2/3, 6.9.1.9.12.2.3.1/2/3

6.9.1.9.13 Fragebogen zur Perzipierten Familialen Unterstützung und Kommunikation – Revision (PFUK-R)

The PFUK-R consists of two subscores with items ranging from 1 to 5 assessing the subject’s family support.

The following subscores can be calculated: The first score (global availability) [P0/P1/P2_1_glob_v] consists of 12 items (PFUKR01A, PFUKR06A, PFUKR09A, PFUKR10A, PFUKR12A, PFUKR13A, PFUKR18A, PFUKR14A, PFUKR20A, PFUKR24A, PFUKR25A, PFUKR26A) and the second score (global appropriateness) [P0/P1/P2_1_glob_a] consists of 12 items (PFUKR01B, PFUKR06B, PFUKR09B, PFUKR10B, PFUKR12B, PFUKR13B, PFUKR18B, PFUKR14B, PFUKR20B, PFUKR24B, PFUKR25B, PFUKR26B). Both scores are the mean of the respective items. The subscores will be set to missing, if more than 2 items are missing.

There are 4 additional subscores: Score A (emotional support availability) [P0/P1/P2_1_emos_v] will be derived from 7 items (PFUKR01A, PFUKR06A, PFUKR09A, PFUKR10A, PFUKR12A, PFUKR13A, PFUKR18A). Score B (emotional support appropriateness) [P0/P1/P2_1_emos_a] will be derived from 8 items (PFUKR01B, PFUKR06B, PFUKR09B, PFUKR10B, PFUKR12B, PFUKR13B, PFUKR18B). Score C (overprotective support availability) [P0/P1/P2_1_entl_v] will be derived from 5 items (PFUKR14A, PFUKR20A, PFUKR24A, PFUKR25A, PFUKR26A). Score D (overprotective support appropriateness) [P0/P1/P2_1_entl_a] will be derived from 5 items (PFUKR14B, PFUKR20B, PFUKR24B, PFUKR25B, PFUKR26B). All additional subscores are the mean of the respective items. The score will be set to missing, if more than 1 item is missing.

The scores as well as changes versus Visit P0 will be summarised by the descriptive statistics n, mean, standard deviation, median, interquartile-range (IQA), minimum and maximum.

Exploratory p-values of the baseline-adjusted ANCOVA (omitted for the changes) and t-test per visit and the effect size for the absolute values and the changes to baseline at the Visits P1 and P2 between treatment groups [S1_12] will be presented.

The results will be displayed for the Randomized Population [Randomized], Safety

Population [Safety] and ITT Population [ITT].

Tables 6.9.1.9.13.1.1/2/3, 6.9.1.9.13.2.1/2/3

6.9.1.9.14 Hopelessness Scale (H-Skala)

The H-Skala [P0_1_x_hrasc, P1_1_x_hrasc, P2_1_x_hrasc] is a 10-item score (1=”completely wrong” to 6=”completely right”) assessing the subject’s hopelessness. The total score is the mean of all non-missing single items (the items 1, 3, 5, 6, and 7 will be reversed) multiplied by 10. The total score will be set to missing, if more than 2 items are missing.

The score as well as changes versus Visit P0 will be summarised by the descriptive statistics n, mean, standard deviation, median, interquartile-range (IQA), minimum and maximum.

Exploratory p-values of the baseline-adjusted ANCOVA (omitted for the changes) and t-test per visit and the effect size for the absolute values and the changes to baseline at the Visits P1 and P2 between treatment groups [S1_12] will be presented.

The results will be displayed for the Randomized Population [Randomized], Safety Population [Safety] and ITT Population [ITT].

Tables 6.9.1.9.14.1.1/2/3, 6.9.1.9.14.2.1/2/3

6.9.1.9.15 Inkongruenzfragebogen (K-INK)

The K-INK analyses the motivational incongruity and consists of 23 items in total and two subscores (Approach Aims (items 1 to 14) [P0/P1/P2_1_x_apasc], Avoidance Aims (items 15 to 23) [P0/P1/P2_1_x_avasc]). The approach aims score is 6 minus the mean value of the first 14 items and the avoidance aims score is the mean value of the items 15 to 23. The total score [P0/P1/P2_x_tssc] is the mean of the approach aims score and the avoidance aims score. The approach aims subscore will be set to missing, if more than 3 items are missing. The avoidance aims subscore will be set to missing, if more than 2 items are missing. For the total score both subscores have to be present.

The scores as well as changes versus Visit P0 will be summarised by the descriptive statistics n, mean, standard deviation, median, interquartile-range (IQA), minimum and maximum.

Exploratory p-values of the baseline-adjusted ANCOVA (omitted for the changes) and t-test per visit and the effect size for the absolute values and the changes to baseline at the Visits P1 and P2 between treatment groups [S1_12] will be presented.

The results will be displayed for the Randomized Population [Randomized], Safety Population [Safety] and ITT Population [ITT].

Tables 6.9.1.9.15.1.1/2/3, 6.9.1.9.15.2.1/2/3

6.9.2 Safety

Adverse events

Adverse Events will be coded by MedDRA. Adverse events could be entered twice in the CRF (by the diabetologist and the psychologist). Entries from both were pooled together and

double entries were removed by checking the coded terms [AE_Ereignis_No_1 – AE_Ereignis_No_26].

Number and percentage of AEs and number and percentage of subjects with AEs will be presented for each occurring system organ class and for each preferred term within system organ class by treatment group and with a total column. Additionally, this will be done for relationship [AE_Kausalitaet_No_1 – AE_Kausalitaet_No_26] (AEs with possible, likely or definite relationship will be considered as related AEs), intensity [AE_Intensitaet_No_1 – AE_Intensitaet_No_26] and seriousness [AE_SAE_No_1 – AE_SAE_no_26] of AEs.

The results will be displayed for the Safety Population [Safety].

Tables 6.9.2.1, 6.9.2.2, 6.9.2.3, 6.9.2.4

6.10 Subgroup Analyses

6.10.1 Type of Diabetes

A subgroup analysis for type of diabetes [SC_54] will be done for the primary parameter. This will be done by including an interaction term of diabetes type and treatment group [S1_12] in the ANCOVA. Further covariates are baseline HbA1c [S1_77] and baseline HRSD-17 score [p0_hrsdsc]. If the HbA1c value at baseline is missing, the HbA1c at visit SC [SC_118_1/2] will be used. HRSD-17 baseline is the latest value before treatment start at visit P0 [p0_hrsdsc]. Statistical results comparing the subgroups within treatment will be displayed.

Tables: 6.10.1.9.6.1.1/2/3

The HbA1c values [SC_118_1/2, S1_77, S4_67, I1_69, I2_67, I3_67, I4_69] by treatment group [S1_12] within type of diabetes [SC_54], type of diabetes [SC_54] within treatment group [S1_12] and type of diabetes [SC_54] will be displayed by descriptive statistics (n, mean, standard deviation, median, interquartile-range (IQA), minimum and maximum) and they will be compared by exploratory p-values of t-tests for each visit.

Tables: 6.10.1.9.1.1/2/3, 6.10.1.9.2.1/2/3, 6.10.1.9.3.1/2/3, 6.10.1.9.4.1/2/3, 6.10.1.9.5.1/2/3

Descriptive statistics of demographic [see section 6.2], baseline medical [view section 6.3.2] and psychosocial characteristics [see section 6.3.1] will be displayed by type of diabetes [SC_54]. Exploratory p-values of t-tests and chi-square tests will be presented to examine differences between both types of diabetes.

Tables:

Demographic: 6.10.1.1.1.1/2/3, 6.10.1.1.2.1/2/3, 6.10.1.1.3.1/2/3

Baseline: 6.10.1.2.1.1/2/3, 6.10.1.2.2.1/2/3, 6.10.1.2.3.1/2/3, 6.10.1.2.4.1/2/3, 6.10.1.2.5.1.1/2/3, 6.10.1.2.5.2.1/2/3, 6.10.1.2.6.1/2/3, 6.10.1.2.7.1/2/3, 6.10.1.2.8.1/2/3, 6.10.1.2.9.1.1/2/3, 6.10.1.2.9.2.1/2/3, 6.10.1.2.9.3.1/2/3, 6.10.1.2.10.1.1/2/3, 6.10.1.2.10.2.1/2/3, 6.10.1.2.10.3.1/2/3, 6.10.1.2.10.4.1/2/3

Concomitant diseases: 6.10.1.3.1.1/2/3, 6.10.1.3.2.1/2/3

Concomitant medication: 6.10.1.4.1.1/2/3, 6.10.1.4.2.1/2/3

Psychosocial variables at baseline: 6.10.1.5.1.1/2/3, 6.10.1.5.2.1/2/3, 6.10.1.5.3.1/2/3, 6.10.1.5.4.1/2/3, 6.10.1.5.5.1/2/3, 6.10.1.5.6.1/2/3, 6.10.1.5.7.1/2/3, 6.10.1.5.8.1/2/3, 6.10.1.5.9.1/2/3, 6.10.1.5.10.1/2/3, 6.10.1.5.11.1/2/3, 6.10.1.5.12.1/2/3, 6.10.1.5.13.1/2/3

Remission of depression [P2_1_hrsdRem], improvement of depression [P1_1_hrsdImp, P2_1_hrsdImp] and HRSD-17 score over time [P0_hrsdsc, P1_hrsdsc, P2_hrsdsc] (mean,

standard deviation, interquartile-range (IQA), median, minimum and maximum for quantitative variables and absolute and relative frequencies for qualitative variables) will be displayed by treatment group [S1_12] within type of diabetes [SC_54], type of diabetes [SC_54] within treatment group [S1_12] and type of diabetes [SC_54]. Exploratory p-values of t-tests and chi-square tests will be presented to examine differences between both types of diabetes or treatment groups respectively.

Tables: 6.10.1.7.1.1/2/3, 6.10.1.7.2.1/2/3, 6.10.1.7.3.1/2/3, 6.10.1.7.4.1/2/3, 6.10.1.7.5.1/2/3, 6.10.1.8.1.1/2/3, 6.10.1.8.2.1/2/3, 6.10.1.8.3.1/2/3, 6.10.1.8.4.1/2/3, 6.10.1.8.5.1/2/3, 6.10.1.6.1.1/2/3, 6.10.1.6.2.1/2/3, 6.10.1.6.3.1/2/3, 6.10.1.6.4.1/2/3, 6.10.1.6.5.1/2/3

A logistic regression will be performed for remission of depression [p2_1_hrsdRem] controlled for the HRSD-17 baseline [p0_hrsdsc], HbA1c baseline, diabetes type [SC_54] and interaction of diabetes type [SC_54] and treatment group [S1_12]. HbA1c baseline is the value at visit S1 [S1_77]. If the HbA1c value at baseline is missing, the HbA1c at visit SC [SC_118_1/2] will be used. HRSD-17 baseline is the latest value before treatment start at visit P0 [p0_hrsdsc].

Tables: 6.10.1.10.1/2/3

HRSD-17 score at P2 [p2_hrsdsc]

Analyses:

1. An ANCOVA will be performed controlled for the baseline HRSD-17 baseline [p0_hrsdsc], HbA1c baseline, diabetes type [SC_54] and interaction of diabetes type and treatment group. HbA1c baseline is the value at visit S1 [S1_77]. If the HbA1c value at baseline is missing, the HbA1c at visit SC [SC_118_1/2] will be used. HRSD-17 baseline is the latest value before treatment start at visit P0 [p0_hrsdsc].
2. Correlation analyses (Pearson) will be performed to identify further covariates (those with a p-value < .10 will be included in the regression model) with the variables stated above: HRSD-17 baseline, age [Age], sex [SC_14], coordinating institution [K_Zentrum], diabetes type [SC_54], Late complications [DiabComp], Macrovascular complications [Comorb], education years [Education], continuous income [Income], single/recurrent episode(s) [p0_20/(p0_21,p0_22)] and comorbidity of other mental disorders [CoMenDis]. HRSD-17 baseline is the latest value before treatment start at visit P0 [p0_hrsdsc].

Tables: 6.10.1.11.1.1/2/3, 6.10.1.11.2.1/2/3, 6.10.1.11.3.1/2/3

The results will be displayed for the Randomized Population [Randomized], Safety Population [Safety] and ITT Population [ITT].

6.10.2 Adherence groups

A subgroup analysis for adherence group [Adherence_Randomised, Adherence_ITT] will be done for change in HbA1c (L4-S4) [l4_69- S4_67]. This will be done by including an interaction term of adherence group [Adherence_Randomised, Adherence_ITT] and treatment group [S1_12] in the ANCOVA. Further covariates are HbA1c at visit S4 [S4_67] and HRSD-17 score at visit P1 [p1_hrsdsc]. Statistical results comparing the subgroups within treatment will be displayed.

The results will be displayed for the Randomized Population [Randomized] and ITT Population [ITT].

Tables: 6.10.2.8.3.1/2

The HbA1c values [SC_118_1/2, S1_77, S4_67, I1_69, I2_67, I3_67, I4_69] by treatment group [S1_12] within adherence group [Adherence_Randomised], adherence group [Adherence_Randomised] within treatment group [S1_12] and adherence group [Adherence_Randomised] will be displayed by descriptive statistics (n, mean, standard deviation, median, interquartile-range (IQA), minimum and maximum) and they will be compared by exploratory p-values of t-tests for each visit.

The results will be displayed for the Randomized Population [Randomized] and ITT Population [ITT].

Tables: 6.10.2.8.1.1.1/2, 6.10.2.8.1.2.1/2, 6.10.2.8.1.3.1/2, 6.10.2.8.1.4.1/2, 6.10.2.8.1.5.1/2

The HbA1c values [SC_118_1/2, S1_77, S4_67, I1_69, I2_67, I3_67, I4_69] by treatment group [S1_12] within adherence group [Adherence_ITT] and adherence group [Adherence_ITT] will be displayed by descriptive statistics (n, mean, standard deviation, median, interquartile-range (IQA), minimum and maximum) and they will be compared by exploratory p-values of t-tests for each visit.

The results will be displayed for the ITT Population [ITT].

Tables: 6.10.2.8.2.1.2, 6.10.2.8.2.2.2, 6.10.2.8.2.3.2

Descriptive statistics of demographic [see section 6.2], baseline medical [see section 6.3.2] and psychosocial characteristics [see section 6.3.1], will be displayed by adherence groups [Adherence_Randomised]. Exploratory p-values of t-tests and chi-square tests will be presented to examine differences between both categories of adherence.
The results will be displayed for the Randomized Population [Randomized] and ITT Population [ITT].

Tables:

Demographic: 6.10.2.1.1.1/2, 6.10.2.1.2.1/2, 6.10.2.1.3.1/2

Baseline: 6.10.2.2.1.1/2, 6.10.2.2.2.1/2, 6.10.2.2.3.1/2, 6.10.2.2.4.1/2, 6.10.2.2.5.1.1/2, 6.10.2.2.5.2.1/2, 6.10.2.2.6.1/2, 6.10.2.2.7.1/2, 6.10.2.2.8.1/2, 6.10.2.2.9.1.1/2, 6.10.2.2.9.2.1/2, 6.10.2.2.9.3.1/2, 6.10.2.2.10.1.1/2, 6.10.2.2.10.2.1/2, 6.10.2.2.10.3.1/2, 6.10.2.2.10.4.1/2

Concomitant diseases: 6.10.2.3.1.1/2, 6.10.2.3.2.1/2

Concomitant medication: 6.10.2.4.1.1/2, 6.10.2.4.2.1/2

Remission of depression [P2_1_hrsdRem], improvement of depression [P1_1_hrsdImp, P2_1_hrsdImp] and HRSD-17 score [P0_hrsdsc, P1_hrsdsc, P2_hrsdsc] over time by treatment group [S1_12] within adherence group [Adherence_Randomised], adherence group [Adherence_Randomised] within treatment group [S1_12] and adherence group [Adherence_Randomised] will be displayed by descriptive statistics (mean, standard deviation, interquartile-range (IQA), median, minimum and maximum for quantitative variables and absolute and relative frequencies for qualitative variables) and will be compared by exploratory p-values of t-tests and chi-square tests.

The results will be displayed for the Randomized Population [Randomized] and ITT Population [ITT].

Tables: 6.10.2.6.1.1.1/2, 6.10.2.6.1.2.1/2, 6.10.2.6.1.3.1/2, 6.10.2.6.1.4.1/2, 6.10.2.6.1.5.1/2, 6.10.2.7.1.1.1/2, 6.10.2.7.1.2.1/2, 6.10.2.7.1.3.1/2, 6.10.2.7.1.4.1/2, 6.10.2.7.1.5.1/2, 6.10.2.5.1.1.1/2, 6.10.2.5.1.2.1/2, 6.10.2.5.1.3.1/2, 6.10.2.5.1.4.1/2, 6.10.2.5.1.5.1/2

Remission of depression [P2_1_hrsdRem], improvement of depression [P1_1_hrsdImp, P2_1_hrsdImp] and HRSD-17 score [P0_hrsdsc, P1_hrsdsc, P2_hrsdsc] over time by treatment group [S1_12] within adherence group [Adherence_ITT] and adherence group [Adherence_ITT] will be displayed by descriptive statistics (mean, standard deviation, interquartile-range (IQA), median, minimum and maximum for quantitative variables and absolute and relative frequencies for qualitative variables) and will be compared by exploratory p-values of t-tests and chi-square tests.

The results will be displayed for the ITT Population [ITT].

Tables: 6.10.2.6.2.1.2, 6.10.2.6.2.2.2, 6.10.2.6.2.3.2, 6.10.2.7.2.1.2, 6.10.2.7.2.2.2, 6.10.2.7.2.3.2, 6.10.2.5.2.1.2, 6.10.2.5.2.2.2, 6.10.2.5.2.3.2

A logistic regression will be performed for remission of depression [p2_1_hrsdRem] controlled for the HRSD-17 at visit P1 [p1_hrsdsc], HBA1c at visit S4 [S4_67], adherence group [Adherence_Randomised] and interaction of adherence group [Adherence_Randomised] and treatment group [S1_12].

The results will be displayed for the Randomized Population [Randomized] and ITT Population [ITT].

Tables: 6.10.2.9.1/2

6.11 Interim Analysis

No interim analysis will be conducted.

7 SOFTWARE

All analyses will be performed by the Statistical Analysis Software (SAS), Version 9.

8 APPENDIX 1 – LIST OF VARIABLES

Boldface variables (or their corresponding coded variables) in the following section will be displayed by summary tables. All continuous variables will be summarised using the following descriptive statistics: n, mean, standard deviation, median, interquartile-range (IQA), minimum and maximum. The frequency and percentage of observed levels will be presented for all categorical measures. Derived variables are displayed in italics.

8.1 Disposition of Patients

- **Informed Consent (questionnaires and diagnostics) and Inclusion/Exclusion Criteria**

- **Informed Consent given to questionnaires? (Yes / No) [SC_2]**
- Date of Informed Consent to questionnaires [dd/mm/yyyy] [SC_3]
- **Inclusion Criteria (questionnaires) (Yes / No for all criteria) [SC_4 – SC_9]**
- **Exclusion Criteria (questionnaires) (Yes / No for all criteria) [SC_10 – SC_13]**
- **Inclusion Criteria (psychologist) (Yes / No for all criteria) [P0_158]**
- **Exclusion Criteria (psychologist) (Yes / No for all criteria) [P0_159 – P0_165]**
- **Informed Consent given to diagnostics? (Yes / No) [SC_46]**
- Date of Informed Consent to diagnostics [dd/mm/yyyy] [SC_47]
- **Exclusion Criteria (diagnostics) (Yes / No for all criteria) [SC_48 – SC_53]**

- **Randomisation**

- Informed Consent for treatment in the coordinating institution (Yes / No) [S1_2]
- Date Informed Consent coordinating institution [dd/mm/yyyy] [S1_3]
- Informed Consent for treatment in the trial centre (investigator) (Yes / No) [S1_4]
- Date Informed Consent trial centre [dd/mm/yyyy] [S1_5]
- Participation in both diabetes trainings (Yes / No) [S1_6] (Note: At least one training or sufficient diabetes knowledge was considered sufficient)
- No violation of inclusion or exclusion criteria (Yes / No) [S1_7]
- Type of diabetes (Type I / Type II) [S1_8]
- Date of Randomisation [dd/mm/yyyy] [S1_9]
- Subject Randomized at the IZKS Mainz (Yes / No) [S1_11]
- Randomisation result (CBT / SER) [S1_12]

- **Analysis populations (Randomized, Safety, ITT, PP)**
- **Diagnostics part of the study completed**

- **Reasons for discontinuation of diagnostics part**

- **Psychological part of the study completed**

- **Reasons for discontinuation of psychological part**

- **Subject completed study according to protocol? (Yes / No) [EP_2]**

  If no, then

- **No interest [EP_3]**
- **Moving [EP_4]**
- **Working reasons [EP_5]**
- **No time [EP_6]**
- **Withdrawal of informed consent [EP_7]**
- **Other (patient) [EP_8]**
- **Violation of inclusion / exclusion criteria [EP_10]**
- **SAE of the patient [EP_12]**
- **Emergence of SUSARs during the study [EP_13]**
- **Sanitary reasons [EP_14]**
- **Absence in more than 3 CBT sessions [EP_15]**
- **Therapy non-responder [EP_16]**
- **Patient did not appear at agreed appointments [EP_17]**
- **Other (investigator) [EP_18]**

- **Participation on every study visit**
- **Participation on every short-term visit**
- **Participation on every long-term visit**
- **Type of protocol violations [ProtViolTyp]**

8.2 Demographics

- **Gender (male / female) [SC_14]**
- Year of birth yyyy [SC_15]
- ***Age [years] [Age]***
- Coordinating institution (Bad Mergenheim, Dortmund/Bochum, Düsseldorf/Köln, Mainz)
- **Marital Status (Married, living with partner / Married, split from partner / Widowed Living without partner) [SC_16]**
- **School Education (CSE (Hauptschulabschluss) / GCSE (Mittlere Reife) / High school diploma (Abitur or Fachhochschulreife) / University degree) [SC_17]**
- ***Education years (<10 / 10-14 / >14) [Education]***
- **Employment status [P0_6]**
- **Employment group [P0_7]**
- **Income *(<750€, 750€- <1500€, 1500€- <2000€, 2000€- <2500€, 2500€- <3000€, 3000€- <4000€, 4000€- <5000€, >5000€)* [P0_8]**
- **Continuous income [Income]**
- **Nationality [P0_9]**

- **Number of children [P0_5]**

8.3 Baseline Characteristics

- **ADS**

- **ADS questionnaire completed? (Yes / No) [SC_18]**
- **ADS Score [SC_19]**
- ***ADS Score categories (none 0-17; subclinical 18-22; >22 clinically relevant depression) [Screening_1_x_adssc]***
- **ADS Inclusion criterion (> 22) fulfilled? (Yes / No) [SC_20] (Note: This inclusion criterion could be overruled, if obvious symptoms of depression existed)**

- **Childhood Trauma Questionnaire (CTQ)**

- 28-items of the CTQ score [P0_557 – P0_589]
- ***Subscore: Emotional Neglect [P0_1_x_easc]***
- ***Subscore: Physical Abuse [P0_1_x_pansc]***
- ***Subscore: Physical Neglect [P0_1_x_phnsc]***
- ***Subscore: Sexual Abuse [P0_1_x_sasc]***
- ***Subscore: Emotional Abuse [P0_1_x_ea2sc]***
- ***Subscore: Extenuation/Denial [P0_1_x_edsc]***

- **Medical Data**

- **Type of diabetes mellitus (Type 1 / Type 2) [SC_54]**
- **Height [cm] [SC_55]**
- **Abdominal Girth [cm] [SC_56]**
- **Weight [kg] [SC_57]**
- **Hip Size [cm] [SC_58]**
- **Systolic Blood Pressure [mmHg] [SC_59]**
- **Diastolic Blood Pressure [mmHg] [SC_60]**
- **Pulse [/min] [SC_61]**
- **Treatment with hypertension medication? (Yes / No) [SC_62]**
- **Diabetes related diseases (Type of disease and Yes / No) [SC_63 – SC_77]**
- ***Late complications (Retinopathie, Nephropathie, Neuropathie) (Yes /No) [DiabComp][sc_63-sc_65]***
- ***Number of Late complications [NumberDiabComp]***
- ***Coronary Heart Disease (Yes /No) [KHK][sc_69-sc_71]***

- ***Macrovascular complications (KHK, Apoplex, PAVK) (Yes /No) [Comorb][KHK,sc_72, sc76]***
- ***Number of macrovascular complications [NumberComorb]***
- **Other relevant diseases? (Yes / No) [SC_78]**
- **Current concomitant medication? (Yes / No) [SC_79]**
- **Was there a change in concomitant medication?(Yes / No) at Visit S1 [S1_43]**

- **Current diabetes treatment**

- First manifestation of diabetes [mm/yyyy] [SC_81]
- ***Diabetes duration [years] [Dur_2]***
- First treatment of diabetes [mm/yyyy] [SC_82]
- **Current diabetes treatment (Diet / Oral Antidiabetics / Insulin) [SC_83, SC_84, SC_85]**
- Insulin Treatment since [mm/yyyy] [SC_86]
- ***Duration of insulin treatment [years] [Dur_3]***
- **Application form of Insulin (Pen or Injection [SC_87] / Pump [SC_88] / Inhalative [SC_89])**
- **Type of Insulin (Insulinanalogue [SC_90, SC_92] / Normal Insulin [SC_94, SC_96]/ Mixed Type [SC_98])**
- **Daily basal dose, daily prandial dose and daily total dose of Insulin [Units], [SC_91, SC_93, SC_95, SC_97, SC_99]**
- Antidiabetic medication since [mm/yyyy] [SC_100]
- ***Duration of oral antidiabetic medication [years] [Dur_4]***
- **Type of antidiabetic medication (Metformin[SC_101] / A-Glucosidase Inhibitor[SC_103] / Sulfonylharnstoffe or Glinide [SC_105]/ Glitazone [SC_107])**
- **Total daily dose [mg] [SC_101, SC_103, SC_105, SC_107] (Comment: Doses will be displayed for each medication type separately)**
- Single dose [mg] [SC_102, SC_104, SC_106, SC_108]
- **Was there a change in diabetes treatment? (Yes / No) at Visit S1 [S1_17]**

- **Lab**

- Laboratory measurements (Yes / No) by parameter (HbA1c [SC_109_1 - SC_109_2]/ GOT[SC_110_1 – SC_110_2] / GPT[SC_111_1 – SC_111_2])
- Endocrinologic parameters (Yes / No) at Visit S1 [S1_16]
- Date of lab value transfer [dd/mm/yyyy] [SC_117_1 – SC_117_2]
- **HbA1c [%] [SC_118_1 ,SC_118_2]**
- **ASAT/SGOT [U/l] [SC_119_1, SC_119_2]**
- **ALAT/SGPT [U/l] [SC_120_1, SC_120_2]**

- ASAT/SGOT exceeds 3 fold the upper reference limit? (Yes / No) [SC_120_1, SC_121_2]
- ALAT/SGPT exceeds 3 fold the upper reference limit? (Yes / No) [SC_122_1, SC_122_2]
- Is the patient in a fasting status? (Yes / No) at Visit S1 [S1_14]

- **SAE reminder**

-SAEs occurred? (Yes / No) [SC_112_1 – SC_112_2]

- **Study Participation**

-Patient qualified for further study participation? (Yes / No) [SC_113_1 – SC_113_2]

- **Concomitant psychological disorders according to SKID**
    - **Bipolar I** (lifetime/**current (yes/no)**) [P0_13, **P0_14**]
    - **Bipolar II** (lifetime/**current (yes/no)**) [P0_15, **P0_16**]
    - **Other Bipolar Disorder** (lifetime/**current (yes/no)**) [P0_17, **P0_18**]
    - **Current Major Depression (yes/no) [P0_19]**
    - Course
        - **Single episode [P0_20]**
        - **Recurrent episode [P0_21]**
        - **With seasonal pattern [P0_22]**
    - Subtype of the current episode
        - Either melancholic, atypical or catatonic characteristics [P0_23]
        - With postpartal beginning [P0_24]
        - Catatonic type [P0_25]
        - Melancholic subtype [P0_26]
        - With atypical characteristics [P0_27]
    - Severity of the current episode
        - Mild [P0_28]
        - Moderate [P0_29]
        - Severe with psychotic characteristics [P0_30]
        - Severe with mood-congruent psychotic characteristics [P0_31]
        - Severe with mood-incongruent psychotic characteristics [P0_32]
    - Current partly remission or complete remission
        - No remission [P0_33]
        - Partly remission [P0_34]
        - Complete remission [P0_35]
    - Chronology of the depressive episode

    - Age at first manifestation [years] [P0_36]
    - Number of previous episodes (including current episode) [P0_37]
    - Duration of current episode [weeks] [P0_38]
  - Previous major depression [P0_39]
    - Single episode [P0_40]
    - Recurrent episode [P0_41]
    - **Age at the beginning of the depressive episode [years] [P0_42]**
    - **Number of previous episodes (including current episode) [P0_43]**
  - **Dysthmia (yes/no) [P0_44]**
    - Without major depressive episode [P0_45]
    - With major depressive episode [P0_46]
    - Early start (at age of 21 or before) [P0_47]
    - Late start (at age of 22 or later) [P0_48]
  - **Depression not otherwise specified (NOS), current (yes/no) [P0_50]** lifetime [P0_49]
    - Postpsychotic depressive disorder [P0_51]
    - Major depressive episode, additional to a psychotic disorder [P0_52]
    - Premenstrual dysphoric disorder [P0_53]
    - Minor depression [P0_54]
    - Recurrent short depressive disorder [P0_55]
    - Other [P0_56]
  - Affective disorder due to disease factors [P0_57, P0_58]
    - With episodes like major depressive episodes [P0_59]
    - With depressive characteristics [P0_60]
    - With manic characteristics [P0_61]
    - With mixed characteristics [P0_62]
  - Substance-induced affective disorders [P0_63, P0_64]
    - With depressive characteristics [P0_65]
    - With manic characteristics [P0_66]
    - With mixed characteristics [P0_67]
  - Psychotic disorders
    - Schizophrenia [P0_68, P0_69]
    - Schizoid [P0_70, P0_71]
    - Schizoaffective [P0_72, P0_73]
    - Delusive [P0_74, P0_75]
    - Short psychotic disorder [P0_76, P0_77]
    - Psychotic disorder NOS [P0_78, P0_79]

- Psychotic disorder due to disease factors [P0_80, P0_81]
- Substance-induced psychotic disorder [P0_82, P0_83]

o Substance abuse and substance addiction
- Alcohol abuse [P0_84, P0_85]
- Alcohol addiction [P0_86, P0_87]
- Sedative abuse [P0_88, P0_89]
- Sedative addiction [P0_90, P0_91]
- Cannabis abuse [P0_92, P0_93]
- Cannabis addiction [P0_94, P0_95]
- Stimulative agents abuse [P0_96, P0_97]
- Stimulative agents addiction [P0_98, P0_99]
- Opioids abuse [P0_100, P0_101]
- Opioids addiction [P0_102, P0_103]
- Cocaine abuse [P0_104, P0_105]
- Cocaine addiction [P0_106, P0_107]
- Hallucinogen abuse [P0_108, P0_109]
- Hallucinogen addiction [P0_110, P0_111]
- Polytoxicomania abuse [P0_112, P0_113]
- Polytoxicomania addiction [P0_114, P0_115]
- Others abuse [P0_117, P0_118]
- Others addiction [P0_119, P0_120]

o Anxiety disorder
- Panic disorder without agoraphobia [P0_121, P0_122]
- Panic disorder with agoraphobia [P0_123, P0_124]
- Agoraphobia without panic disorder [P0_125, P0_126]
- Social phobia [P0_127, P0_128]
- Specific phobia [P0_129, P0_130]
- Obsessive-compulsive disorder [P0_131, P0_132]
- Posttraumatic stress disorder [P0_133, P0_134]
- Generalised anxiety disorder [P0_135, P0_136]
- Anxiety disorder NOS [P0_137, P0_138]
- Anxiety disorder due to disease factors [P0_139, P0_140]
- Substance-induced anxiety disorder [P0_141, P0_142]

o Somatoform disorders
- Somatization disorder [P0_143]
- Pain disorder [P0_144]
- Unspecified somatoform disorder [P0_145]

        - Hypochondria [P0_146]
        - Body-dysmorphic disorder [P0_147]
    - Eating disorder
        - Anorexia nervosa [P0_148, P0_149]
        - Bulimia nervosa [P0_150, P0_151]
        - Disorder with eating attacks [P0_152, P0_153]
    - Other DSM-IV disorders
        - Adjustment disorder [P0_154]
        - Other DSM-IV disorders [P0_155, P0_156]

- **Concomitant diseases**

- **Diagnosis [mh_1_1 – mh_1_<n>]**
- ICD-10 Code [mh_2_1 – mh_2_<n>]
- Start concomitant disease [dd/mm/yyyy] [mh_3_1 – mh_3_<n>]
- End concomitant disease [dd/mm/yyyy] [mh_4_1 – mh_4_<n>]
- Ongoing (Yes / No) [mh_5_1 – mh_5_<n>]
- ***Duration of concomitant disease including diabetes related diseases (for ongoing diseases) [years] [Dur_5_1 – Dur_5_<n>]***
- ***Avg. Duration of Concom. Diseases (yrs) [ConDisDur]***

- **Concomitant medication**

- **Medication name (coded according to Rote Liste) [CMS_1_1 – CMS_1_<n>] (Note: Those CRF entries with a stop date before study start will be removed from the table)**
- Daily dose [CMS_2_1 – CMS_2_<n>]
- Unit [CMS_3_1 – CMS_3_<n>]
- Start concomitant medication [dd/mm/yyyy] [CMS_4_1 – CMS_4_<n>]
- End concomitant medication [dd/mm/yyyy] [CMS_5_1 – CMS_5_<n>]
- Ongoing (Yes / No) [CMS_6_1 – CMS_6_<n>]
- ***Duration of concomitant medications (for ongoing medications) [Dur_6_1 – Dur_6_<n>]***
- ***Avg. Duration of Concom. Medications (yrs)[ConMedDur]***

- **Sertraline specific items**

- **Intensity of depression according to CGI (0 to 9) [S1_44]**
- Sertraline dispensed (Yes / No) [S1_45]
- Number of sertraline tablets dispensed [s1_46]
- **Daily dose [mg] [S1_47]**

- Comment, if daily dose deviates from the recommended dose [S1_48]
- Pregnancy (Yes / No / Not applicable) [S1_49]
- **Plasma concentrations of sertraline and desmethylsertraline [SER_9_1, SER_11_1]**
- ***Ratio of desmethylsertraline/sertraline [SER_15_1 – SER_15_<n>]***

- **Extend of exposure**
- ***Number of patients participating each visit (i.e. for the CBT group patients participating the visits S1, S4, L1, L2, L3 and L4; for the SER group patients participating the visits S1, S2, S3, S4, L1, L2, L3 and L4) and summaries over short-term phase and overall.***
- ***Number of days in the study after randomisation [Dur_4]***
- **Drop-out (Starter, KZG, LZG)**

- **Adherence to therapy**
- ***CBT-adherence [Adherence_CBT]***
- ***SER-adherence [Adherence1_SER_ITT, Adherence1_SER_RAN, Adherence2_SER_ITT, Adherence2_SER_RAN]***
- **Number of CBT sessions**

8.4 Primary Variable

- ***Difference of the HbA1c value at visit L4 and baseline [l4_69-S1_77]***

HbA1c values per visit [SC_118_1/2, S1_77, S4_67, l1_69, l2_67, l3_67, l4_69]

HbA1c values per visit LOCF [HbA1c_S1_L, HbA1c_S4_L, HbA1c_L1_L, HbA1c_L2_L, HbA1c_L3_L, HbA1c_L4_L]

8.5 Secondary Variables

8.5.1 Efficacy

- **Hamilton Depression Scale (HRSD-17)**
- **17 items of the HRSD-17 score [P0_179_1 – P0_196_1 (P0_179_2 – P0_196_2, P0_179_3 – P0_196_3), P1_16_1 – P1_33_1, P2_69_1 – P2_86_1]**
- ***Total HRSD-17 score [P0_hrsdsc, P1_hrsdsc, P2_hrsdsc]***
- ***Remission of depression [P2_1_hrsdRem]***
- ***Improvement of depression [P1_1_hrsdImp, P2_1_hrsdImp]***

- ***Treatment responders [P1_1_hrsdtre, P2_1_hrsdtre]***

- **Short Form 36 (SF-36, German version, Bullinger et al., 1998)**

- 36-items of the SF 36 score [P0_484 P0_485 P0_487 – P0_500 P0_502 – P0_506 P0_508 – P0_517 P0_519 – P0_523, P1_321 P1_322 P1_324 – P1_337 P1_339 – P1_343 P1_345 – P1_354 P1_356 – P1_360, P2_374 P2_375 P2_377 – P2_390 P2_392 – P2_396 P2_398 – P2_407 P2_409 – P2_413]
- ***8 scales covering physical functioning [P0_1_SF_PF, P1_1_SF_PF, P2_1_SF_PF], role-physical [P0_1_SF_RP, P1_1_SF_RP, P2_1_SF_RP], bodily pain [P0_1_SF_BP, P1_1_SF_BP, P2_1_SF_BP], general health [P0_1_SF_GH, P1_1_SF_GH, P2_1_SF_GH], vitality [P0_1_SF_V, P1_1_SF_V, P2_1_SF_V], social functioning [P0_1_SF_SF, P1_1_SF_SF, P2_1_SF_SF], role-emotional [P0_1_SF_RE, P1_1_SF_RE, P2_1_SF_RE], and mental health [P0_1_SF_MH, P1_1_SF_MH, P2_1_SF_MH].***
- ***Physical health component score (PCS) [P0_SF_sPH, P1_SF_sPH, P2_SF_sPH]***
- ***Mental health component score (MCS) [P0_SF_sMH, P1_SF_sMH, P2_SF_sMH]***

- **Problem Areas in Diabetes Survey (PAID)**

- 20 items of the PAID [P0_374 – P0_383 P0_385 – P0_394, P1_211 – P1_220 P1_222 – P1_231, P2_264 – P2_273 P2_275 – P2_284]
- ***Total score of the PAID [P0_1_x_paisc, P1_1_x_paisc, P2_1_x_paisc]***

- **Allgemeine Depressions Skala (ADS, German translation of the CES-D)**

- 20-items of the ADS score [SC_25 – SC_44, S1_56 – S1_75, S4_70 – S4_89, L1_76 – L1_95, L2_70 – L2_89, L3_70 – L3_89, L4_72 – L4_91]
- ***Total ADS score (categorical) [Screening_1_x_adssc, S1_1_x_adssc, S4_1_x_adssc, L1_1_x_adssc, L2_1_x_adssc, L3_1_x_adssc, L4_1_x_adssc]***
- ***Total ADS score (continuous)***

- **SCL-K-9**

- 9-items of the SCL-K-9 score [P0_536 – P0_544, P1_373 – P1_381, P2_426 – P2_434]
- ***Total SCL-K-9 score [P0_1_x_sclk9sc, P1_1_x_sclk9sc, P2_1_x_sclk9sc]***

- **Diabetes Wissens Test: Typ 1 (DWT: Type 1)**

- 30-items of the DWT type 1 score [P0_201 – P0_294, P1_38 – P1_131, P2_91 – P2_184]
- ***Total DWT type 1 score [P0_1_x_dt1sc, P1_1_x_dt1sc, P2_1_x_dt1sc]***

- **Diabetes Wissens Test: Typ 2 (DWT: Type 2)**

- 17-items of the DWT type 2 score [P0_297 – P0_355, P1_134 – P1_192, P2_187 – P2_245]

- ***Total DWT type 2 score [P0_1_x_dt2sc, P1_1_x_dt2sc, P2_1_x_dt2sc]***
- **Frequency of participation in a diabetes training [P0_356, P1_193, P2_246]**
- **Urine glucose measurement [P0_357, P0_358, P1_194, P1_195, P2_247, P2_248]**
- **Plasma glucose measurement [P0_359, P0_360, P1_196, P1_197, P2_249, P2_250]**

- **IPC Diabetes-Fragebogen (IPC-D1)**

- 29-items of the IPC score [P0_396 – P0_404 P0_406 – P0_415 P0_417 – P0_426, P1_233 – P1_241 P1_243 – P1_252 P1_254 – P1_263, P2_286 – P2_294 P2_296 – P2_305 P2_307 – P2_316]
- ***Subscore: Internality [P0_1_x_intSC, P1_1_x_intSC, P2_1_x_intSC]***
- ***Subscore: Physician related externality [P0_1_x_PhySC, P1_1_x_PhySC, P2_1_x_PhySC]***
- ***Subscore: Unpredictability [P0_1_x_UnpSC, P1_1_x_UnpSC, P2_1_x_UnpSC]***
- ***Subscore: Fortune and Chance [P0_1_x_FoChSC, P1_1_x_FoChSC, P2_1_x_FoChSC]***

- **Freiburger Fragebogen zur Krankheitsverarbeitung (FKV-15)**

- 15 items of the FKV-15 score [P0_468 – P0_482, P1_305 – P1_319, P2_358 – P2_372]
- ***Subscore: Active problem-focused coping [P0_1_x_acsc, P1_1_x_acsc, P2_1_x_acsc]***
- ***Subscore: Depressive coping [P0_1_x_dhsc, P1_1_x_dhsc, P2_1_x_dhsc]***
- ***Subscore: Distraction [P0_1_x_disc, P1_1_x_disc, P2_1_x_disc]***
- ***Subscore: Trivializing [P0_1_x_exsc, P1_1_x_exsc, P2_1_x_exsc]***
- ***Subscore: Doctor-centered coping [P0_1_x_cosc, P1_1_x_cosc, P2_1_x_cosc]***

- **Rosenberg-Scale (SE)**

- 10 items of the SE score [P0_525 – P0_534, P1_362 – P1_371, P2_415 – P2_424]
- ***Total SE score [P0_1_x_sesc, P1_1_x_sesc, P2_1_x_sesc]***

- **Barriers to Insulin Therapy Questionnaire (BIT)**

- 14 items of the BIT score [P0_428 – P0_434 P0_436 – P0_442, P1_265 – P1_271 P1_273 – P1_279, P2_318 – P2_324 P2_326 – P2_332]
- ***Subscore: Fear of injections and self-testing [P0_1_x_fitsc, P1_1_x_fitsc, P2_1_x_fitsc]***
- ***Subscore: Expectations regarding positive insulin related outcomes [P0_1_x_rosc, P1_1_x_rosc, P2_1_x_rosc]***
- ***Subscore: Expected hardship from insulin therapy [P0_1_x_ehsc, P1_1_x_ehsc, P2_1_x_ehsc]***

- ***Subscore: Stigmatization by insulin injections [P0_1_x_stsc, P1_1_x_stsc, P2_1_x_stsc]***
- ***Subscore: Fear of hypoglycaemia [P0_1_x_hysc, P1_1_x_hysc, P2_1_x_hysc]***
- ***Total BIT score [P0_1_x_bitsc, P1_1_x_bitsc, P2_1_x_bitsc]***

- **Summary of Diabetes Self Care Activities Measure (SDSCA)**

- 10 items of the SDSCA score [P0_362 – P0_372, P1_199 – P1_209, P2_252 – P2_262]
- ***Subscore: General Diet [P0_1_x_gdsc, P1_1_x_gdsc, P2_1_x_gdsc]***
- ***Subscore: Specific Diet [P0_1_x_sdsc, P1_1_x_sdsc, P2_1_x_sdsc]***
- ***Subscore: Exercise [P0_366, P1_203, P2_256]***
- ***Subscore: Blood-Glucose Testing [P0_1_x_bgsc, P1_1_x_bgsc, P2_1_x_bgsc]***
- ***Subscore: Foot-Care [P0_1_x_fcsc, P1_1_x_fcsc, P2_1_x_fcsc]***
- **Smoking Status [P0_371, P1_208, P2_261]**
- **Smoked number of cigarettes [P0_372, P1_209, P2_262]**

- **Fragebogen zur Perzipierten Familialen Unterstützung und Kommunikation – Revision (PFUK-R)**

- Items of the PFUK-R [P0_592 – P0_609 P0_611 – P0_624 P0_626 – P0_641 P0_643 – P0_646, P1_395 – P1_412 P1_414 – P1_427 P1_429 – P1_444 P1_446 – P1_449, P2_448 – P2_465 P2_467 P2_480 P2_482 – P2_497 P2_499 – P2_502]
- ***Subscore: Global appropriateness [P0_1_glob_a, P1_1_glob_a, P2_1_glob_a]***
- ***Subscore: Global availability [P0_1_glob_v, P1_1_glob_v, P2_1_glob_v]***
- ***Subscore: Emotional support appropriateness [P0_1_emos_a, P1_1_emos_a, P2_1_emos_a])***
- ***Subscore: Emotional support availability [P0_1_emos_v, P1_1_emos_v, P2_1_emos_v])***
- ***Subscore: Overprotective support appropriateness [P0_1_entl_a, P1_1_entl_a, P2_1_entl_a])***
- ***Subscore: Overprotective support availability [P0_1_entl_v, P1_1_entl_v, P2_1_entl_v])***

- **Hopelessness Scale (H-Skala)**

- 10-items of the H-Scale score [P0_546 – P0_555, P1_383 – P1_392, P2_436 – P2_445]
- ***Total H-Scale score [P0_1_x_hrasc, P1_1_x_hrasc, P2_1_x_hrasc]***

- **Inkongruenzfragebogen (K-INK)**

- 23 items of the K-INK score [P0_444 – P0_466, P1_281 – P1_303, P2_334 – P2_356]
- ***Subscore: Approach Aims [P0_1_x_apasc, P1_1_x_apasc, P2_1_x_apasc]***
- ***Subscore: Avoidance Aims [P0_1_x_avasc, P1_1_x_avasc, P2_1_x_avasc]***

- ***Total K-INK score [P0_x_tssc, P1_x_tssc, P2_x_tssc]***

8.5.2 Safety

- **Adverse Events (AE) [AE_PS_1_1- AE_PS_10_<n> (for Psychologist) and AE_P_1_1 – AE_P_1_<n> (for Physician)]**

- AE number
- **AE Term (coded according to Medical Dictionary for Medical Activities (MedDRA)) [AE_PS_1_1 – AE_PS_1_<n>, AE_P_1_1 – AE_P_1_<n>]**
- **Serious Adverse Event (SAE) [AE_PS_2_1 – AE_PS_2_<n>, AE_P_2_1 – AE_P_2_<n>] (Yes / No)**
- **AE Intensity [AE_PS_3_1 – AE_PS_3_<n>, AE_P_3_1 – AE_P_3_<n>] (mild / moderate / severe)**
- AE Start Date [AE_PS_4_1 – AE_PS_4_<n>, AE_P_4_1 – AE_P_4_<n>] [dd/mm/yyyy]
- AE End Date [AE_PS_5_1 – AE_PS_5_<n>, AE_P_5_1 – AE_P_5_<n>] [dd/mm/yyyy]
- AE ongoing [AE_PS_6_1 – AE_PS_6_<n>, AE_P_6_1 – AE_P_6_<n>] (Yes / No)
- Action taken, study medication [AE_PS_7_1 – AE_PS_7_<n>, AE_P_7_1 – AE_P_7_<n>] (none / discontinued / increased / reduced / unknown / not applicable)
- **AE relationship [AE_PS_8_1 – AE_PS_8_<n>, AE_P_8_1 – AE_P_8_<n>] (none / definitive / likely / possible / improbable / clarification not possible)**
- Action taken, other action [AE_PS_9_1 – AE_PS_9_<n>, AE_P_9_1 – AE_P_9_<n>] (none / no treatment necessary / hospitalisation / study discontinued / other)
- AE outcome [AE_PS_10_1 – AE_PS_10_<n>, AE_P_10_1 – AE_P_10_<n>] (recovered / transient defect / recovered with sequelae / not recovered / unknown / death)

9 APPENDIX 2 – RECODED VALUES OF THE SF-36

Item numbers	Original value	Coded value
1 [P0_484, P1_321, P2_374]	1	4
	2	3.4
	3	2.4
	4	1
	5	0
2 (no recoding necessary, item 2 is not part of any composite score) [P0_485 , P1_322, P2_375]	1	1
	2	2
	3	3
	4	4
	5	5
3a [P0_487, P1_324, P2_377], 3b [P0_488, P1_325, P2_378], 3c [P0_489, P1_326, P2_379], 3d [P0_490, P1_327, P2_380], 3e [P0_491, P1_328, P2_381], 3f [P0_492, P1_329, P2_382], 3g [P0_493, P1_330, P2_383], 3h [P0_494, P1_331, P2_384], 3i [P0_495, P1_332, P2_385], 3j [P0_496, P1_333, P2_386]	1	0
	2	1
	3	2
4a [P0_497, P1_334, P2_387], 4b [P0_498, P1_335, P2_388], 4c [P0_499, P1_336, P2_389], 4d [P0_500, P1_337, P2_390]	1	0
	2	1
5a [P0_502, P1_339, P2_392], 5b [P0_503, P1_340, P2_393], 5c [P0_504, P1_341, P2_394]	1	0
	2	1
6 [P0_505, P1_342, P2_395]	5	0
	4	1
	3	2
	2	3
	1	4
7 [P0_506, P1_343, P2_396]	1	5
	2	4.1
	3	3.2
	4	2.1
	5	1.1

	6	0
8 [P0_508, P1_345, P2_398]	1	4
	2	3
	3	2
	4	1
	5	0
	IF item 7=5 and item 8=4	5
	IF .<item 7<5 and item 8=4	4
	IF item 7=.	item 8 multiplied by 1.25
9a [P0_509, P1_346, P2_399], 9d [P0_512, P1_349, P2_402], 9e [P0_513, P1_350, P2_403], 9h [P0_516, P1_353, P2_406]	1	5
	2	4
	3	3
	4	2
	5	1
	6	0
9b [P0_510, P1_347, P2_400], 9c [P0_511, P1_348, P2_401], 9f [P0_514, P1_351, P2_404], 9g [P0_515, P1_352, P2_405], 9i [P0_517, P1_354, P2_407]	1	0
	2	1
	3	2
	4	3
	5	4
	6	5
10 [P0_519, P1_356, P2_409], 11a [P0_520, P1_357, P2_410], 11c [P0_522, P1_359, P2_412]	1	0
	2	1
	3	2
	4	3
	5	4
11b [P0_521, P1_358, P2_411], 11d [P0_523, P1_360, P2_413]	1	4
	2	3
	3	2
	4	1
	5	0

10 APPENDIX 3 – POPULATION MEANS AND STANDARD DEVIATIONS FOR CALCULATION OF SF-36 Z-SCORES

Physical Functioning

Sex	Age	Pop. mean	Pop. SD
male	0 to 20	95.81	18.69
	21 to 30	96.44	9.97
	31 to 40	96.44	9.97
	41 to 50	90.50	18.66
	51 to 60	84.09	19.41
	61 to 70	77.32	22.92
	71 and more	69.28	26.36
female	0 to 20	94.78	20.06
	21 to 30	93.95	12.89
	31 to 40	91.03	16.86
	41 to 50	87.27	16.59
	51 to 60	83.34	19.68
	61 to 70	74.79	22.29
	71 and more	53.73	26.68

Role Physical

Sex	Age	Pop. mean	Pop. SD
male	0 to 20	98.29	10.91
	21 to 30	95.82	17.56
	31 to 40	91.43	26.26
	41 to 50	88.02	30.04
	51 to 60	83.33	29.89
	61 to 70	72.34	33.96
	71 and more	72.76	35.61
female	0 to 20	92.99	25.12

	21 to 30	87.75	28.57
	31 to 40	88.99	22.45
	41 to 50	86.91	25.62
	51 to 60	77.81	33.59
	61 to 70	72.71	36.38
	71 and more	57.24	42.69

Role Emotional

Sex	Age	Pop. mean	Pop. SD
male	0 to 20	94.91	28.82
	21 to 30	94.46	19.51
	31 to 40	93.57	23.35
	41 to 50	92.41	23.87
	51 to 60	89.74	26.27
	61 to 70	89.99	24.67
	71 and more	85.92	29.02
female	0 to 20	94.32	20.15
	21 to 30	91.29	22.48
	31 to 40	89.26	23.74
	41 to 50	90.56	21.82
	51 to 60	88.05	27.57
	61 to 70	87.98	26.70
	71 and more	81.69	36.08

Vitality

Sex	Age	Pop. mean	Pop. SD
male	0 to 20	72.90	19.57
	21 to 30	67.52	18.80
	31 to 40	68.53	15.00
	41 to 50	65.79	17.36

	51 to 60	64.16	16.64
	61 to 70	62.31	19.14
	71 and more	60.01	19.97
female	0 to 20	72.00	22.42
	21 to 30	62.21	17.95
	31 to 40	63.06	15.79
	41 to 50	62.34	15.56
	51 to 60	58.29	17.59
	61 to 70	60.08	17.80
	71 and more	51.17	21.62

Mental Health

Sex	Age	Pop. mean	Pop. SD
male	0 to 20	78.30	18.25
	21 to 30	76.49	16.58
	31 to 40	77.06	16.44
	41 to 50	76.01	16.65
	51 to 60	74.69	14.41
	61 to 70	78.22	15.80
	71 and more	76.10	16.05
female	0 to 20	73.31	18.84
	21 to 30	71.75	18.84
	31 to 40	71.81	14.03
	41 to 50	71.11	14.23
	51 to 60	70.22	17.39
	61 to 70	73.60	17.14
	71 and more	69.30	17.43

Social Functioning

Sex	Age	Pop. mean	Pop. SD

male	0 to 20	94.18	20.32
	21 to 30	93.12	17.04
	31 to 40	92.10	16.38
	41 to 50	91.09	18.18
	51 to 60	88.35	15.93
	61 to 70	88.75	16.80
	71 and more	83.57	20.15
female	0 to 20	92.20	17.78
	21 to 30	89.14	18.34
	31 to 40	87.24	17.37
	41 to 50	87.51	16.47
	51 to 60	85.30	19.84
	61 to 70	85.67	19.68
	71 and more	84.12	21.92

Bodily Pain

Sex	Age	Pop. mean	Pop. SD
male	0 to 20	91.76	30.70
	21 to 30	88.91	21.62
	31 to 40	89.64	20.42
	41 to 50	80.78	30.38
	51 to 60	75.74	26.37
	61 to 70	72.15	26.05
	71 and more	71.61	26.24
female	0 to 20	87.04	31.43
	21 to 30	83.97	26.57
	31 to 40	84.33	21.23
	41 to 50	76.89	25.90
	51 to 60	69.77	28.00

	61 to 70	70.41	27.82
	71 and more	60.78	28.54

General Health

Sex	Age	Pop. mean	Pop. SD
male	0 to 20	80.28	20.62
	21 to 30	77.12	18.34
	31 to 40	76.54	16.13
	41 to 50	67.86	21.35
	51 to 60	60.98	18.55
	61 to 70	58.92	18.13
	71 and more	58.61	21.26
female	0 to 20	82.04	17.43
	21 to 30	74.49	17.65
	31 to 40	72.12	15.39
	41 to 50	68.14	16.58
	51 to 60	61.08	19.70
	61 to 70	58.49	18.17
	71 and more	53.76	20.65

11 APPENDIX 4 – CALCULATION OF SER-ADHERENCE

Definition and rating of adherence

Adherence to treatment was measured for both treatment groups separately.

1. Adherence to CBT

Adherence to CBT was assessed by the number of attended CBT-sessions. As there exists no established measure of adherence for psychotherapy, in the current study adherence to CBT was defined as participation in at least eight out of ten sessions. Adherence was categorised as adherent, partially non-adherent and non-adherent (see table 1 for the definition of adherence).

Derived variables:

CBT-adherence [Adherence_CBT]: 1=adherent, patient attended to at least 8 CBT sessions, 2= partially non-adherent, patient attended to 1-7 CBT sessions, 3= non-adherent, patients participated in 0 CBT sessions

2. Adherence to Sertraline

Adherence to sertraline treatment was assessed by repeated measures of serum sertraline concentrations and ratio of metabolite:parent compound (N-Desmethylsertraline, DSER: Sertraline, SER) as recommended by Reis and colleagues. As patients identified as treatment non-responders after the end of the short-term phase were excluded from the treatment protocol, sertraline adherence was evaluated separately for randomized patients and ITT patients.

For randomized patients adherence was assessed based on SER concentrations and DSER:SER measured at week 8 and 12, for ITT patients adherence was assessed based on SER concentrations and DSER:SER measured at week 8, 12, 24, 36, 48 and 60.

Notwithstanding initial planning and TDM guidelines [43], which recommend concentrations between 10-50 ng/ml, target ranges of SER were defined as concentrations between 10-100 ng/ml, as up to 100 ng/ml no severe adverse effects are to be expected. Furthermore, it has to be taken into account that the study participants suffer from chronic disease and receive concomitant medications, which affect drug metabolism resulting in higher serum concentration [44]. Target ranges of DSER:SER were defined as 0.85 – 4.25, corresponding to the mean ± 2 standard deviations (M=2.55, SD=0.85) derived from 348 samples by Reis et al. (2004).

Adherence to sertraline was categorised as adherent, partially non-adherent and non-adherent (see table 1). For the sertraline group, we also aimed at identifying overt and hidden forms of complete non-adherence. Non-adherence was considered overt, if patients chose to discontinue or refuse sertraline treatment or withdrew informed consent during the short-term phase of the study for randomized patients and during the whole study phase for ITT patients, respectively.All other cases of complete non-adherence were considered hidden.

To avoid biased adherence ratings, patients discontinuing the study participation due to severe adverse events or discontinuing sertraline intake because of their physician’s recommendation, were excluded from the adherence rating.

In cases of missing blood samples or concentrations not detectable due to interferences, all available blood samples of the patient were discussed with a psychiatrist with expertise in therapeutic drug monitoring. Based on the course of available SER concentrations, DSER concentrations and ratios DSER:SER adherence was estimated for these patients. If only one blood sample was available, the patient was excluded from the adherence rating. As high SER concentrations (> 100 ng/ml) are often observed in case of concomitant medication, SER concentrations above the target range were considered adherent, if the corresponding ratio DSER:SER fell into the target range.

Derived variables:

Adherence1_SER_Ran: 1 = adherent, 2 = partially non-adherent, 3 = non-adherent

Adherence1_SER_ITT: 1 = adherent, 2 = partially non-adherent, 3 = Non-adherent

Adherence2_SER_Ran: 1 = adherent, 2 = partially non-adherent, 3 = hidden non-adherent, 4 = open Non-adherent.

Adherence2_SER_ITT: 1 = adherent, 2 = partially non-adherent, 3 = hidden non-adherent, 4 = open Non-adherent.

3. Overall adherence

In addition, a binary overall adherence rating was employed distinguishing adherent from completely non-adherent/partially non-adherent patients for all randomised patients and all ITT patients, respectively. Overall adherence to treatment for randomised patients was defined as attendance to at least eight sessions of CBT or both sertraline concentrations and corresponding ratios DSER:SER in the target range during the short-term phase. Overall adherence for ITT patients was defined as attendance to at least eight sessions of CBT or at least at five sertraline concentrations and corresponding DSER:SER ratios in the target range. Patients considered partially or completely non-adherent with sertraline treatment or participating in one to seven CBT sessions were summarised as non-adherent.

Derived variables:

1) Adherence full ITT sample: 0= nonadherent/partially non-adherent, 1= adherent.
2) Adherence full Random sample: 0= nonadherent/partially non-adherent, 1= adherent.

Table 1 – Definition of adherence in CBT and sertraline patients

**Adherence categories**	**CBT**	**Sertraline (randomised sample)**	**Sertraline (ITT sample)**
Adherent	Participation in ≥ 8 sessions of CBT	2 SER and corresponding DSER: SER in target range	≥ 5 SER and corresponding DSER: SER in target range
Partially non-adherent	Participation in 1-7 sessions of CBT	1 SER and corresponding DSER: SER in target range	1-4 SER and corresponding DSER: SER in target range
Non-adherent	No participation in	no SER and	no SER and

	any session of CBT	corresponding DSER: SER in target range	corresponding DSER: SER in target range

Note: SER: blood concentration of sertraline. DSER: SER: ratio of desmethylser-traline to sertraline. Target ranges of SER were defined as concentrations between 10-100 ng/ml. Target ranges of DSER:SER were defined as 0.85 – 4.25, corresponding to the mean ± 2 standard deviations (M=2.55, SD=0.85) derived from 348 samples by Reis et al.
