# Supplementary material for: Study protocol of the Diabetes and Depression Study (DAD): a multi-center randomized controlled trial to compare the efficacy of a diabetes-specific cognitive behavioral group therapy versus sertraline in patients with major depression and poorly controlled diabetes mellitus
Source: BMC Psychiatry. 2013 Aug 6;13:206. doi: 10.1186/1471-244X-13-206 (PMC3750698; doi:10.1186/1471-244X-13-206)
Supplement: Additional file 7 — Data management plan. [file 1471-244X-13-206-S7.pdf]

Data Management Plan

**Short-Title:**	**Diabetes and Depression Study (DAD Study)**
**EudraCT:**	**2005-004525-26**
**Clinical Trial Code:**	**2005-009_DAD-Petrak_BMBF**
**Sponsor:**	**Ruhr-Universität Bochum, represented by: Dr. rer. soc. Dipl.-Psych. Frank Petrak**
**Date of Creation:**	**07.08.2008**
**Version:**	**1.0**

The present Data Management Plan was subject to critical review and has been approved in the present version by the undersigned. The information contained is consistent with the Clinical Trial Protocol.

Name:	Dr. rer. Soc. Dipl.-Psych. Frank Petrak		Function: Sponsor/Representative
Date:	|__|__|.|__|__|.|__|__|__|__|	Signature:	________________________________
Name:	Dip. Inform. (FH) I. Stamm	Function:	Head of Clinical Data Management
Date:	|__|__|.|__|__|.|__|__|__|__|	Signature:	________________________________
Name:	Dipl.-Dok. (FH) C. Reck	Function:	Trial Data Manager
Date:	|__|__|.|__|__|.|__|__|__|__|	Signature:	________________________________

1 Introduction 3
1.1 Version History 3
1.2 Background 3
1.3 Trial Objectives 3
1.4 Purpose and Scope 3

2 Data Management Overview 4
2.1 Systems Used 4
2.2 Project Timing 4
2.3 Risk Management 4
2.4 Data Flow 5
2.5 Data Collection 5
2.5.1 Creation of CRF and CRF Data Processing 5
2.6 Creation of the Data Entry Application and the Database 5
2.7 Data Entry 6
2.7.1 CRF Data Entry 6
2.7.2 Medical Coding 6
2.8 Data Validation 6
2.9 Query Process and Data Correction 6
2.10 Locking of the Trial Database 7

3 Database and Patient Data Validation Strategy 8
3.1 Data Validation Plan 8
3.2 Query Management System (QMS) 8

4 Validation Processes prior and during Data Entry 9
4.1 Creation of Database and Data Entry Screens 9
4.2 Data Validation 9
4.2.1 Visual Control of Completed CRF Pages 9
4.2.2 Data Correction Processes 9
4.2.3 Handling of Self-Evident Corrections (during data entry) 9
4.2.4 Data Correction by Manual Query 9

5 Validation Processessess after Data Entry 10
5.1 SAS Database Validation 10
5.2 Data Validation 10
5.2.1 SAS-Compare of Double Entered Data 10
5.2.2 Visual Check of Single Entered Data 10
5.2.3 Query Process and Data Correction 10
5.2.4 Missing Information 11
5.2.5 Handling of Self-evident Corrections (after data entry) 11
5.3 Medical Coding 11
5.3.1 Adverse Events 11
5.3.2 Concomitant Medication 11
5.3.3 Concomitant Diseases 11
5.4 SAE Reconciliation 12

6 Processes after Data Validation 13
6.1 Database Audit 13
6.2 Database Closure 13

1 INTRODUCTION

The Data Management Plan (DMP) specifies each data management process for the DAD trial. This paper additionally documents trial specific data necessary for the data management e.g. time schedules.

1.1 Version History

Version	Reason for Change	Date

1.2 Background

The purpose of the DMP is to describe the underlying data management process which governs collection, management, review and reporting of data from a clinical trial. The content of a DMP includes, but is not limited to, trial timelines, data management procedures including data collection and data flow. The DMP documents the validation strategy and tools, and references responsibilities for different tasks.

1.3 Trial Objectives

Multicentre randomised controlled trial (RCT) comparing sertaline vs. CBT. After 12 weeks of open-label therapy, only the treatment-responders (50% improvement of depression) of both groups will be included in the one-year long-term phase of the study. In the long-term phase, diabetological treatment as usual will be given to both groups. CBT-responders will receive no further treatment, while SSRI-responders will be given a sustained sertraline regimen as relapse prevention. The primary outcome variable is a 1% improvement of the $HbA1_c$ at the one-year follow-up; the most important secondary outcome variable is remission of depression.

1.4 Purpose and Scope

The DAD trial is conducted in one country. In accordance with the approved protocol 304 patients are expected to be included into the trial.

2 DATA MANAGEMENT OVERVIEW

The processes of the data management are defined by the nature of the trial. DAD will be conducted as a paper based trial. The major part of data will be recorded on paper CRFs (Case Record/ Report Form) and initially processed using the Clinical Data Management (CDM) database system MACRO (InferMed, London). Clinical data will furthermore be processed by means of the software SAS. The following major parts of the data management processes for DAD will be realised under the conduct of IZKS Mainz.

2.1 Systems Used

The major part of data will be recorded and initially processed using the CDM database system MACRO. MACRO has been designed to support the requirements of internationally recognised ICH Good Clinical Practice and FDA 21 CFR Part 11. It is acknowledged as a highly user-friendly electronic data collection solution for clinical research and is used by pharmaceutical companies and academic research groups worldwide. Scope, distribution and hardware requirements for the system are determined by the CDM group. Additional SAS data files will be generated for further data analysis and processing.

2.2 Project Timing

The duration of the trial is 36 month

Beginning of trial: 01.03.2006

End of trial: 31.01.2009

2.3 Risk Management

In order to discover possible risks at an early stage and to minimise a potential drop in the data quality risks should be considered on system as well as on process level. The study data management system used by IZKS Mainz is used in a validated environment. System side risks can therefore be neglected as long as the validity of the system is guaranteed by the system responsible (which is expected here). To minimise risks on process level a detailed risk analysis can be omitted while the processes are operated as in this data management plan stated.

2.4 Data Flow

An overview of the data flow is given in the following diagram.

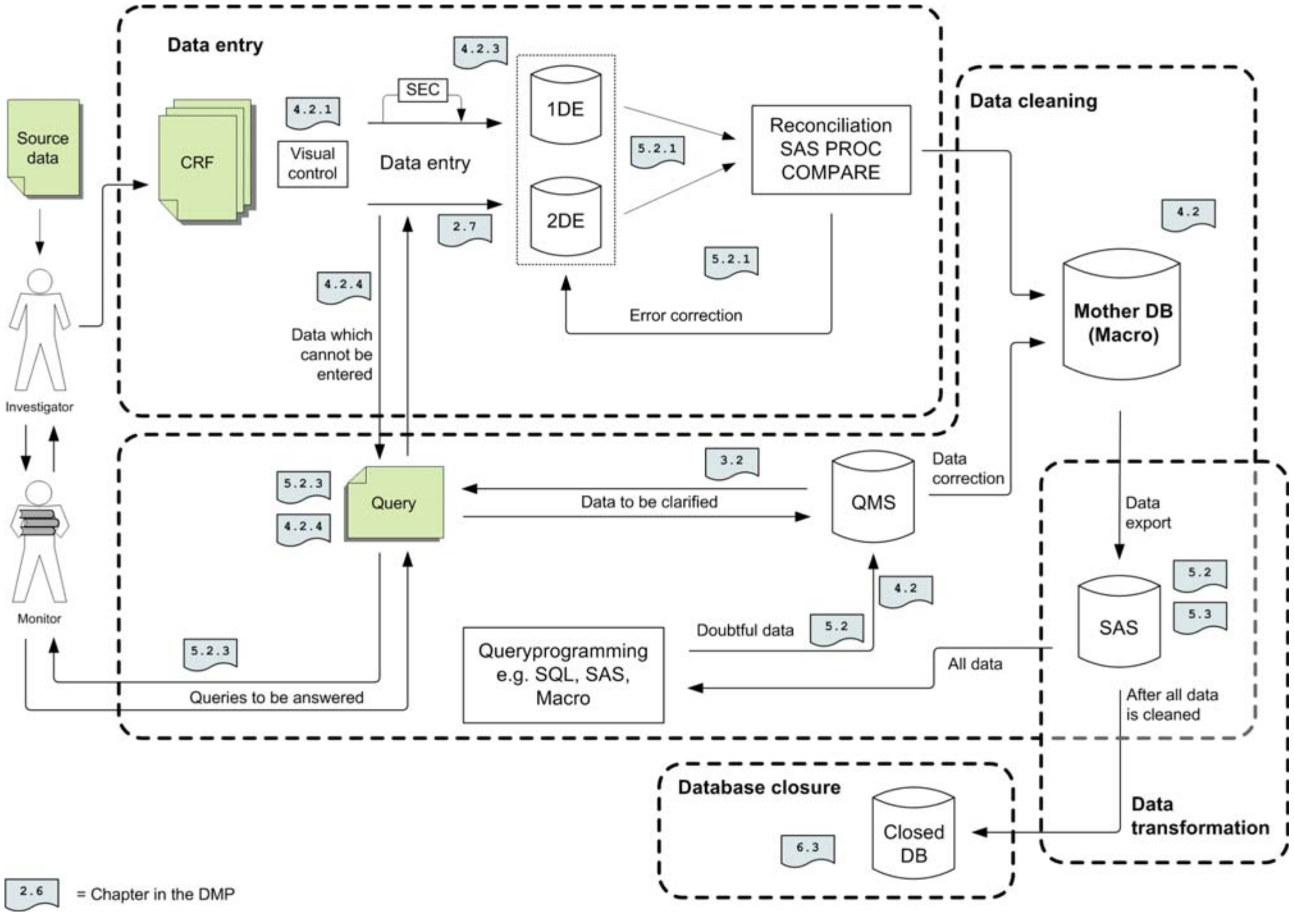

Figure 1: Overview of the processes in the data management.

2.5 Data Collection

2.5.1 Creation of CRF and CRF Data Processing

The CRFs will be designed by IZKS Mainz on basis of the trial protocol and in cooperation with the trial monitor and the investigator. The IZKS Mainz standards for the development of CRFs will be used. The principle investigator will sign a CRF Release Documentation to show his approval with the finalised CRF.

Completed CRF-pages will be reviewed by a monitor. Any finding will be resolved at the site with the help of the investigator before the CRFs will be transferred to the IZKS for data entry.

2.6 Creation of the Data Entry Application and the Database

Data collected on paper CRFs will be entered into a database by data entry personnel at IZKS Mainz. The design of the data entry application and the database is based on the CDM database system MACRO. Data should be entered unchanged whenever possible. Therefore no data entry checks will be implemented with the possible exception of checks on key identifying variables to

ensure, that entered data match the right patient. To develop the trial specific data entry application and the database variable names and possible data entry checks will be planned by means of a List of Variables. Before the application and the database will be created and tested (validated) before being released for data entry.

2.7 Data Entry

2.7.1 CRF Data Entry

Paper CRFs are used for the primary capture of trial data and will then be entered exactly into the database. To minimise errors during data transcription the data will be entered into two separate but identical databases by two independent data entry operators. Therefore a copy of the MACRO trial database will be created after its finalisation and named DAD_1DE and DAD_2DE. Data entry personnel will be instructed by the trial data manager and will follow the Data Entry Instructions. Data entry will only be allowed by entering a user-specific username and a user specific password. All non-textual data will be entered by two independent data entry operators. Textual data will be entered only in DAD_1DE and cross-read by a second data entry operator. After termination of data entry both databases will be compared and possibly existing differences will be solved.

2.7.2 Medical Coding

In this trial medical coding of the following item groups will be performed by IZKS Mainz according to the referenced international dictionaries.

- Adverse events: MedDRA
- Concomitant medication: Rote Liste
- Concomitant diseases: MedDRA

The medical codes/decodes will be added to the SAS database. All coding activities are subject to quality securing measures.

2.8 Data Validation

Data validation is performed at all stages in the course of the trial. A detailed description of data validation processes is subject to chapter 3, 4 and 5.

The programmes of the automated plausibility checks are the main tool for data validation. The programmes will run over the database several times to discover missing or questionable data.

2.9 Query Process and Data Correction

Queries, requests to the investigator due to missing or questionable data, arise at different stages in the course of the trial. They can result from automated plausibility checks or from manual processes (e.g. monitor review, data entry, medical coding) and will be managed via the Query Management System (QMS) developed at IZKS Mainz.

2.10 Locking of the Trial Database

As soon as all findings have been processed the MACRO database will be closed. Likewise will the SAS database be closed after completion of the data validation process and external data has been appended.

The database will then be passed to the biometricians for analysis.

3 DATABASE AND PATIENT DATA VALIDATION STRATEGY

Please note that the word “database” can be used in the meaning of the structure that describes and holds data as well as in the meaning of the entirety of data within the structure. As that may lead to confusion “data” or “patient data” will in future be used for the entirety of data and “database” will only be used in the meaning of the structure.

Patient data may only be processed using validated computer applications as obliged by ICH-GCP. Therefore all applications used must be validated this includes the database build for the study.

Patient data validation is a central aspect in order to detect errors related to patient data in the course of a trial. Errors that arise during data entry and implausible electronic data can be reduced by data validation. The intensity of data validation is a trade-off between desired data quality and accepted effort.

3.1 Data Validation Plan

The Data Validation Plan (DVP) verbalises conditions of consistency, completeness and accuracy the patient data has to satisfy. Most of the conditions will be converted into SAS programs and are then called plausibility checks. The patient data will be checked by means of the plausibility checks. Conditions that cannot be handled by SAS programs will be checked manually. Findings (= Queries) will be managed via the Query Management System (see also the following chapter 3.2 Query Management System (QMS)). Corrections will be performed in the MACRO database and checked by an independent person. Both, corrections and checks, will be documented.

3.2 Query Management System (QMS)

All manual and automated queries arising in the course of the trial will be managed by the QMS developed at IZKS Mainz. Before the query processing is started a trial specific query database will be initialised by the QMS and trial specific information like address data of the trial sites will be imported. Requests resulting from automatic or manual processes will be imported or entered into the QMS where it will be completed by identifying data as the patient number and the trial site. Furthermore a processing status is assigned to each query which will be adapted for each processing step (e.g. when the query has been sent the status is “Query sent“, when the query has got back the status may be “Returned: resolvable investigator“). Answers to queries will not be entered in the QMS as well as information whether data in the database has been changed as the answered query stated.

4 VALIDATION PROCESSES PRIOR AND DURING DATA ENTRY

4.1 Creation of Database and Data Entry Screens

Data will be collected on paper CRFs and entered into the trial database by data entry personnel. The tool which includes the entry screens as well as the database is based on the CDM database system MACRO. The tool will be designed according to the specifications made in the trial protocol and the intention to map the paper CRFs. All necessary variables will be planned beforehand in the List of Variables. The development will follow the standards of the IZKS Mainz including quality assurance tests.

Access rights for the trial database assigned to persons involved in the trial database will be reported in the Access Rights Log. All persons provided with access rights will be trained in the correct handling of the data entry application. The training will be documented in the Data Entry Instruction Certification.

4.2 Data Validation

4.2.1 Visual Control of Completed CRF Pages

After the CRF review by the monitoring staff a data manager will roughly check the completed CRF pages in order to detect CRF-completion problems or bias. This process can be substituted by a realtime monitoring or a close inspection of data entry.

4.2.2 Data Correction Processes

In paper based studies no data entry checks will be integrated as the data should be entered as unchanged as possible into the trial database. Data validation will be accomplished separately from data entry with the following three exceptions:

- Self-evident corrections by data entry staff
- Self-evident corrections by CDM staff
- Clarification of data which cannot be entered as reported on the CRF for different reasons

4.2.3 Handling of Self-Evident Corrections (during data entry)

Self-evident corrections will be conducted during data entry by data entry personnel. Data entry personnel will be instructed in which cases they are authorized to enter data divergently from the way documented in the CRF without initiating a query to the investigator. All data that has been entered changed will be documented in the log of self-evident corrections by data entry personnel and additionally signed by a responsible data manager and the responsible investigator.

4.2.4 Data Correction by Manual Query

Illegibility of CRF data or other reasons why transferring CRF data into the trial database is impossible require data clarification. If so data entry staff will specify the problem so the data manager can ask the investigator for clarification by use of a manual query.

5 VALIDATION PROCESSESS AFTER DATA ENTRY

5.1 SAS Database Validation

All SAS programmes developed within the scope of the trial will be validated before their application. Every programme will be validated according to its classification of risks but in any case by a person different of the programmer. More details of the validation process are given in the most recent version of SOP (SOP = Standard Operation Procedure) BIM06 “Validierung von SAS-Programmen” of IZKS Mainz.

5.2 Data Validation

5.2.1 SAS-Compare of Double Entered Data

After all data have been entered, both MACRO trial databases (DAD_1DE and DAD_2DE) will be exported to SAS, where the data of both databases will be compared. The comparison affects only double entered data. Any conflicts arising will be resolved by the data manager using the original CRF sheets. If the conflicts arising out of the compare can not be resolved queries will be generated. Possible data corrections will be captured and the resolved query will be filed to the corresponding CRF. Self evident corrections will be reviewed and confirmed via signature by the investigator.

5.2.2 Visual Check of Single Entered Data

Textual data will be visually cross-read against the CRF by staff other than data entry personnel. The persons involved will be familiar with medical terminology.

5.2.3 Query Process and Data Correction

Queries, requests to the investigator due to missing or questionable data, arise at different stages in the course of the trial. They can result from automated plausibility checks or from manual processes (e.g. monitor review, data entry, medical coding) and will be managed via the Query Management System (QMS) developed at IZKS Mainz. A printed version of the queries will be sent to the appropriate trial site.

The query sheets include the request and for each query a space for the investigators answer. Answered queries will be sent back to IZKS Mainz where data corrections on basis of these queries will be conducted by data entry personnel. The correct transfer of the answers of all manual queries will be verified by a member of CDM via the audit trail where all changes of data in the database are documented. Results of these checks can be found in the Database Verification Log. After the answers have been worked in the status of each query will be updated in the QMS. The query sheets will be filed to the corresponding CRF for archiving. A copy of the query sheets should remain at the trial side.

The programmes of the automated plausibility checks will run several times over the database until no more unresolved or new findings are discovered. Subsequently there will be several cycles of queries in the course of a trial.

5.2.4 Missing Information

Procedures dealing with missing data will be determined in advance.

Questions that need to be answered in any case will be specified in the DVP. Missing values of these variables lead to queries and will be resolved by the investigator.

5.2.5 Handling of Self-evident Corrections (after data entry)

Self-evident corrections after data entry will be conducted by CDM staff. Cases in which the CDM staff is authorized to change data without asking the investigator for clarification by use of a query will be strictly limited to those cases where the correction is doubtlessly clear. All data that has been changed will be documented in the log of self-evident corrections by the responsible data manager and will be additionally signed by the responsible investigator.

5.3 Medical Coding

5.3.1 Adverse Events

The international coding dictionary MedDRA available at IZKS Mainz will be used for encoding and classifying reported verbatim medical terms of adverse events. Coding of adverse events will be performed by IZKS Mainz on the “Preferred Term” (PT) and/ or “System Organ Class” (SOC) level. All terms will be coded using one version of the coding dictionary. The version will be mentioned in the DMR.

Auto-encoding procedures match the reported terms to dictionary terms. Terms that cannot be coded automatically will be coded manually by a medical coder at IZKS Mainz.

Terms which cannot be coded will be collected and submitted to the principle investigator for signature.

5.3.2 Concomitant Medication

Concomitant medications will be encoded and classified by its reported verbatim terms using Rote Liste. All terms will be coded using one version of the coding dictionary. Categorisation will be performed according to active ingredients. This manual coding will be carried out under the responsibility of IZKS Mainz.

Terms which cannot be coded will be collected and submitted to the principle investigator for signature.

5.3.3 Concomitant Diseases

The international coding dictionary MedDRA will be used for encoding and classifying reported verbatim medical terms of concomitant diseases. Coding of concomitant disease will be performed by IZKS Mainz on the “Preferred Term” (PT) and/ or “System Organ Class” (SOC) level. All terms will be coded using one version of the coding dictionary. Terms which cannot be coded will be collected and submitted to the principle investigator for signature.

5.4 SAE Reconciliation

The SAE reconciliation enfolds the comparison between the SAE fax data and the AE data in the database. It will be performed by CDM of IZKS Mainz.

6 PROCESSES AFTER DATA VALIDATION

6.1 Database Audit

To verify the data capturing process a comparison of 20% of all items of the CRFs, respectively 100% of the primary endpoint parameter, versus the MACRO database items will be performed. Findings detected during the database audit will be corrected in the MACRO database audit documentation.

Items that have been visually checked earlier can be excepted form the database audit.

6.2 Database Closure

As soon as database validation processes are completed both the MACRO database and the SAS database will be closed and released for analysis. This process will be documented in the Database Closure Documentation.
